# Diverse ring-opening reactions of rhodium η^4^-azaborete complexes†

**DOI:** 10.1039/d0sc02283g

**Published:** 2020-08-04

**Authors:** Merlin Heß, Tom E. Stennett, Felipe Fantuzzi, Rüdiger Bertermann, Marvin Schock, Marius Schäfer, Torsten Thiess, Holger Braunschweig

**Affiliations:** Institute for Inorganic Chemistry and Institute for Sustainable Chemistry & Catalysis with Boron, Julius-Maximilians-Universität Würzburg Am Hubland 97074 Würzburg Germany h.braunschweig@uni-wuerzburg.de

## Abstract

Sequential treatment of [Rh(COE)_2_Cl]_2_ (COE = cyclooctene) with P^i^Pr_3_, alkyne derivatives and ^*t*^BuN≡BMes (Mes = 2,4,6-trimethylphenyl) provided functionalized rhodium η^4^-1,2-azaborete complexes of the form (η^4^-azaborete)RhCl(P^i^Pr_3_). The scope of this reaction was expanded to encompass alkynes with hydrogen, alkyl, aryl, ferrocenyl, alkynyl, azaborinyl and boronate ester substituents. Treatment of these complexes with PMe_3_ led to insertion of the rhodium atom into the B–C bond of the BNC_2_ ring, forming 1-rhoda-3,2-azaboroles. Addition of N-heterocyclic carbenes to azaborete complexes led to highly unusual rearrangements to rhodium η^2^,κ^1^-allenylborylamino complexes *via* deprotonation and C–N bond cleavage. Heating and photolysis of an azaborete complex also led to rupture of the C–N bond followed by subsequent rearrangements, yielding an η^4^-aminoborylallene complex and two isomeric η^4^-butadiene complexes.

## Abstract 2

Rhodium η^4^-azaborete complexes can be transformed into a variety of species with ring-opened, BN-containing ligands by treatment with Lewis bases.

## Graphical abstract

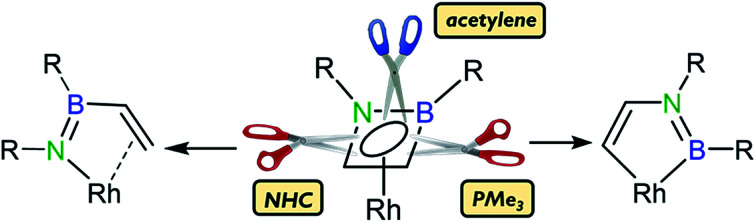

## Introduction

**Scheme 1:**
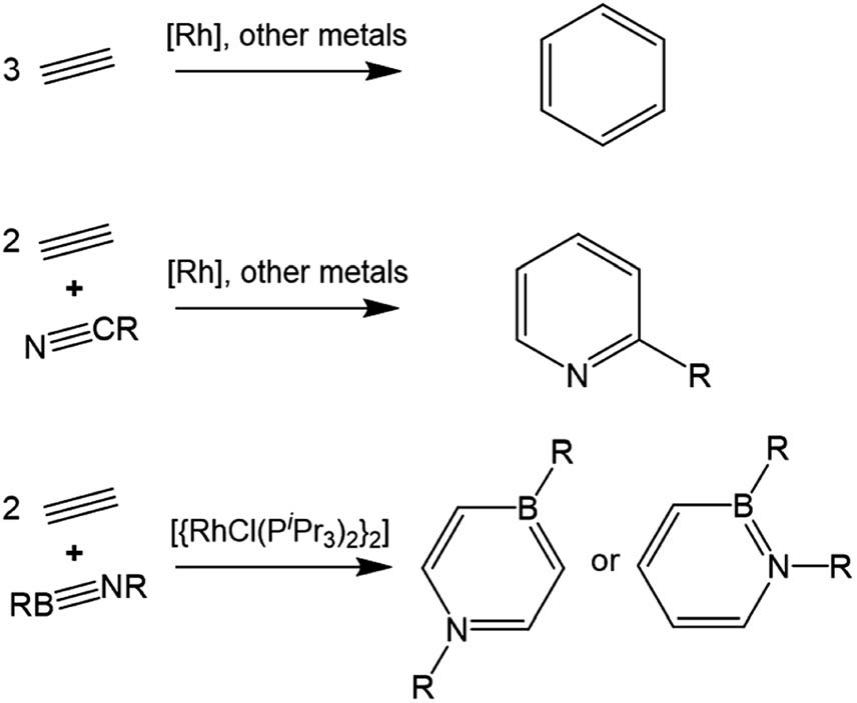
Simplified representation of the metal-catalyzed synthesis of (hetero)arenes by trimerization of alkyne derivatives.

Complexes of the late transition metals have long been known to catalyze the cyclotrimerization of alkynes to arenes (Scheme 1).^1–7^ Incorporation of nitriles under appropriate conditions can also provide an efficient route to substituted pyridines.^8^ Mechanistic studies on Rh-based catalyst systems indicate that two coordinated alkyne molecules undergo reductive coupling to produce a rhodacyclopentadiene intermediate, from which a further alkyne/nitrile insertion step and subsequent reductive elimination yields the arene.^1,9,10^ In 2012, our group exploited the isolobal relationship between alkynes and iminoboranes to prepare a 1,4-azaborinine by an analogous catalytic cyclization of two equivalents of alkyne and one of the iminoborane ^*t*^BuN≡B^*t*^Bu.^11^ Shortly afterwards it was established that replacing acetylene with the bulkier ethynylferrocene affords a 1,2-azaborinine rather than the 1,4-isomer,^12^ while use of ^*t*^BuN≡BMes as the iminoborane unit was also found to produce 1,2-azaborinines with a range of alkynes.^13^ These BN isosteres of common aromatic compounds are of interest for biomedical and materials applications.^14,15^ Employing bridged di- or trialkynes also allowed the preparation of the respective bis- and tris-azaborinine products.^16^ Both of these reaction types appear to proceed *via* rhodium-bound η^4^-1,2-azaborete intermediates, which can be isolated from stoichiometric reactions. The divergent regioselectivity of the subsequent reactions with a second equivalent of alkyne indicates that the azaborete can undergo ring opening *via* cleavage of either the B–N bond (leading to 1,4-azaborinines) or the B–C bond (leading to the 1,2-isomer).

It should be emphasized that the intermediacy of azaboretes in these reactions stands in sharp contrast to the reactions of all-carbon analogues in this regard – whereas five-membered metallacyclopentadiene derivatives^17^ are believed to be key reaction intermediates in the trimerization of alkynes, the corresponding η^4^-cyclobutadiene complexes^18,19^ resulting from reductive elimination are highly stable and thought to be catalyst deactivation products rather than intermediates.^9,20^ In this work, we report the synthesis of a range of new rhodium 1,2-azaborete complexes by alkyne/iminoborane coupling and explore their reactivity towards neutral Lewis bases. Our results reveal that both the B–C and N–C bonds of the azaborete can be ruptured, depending on the donor species employed.

## Results and discussion

**Scheme 2:**
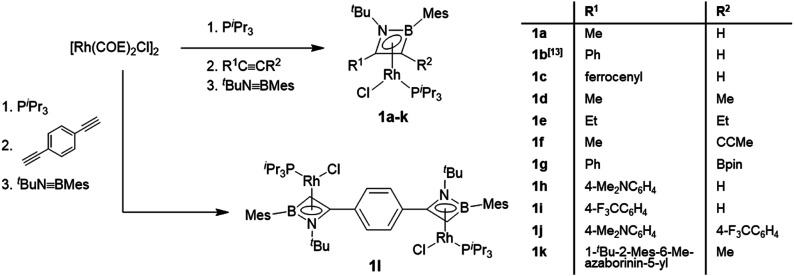
Synthesis of rhodium η^4^-azaborete complexes.

The rhodium η^4^-azaborete complexes **1a–l** were prepared according to a procedure adapted from a previously published route (Scheme 2).^12^ The complex [Rh(COE)_2_Cl]_2_ (COE = cyclooctene) was subsequently treated with P^i^Pr_3_, an alkyne derivative and ^*t*^BuN≡BMes in a one-pot synthesis, giving the complexes as yellow/orange solids after workup. The ^11^B NMR chemical shifts fall within a narrow range (20.3–24.8 ppm), indicating that the influence of the alkyne substituents on the ring electronics is relatively small. The ^31^P NMR signals appear between 48 and 61 ppm, with characteristic ^1^*J*_Rh-P_ coupling constants of around 195 Hz. A wide variety of alkyne substituents can be employed, including electron-donating (Me, Et, 4-Me_2_NC_6_H_4_) and electron-withdrawing groups (4-F_3_CC_6_H_4_, Bpin), as well as one example with an azaborininyl substituent, prepared by the aforementioned rhodium-mediated protocol. The diyne 1,4-diethynylbenzene could also be employed to afford bis(η^4^-1,2-azaborete) complex **1l**. Despite the multiple reaction steps, the yields of complexes **1** were generally high (60–89%), with the exception of **1l** (31%), where impurities with similar solubility hampered the workup. The crystallographically determined bond lengths of the azaborete ring in compounds **1a–l** are remarkably similar across the series (see ESI†).

### Reactions with phosphines

**Scheme 3:**
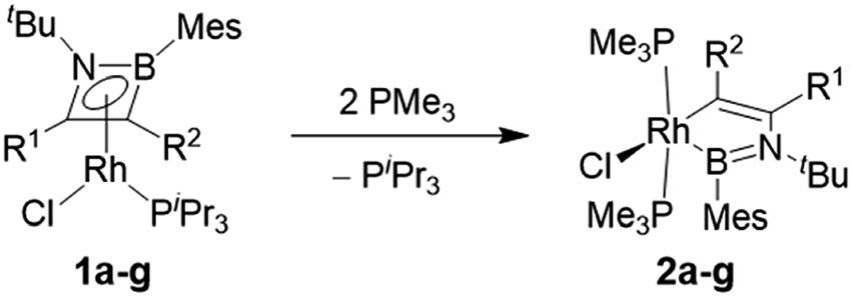
Synthesis of 1-rhoda-3,2-azaborole complexes. See Scheme 2 for R-group definitions.

**Fig. 1:**
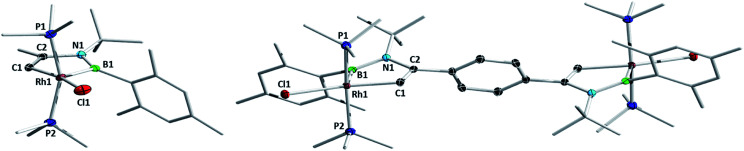
Molecular structures of complexes **2a** (left) and **2l** (right) with atomic displacement ellipsoids at the 50% probability level. Co-crystallized solvent molecules and hydrogen atoms are not shown. Selected bond lengths (Å): **2a**: Rh1–B1 2.025(2), B1–N1 1.426(2), N1–C2 1.441(2), C2–C1 1.335(2), C1–Rh1 1.999(2), Rh1–P1 2.2889(8), Rh1–P2 2.3082(8); **2l**: Rh1–B1 2.021(3), B1–N1 1.434(3), N1–C2 1.435(2), C2–C1 1.340(4), C1–Rh1 2.009(2), Rh1–P1 2.2956(9), Rh1–P2 2.3083(7).

The computationally predicted mechanism for the formation of 1,4-di-*tert*-butyl-2-phenyl-1,4-azaborinine from its respective [RhCl(η^4^-azaborete)(P^i^Pr_3_)] complex and acetylene involves formation of a π-complex between the incoming acetylene molecule and rhodium.^16^ Acetylene inserts into the azaborete ring by attacking the boron atom, causing subsequent cleavage of the B–N bond, whereas in the case of 1,2-azaborinines the B–C bond is broken. We hoped to gain evidence for intermediate species in these reactions by treating azaborete complexes with Lewis bases that are less susceptible to engaging in further reactivity, such as trialkylphosphines and N-heterocyclic carbenes. Reaction of compound **1a** with 2.2 equivalents of PMe_3_ at room temperature in benzene solution led to a new compound with a doublet signal in the ^31^P NMR spectrum at −4.7 ppm (^1^*J*_Rh-P_ = 130 Hz) alongside free P^i^Pr_3_. Conversion was complete within one hour. The sole peak at 67.8 ppm in the ^11^B NMR spectrum indicated a dramatic change in the boron environment. Removal of volatiles and trituration with hexane gave 1-rhoda-3,2-azaborole complex **2a** in 70% yield (Scheme 3). Crystals grown from hexane allowed the unambiguous identification of the compound by X-ray diffraction (Fig. 1). The geometry at rhodium can be described as a distorted square-based pyramid, with the phosphines located *trans* to one another and capped by the boryl ligand. The 1-rhoda-3,2-azaborole ring is slightly distorted from planarity, while the C1–C2 (1.335(2) Å) and B–N (1.426(2) Å) bond lengths are in the range of double bonds. The Rh–B distance (2.025(2) Å) falls in the middle of the range for known rhodium boryl complexes.^21,22^ The only similar structure in the literature is that of an osmium aminoboryl complex bearing a five-membered osmacycle unit,^23^ which was prepared by reaction of an osmium tetrahydroborate complex with two equivalents of aniline. The carbon atoms in the ring form part of a C–H activated aryl group, and as such, the bonding situation is not directly comparable to that in **2a**.

Compounds **1b** and **1c** both reacted with PMe_3_ in an identical fashion within 1 h to yield the corresponding 1-rhoda-3,2-azaborole complexes in good yields. Complexes **1d–f**, bearing hydrocarbon substituents at both carbon atoms of the azaborete ring, required longer reaction times (up to 4 h), but nonetheless produced good yields (79–87%) of the corresponding 1-rhoda-3,2-azaborole complexes **2d–f**. Boryl-substituted complex **1g** could also be converted to its corresponding rhoda-3,2-azaborole, **2g**, although in this case, the reaction was hampered by low reactivity and formation of side-products, necessitating a reaction time of four days and the use of a larger excess of PMe_3_ (4.4 eq.) and limiting the yield to 35%.

Azaborinine derivative **1k** did not react with PMe_3_ at room temperature, whereas heating led to an unselective reaction, with only traces of the intended product observed in the NMR spectra. Bis(azaborete) complex **1l** was converted cleanly to rhoda-3,2-azaborole **2l** (Fig. 1) after 15 h at room temperature, with a reasonable isolated yield of 60%. Notably, complexes **2a–g** and **2l** remain 16-valence-electron (VE) Rh(iii) species, and do not bind the excess phosphine present in the reaction mixture. A series of related, carbon-based rhodacyclopentadiene complexes reported by Marder and co-workers retain three PMe_3_ ligands and one chloride ligand, making them 18 VE species.^24^ While this observation could be related to the steric pressure caused by the boron-bound mesityl group, the strong *trans* influence of boryl groups^25,26^ is also expected to disfavour coordination of a further PMe_3_ ligand.

Given the somewhat unexpected formation of **2a**, we undertook DFT calculations to investigate the mechanism of this reaction. We used a free energy map of proposed intermediates to identify the most feasible pathway for the ring opening process. These results (see ESI† for details) clearly show that association of a further phosphine ligand must occur prior to insertion of the Rh atom into the B–C bond of the azaborete. The thermodynamically most plausible mechanism involves endergonic coordination of PMe_3_ to **1a** (Δ*G* = 7.8 kcal mol^−1^), followed by dissociative substitution of the remaining P^i^Pr_3_ ligand by PMe_3_. The intermediate [RhCl(η^4^-azaborete)(PMe_3_)_2_] (0.4 kcal mol^−1^ less stable than **1a**), all-carbon cyclobutadiene derivatives of which can be isolated with chelating diphosphines,^19^ undergoes ring-slippage to the 16-VE species [RhCl(η^2^-azaborete)(PMe_3_)_2_], with the azaborete now coordinated *via* the B–C bond and mutually *trans* phosphine ligands. This species is then set up to undergo oxidative ring opening to form **2a** (9.1 kcal mol^−1^ more stable than **1a**). A transition state was identified for the B–C bond cleavage step, indicating that this process has a remarkably low barrier (2.3 kcal mol^−1^). Direct ring opening of the starting complex would produce a hypothetical four-coordinate, 14-VE [RhCl(κ^2^-BNCC)(P^i^Pr_3_)] intermediate that is 20.4 kcal mol^−1^ higher in energy than **1a**, and thus unlikely to be accessible under the mild conditions employed in the reaction.

We subsequently performed a number of NMR-scale test reactions with different phosphines, which can be summarised as follows: PEt_3_ reacts with **1a** similarly to PMe_3_, producing rhoda-2,3-azaborole complex [RhCl(κ^2^-BNCC)(PEt_3_)_2_] (**2a(PEt3)**); P^i^Pr_3_ leaves compound **1a** unchanged (although a dynamic process of ligand exchange may occur); PCy_3_ results in partial substitution of P^i^Pr_3_ upon heating to 80 °C, with formation of [RhCl(η^4^-azaborete)(PCy_3_)] (**1a(PCy3))** in an equilibrium that can be driven to the product side by repeated removal of P^i^Pr_3_ under vacuum, but no ring opening was observed. We therefore propose that associative phosphine exchange at Rh in **1a** is facile, but that bulkier phosphines favour reversion to low-coordinate complexes of the form **1a**. Small phosphines stabilise intermediates [RhCl(η^4^-azaborete)(PR_3_)_2_], which can then undergo ring slippage and irreversible ring opening to form complexes **2a**.

It is notable here that the calculated mechanism for the formation of 1,4-di-*tert*-butyl-2-phenyl-1,4-azaborinine by reaction of acetylene with [RhCl(η^4^-1,2-di-*tert*-butyl-4-phenyl-1,2-azaborete)(P^i^Pr_3_)] does not proceed *via* a rhoda-3,2-azaborole intermediate.^16^ Although the initial step of coordination of the incoming Lewis base (alkyne or phosphine) is the same, the barrier to insertion of the alkyne into the B–Rh bond, which was calculated to be only 5 kcal mol^−1^, appears to be lower than that of oxidative addition of the B–C bond to the metal in this case. Nonetheless, it is quite plausible that azaborole complexes of the form **2** are intermediates in the formation of 1,2-azaborinines from 1-*tert*-butyl-2-mesityl-1,2-azaboretes, and we are continuing to investigate this computationally.

### Reactions with N-heterocyclic carbenes

**Scheme 4:**
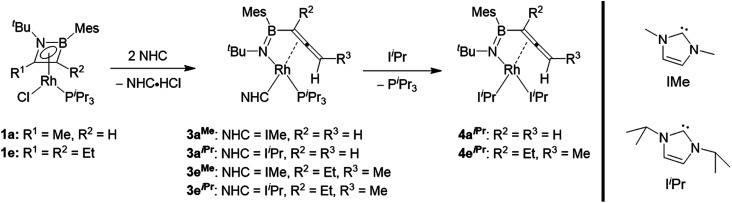
Reaction of azaborete complexes with N-heterocyclic carbenes.

**Fig. 2:**
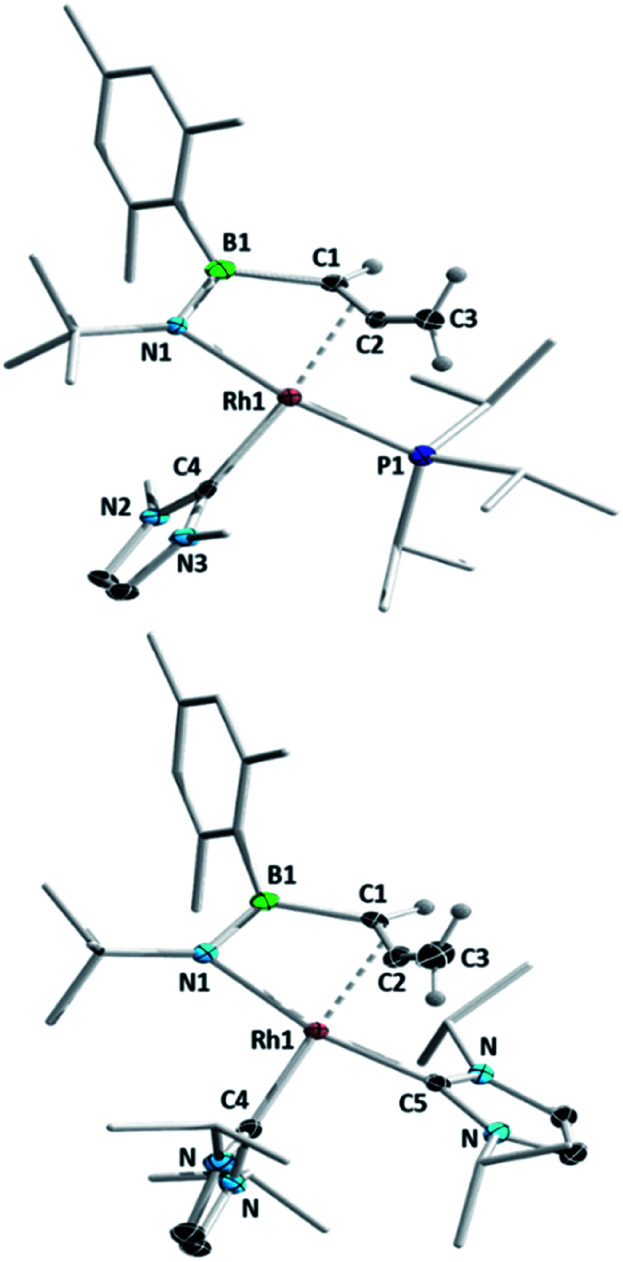
Molecular structures of complexes **3aMe** (above) and **4aiPr** (below) with atomic displacement ellipsoids at the 50% probability level. Hydrogen atoms other than those in the allene unit are not shown. Selected bond lengths (Å) and angles (°) for **3aMe**: Rh1–N1 2.110(3), Rh1–B1 2.525(4), Rh1–C1 2.182(3), Rh1–C2 2.071(3), N1–B1 1.397(5), B1–C1 1.573(6), C1–C2 1.382(5), C2–C3 1.304(4), Rh1–P1 2.280(1), Rh1–C4 2.050(3), C1–C2–C3 150.8(3); for **4aiPr**: Rh1–N1 2.092(1), Rh1–B1 2.516(2), Rh1–C1 2.183(2), Rh1–C2 2.034(2), N1–B1 1.393(2), B1–C1 1.575(2), C1–C2 1.388(3), C2–C3 1.319(3), Rh1–C4 2.059(2), Rh1–C5 2.012(2), C1–C2–C3 148.8(2).

We subsequently investigated the reactivity of the azaborete complexes towards N-heterocyclic carbenes. Addition of three equivalents of the small NHC IMe (1,3-dimethylimidazol-2-ylidene) to a benzene solution of **1a** led to formation of a colourless precipitate within 20 minutes at room temperature, which was filtered off and identified as 1,3-dimethylimidazolium chloride by ^1^H NMR spectroscopy. The ^11^B NMR spectrum of the filtrate revealed a major signal at 33.6 ppm. The predominant signal in the ^31^P NMR spectrum was a doublet at 48.8 ppm with a ^1^*J*_Rh-P_ coupling constant of 140 Hz, with a number of minor by-products including free P^i^Pr_3_ also present. After stirring for 15 h and subsequent work-up, the major species could be isolated as a yellow solid, which was identified by X-ray diffraction as complex **3aMe** (Scheme 4, Fig. 2), in 40% yield. Here, the N–C bond of the azaborete ligand had been cleaved, while the CH_3_ ring substituent had been deprotonated, forming an allene. Coordination of the η^2^,κ^1^-allenylborylamino fragment to rhodium occurs through the nitrogen atom and the internal carbon–carbon π bond. Although the B–N distance of 1.397(5) Å is firmly in the range of a double bond, the long B–Rh distance of 2.525(4) Å rules out any bonding interaction between these atoms. The C1–C2 distance of 1.382(5) Å is that of an elongated double bond, consistent with significant backbonding from the electron-rich Rh(i) centre. The C2–C3 distance is comparatively short, at 1.304(4) Å, whereas the C1–C2–C3 angle of 150.8(3)° indicates considerable deviation from linearity, the distal CH_2_ group being tilted away from the Rh centre. These observations correlate well with allene complexes in the literature with moderately electron-rich metal fragments.^27^

Reaction of the bulkier NHC I^i^Pr (1,3-di*iso*propylimidazol-2-ylidene) with **1a** initially led to similar results to the IMe case, with observation of imidazolium chloride formation and similar NMR signals (*δ*(^11^B) = 33 ppm, *δ*(^31^P) = 46.8 ppm, ^1^*J*_Rh-P_ = 138 Hz) after 20 minutes. However, here we observed larger quantities of P^i^Pr_3_ in the ^31^P NMR spectrum, while a small amount of **1a** also remained unreacted. Increasing the amount of I^i^Pr to 3.3 molar equivalents gave gradual conversion over 6 days at room temperature to the doubly NHC-substituted complex **4aiPr**, whose structure was unambiguously identified by X-ray diffraction (Fig. 2). The bond lengths and angles of the BNC_2_ ligand are not significantly different to those in **3aMe**. The (P^i^Pr_3_)(I^i^Pr) complex **3aiPr** is evidently formed as an intermediate before substitution of the P^i^Pr_3_ ligand by a third equivalent of NHC. Whereas **4aiPr** could be isolated as a pure compound, attempts to isolate **3aiPr** were hampered by contamination with **4aiPr**. A single crystal X-ray diffraction experiment nonetheless confirmed the identity of **3aiPr**, with the structural parameters of the allenylborylamino ligand not significantly altered from those in **3aMe**. This reactivity stands in contrast to the reaction with IMe, where use of excess NHC increased the amount of free phosphine observed, but significant conversion to the putative bis(NHC) complex **4aMe** could not be achieved. We tentatively ascribe the preferential formation of the bulkier complex **4aiPr** to stabilizing dispersion interactions between the ligands.

A terminal methyl group is not necessary for the conversion of the azaborete ligand to an allene. Reaction of 3,4-diethyl-1,2-azaborete complex **1e** with IMe and I^i^Pr led to the η^2^,κ^1^-allenylborylamino complexes **3eMe** and **3eiPr**/**4eiPr**, respectively, in an almost identical fashion to **1a**. Complex **3eMe** is formed rapidly at room temperature, while **3eiPr** requires 4 h for completion, presumably due to steric hindrance. As with the conversion of **3aiPr** to **4aiPr**, a longer reaction time (4 days) was necessary to achieve complete conversion to the bis(NHC) complex **4eiPr**, which was isolated and fully characterized.

**Scheme 5:**
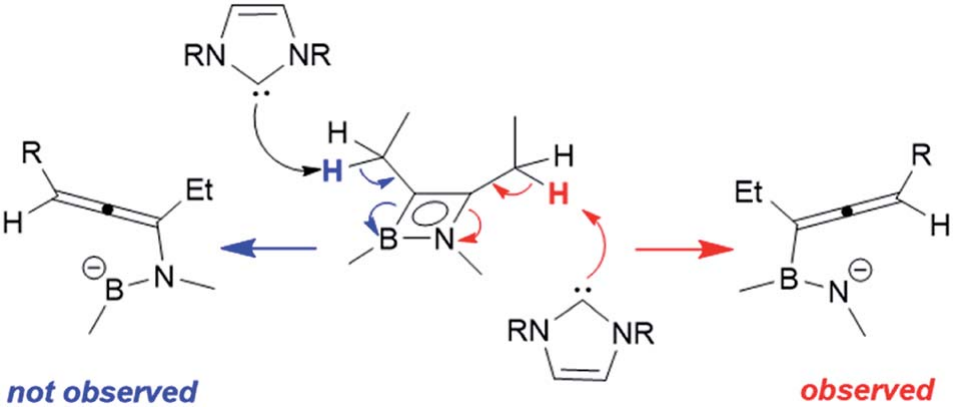
Proposed explanation for the regioselective formation of **3eMe** and **3eiPr**.

Rhodium-mediated conversions of alkylacetylenes to allenes are known, proceeding *via* a β-hydride shift to the metal centre.^28,29^ In light of the fact that the reactions with phosphines do not cause a H-shift, and that a different bond of the azaborete is broken, we propose that the more basic NHC is able to directly deprotonate the pendent alkyl group. Whereas **1a** only has one β-hydrogen site for deprotonation, the regioselectivity of the reaction of **1e** to compounds **3e** can be explained by the increased acidity of these protons due to a resonance structure (Scheme 5) bearing a negative charge at nitrogen (as opposed to at boron). Treatment of compound **1b**, which does not possess any β-hydrogen atoms, with one equivalent of IMe does not lead to ring opening. Instead, clean phosphine substitution gives the complex [(η^4^-azaborete)RhCl(IMe)] (**1b(IMe)**, characterised in solution by NMR) alongside free P^i^Pr_3_, suggesting that the NHC does not directly cause cleavage of one of the ring bonds *via* coordination to Rh as an initial step.

**Scheme 6:**
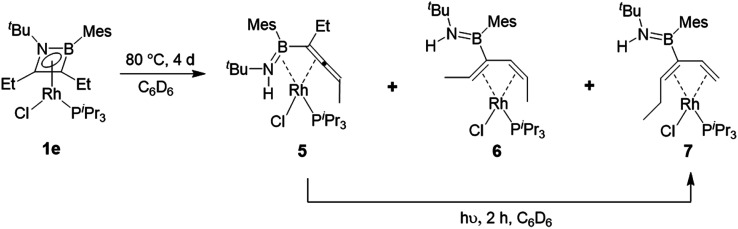
Thermal and photolytic rearrangements of η^4^-1,2-azaborete complex 1e.

**Fig. 3:**
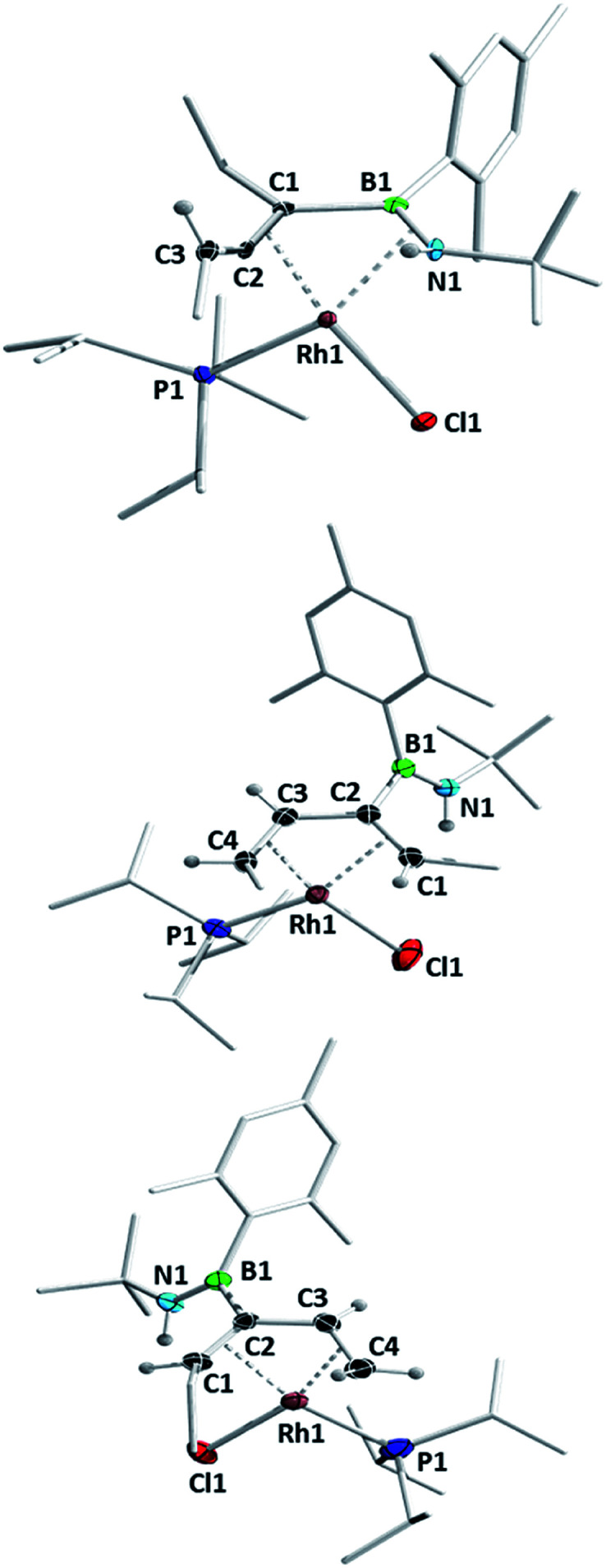
Molecular structures of complexes **5** (top) and **6** (middle) and **7** (bottom) with atomic displacement ellipsoids at the 50% probability level. Selected hydrogen atoms are shown. Selected bond lengths (Å) and angles (°) for **5**: Rh1–N1 2.260(2), Rh1–B1 2.342(3), Rh1–C1 2.157(2), Rh1–C2 2.031(3), N1–B1 1.451(4), B1–C1 1.559(4), C1–C2 1.398(4), C2–C3 1.328(4), Rh1–P1 2.288(1) C1–C2–C3 148.3(3); for **6**: Rh1–C1 2.316(3), Rh1–C2 2.203(2), Rh1–C3 2.084(3), Rh1–C4 2.132(3), C1–C2 1.398(4), C2–C3 1.454(5), C3–C4 1.416(4), C2–B1 1.597(4), B1–N1 1.383(5), Rh1–P1 2.292(1); for **7**: Rh1–C1 2.29(1), Rh1–C2 2.15(1), Rh1–C3 2.098(9), Rh1–C4 2.15(1), C1–C2 1.43(1), C2–C3 1.45(1), C3–C4 1.42(1), C2–B1 1.59(1), B1–N1 1.38(1), Rh1–P1 2.309(3).

Heating compound **1e** to 80 °C in the absence of any further Lewis base resulted in slow and somewhat unselective conversion over four days to three main species as judged by ^11^B and ^31^P NMR spectroscopy. Removal of volatiles and trituration of the residue with hexane produced a pale yellow solid with signals at 29.3 ppm in its ^11^B NMR spectrum and 53.9 ppm (^1^*J*_Rh-P_ = 181 Hz) in its ^31^P NMR spectrum. Crystals suitable for X-ray diffraction were obtained and confirmed the structure as that of complex **5** (Scheme 6, Fig. 3). In the absence of an external HCl acceptor, the amino nitrogen from the ring obtains a proton from the CH_2_ unit of the adjacent ethyl group. In contrast to the η^2^,κ^1^-allenylborylamino ligands in complexes **3** and **4**, the protonated amide results in the neutral aminoborylallene ligand adopting an η^4^ coordination mode. Schmid and co-workers prepared the only reported compounds of this type of acyclic η^4^-C=C–B=N ligand, as iron carbonyl complexes,^30^ but **5** is to the best of our knowledge the first crystallographically characterized example. The bonding parameters around the B=N moiety are markedly different to those in **4aiPr**. Most notably, the Rh–B distance is reduced from 2.516(2) Å to 2.342(3) Å, indicating a bonding interaction between these atoms in **5**. The lengthening of the Rh–N distance from 2.092(1) Å to 2.260(2) Å and of the B–N bond from 1.393(2) Å to 1.451(4) Å are also consistent with the switch from an amido ligand to a side-on coordinated aminoborane.

Concentration of the filtrate from this reaction produced X-ray quality crystals of one of the other products (Scheme 5, Fig. 3). Compound **6** (*δ*(^11^B) = 40 ppm, *δ*(^31^P) = 51.2 ppm, ^1^*J*_Rh-P_ = 187 Hz) is a further isomer of **1e**, in which the aminoborane moiety is completely dissociated from the rhodium centre, while the carbon chain has isomerized into a 2,4-hexadiene ligand coordinated in an η^4^ fashion to Rh. It appears to be the product of a formal 1,3-hydrogen shift from the remaining ethyl arm of compound **5** to the central allene carbon atom, followed by a shift in coordination mode. Bosch and Werner reported the related compound [Rh(C_4_H_6_)(P^i^Pr_3_)Br], although no crystallographic data was available for comparison.^31^

Hoping to prove the intermediacy of **5** in the formation of **6**, we further heated **5** to 80 °C in C_6_D_6_ solution overnight, but observed little change in the NMR spectra. However, UV-irradiation with a mercury–xenon-vapour lamp for 2 h led to complete conversion to the third species from the original thermolysis of **1e**, with an ^11^B NMR signal at 39.9 ppm and a doublet at 47.6 ppm (^1^*J*_Rh-P_ = 169 Hz) in the ^31^P NMR spectrum, alongside traces of side products. Although the similar solubility of the main product and side products prevented isolation of the pure compound, slow evaporation of a pentane solution produced X-ray quality crystals of a species matching the main signals of the crude NMR spectra. Like **6**, compound **7** (Scheme 6, Fig. 3) is an η^4^-butadiene complex isomeric to **1e**, but in this case the rearrangement leads to a 3-aminoboryl-1,3-hexadiene ligand. This transformation likely involves at least two hydrogen atom shifts from the former ethyl group in **5**, although a shift of the aminoboryl group cannot be ruled out. This reaction again demonstrates the preference of rhodium to bond C=C rather than B=N units.

## Conclusions

In summary, we have described several diverse ring-opening reactions of rhodium η^4^-1,2-azaborete complexes, both by treatment with neutral Lewis bases and through forcing thermal or photolytic conditions. In combination with the previously observed transformation of 1,2-di-*tert*-butyl-1,2-azaborete complexes to 1,4-azaborinines *via* B–N cleavage in reactions with acetylene, these results show that three of the four bonds of azaborete rings can be broken by addition of the appropriate reagent. We are currently focussed on obtaining the BN-containing ligands described here as metal-free species.

## Conflicts of interest

There are no conflicts to declare.

## Supplementary Material

SC-011-D0SC02283G-s001

SC-011-D0SC02283G-s002
