# Supplementary material for: Diverse ring-opening reactions of rhodium η4-azaborete complexes
Source: Chem Sci. 2020 Aug 4;11(34):9134–40. doi: 10.1039/d0sc02283g (PMC8163374; doi:10.1039/d0sc02283g)
Supplement: SC-011-D0SC02283G-s001 [file SC-011-D0SC02283G-s001.pdf]

Electronic Supplementary Material (ESI) for Chemical Science.
This journal is © The Royal Society of Chemistry 2020

Supporting information for:

Diverse Ring-Opening Reactions of Rhodium η^4-Azaborete Complexes

Merlin Heß, Tom E. Stennett, Felipe Fantuzzi, Rüdiger Bertermann, Marvin Schock, Marius Schäfer, Torsten Thiess, Holger Braunschweig*

Institute for Inorganic Chemistry and Institute for Sustainable Chemistry & Catalysis with Boron, Julius-Maximilians-Universität Würzburg, Am Hubland, 97074 Würzburg, Germany

Table of contents

General considerations....2

Synthetic procedures....3

NMR spectra....34

UV-Vis spectra....97

Crystal structure determination....102

Computational details....115

Cartesian coordinates....120

References....151

General considerations

All manipulations were performed under an atmosphere of dry argon using glovebox or standard Schlenk line techniques. Deuterated solvents were dried over 4 Å molecular sieves and degassed by three freeze-pump-thaw cycles. All other solvents were dried by distillation from appropriate drying agents under an argon atmosphere and stored under argon over activated 4 Å molecular sieves. All NMR spectra were obtained from a Bruker Avance III HD 300 NMR spectrometer ($^{13}C\{^1H, ^{31}P\}$: 75.5 MHz), from a Bruker Avance I 400 NMR spectrometer (^1H: 400.6 MHz, $^{13}C\{^1H\}$: 100.6 MHz, ^{11}B: 128.5 MHz, $^{31}P\{^1H\}$: 162.2 MHz, ^{19}F: 376.5 MHz) or from a Bruker Avance I 500 NMR spectrometer (^1H: 500.1 MHz, $^1H\{^{31}P\}$: 500.1 MHz, $^{13}C\{^1H\}$: 125.8 MHz, ^{11}B: 160.5 MHz, $^{31}P\{^1H\}$: 202.5 MHz, ^{19}F: 470.6 MHz) at 298 K unless otherwise stated. Chemical shifts (δ) are provided in ppm and internally referenced to the carbon nuclei ($^{13}C\{^1H\}$, $^{13}C\{^1H, ^{31}P\}$) or residual protons (^1H, $^1H\{^{31}P\}$) of the solvent. ^{11}B, $^{31}P\{^1H\}$ and ^{19}F NMR spectra were referenced against external $BF_3{\cdot}Et_2O$, 85% H_3PO_4 or Cl_3CF, respectively. For higher-order spin systems of the $P(CH_3)_3$ groups N ($N = |{}^1J_{PC} + {}^3J_{PC}|$ or $|{}^2J_{PH} + {}^4J_{PH}|$) is given. UV/Vis absorption spectra were measured on a JASCO V-660 UV/Vis spectrometer or on a METTLER TOLEDO UV/Vis-Excellence UV5 spectrophotometer. High-resolution mass spectrometry data were acquired on a Thermo Scientific Exactive Plus Spectrometer in LIFDI or ASAP mode. Photoreactions were performed using a LOT-Quantum Design GmbH mercury-xenon vapor lamp (I = 19 A, U = 26 V).

Chemicals: [{$(COE)_2RhCl$}$_2$],[1] trimethylphosphine,[2] tri*iso*propylphosphine,[3] (*tert*-butylimino)mesitylborane,[4] *N*,*N*-dimethyl-4-[2-[4-trifluoromethyl)phenyl]ethynyl]-benzenamine,[5] ethynylferrocene,[6] 1,3-dimethylimidazol-2-ylidene (IMe),[7] 1,3-di*iso*propylimidazol-2-ylidene (I*i*Pr)[8] and **1b**[4] were synthesized according to modified literature procedures. [{$(P\textit{i}Pr_3)_2RhCl$}$_2$] was prepared in situ according to a modified literature procedure.[9] All other chemicals were purchased from either abcr, Acros, Sigma-Aldrich or TCI Chemical Co. and used without further purification.

The rhodium azaborete complexes **1a**, **1c**, **1d**, **1e**, **1f**, **1g**, **1h**, **1i**, **1j**, **1k** and **1l** were synthesized according to a standardized procedure adapted from a previously published route.[10] All manipulations (except the washing procedure) during the synthesis of the complexes **1a**, **1b**,[4] **1c**, **1d**, **1e**, **1f**, **1g**, **1h**, **1i**, **1j**, **1k** and **1l** were performed as far as possible with the exclusion of light.

Synthetic procedures

Abbreviations

Aza	azaborete (four-membered ring system)
Aza1	azaborinine (six-membered ring system)
Bpin	4,4,5,5-tetramethyl-1,3,2-dioxaborolanyl
br	broad
COE	cyclooctene
Cp	cyclopentadienyl
d	doublet
Et	ethyl
Fc	ferrocenyl
IiPr	1,3-di*iso*propylimidazol-2-ylidene
IMe	1,3-dimethylimidazol-2-ylidene
*i*Pr	*iso*propyl
m	multiplet
Me	methyl
Mes	mesityl = 2,4,6-trimethylphenyl
Ph	phenyl
q	quartet
s	singlet
sept	septet
t	triplet
*t*Bu	*tert*-butyl
THF	tetrahydrofuran
v	virtual

Synthesis of 1a

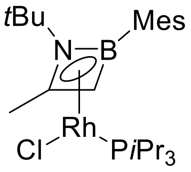

[{(COE)$_2$RhCl}$_2$] (1.00 g, 1.39 mmol) was suspended in pentane (15 mL) and tri*iso*propylphosphine (2.00 mL, 10.5 mmol) was added. After stirring the reaction mixture for 15 min, propyne was passed through the suspension for 2 min. All volatiles were removed *in vacuo* and the residue was dissolved in THF (15 mL). A stock solution of (*tert*-butylimino)mesitylborane in heptane (3.45 mL, 6.97 mmol, 2.02 M) was added and the reaction mixture was stirred for 15 h at room temperature. After removing all volatiles *in vacuo* the residue was washed with pentane (5 x 5 mL) and dried under reduced pressure to yield **1a** as a yellow solid (1.27 g, 2.35 mmol, 85%). Crystals of **1a** suitable for X-ray diffraction were obtained by evaporation of a saturated pentane solution at −30 °C.

^{1}H NMR (500.1 MHz, C_6D_6, 298 K): δ= 6.83 (s, 2H, Mes-C*H*), 3.39 (s, 3H, Mes-C*H*$_3$), 2.78 (s, 1H, Aza-C*H*), 2.54 (s, 3H, Mes-C*H*$_3$), 2.18-2.08 (m, 3H, *i*Pr-C*H*) overlapping with 2.14 (s, 3H, Mes-C*H*$_3$), 1.53 (s, 3H, Aza-C*H*$_3$), 1.34 (s, 9H, *t*Bu-C*H*$_3$), 1.12 (dd, $^3J_{PH}$ = 14.0 Hz, $^3J_{HH}$ = 7.3 Hz, 9H, *i*Pr-C*H*$_3$), 1.07 (dd, $^3J_{PH}$ = 13.3 Hz, $^3J_{HH}$ = 7.2 Hz, 9H, *i*Pr-C*H*$_3$) ppm.

^{1}H{^{31}P} NMR (500.1 MHz, C_6D_6, 298 K): δ= 6.83-6.82 (m, 2H, Mes-C*H*), 3.38 (s, 3H, Mes-C*H*$_3$), 2.78 (s, 1H, Aza-C*H*), 2.54 (s, 3H, Mes-C*H*$_3$), 2.17-2.09 (m, 3H, *i*Pr-C*H*) overlapping with 2.14 (s, 3H, Mes-C*H*$_3$), 1.53 (s, 3H, Aza-C*H*$_3$), 1.34 (s, 9H, *t*Bu-C*H*$_3$), 1.12 (d, $^3J_{HH}$ = 7.2 Hz, 9H, *i*Pr-C*H*$_3$), 1.07 (d, $^3J_{HH}$ = 7.2 Hz, 9H, *i*Pr-C*H*$_3$) ppm.

^{13}C{^{1}H} NMR (125.8 MHz, C_6D_6, 298 K): δ = 141.5 (s, Mes-C_q), 140.4 (s, Mes-C_q), 138.1 (s, Mes-C_q), 131.1 (bs, Mes-C_q), 128.5 (s, Mes-*C*H), 127.5 (s, Mes-*C*H), 103.8-103.7 (m, Aza-C_q), 56.0 (d, $^3J_{PC}$ = 1.5 Hz, *t*Bu-C_q), 49.0 (bs, Aza-*C*H), 29.0 (d, $^4J_{PC}$ = 2.9 Hz, *t*Bu-*C*H$_3$), 26.9 (d, $^4J_{RhC}$ = 0.5 Hz, Mes-*C*H$_3$), 25.2 (dd, $^1J_{PC}$ = 21.0 Hz, $^2J_{RhC}$ = 1.3 Hz, *i*Pr-*C*H), 23.7 (s, Mes-*C*H$_3$), 21.3 (s, Mes-*C*H$_3$), 20.3 (dd, $^2J_{PC}$ = 1.9 Hz, $^3J_{RhC}$ = 0.5 Hz, *i*Pr-*C*H$_3$), 19.6 (s, *i*Pr-*C*H$_3$), 19.6 (d, $^2J_{RhC}$ = 1.3 Hz, Aza-*C*H$_3$) ppm.

^{13}C{^{1}H, ^{31}P} NMR (75.5 MHz, C_6D_6, 298 K): δ = 141.5 (s, Mes-C_q), 140.4 (s, Mes-C_q), 138.1 (s, Mes-C_q), 128.5 (s, Mes-*C*H), 127.5 (s, Mes-*C*H), 103.9-103.7 (m, Aza-C_q), 56.0 (s, *t*Bu-C_q), 49.0 (bs, Aza-*C*H), 29.1 (s, *t*Bu-*C*H$_3$), 26.9 (d, $^4J_{RhC}$ = 0.6 Hz, Mes-*C*H$_3$), 25.2 (d,

$^{2}J_{RhC}$ = 1.5 Hz, *i*Pr-*C*H), 23.7 (s, Mes-*C*H$_3$), 21.3 (s, Mes-*C*H$_3$), 20.3 (d, $^{3}J_{RhC}$ = 0.7 Hz, *i*Pr-*C*H$_3$), 19.6 (d, $^{3}J_{RhC}$ = 0.6 Hz, *i*Pr-*C*H$_3$), 19.6 (d, $^{2}J_{RhC}$ = 1.4 Hz, Aza-*C*H$_3$) ppm.

Comment: The broad singlet corresponding to the Mes-C$_q$ nucleus bound to the boron atom could not be observed in the $^{13}C\{^{1}H, ^{31}P\}$ NMR spectrum.

^{11}B NMR (160.5 MHz, C_6D_6, 298 K): δ = 20.9 (br s) ppm.

^{31}P{^{1}H} NMR (202.5 MHz, C_6D_6, 298 K): δ = 61.0 (d, $^{1}J_{RhP}$ = 197 Hz) ppm.

HRMS (LIFDI, $C_{25}H_{45}BClNPRh$): *calcd*: m/z = 539.2121, *found*: m/z = 539.2116.

UV-vis (hexane): λ_{abs} = 240, 296 (shoulder), 383 nm.

Synthesis of 1c

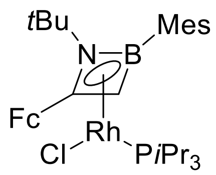

[{(COE)$_2$RhCl}$_2$] (400 mg, 557 µmol) was suspended in pentane (12 mL) and treated with tri*iso*propylphosphine (0.80 mL, 4.19 mmol). After stirring the suspension for 10 min, ethynylferrocene (233 mg, 1.11 mmol) was added. The reaction mixture was stirred for 10 min and all volatiles were removed *in vacuo*. The residue was dissolved in THF (10 mL) and treated with a stock solution of (*tert*-butylimino)mesitylborane in heptane (1.45 mL, 2.79 mmol, 1.93 M). The reaction mixture was stirred for 15 h at ambient temperature and all volatiles were removed *in vacuo*. The residue was washed with pentane (6 x 4 mL) and dried under reduced pressure to yield **1c** as a red solid (582 mg, 820 µmol, 74%). Crystals of **1c** suitable for X-ray diffraction were obtained by evaporation of a saturated hexane/benzene (5:1) solution.

^{1}H NMR (500.1 MHz, C_6D_6, 298 K): δ = 6.88 (s, 2H, Mes-C*H*), 4.440-4.435 (m, 1H, C_5H_4-C*H*), 4.35-4.34 (m, 1H, C_5H_4-C*H*), 4.09 (s, 5H, Cp-C*H*), 4.06-4.05 (m, 1H, C_5H_4-C*H*), 4.04-4.03 (m, 1H, C_5H_4-C*H*), 3.45 (s, 3H, Mes-C*H*$_3$), 3.17 (s, 1H, Aza-C*H*), 2.72 (s, 3H, Mes-C*H*$_3$), 2.24-2.16 (m, 3H, *i*Pr-C*H*) overlapping with 2.18 (s, 3H, Mes-C*H*$_3$), 1.56 (s, 9H, *t*Bu-C*H*$_3$), 1.15 (dd, $^{3}J_{PH}$ = 13.5 Hz, $^{3}J_{HH}$ = 7.2 Hz, 9H, *i*Pr-C*H*$_3$), 1.11 (dd, $^{3}J_{PH}$ = 13.5 Hz, $^{3}J_{HH}$ = 7.2 Hz, 9H, *i*Pr-C*H*$_3$) ppm.

Comment: The spectrum contains residual hexane from the crystallization process, corresponding to signals at 1.25 (m) and 0.89 (t) ppm.

$^{13}C\{^1H\}$ NMR (125.8 MHz, C_6D_6, 298 K): δ = 141.5 (s, Mes-C_q), 140.2 (s, Mes-C_q), 138.1 (s, Mes-C_q), 131.8 (s, Mes-C_q), 128.6 (s, Mes-*C*H), 127.5 (s, Mes-*C*H), 103.9-103.8 (m, Aza-C_q), 80.48-80.47 (m, C_5H_4-C_q), 71.0 (s, C_5H_4-*C*H), 70.6 (s, C_5H_4-*C*H), 69.9 (s, Cp-*C*H), 69.7 (s, C_5H_4-*C*H), 68.8 (s, C_5H_4-*C*H), 56.6 (d, $^3J_{PC}$ = 1.7 Hz, *t*Bu-C_q), 49.0 (br s, Aza-*C*H), 29.7 (d, $^4J_{PC}$ = 2.8 Hz, *t*Bu-CH_3), 27.4 (s, Mes-CH_3), 25.6 (dd, $^1J_{PC}$ = 20.5 Hz, $^2J_{RhC}$ = 1.2 Hz, *i*Pr-*C*H), 24.2 (s, Mes-CH_3), 21.3 (s, Mes-CH_3), 20.3 (s, *i*Pr-CH_3), 20.1 (s, *i*Pr-CH_3) ppm.

Comment: The spectrum contains signals corresponding to residual hexane at 31.97, 23.06 and 14.35 ppm and residual benzene at 128.60 ppm resulting from the crystallization process.

^{11}B NMR (160.5 MHz, C_6D_6, 298 K): δ = 20.5 (br s) ppm.

$^{31}P\{^1H\}$ NMR (202.5 MHz, C_6D_6, 298 K): δ = 58.0 (d, $^1J_{RhP}$ = 197 Hz) ppm.

HRMS (LIFDI, $C_{34}H_{51}BClFeNPRh$): *calcd*: m/z = 709.1940, *found*: m/z = 709.1925.

UV-vis (hexane): λ_{abs} = 311, 427 nm.

Synthesis of 1d

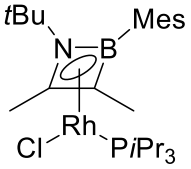

[{(COE)$_2$RhCl}$_2$] (440 mg, 613 μmol) was suspended in pentane (10 mL) and treated with tri*iso*propylphosphine (0.88 mL, 4.61 mmol). After stirring the suspension for 10 min, 2-butyne (0.1 mL, 1.28 mmol) was added. The reaction mixture was stirred for 10 min and all volatiles were removed *in vacuo*. The residue was dissolved in THF (12 mL) and treated with a stock solution of (*tert*-butylimino)mesitylborane in heptane (1.22 mL, 2.46 mmol, 2.02 M). The reaction mixture was stirred for 15 h at room temperature and then all volatiles were removed *in vacuo*. The residue was washed with pentane (4 x 4 mL) and dried under reduced pressure to yield **1d** as a yellow solid (602 mg, 1.09 mmol, 89%). Crystals of **1d** suitable for X-ray diffraction were obtained by evaporation of a saturated hexane solution.

^1H NMR (500.1 MHz, C_6D_6, 298 K): δ= 6.84 (s, 1H, Mes-C*H*), 6.80 (s, 1H, Mes-C*H*), 3.45 (s, 3H, Mes-CH_3), 2.42 (s, 3H, Mes-CH_3), 2.38-2.27 (m, 3H, *i*Pr-C*H*), 2.14 (s, 3H, Mes-CH_3), 1.47 (s, 3H, Aza-CH_3), 1.39 (s, 9H, *t*Bu-CH_3), 1.21-1.17 (m, 12H, *i*Pr-CH_3 overlapping with Aza-CH_3), 1.12 (dd, $^3J_{PH}$ = 12.9 Hz, $^3J_{HH}$ = 7.3 Hz, 9H, *i*Pr-CH_3) ppm.

^{1}H{^{31}P} NMR (500.1 MHz, C_6D_6, 298 K): δ = 6.83 (s, 1H, Mes-C*H*), 6.80 (s, 1H, Mes-C*H*), 3.45 (s, 3H, Mes-CH_3), 2.42 (s, 3H, Mes-CH_3), 2.32 (sept, $^{3}J_{HH}$ = 7.2 Hz, 3H, *i*Pr-C*H*), 2.14 (s, 3H, Mes-CH_3), 1.47 (s, 3H, Aza-CH_3), 1.39 (s, 9H, *t*Bu-CH_3), 1.21 (s, 3H, Aza-CH_3) overlapping with 1.19 (d, $^{3}J_{HH}$ = 7.3 Hz, 9H, *i*Pr-CH_3), 1.12 (d, $^{3}J_{HH}$ = 7.2 Hz, 9H, *i*Pr-CH_3) ppm.

^{13}C{^{1}H} NMR (125.8 MHz, C_6D_6, 298 K): δ = 141.9 (s, Mes-C_q), 140.3 (s, Mes-C_q), 138.1 (s, Mes-C_q), 131.8 (Mes-C_q, detected by HMBC), 128.4 (s, Mes-*C*H), 127.5 (s, Mes-*C*H), 102.7-102.6 (m, Aza-C_q), 64.1 (br s, Aza-C_q), 56.2 (d, $^{3}J_{PC}$ = 1.7 Hz, *t*Bu-C_q), 29.0 (d, $^{4}J_{PC}$ = 2.9 Hz, *t*Bu-CH_3), 27.2 (d, $^{4}J_{RhC}$ = 0.6 Hz, Mes-CH_3), 23.3 (dd, $^{1}J_{PC}$ = 20.2 Hz, $^{2}J_{RhC}$ = 1.2 Hz, *i*Pr-*C*H), 22.9 (s, Mes-CH_3), 21.3 (s, Mes-CH_3), 20.3 (d, $^{2}J_{PC}$ = 1.8 Hz, *i*Pr-CH_3), 19.5 (s, *i*Pr-CH_3), 16.1 (d, $^{2}J_{RhC}$ = 1.2 Hz, Aza-CH_3), 11.5 (s, Aza-CH_3) ppm.

^{13}C{^{1}H, ^{31}P} NMR (75.5 MHz, C_6D_6, 298 K): δ = 141.9 (s, Mes-C_q), 140.3 (s, Mes-C_q), 138.1 (s, Mes-C_q), 128.4 (s, Mes-*C*H), 127.5 (s, Mes-*C*H), 102.7-102.5 (m, Aza-C_q), 64.1 (br s, Aza-C_q), 56.2 (s, *t*Bu-C_q), 29.0 (s, *t*Bu-CH_3), 27.2 (d, $^{4}J_{RhC}$ = 0.7 Hz, Mes-CH_3), 23.3 (d, $^{2}J_{RhC}$ = 1.3 Hz, *i*Pr-*C*H), 22.9 (s, Mes-CH_3), 21.3 (s, Mes-CH_3), 20.3 (d, $^{3}J_{RhC}$ = 0.6 Hz, *i*Pr-CH_3), 19.5 (d, $^{3}J_{RhC}$ = 0.5 Hz, *i*Pr-CH_3), 16.1 (d, $^{2}J_{RhC}$ = 1.3 Hz, Aza-CH_3), 11.5 (s, Aza-CH_3) ppm.

Comment: The broad singlet corresponding to the Mes-C_q nucleus bound to the boron atom could not be observed in the ^{13}C{^{1}H, ^{31}P} NMR spectrum.

^{11}B NMR (160.5 MHz, C_6D_6, 298 K): δ = 20.3 (br s) ppm.

^{31}P{^{1}H} NMR (202.5 MHz, C_6D_6, 298 K): δ = 52.5 (d, $^{1}J_{RhP}$ = 196 Hz) ppm.

HRMS (LIFDI, $C_{26}H_{47}BClNPRh$): *calcd*: m/z = 553.2277, *found*: m/z = 553.2278.

UV-vis (hexane): λ_{abs} = 252, 302, 399 nm.

Synthesis of 1e

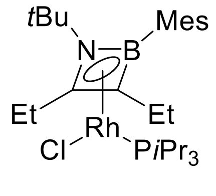

[{$(COE)_2RhCl$}$_2$] (530 mg, 739 µmol) was suspended in pentane (12 mL) and treated with tri*iso*propylphosphine (1.06 mL, 5.55 mmol). After stirring the suspension for 10 min, 3-hexyne (121 mg, 168 µL, 1.48 mmol) was added. The reaction mixture was stirred for 10 min and all volatiles were removed *in vacuo*. The residue was dissolved in THF (10 mL) and treated with

a stock solution of (*tert*-butylimino)mesitylborane in heptane (1.54 mL, 2.96 mmol, 1.93 M). The reaction mixture was stirred for 15 h at room temperature and then all volatiles were removed *in vacuo*. The residue was washed with pentane (4 x 5 mL) and dried under reduced pressure to yield **1e** as an orange solid (526 mg, 904 µmol, 61%). Crystals of **1e** suitable for X-ray diffraction were obtained by evaporation of a saturated hexane solution.

^{1}H NMR (500.1 MHz, C_6D_6, 298 K): δ = 6.84 (s, 1H, Mes-C*H*), 6.81 (s, 1H, Mes-C*H*), 3.50 (s, 3H, Mes-CH_3), 2.45 (s, 3H, Mes-CH_3), 2.41-2.33 (m, 3H, *i*Pr-C*H*), 2.26 (q, $^3J_{HH}$ = 7.6 Hz, 2H, Et-CH_2), 2.16 (s, 3H, Mes-CH_3), 1.66-1.54 (m, 2H, Et-CH_2), 1.46 (s, 9H, *t*Bu-CH_3), 1.25 (dd, $^3J_{PH}$ = 13.7 Hz, $^3J_{HH}$ = 7.2 Hz, 9H, *i*Pr-CH_3), 1.18-1.13 (m, 12H, *i*Pr-CH_3 overlapping with Et-CH_3), 1.03 (t, $^3J_{HH}$ = 7.5 Hz, 3H, Et-CH_3) ppm.
^{13}C{^{1}H} NMR (125.8 MHz, C_6D_6, 298 K): δ = 141.6 (s, Mes-C_q), 139.2 (s, Mes-C_q), 137.9 (s, Mes-C_q), 131.6 (br s, Mes-C_q), 128.4 (s, Mes-*C*H), 127.5 (s, Mes-*C*H), 106.5-106.3 (m, Aza-C_q), 68.2 (br s, Aza-C_q), 56.8 (d, $^3J_{PC}$ = 2.0 Hz, *t*Bu-C_q), 29.2 (d, $^4J_{PC}$ = 2.8 Hz, *t*Bu-*C*H_3), 27.7 (d, $^4J_{RhC}$ = 0.8 Hz, Mes-*C*H_3), 24.4 (d, $^2J_{RhC}$ = 0.7 Hz, Et-*C*H_2), 23.9 (s, Mes-*C*H_3), 23.8 (dd, $^1J_{PC}$ = 19.8 Hz, $^2J_{RhC}$ = 1.3 Hz, *i*Pr-*C*H), 21.3 (s, Mes-*C*H_3), 20.6 (d, $^2J_{PC}$ = 1.5 Hz, *i*Pr-*C*H_3), 20.0 (s, Et-*C*H_2), 19.6 (s, *i*Pr-*C*H_3), 14.8 (s, Et-*C*H_3), 11.2 (d, $^3J_{RhC}$ = 1.4 Hz, Et-*C*H_3) ppm.
^{11}B NMR (160.5 MHz, C_6D_6, 298 K): δ = 21.4 (br s) ppm.
^{31}P{^{1}H} NMR (202.5 MHz, C_6D_6, 298 K): δ = 50.0 (d, $^1J_{RhP}$ = 196 Hz) ppm.
HRMS (LIFDI, $C_{28}H_{51}BClNPRh$): *calcd*: m/z = 581.2590, *found*: m/z = 581.2563.
UV-vis (hexane): λ_{abs} = 253, 298, 395 nm.

Synthesis of 1f

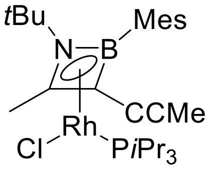

[{(COE)$_2$RhCl}$_2$] (815 mg, 1.14 mmol) was suspended in pentane (15 mL) and treated with tri*iso*propylphosphine (1.63 mL, 8.53 mmol). After stirring the suspension for 15 min, 2,4-hexadiyne (178 mg, 2.28 mmol) was added. The reaction mixture was stirred for 15 min and all volatiles were removed *in vacuo*. The residue was dissolved in THF (15 mL) and treated with a stock solution of (*tert*-butylimino)mesitylborane in heptane (2.68 mL, 6.84 mmol,

2.55 M). After stirring the reaction mixture for 15 h at ambient temperature, all volatiles were removed *in vacuo*. The residue was washed with pentane (5 x 5 mL) and dried under reduced pressure to yield **1f** as an orange solid (936 mg, 1.62 mmol, 71%). Crystals of **1f** suitable for X-ray diffraction were obtained by evaporation of a saturated hexane/benzene (10:1) solution.

^{1}H NMR (500.1 MHz, C_6D_6, 298 K): δ = 6.82 (s, 1H, Mes-C*H*), 6.76 (s, 1H, Mes-C*H*), 3.45 (s, 3H, Mes-CH_3), 2.75-2.67 (m, 3H, *i*Pr-C*H*), 2.63 (s, 3H, Mes-CH_3), 2.11 (s, 3H, Mes-CH_3), 1.64 (s, 3H, Aza-CH_3), 1.44 (s, 3H, CCCH_3) 1.35 (s, 9H, *t*Bu-CH_3), 1.30-1.20 (m, 18H, *i*Pr-CH_3) ppm.

^{1}H{^{31}P} NMR (500.1 MHz, C_6D_6, 298 K): δ = 6.819-6.815 (m, 1H, Mes-C*H*), 6.76-6.75 (m, 1H, Mes-C*H*), 3.44 (s, 3H, Mes-CH_3), 2.70 (sept, $^{3}J_{HH}$ = 7.2 Hz, 3H, *i*Pr-C*H*), 2.63 (s, 3H, Mes-CH_3), 2.11 (s, 3H, Mes-CH_3), 1.637-1.636 (m, 3H, Aza-CH_3), 1.44 (s, 3H, CCCH_3), 1.34 (s, 9H, *t*Bu-CH_3), 1.27 (d, $^{3}J_{HH}$ = 7.2 Hz, 9H, *i*Pr-CH_3), 1.22 (d, $^{3}J_{HH}$ = 7.3 Hz, 9H, *i*Pr-CH_3) ppm.

^{13}C{^{1}H} NMR (125.8 MHz, C_6D_6, 298 K): δ = 141.7 (s, Mes-C_q), 141.2 (s, Mes-C_q), 138.5 (s, Mes-C_q), 129.9 (Mes-C_q, detected by HMBC), 128.4 (s, Mes-*C*H), 127.7 (s, Mes-*C*H), 102.7-102.6 (m, Aza-C_q), 85.48-85.46 (m, *C*CCH$_3$), 78.31-78.30 (m, C*C*CH$_3$), 56.4 (d, $^{3}J_{PC}$ = 1.7 Hz, *t*Bu-C_q), 49.4 (br s, Aza-C_q), 28.8 (d, $^{4}J_{PC}$ = 2.8 Hz, *t*Bu-*C*H$_3$), 27.0 (d, $^{4}J_{RhC}$ = 0.5 Hz, Mes-*C*H$_3$), 23.2 (s, Mes-*C*H$_3$), 22.5 (dd, $^{1}J_{PC}$ = 21.4 Hz, $^{2}J_{RhC}$ = 1.0 Hz, *i*Pr-*C*H), 21.3 (s, Mes-*C*H$_3$), 20.31-20.30 (m, *i*Pr-*C*H$_3$), 19.5 (s, *i*Pr-*C*H$_3$), 17.3 (d, $^{2}J_{RhC}$ = 0.9 Hz, Aza-*C*H$_3$), 4.6 (s, CC*C*H$_3$) ppm.

Comment: The spectrum contains a signal corresponding to residual benzene from the crystallization process at 128.59 ppm.

^{13}C{^{1}H, ^{31}P} NMR (75.5 MHz, C_6D_6, 298 K): δ = 141.7 (s, Mes-C_q), 141.2 (s, Mes-C_q), 138.5 (s, Mes-C_q), 129.8 (br s, Mes-C_q), 102.8-102.6 (m, Aza-C_q), 85.5 (d, $^{2}J_{RhC}$ = 0.6 Hz, *C*CCH$_3$), 78.3 (d, $^{3}J_{RhC}$ = 0.6 Hz, C*C*CH$_3$), 56.4 (s, *t*Bu-C_q), 49.5 (br s, Aza-C_q), 28.8 (s, *t*Bu-*C*H$_3$), 27.0 (d, $^{4}J_{RhC}$ = 0.7 Hz, Mes-*C*H$_3$), 23.2 (s, Mes-*C*H$_3$), 22.5 (d, $^{2}J_{RhC}$ = 1.2 Hz, *i*Pr-*C*H), 21.3 (s, Mes-*C*H$_3$), 20.3 (d, $^{3}J_{RhC}$ = 0.6 Hz, *i*Pr-*C*H$_3$), 19.5 (d, $^{3}J_{RhC}$ = 0.5 Hz *i*Pr-*C*H$_3$), 17.3 (d, $^{2}J_{RhC}$ = 1.2 Hz, Aza-*C*H$_3$), 4.6 (s, CC*C*H$_3$) ppm.

Comment: The broad singlet corresponding to the Mes-C_q nucleus bound to the boron atom could not be observed in the ^{13}C{^{1}H, ^{31}P} NMR spectrum. Due to overlapping with the signal of C_6D_6, the singlet of one Mes-CH nucleus could not be observed in the ^{13}C{^{1}H, ^{31}P} NMR spectrum.

^{11}B NMR (160.5 MHz, C_6D_6, 298 K): δ = 20.3 (br s) ppm.

^{31}P{^{1}H} NMR (202.5 MHz, C_6D_6, 298 K): δ = 50.2 (d, $^{1}J_{RhP}$ = 192 Hz) ppm.

HRMS (LIFDI, $C_{28}H_{47}BClNPRh$): *calcd*: m/z = 577.2277, *found*: m/z = 577.2253.
UV-vis (hexane): λ_{abs} = 267, 306, 387 nm.

Synthesis of 1g

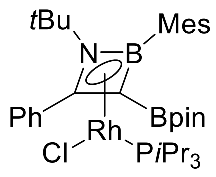

[{(COE)$_2$RhCl}$_2$] (400 mg, 557 µmol) was suspended in pentane (12 mL) and treated with tri*iso*propylphosphine (0.80 mL, 4.19 mmol). After stirring the suspension for 10 min, 4,4,5,5-tetramethyl-2-(2-phenylethynyl)-1,3,2-dioxaborolane (254 mg, 1.11 mmol) was added. The reaction mixture was stirred for 10 min and all volatiles were removed *in vacuo*. The residue was dissolved in THF (12 mL) and treated with a stock solution of (*tert*-butylimino)mesitylborane in heptane (1.16 mL, 2.22 mmol, 1.92 M). After stirring the reaction mixture for 15 h at ambient temperature, all volatiles were removed *in vacuo*. The residue was washed with pentane (7 x 5 mL) and dried under reduced pressure to yield **1g** as an orange solid (490 mg, 673 µmol, 60%). Crystals of **1g** suitable for X-ray diffraction were obtained by evaporation of a saturated hexane solution.

^{1}H NMR (500.1 MHz, C_6D_6, 298 K): δ = 8.29-8.27 (m, 2H, Ph-C*H*), 7.16-7.12 (m, 2H, Ph-C*H*), 7.10-7.07 (m, 1H, Ph-C*H*), 6.86 (s, 1H, Mes-C*H*), 6.81 (s, 1H, Mes-C*H*), 3.51 (s, 3H, Mes-C*H*$_3$), 2.73 (s, 3H, Mes-C*H*$_3$), 2.52-2.44 (m, 3H, *i*Pr-C*H*), 2.12 (s, 3H, Mes-C*H*$_3$), 1.38-1.33 (m, 9H, *i*Pr-C*H*$_3$) overlapping with 1.33 (s, 9H, *t*Bu-C*H*$_3$), 1.11 (dd, $^{3}J_{PH}$ = 13.4 Hz, $^{3}J_{HH}$ = 7.3 Hz, 9H, *i*Pr-C*H*$_3$), 0.96 (s, 6H, Bpin-C*H*$_3$), 0.90 (s, 6H, Bpin-C*H*$_3$) ppm.
^{13}C{^{1}H} NMR (125.8 MHz, C_6D_6, 298 K): δ = 141.3 (s, Mes-C_q), 139.9 (s, Mes-C_q), 138.0 (s, Mes-C_q), 133.9 (d, $^{2}J_{RhC}$ = 1.1 Hz, Ph-C_q), 132.0 (s, Ph-*C*H), 131.9 (br s, Mes-C_q), 129.4 (s, Ph-*C*H), 128.4 (s, Mes-*C*H), 127.6 (s, Mes-*C*H), 127.2 (s, Ph-*C*H), 107.1-107.0 (m, Aza-C_q), 82.3 (s, Bpin-C_q), 57.9 (d, $^{3}J_{PC}$ = 1.3 Hz, *t*Bu-C_q), 43.1 (br s, Aza-C_q), 29.2 (d, $^{4}J_{PC}$ = 2.7 Hz, *t*Bu-*C*H$_3$), 27.2 (d, $^{4}J_{RhC}$ = 0.6 Hz, Mes-*C*H$_3$), 25.7 (d, $^{1}J_{PC}$ = 20.9 Hz, *i*Pr-*C*H), 25.5 (s, Bpin-*C*H$_3$), 25.0 (s, Bpin-*C*H$_3$), 23.7 (s, Mes-*C*H$_3$), 21.3 (s, Mes-*C*H$_3$), 20.4 (s, *i*Pr-*C*H$_3$), 19.5 (s, *i*Pr-*C*H$_3$) ppm.
^{11}B NMR (160.5 MHz, C_6D_6, 298 K): δ = 33.1 (br s, Bpin-B), 24.8 (br s, Aza-B) ppm.

^{31}P{^{1}H} NMR (162.2 MHz, C_6D_6, 298 K): δ = 57.2 (d, $^{1}J_{RhP}$ = 189 Hz) ppm.

HRMS (LIFDI, $C_{36}H_{58}B_2ClNO_2PRh$): *calc*: m/z = 727.3129, *found*: m/z = 727.3090.

UV-vis (hexane): λ_{abs} = 236 (shoulder), 255, 398 nm.

Synthesis of 1h

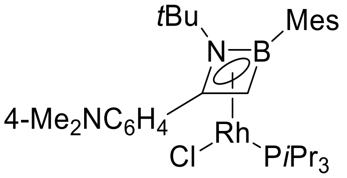

[{(COE)$_2$RhCl}$_2$] (208 mg, 290 μmol) was suspended in pentane (7 mL) and treated with tri*iso*propylphosphine (0.42 mL, 2.20 mmol). After stirring the suspension for 10 min, 4-ethynyl-*N*,*N*-dimethylaniline (84.2 mg, 580 μmol) was added. The reaction mixture was stirred for 10 min and all volatiles were removed *in vacuo*. The residue was dissolved in THF (8 mL) and treated with a stock solution of (*tert*-butylimino)mesitylborane in heptane (0.59 mL, 1.16 mmol, 1.96 M). The reaction mixture was stirred for 15 h at ambient temperature and all volatiles were removed *in vacuo*. The residue was washed with pentane (5 x 4 mL) and dried under reduced pressure to yield **1h** as an orange solid (330 mg, 512 μmol, 88%). Crystals of **1h** suitable for X-ray diffraction were obtained by evaporation of a saturated hexane/benzene (5:1) solution.

^{1}H NMR (500.1 MHz, C_6D_6, 298 K): δ = 7.97-7.94 (m, 2H, 4-$Me_2NC_6H_4$-C*H*), 6.89 (s, 1H, Mes-C*H*), 6.88 (s, 1H, Mes-C*H*), 6.41-6.38 (m, 2H, 4-$Me_2NC_6H_4$-C*H*), 3.47 (s, 3H, Mes-CH_3), 3.19 (s, 1H, Aza-C*H*), 2.78 (s, 3H, Mes-CH_3), 2.41 (s, 6H, N(CH_3)$_2$), 2.25-2.18 (m, 3H, *i*Pr-C*H*) overlapping with 2.18 (s, 3H, Mes-CH_3), 1.39 (s, 9H, *t*Bu-CH_3), 1.15 (dd, $^{3}J_{PH}$ = 13.8 Hz, $^{3}J_{HH}$ = 7.2 Hz, 9H, *i*Pr-CH_3), 0.96 (dd, $^{3}J_{PH}$ = 13.2 Hz, $^{3}J_{HH}$ = 7.2 Hz, 9H, *i*Pr-CH_3) ppm.

^{13}C{^{1}H} NMR (125.8 MHz, C_6D_6, 298 K): δ = 151.0 (s, 4-$Me_2NC_6H_4$-C_q), 141.6 (s, Mes-C_q), 140.5 (s, Mes-C_q), 138.1 (s, Mes-C_q), 132,4 (s, 4-$Me_2NC_6H_4$-*C*H), 132.0 (br s, Mes-C_q), 128.6 (s, Mes-*C*H), 127.7 (s, Mes-*C*H), 121.1 (d, $^{2}J_{RhC}$ = 1.2 Hz, 4-$Me_2NC_6H_4$-C_q), 110.9 (s, 4-$Me_2NC_6H_4$-*C*H), 106.1-106.0 (m, Aza-C_q), 57.0 (d, $^{3}J_{PC}$ = 1.1 Hz, *t*Bu-C_q), 50.7 (br s, Aza-*C*H), 39.6 (s, N(*C*H$_3$)$_2$), 29.6 (d, $^{4}J_{PC}$ = 2.8 Hz, *t*Bu-*C*H$_3$), 26.9 (d, $^{4}J_{RhC}$ = 0.5 Hz, Mes-*C*H$_3$), 25.1 (dd, $^{1}J_{PC}$ = 20.8 Hz, $^{2}J_{RhC}$ = 1.2 Hz, *i*Pr-*C*H), 24.0 (s, Mes-*C*H$_3$), 21.4 (s, Mes-*C*H$_3$), 20.10-20.09 (m, *i*Pr-*C*H$_3$), 19.2 (s, *i*Pr-*C*H$_3$) ppm.

^{11}B NMR (128.5 MHz, C_6D_6, 298 K): δ = 21.4 (br s) ppm.

^{31}P{^{1}H} NMR (202.5 MHz, C_6D_6, 298 K): δ = 59.6 (d, $^{1}J_{RhP}$ = 196 Hz) ppm.

HRMS (LIFDI, $C_{32}H_{52}BClN_2PRh$): *calc*: m/z = 644.2699, *found*: m/z = 644.2689.

UV-vis (hexane): λ_{abs} = 248, 304, 399 nm.

Synthesis of 1i

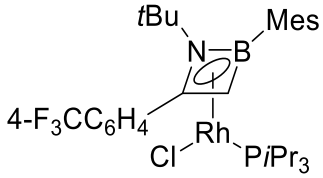

[{(COE)$_2$RhCl}$_2$] (233 mg, 325 μmol) was suspended in pentane (7 mL) and treated with tri*iso*propylphosphine (0.47 mL, 2.46 mmol). After stirring the suspension for 10 min, a solution of 4-ethynyl-α,α,α-trifluorotoluene (111 mg, 106 μL, 650 μmol) in pentane (2 mL) was added. The reaction mixture was stirred for 10 min and all volatiles were removed *in vacuo*. The residue was dissolved in THF (8 mL) and treated with a stock solution of (*tert*-butylimino)mesitylborane in heptane (0.67 mL, 1.31 mmol, 1.96 M). The reaction mixture was stirred for 15 h at room temperature and all volatiles were removed *in vacuo*. The residue was washed with pentane (5 x 4 mL) and dried under reduced pressure to yield **1i** as an orange solid (357 mg, 533 μmol, 82%). Crystals of **1i** suitable for X-ray diffraction were obtained by evaporation of a saturated hexane/benzene (5:1) solution.

^{1}H NMR (500.1 MHz, C_6D_6, 298 K): δ= 7.95 (d, $^{3}J_{HH}$ = 8.1 Hz, 2H, 4-$F_3CC_6H_4$-C*H*), 7.26 (d, $^{3}J_{HH}$ = 8.2 Hz, 2H, 4-$F_3CC_6H_4$-C*H*), 6.89 (s, 1H, Mes-C*H*), 6.85 (s, 1H, Mes-C*H*), 3.37 (s, 3H, Mes-C*H*$_3$), 3.01 (s, 1H, Aza-C*H*), 2.69 (s, 3H, Mes-C*H*$_3$), 2.17 (s, 3H, Mes-C*H*$_3$) overlapping with 2.17-2.08 (m, 3H, *i*Pr-C*H*), 1.22 (s, 9H, *t*Bu-C*H*$_3$), 1.05 (dd, $^{3}J_{PH}$ = 14.0 Hz, $^{3}J_{HH}$ = 7.2 Hz, 9H, *i*Pr-C*H*$_3$), 0.83 (dd, $^{3}J_{PH}$ = 13.4 Hz, $^{3}J_{HH}$ = 7.2 Hz, 9H, *i*Pr-C*H*$_3$)) ppm.

^{13}C{^{1}H} NMR (125.8 MHz, C_6D_6, 298 K): δ = 141.4 (s, Mes-*C*$_q$), 140.4 (s, Mes-*C*$_q$), 138.6 (s, Mes-*C*$_q$), 138.28-138.26 (m, 4-$F_3CC_6H_4$-*C*$_q$), 131,8 (s, 4-$F_3CC_6H_4$-*C*H) overlapping with 131.4 (q, $^{2}J_{CF}$ = 32.5 Hz, 4-$F_3CC_6H_4$-*C*$_q$), 131.1 (Mes-*C*$_q$, detected by HMBC), 128.7 (s, Mes-*C*H), 127.8 (s, Mes-*C*H), 124.9 (q, $^{3}J_{CF}$ = 3.7 Hz, 4-$F_3CC_6H_4$-*C*H), 124.6 (q, $^{1}J_{CF}$ = −272.4 Hz, *C*F$_3$), 102.9-102.8 (m, Aza-*C*$_q$), 57.3 (d, $^{3}J_{PC}$ = 1.2 Hz, *t*Bu-*C*$_q$), 49.1 (br s, Aza-*C*H), 29.5 (d, $^{4}J_{PC}$ = 2.8 Hz, *t*Bu-*C*H$_3$), 26.9 (d, $^{4}J_{RhC}$ = 0.5 Hz, Mes-*C*H$_3$), 25.0 (dd, $^{1}J_{PC}$ = 21.3 Hz, $^{2}J_{RhC}$ = 1.2 Hz,

*i*Pr-*C*H), 23.9 (s, Mes-*C*H_3), 21.3 (s, Mes-*C*H_3), 20.03-20.02 (m, *i*Pr-*C*H_3), 19.0 (s, *i*Pr-*C*H_3) ppm.

Comment: The spectrum contains signals corresponding to residual THF at 67.83 and 25.82 ppm.

^{11}B NMR (160.5 MHz, C_6D_6, 298 K): δ = 21.2 (br s) ppm.

^{19}F NMR (470.6 MHz, C_6D_6, 298 K): δ = −62.5 (s) ppm.

^{31}P{^{1}H} NMR (202.5 MHz, C_6D_6, 298 K): δ = 60.1 (d, $^1J_{RhP}$ = 195 Hz) ppm.

HRMS (LIFDI, $C_{31}H_{46}BClF_3NPRh$): *calcd*: m/z = 669.2151, *found*: m/z = 669.2141.

UV-vis (hexane): λ_{abs} = 231 (shoulder), 302 (shoulder), 408 nm.

Synthesis of 1j

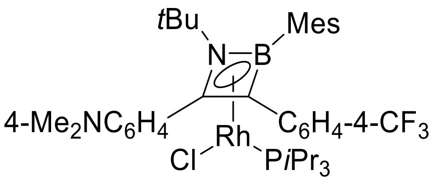

[{(COE)$_2$RhCl}$_2$] (216 mg, 301 µmol) was suspended in pentane (10 mL) and treated with tri*iso*propylphosphine (0.43 mL, 2.25 mmol). After stirring the suspension for 10 min, *N*,*N*-dimethyl-4-{2-[4-trifluoromethyl)phenyl]ethynyl}benzenamine (174 mg, 602 µmol) was added. The reaction mixture was stirred for 10 min and all volatiles were removed *in vacuo*. The residue was dissolved in THF (10 mL) and treated with a stock solution of (*tert*-butylimino)mesitylborane in heptane (0.77 mL, 1.51 mmol, 1.96 M). After stirring the reaction mixture for 15 h at room temperature, all volatiles were removed *in vacuo*. The residue was washed with pentane (5 x 4 mL) and dried under reduced pressure to yield **1j** as an orange solid (380 mg, 482 µmol, 80%). Crystals of **1j** suitable for X-ray diffraction were obtained by evaporation of a saturated hexane/benzene (2:1) solution.

^{1}H NMR (500.1 MHz, d_8-THF, 298 K): δ= 8.30 (br s, 1H, 4-$Me_2NC_6H_4$-C*H*), 7.55 (br s, 1H, 4-$Me_2NC_6H_4$-C*H*), 7.24 (br s, 2H, 4-$F_3CC_6H_4$-C*H*), 7.15 (br s, 1H, 4-$F_3CC_6H_4$-C*H*), 6.97 (br s, 1H, 4-$F_3CC_6H_4$-C*H*), 6.88-6.83 (m, 4H, Mes-C*H* overlapping with 4-$Me_2NC_6H_4$-C*H*), 3.21 (s, 3H, Mes-C*H*$_3$), 3.05 (s, 6H, N(C*H*$_3$)$_2$), 2.35 (s, 3H, Mes-C*H*$_3$), 2.27 (s, 3H, Mes-C*H*$_3$), 1.90-1.83 (m, 3H, *i*Pr-C*H*), 1.19 (s, 9H, *t*Bu-C*H*$_3$), 1.15 (dd, $^3J_{PH}$ = 13.7 Hz, $^3J_{HH}$ = 7.2 Hz, 9H, *i*Pr-C*H*$_3$), 1.04 (dd, $^3J_{PH}$ = 13.1 Hz, $^3J_{HH}$ = 7.3 Hz, 9H, *i*Pr-C*H*$_3$) ppm.

^{13}C{^{1}H} NMR (125.8 MHz, d_8-THF, 298 K): δ = 152.2 (s, 4-$Me_2NC_6H_4$-C_q), 145.37-145.34 (m, 4-$F_3CC_6H_4$-C_q), 141.8 (s, Mes-C_q), 140.7 (s, Mes-C_q), 139.0 (s, Mes-C_q), 134,4 (br s, 4-$Me_2NC_6H_4$-*C*H), 131.5 (br s, Mes-C_q), 130.8 (br s, 4-$F_3CC_6H_4$-*C*H overlapping with 4-$Me_2NC_6H_4$-*C*H), 128.8 (s, Mes-*C*H), 128.3 (s, Mes-*C*H), 127.1 (q, $^{2}J_{CF}$ = 32.0 Hz, 4-$F_3CC_6H_4$-C_q), 127.0 (br s, 4-$F_3CC_6H_4$-*C*H), 125.7 (q, $^{1}J_{CF}$ = −271.3 Hz, CF_3), 125.6 (br s, 4-$F_3CC_6H_4$-*C*H), 125.2 (br s, 4-$F_3CC_6H_4$-*C*H), 119.1 (d, $^{2}J_{RhC}$ = 0.7 Hz, 4-$Me_2NC_6H_4$-C_q), 111.7 (s, 4-$Me_2NC_6H_4$-*C*H), 101.6-101.5 (m, Aza-C_q), 63.8 (br s, Aza-C_q), 58.0 (d, $^{3}J_{PC}$ = 1.6 Hz, *t*Bu-C_q), 40.1 (s, N(CH_3)$_2$), 29.3 (d, $^{4}J_{PC}$ = 2.8 Hz, *t*Bu-CH_3), 27.3 (s, Mes-CH_3), 23.8 (dd, $^{1}J_{PC}$ = 20.9 Hz, $^{2}J_{RhC}$ = 0.8 Hz, *i*Pr-*C*H), 22.9 (s, Mes-CH_3), 21.4 (s, Mes-CH_3), 20.2 (s, *i*Pr-CH_3), 19.6 (s, *i*Pr-CH_3) ppm.

^{11}B NMR (128.5 MHz, d_8-THF, 298 K): δ = 22.4 (br s) ppm.

^{19}F NMR (470.6 MHz, d_8-THF, 298 K): δ = −63.5 (s) ppm.

^{31}P{^{1}H} NMR (162.2 MHz, d_8-THF, 298 K): δ = 48.4 (d, $^{1}J_{RhP}$ = 189 Hz) ppm.

^{1}H NMR (500.1 MHz, d_8-THF, 233 K): δ = 8.27-8.25 (m, 1H, 4-$Me_2NC_6H_4$-C*H*), 7.55-7.53 (m, 1H, 4-$Me_2NC_6H_4$-C*H*), 7.31 (d, $^{3}J_{HH}$ = 8.2 Hz, 1H, 4-$F_3CC_6H_4$-C*H*), 7.28 (d, $^{3}J_{HH}$ = 8.4 Hz, 1H, 4-$F_3CC_6H_4$-C*H*), 7.12 (d, $^{3}J_{HH}$ = 8.2 Hz, 1H, 4-$F_3CC_6H_4$-C*H*), 6.96 (d, $^{3}J_{HH}$ = 8.2 Hz, 1H, 4-$F_3CC_6H_4$-C*H*), 6.90-6.89 (m, 2H, Mes-C*H*, 4-$Me_2NC_6H_4$-C*H*), 6.84 (s, 1H, Mes-C*H*), 6.79-6.77 (m, 1H, 4-$Me_2NC_6H_4$-C*H*), 3.19 (s, 3H, Mes-CH_3), 3.07 (s, 6H, N(CH_3)$_2$), 2.34 (s, 3H, Mes-CH_3), 2.28 (s, 3H, Mes-CH_3), 1.17 (s, 9H, *t*Bu-CH_3) overlapping with 1.04 (br s, 18H, *i*Pr-CH_3) ppm.

Comment: The signal for the iPr-CH nucleus could not be observed due to overlapping with several signals.

^{13}C{^{1}H} NMR (125.8 MHz, d_8-THF, 233 K): δ = 151.8 (s, 4-$Me_2NC_6H_4$-C_q), 145.29-145.25 (m, 4-$F_3CC_6H_4$-C_q), 141.6 (s, Mes-C_q), 140.8 (s, Mes-C_q), 138.9 (s, Mes-C_q), 134,1-134.0 (m, 4-$Me_2NC_6H_4$-*C*H), 131.3 (br s, Mes-C_q), 130.6 (s, 4-$F_3CC_6H_4$-*C*H), 130,5 (s, 4-$Me_2NC_6H_4$-*C*H), 128.8 (s, Mes-*C*H), 128.3 (s, Mes-*C*H), 126.9 (s, 4-$F_3CC_6H_4$-*C*H), 126.7 (q, $^{2}J_{CF}$ = 31.9 Hz, 4-$F_3CC_6H_4$-C_q), 125.77-125.72 (m, 4-$F_3CC_6H_4$-*C*H), 125.7 (q, $^{1}J_{CF}$ = −271.3 Hz, CF_3), 125.29-125.24 (m, 4-$F_3CC_6H_4$-*C*H), 118.6 (s, 4-$Me_2NC_6H_4$-C_q), 111.6 (s, 4-$Me_2NC_6H_4$-*C*H), 111.4 (s, 4-$Me_2NC_6H_4$-*C*H), 101.6-101.5 (m, Aza-C_q), 63.74-63.66 (m, Aza-C_q), 57.8 (d, $^{3}J_{PC}$ = 1.0 Hz, *t*Bu-C_q), 40.1 (s, N(CH_3)$_2$), 29.02-29.01 (m, *t*Bu-CH_3), 27.2 (s, Mes-CH_3), 23.6 (br s, *i*Pr-*C*H), 23.0 (s, Mes-CH_3), 21.5 (s, Mes-CH_3), 19.4 (br s, *i*Pr-CH_3) ppm.

HRMS (LIFDI, $C_{39}H_{55}BClF_3N_2PRh$): *calcd*: m/z = 788.2886, *found*: m/z = 788.2868.

UV-vis (hexane): λ_{abs} = 269, 312, 395 nm.

Synthesis of I

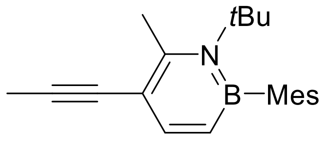

1f (936 mg, 1.62 mmol) was dissolved in benzene (12 mL) and the argon atmosphere was replaced by acetylene. After stirring the reaction mixture for 1.5 h at 86 °C, all volatiles were removed *in vacuo*. The residue was purified by column chromatography on silica gel with a mixture of dichloromethane and hexane (1:10) as eluent. Evaporation of the solvent from the second fraction yielded pure **I** as a white solid (360 mg, 1.18 mmol, 73%). Crystals of **I** suitable for X-ray diffraction were obtained by evaporation of a saturated hexane/dichloromethane (10:1) solution (crystal data **Ia**) or by evaporation of a saturated pentane/ether (10:1) solution (crystal data **Ib**).

^{1}H NMR (500.1 MHz, C_6D_6, 298 K): δ= 7.76 (d, $^{3}J_{HH}$ = 10.9 Hz, 1H, Aza1-C*H*), 6.84 (m, 2H, Mes-C*H*), 6.54 (d, $^{3}J_{HH}$ = 10.9 Hz, 1H, Aza1-C*H*), 2.78 (s, 3H, Aza1-CH_3), 2.25 (s, 3H, Mes-CH_3), 2.21 (s, 6H, Mes-CH_3), 1.81 (s, 3H, CCCH_3), 1.30 (s, 9H, *t*Bu-CH_3) ppm.
^{13}C{^{1}H} NMR (125.8 MHz, C_6D_6, 298 K): δ = 150.2 (s, Aza1-C_q), 147.3 (br s, Mes-C_q), 145.6 (s, Aza1-*C*H), 136.7 (s, Mes-C_q), 135.6 (s, Mes-C_q), 131.9 (br s, Aza1-*C*H), 127.8 (s, Mes-*C*H), 111.9 (s, Aza1-C_q), 88.3 (s, C*C*CH$_3$), 81.1 (s, *C*CCH$_3$), 61.7 (s, *t*Bu-C_q), 34.5 (s, *t*Bu-*C*H$_3$), 25.1 (s, Aza1-*C*H$_3$), 23.9 (s, Mes-*C*H$_3$), 21.3 (s, Mes-*C*H$_3$), 4.4 (s, CC*C*H$_3$) ppm.
^{11}B NMR (160.5 MHz, C_6D_6, 298 K): δ = 39.0 (br s) ppm.
Elemental analysis: % *calc*: C 82.63, H 9.25, N 4.59; *found*: C 82.40, H 9.36, N 4.68.
HRMS (ASAP, $C_{21}H_{28}BN$ + H): *calcd*: m/z = 306.2388, *found*: m/z = 306.2377.

Synthesis of 1k

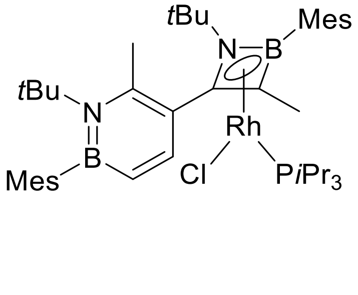

[{(COE)$_2$RhCl}$_2$] (70.0 mg, 97.6 µmol) was suspended in pentane (7 mL) and treated with tri*iso*propylphosphine (0.14 mL, 73.3 µmol). After stirring the suspension for 10 min, 1-(*tert*-butyl)-2-mesityl-6-methyl-5-(prop-1-yn-1-yl)-1,2-azaborinine (59.5 mg, 195 µmol) was added. The reaction mixture was stirred for 10 min and all volatiles were removed *in vacuo*. The residue was dissolved in THF (8 mL) and treated with a stock solution of (*tert*-butylimino)mesitylborane in heptane (0.20 mL, 386 µmol, 1.93 M). The reaction mixture was stirred for 15 h at room temperature and all volatiles were removed *in vacuo*. The residue was washed with pentane (4 x 3 mL) and dried under reduced pressure to yield **1k** as an orange solid (128 mg, 159 µmol, 82%). Crystals of **1k** suitable for X-ray diffraction were obtained by evaporation of a saturated hexane/benzene (5:1) solution.

^{1}H NMR (500.1 MHz, C_6D_6, 298 K): δ = 9.34 (d, $^3J_{HH}$ = 11.1 Hz, 1H, Aza1-C*H*), 6.91 (s, 1H, Mes-C*H*), 6.88 (s, 1H, Mes-C*H*), 6.867-6.866 (m, 2H, Mes-C*H*), 6.61 (d, $^3J_{HH}$ = 11.1 Hz, 1H, Aza1-C*H*), 3.57 (s, 3H, Mes-CH_3), 2.82 (s, 3H, Aza1-CH_3), 2.67 (s, 3H, Mes-CH_3), 2.43-2.36 (m, 3H, *i*Pr-C*H*), 2.27 (s, 3H, Mes-CH_3), 2.24 (s, 3H, Mes-CH_3), 2.20 (s, 3H, Mes-CH_3), 2.18 (s, 3H, Mes-CH_3), 1.43 (s, 9H, *t*Bu-CH_3), 1.38-1.37 (m, 3H, Aza-CH_3) overlapping with 1.37 (s, 9H, *t*Bu-CH_3), 1.20 (dd, $^3J_{PH}$ = 13.5 Hz, $^3J_{HH}$ = 7.2 Hz, 9H, *i*Pr-CH_3), 1.05 (dd, $^3J_{PH}$ = 13.0 Hz, $^3J_{HH}$ = 7.3 Hz, 9H, *i*Pr-CH_3) ppm.

^{13}C{^{1}H} NMR (125.8 MHz, C_6D_6, 298 K): δ = 148.2 (s, Aza1-*C*H), 146.8 (br s, Mes-C$_q$), 146.1 (s, Aza1-C$_q$), 142.3 (s, Mes-C_q), 140.6 (s, Mes-C_q), 138.2 (s, Mes-C_q), 136.8 (s, Mes-C_q), 136.6 (s, Mes-C_q), 135.9 (s, Mes-C_q), 131.4 (br s, Mes-C_q), 130.5 (br s, Aza1-*C*H), 128.8 (s, Mes-*C*H), 128.0 (s, Mes-*C*H), 127.9 (s, Mes-*C*H), 127.8 (s, Mes-*C*H), 118.0 (d, $^2J_{RhC}$ = 0.7 Hz, Aza1-C$_q$), 105.8-105.6 (m, Aza-C_q), 68.0 (br s, Aza-C_q), 61.7 (s, *t*Bu-C_q), 57.0 (d, $^3J_{PC}$ = 1.5 Hz, *t*Bu-C_q), 34.6 (s, *t*Bu-*C*H$_3$), 29.6 (d, $^4J_{PC}$ = 2.8 Hz, *t*Bu-*C*H$_3$), 27.7 (s, Mes-*C*H$_3$), 24.2 (s, Aza1-*C*H$_3$), 23.9 (s, Mes-*C*H$_3$), 23.6 (s, Mes-*C*H$_3$), 23.3 (s, Mes-*C*H$_3$) overlapping with 23.3-23.2 (m, *i*Pr-*C*H), 21.3 (s, Mes-*C*H$_3$), 21.3 (s, Mes-*C*H$_3$), 19.871-19.866 (m, *i*Pr-*C*H$_3$), 19.3 (s, *i*Pr-*C*H$_3$), 13.8 (s, Aza-*C*H$_3$) ppm.

^{11}B NMR (128.5 MHz, C_6D_6, 298 K): δ = 39.2 (br s, Aza1-B), 20.3 (br s, Aza-B) ppm.

^{31}P{^{1}H} NMR (202.5 MHz, C_6D_6, 298 K): δ = 49.7 (d, $^1J_{RhP}$ = 194 Hz) ppm.

HRMS (LIFDI, $C_{43}H_{69}B_2ClN_2PRh$): *calcd*: m/z = 804.4123, *found*: m/z = 804.4110.

UV-vis (hexane): λ_{abs} = 254, 299 (shoulder), 413 nm.

Synthesis of 1l

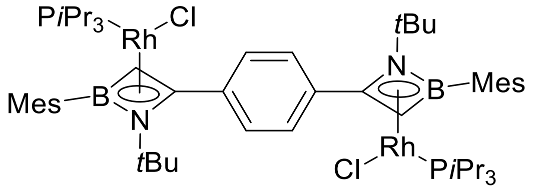

[{(COE)$_2$RhCl}$_2$] (500 mg, 697 µmol) was suspended in pentane (12 mL) and treated with tri*iso*propylphosphine (1.00 mL, 5.24 mmol). After stirring the suspension for 15 min, 1,4-diethynylbenzene (87.9 mg, 697 µmol) was added. The reaction mixture was stirred for 15 min and all volatiles were removed *in vacuo*. The residue was suspended in THF (20 mL) and treated with a stock solution of (*tert*-butylimino)mesitylborane in heptane (1.43 mL, 2.79 mmol, 1.96 M). The reaction mixture was stirred for 15 h at ambient temperature and then all volatiles were removed *in vacuo*. After washing the residue with pentane (5 x 7 mL) and benzene (2 x 3 mL), residual solvent was removed under reduced pressure to yield **1l** as an orange solid (243 mg, 216 µmol, 31%). Crystals of **1l** suitable for X-ray diffraction were obtained by slow evaporation of a saturated benzene solution.

^{1}**H NMR** (500.1 MHz, C_6D_6, 298 K): δ = 7.95 (s, 4H, C_6H_4-C*H*), 6.91 (s, 2H, Mes-C*H*), 6.88 (s, 2H, Mes-C*H*), 3.42 (s, 6H, Mes-CH_3), 3.09 (s, 2H, Aza-C*H*), 2.75 (s, 6H, Mes-CH_3), 2.24-2.16 (m, 6H, *i*Pr-C*H*) overlapping with 2.19 (s, 6H, Mes-CH_3) 1.31 (s, 18H, *t*Bu-CH_3), 1.12 (dd, $^{3}J_{PH}$ = 14.0 Hz, $^{3}J_{HH}$ = 7.2 Hz, 18H, *i*Pr-CH_3), 0.91 (dd, $^{3}J_{PH}$ = 13.4 Hz, $^{3}J_{HH}$ = 7.3 Hz, 18H, *i*Pr-CH_3) ppm.

^{13}**C{^{1}H} NMR** (125.8 MHz, C_6D_6, 298 K): δ = 141.5 (s, Mes-C_q), 140.5 (s, Mes-C_q), 138.6 (s, Mes-C_q), 135.6 (d, $^{2}J_{RhC}$ = 0.9 Hz, C_6H_4-C_q), 131.4 (Mes-C_q, detected by HMBC), 130.8 (s, C_6H_4-*C*H), 128.7 (s, Mes-*C*H), 127.8 (Mes-*C*H, detected by HSQC), 103.8-103.7 (m, Aza-C_q), 57.3 (d, $^{3}J_{PC}$ = 1.2 Hz, *t*Bu-C_q), 49.4 (br s, Aza-*C*H), 29.6 (d, $^{4}J_{PC}$ = 2.7 Hz, *t*Bu-*C*H_3), 26.9 (s, Mes-*C*H_3), 25.1 (d, $^{1}J_{PC}$ = 21.1 Hz, *i*Pr-*C*H), 24.0 (s, Mes-*C*H_3), 21.3 (s, Mes-*C*H_3), 20.1 (d, $^{2}J_{PC}$ = 0.9 Hz, *i*Pr-*C*H_3), 19.2 (s, *i*Pr-*C*H_3) ppm.

^{11}**B NMR** (128.4 MHz, C_6D_6, 298 K): δ = 20.5 (br s, 2 B) ppm.

^{31}**P{^{1}H} NMR** (162.0 MHz, C_6D_6, 298 K): δ = 60.3 (d, $^{1}J_{RhP}$ = 196 Hz, 2 P) ppm.

HRMS (LIFDI, $C_{54}H_{88}B_2Cl_2N_2P_2Rh_2$): *calcd*: m/z = 1124.4090, *found*: m/z = 1124.4094.

UV-vis (THF): λ_{abs} = 242, 304 (shoulder), 409 nm.

Synthesis of 2a

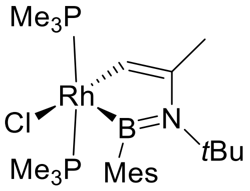

1a (280 mg, 519 µmol) was dissolved in benzene (12 mL) and treated with a stock solution of trimethylphosphine in benzene (1.52 mL, 1.14 mmol, 0.75 M). After stirring the reaction mixture for 30 mins, all volatiles were removed *in vacuo*. The residue was washed with hexane (2 mL) at −30 °C and dried under reduced pressure to yield **2a** as a yellow solid (194 mg, 365 µmol, 70%). Crystals of **2a** suitable for X-ray diffraction were obtained by slow evaporation of a saturated hexane solution at −30 °C.

^{1}H NMR (500.1 MHz, C_6D_6, 298 K): δ = 6.80 (s, 2H, Mes-C*H*), 5.79 (t, $^{3}J_{PH}$ = 5.0 Hz, 1H, RhC*H*), 2.47 (s, 6H, Mes-CH_3), 2.27-2.26 (m, 3H, RhCCCH_3), 2.18 (s, 3H, Mes-CH_3), 1.20 (s, 9H, *t*Bu-CH_3), 1.12 (vtd, *N* = 6.8 Hz, $^{3}J_{RhH}$ = 0.8 Hz, 18H, P(CH_3)$_3$) ppm.
^{13}C{^{1}H} NMR (125.8 MHz, C_6D_6, 298 K): δ = 146.6 (t, $^{3}J_{PC}$ = 4.5 Hz, RhC*C*CH$_3$), 142.8 (br s, Mes-C_q), 137.7 (t, $^{4}J_{PC}$ = 0.8 Hz, Mes-C_q), 136.6 (s, Mes-C_q), 128.3 (Mes-*C*H, detected by HSQC), 125.1 (dt, $^{1}J_{RhC}$ = 30.9 Hz, $^{2}J_{PC}$ = 13.0 Hz, Rh*C*H), 55.4 (d, $^{3}J_{RhC}$ = 1.2 Hz, *t*Bu-C_q), 32.6 (s, *t*Bu-*C*H$_3$), 26.2 (t, $^{5}J_{PC}$ = 1.4 Hz, Mes-*C*H$_3$), 24.5-24.4 (m, RhCC*C*H$_3$), 21.4 (s, Mes-*C*H$_3$), 14.1 (vtd, *N* = 28.9 Hz, $^{2}J_{RhC}$ = 1.3 Hz, P(*C*H$_3$)$_3$) ppm.
^{11}B NMR (160.5 MHz, C_6D_6, 298 K): δ = 67.8 (br s) ppm.
^{31}P{^{1}H} NMR (202.5 MHz, C_6D_6, 298 K): δ = −4.7 (d, $^{1}J_{RhP}$ = 130 Hz, 2 P) ppm.
HRMS (LIFDI, $C_{22}H_{42}BClNP_2Rh$): *calcd*: m/z = 531.1624, *found*: m/z = 531.1612.
UV-vis (hexane): λ_{abs} = 250 (shoulder), 290, 355 nm.

Synthesis of 2b

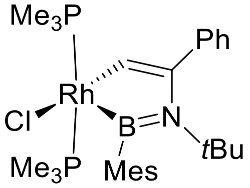

1b (280 mg, 465 μmol) was dissolved in benzene (8 mL) and treated with a stock solution of trimethylphosphine in benzene (1.36 mL, 1.02 mmol, 0.75 M). After stirring the reaction mixture for 1 h, all volatiles were removed *in vacuo*. The residue was washed with hexane (2 mL) at −30 °C and dried under reduced pressure to yield **2b** as a yellow solid (197 mg, 332 μmol, 71%). Crystals of **2b** suitable for X-ray diffraction were obtained by slow evaporation of a saturated hexane solution at −30 °C.

^{1}H NMR (500.1 MHz, C_6D_6, 298 K): δ = 7.42-7.40 (m, 2H, Ph-C*H*), 7.22-7.19 (m, 2H, Ph-C*H*), 7.13-7.10 (m, 1H, Ph-C*H*), 6.85 (s, 2H, Mes-C*H*), 5.80 (t, $^{3}J_{PH}$ = 5.1 Hz, 1H, RhC*H*), 2.63 (s, 6H, Mes-CH_3), 2.19 (s, 3H, Mes-CH_3), 1.16 (s, 9H, *t*Bu-CH_3), 1.14 (vtd, *N* = 6.9 Hz, $^{3}J_{RhH}$ = 0.9 Hz, 18H, P(CH_3)$_3$) ppm.

^{13}C{^{1}H} NMR (125.8 MHz, C_6D_6, 298 K): δ = 153.8 (t, $^{3}J_{PC}$ = 4.7 Hz, N*C*Ph), 144.92-144.88 (m, Ph-C_q), 142.3 (br s, Mes-C_q), 138.1 (t, $^{4}J_{PC}$ = 0.7 Hz, Mes-C_q), 137.1 (s, Mes-C_q), 131.7 (dt, $^{1}J_{RhC}$ = 31.4 Hz, $^{2}J_{PC}$ = 12.8 Hz, Rh*C*H), 128.9 (t, $^{5}J_{PC}$ = 1.8 Hz, Ph-*C*H), 128.5 (s, Mes-*C*H), 127.9 (s, Ph-*C*H), 126.1 (s, Ph-*C*H), 56.3 (d, $^{3}J_{RhC}$ = 1.1 Hz, *t*Bu-C_q), 32.6 (s, *t*Bu-CH_3), 26.6 (t, $^{5}J_{PC}$ = 1.3 Hz, Mes-CH_3), 21.4 (s, Mes-CH_3), 14.1 (vtd, *N* = 29.1 Hz, $^{2}J_{RhC}$ = 1.3 Hz, P(CH_3)$_3$) ppm.

^{11}B NMR (160.5 MHz, C_6D_6, 298 K): δ = 70.2 (br s) ppm.

^{31}P{^{1}H} NMR (202.5 MHz, C_6D_6, 298 K): δ = −4.5 (d, $^{1}J_{RhP}$ = 128 Hz, 2 P) ppm.

HRMS (LIFDI, $C_{27}H_{44}BClNP_2Rh$): *calcd*: m/z = 593.1780, *found*: m/z = 593.1770.

UV-vis (hexane): λ_{abs} = 252, 294, 348 nm.

Synthesis of 2c

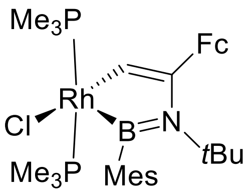

1c (152 mg, 214 μmol) was dissolved in benzene (6 mL) and treated with a stock solution of trimethylphosphine in benzene (0.53 mL, 470 μmol, 0.887 M). After stirring the reaction mixture for 1 h, all volatiles were removed *in vacuo*. The residue was washed with hexane (2 x 2 mL) at −30 °C and dried under reduced pressure to yield **2c** as an orange solid (102 mg,

145 μmol, 68%). Crystals of **2c** suitable for X-ray diffraction were obtained by evaporation of a saturated benzene solution.

^{1}H NMR (500.1 MHz, C_6D_6, 298 K): δ = 6.97 (t, $^3J_{PH}$ = 5.3 Hz, 1H, RhC*H*), 6.83 (s, 2H, Mes-C*H*), 4.24-4.23 (m, 2H, C_5H_4-C*H*), 4.17 (s, 5H, Cp-C*H*), 4.01-4.00 (m, 2H, C_5H_4-C*H*), 2.54 (s, 6H, Mes-CH_3), 2.19 (s, 3H, Mes-CH_3), 1.26 (vtd, *N* = 6.8 Hz, $^3J_{RhH}$ = 0.9 Hz, 18H, P(CH_3)$_3$) 1.06 (s, 9H, *t*Bu-CH_3) ppm.
Comment: The spectrum contains a signal corresponding to residual benzene at 7.16 (s) ppm.
^{13}C{^{1}H} NMR (125.8 MHz, C_6D_6, 298 K): δ = 146.4 (t, $^3J_{PC}$ = 4.4 Hz, N*C*Fc), 142.7 (br s, Mes-C_q), 137.9 (t, $^4J_{PC}$ = 0.8 Hz, Mes-C_q), 136.7 (s, Mes-C_q), 132.9 (dt, $^1J_{RhC}$ = 30.6 Hz, $^2J_{PC}$ = 12.6 Hz, Rh*C*H), 128.4 (s, Mes-*C*H), 95.14-95.09 (m, C_5H_4-C_q), 73.4 (t, $^5J_{PC}$ = 1.7 Hz, C_5H_4-*C*H), 69.4 (s, Cp-*C*H), 66.3 (s, C_5H_4-*C*H), 56.1 (d, $^3J_{RhC}$ = 1.2 Hz, *t*Bu-C_q), 32.8 (s, *t*Bu-*C*H_3), 26.4 (t, $^5J_{PC}$ = 1.2 Hz, Mes-*C*H_3), 21.4 (s, Mes-*C*H_3), 14.4 (vtd, *N* = 28.9 Hz, $^2J_{RhC}$ = 1.3 Hz, P(*C*H_3)$_3$) ppm.
Comment: The spectrum contains a signal corresponding to residual benzene at 128.6 ppm.
^{11}B NMR (160.5 MHz, C_6D_6, 298 K): δ = 68.7 (br s) ppm.
^{31}P{^{1}H} NMR (202.5 MHz, C_6D_6, 298 K): δ = −5.5 (d, $^1J_{RhP}$ = 130 Hz, 2 P) ppm.
HRMS (LIFDI, $C_{31}H_{48}BFeClNP_2Rh$): *calcd*: m/z = 701.1443, *found*: m/z = 701.1432.
UV-vis (THF): λ_{abs} = 255 (shoulder), 349 nm.

Synthesis of 2d

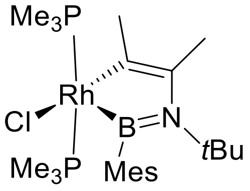

1d (500 mg, 903 μmol) was dissolved in benzene (10 mL) and treated with a stock solution of trimethylphosphine in benzene (2.65 mL, 1.99 mmol, 0.75 M). After stirring the reaction mixture for 1 h, all volatiles were removed *in vacuo*. The residue was washed with hexane (2 mL) at −30 °C and dried under reduced pressure to yield **2d** as a yellow solid (431 mg, 790 μmol, 87%). Crystals of **2d** suitable for X-ray diffraction were obtained by slow evaporation of a saturated hexane solution at −30 °C.

^{1}H NMR (500.1 MHz, C_6D_6, 298 K): δ = 6.806-6.805 (m, 2H, Mes-C*H*), 2.50 (s, 6H, Mes-C*H*$_3$), 2.19 (s, 3H, Mes-C*H*$_3$), 2.10-2.09 (m, 3H, RhCCC*H*$_3$), 2.08-2.07 (m, 3H, RhCC*H*$_3$), 1.22 (s, 9H, *t*Bu-C*H*$_3$), 1.10 (vtd, *N* = 6.8 Hz, $^{3}J_{RhH}$ = 1.0 Hz, 18H, P(C*H*$_3$)$_3$) ppm.

^{1}H{^{31}P} NMR (500.1 MHz, C_6D_6, 298 K): δ = 6.80 (s, 2H, Mes-C*H*), 2.50 (s, 6H, Mes-C*H*$_3$), 2.19 (s, 3H, Mes-C*H*$_3$), 2.10 (s, 3H, RhCCC*H*$_3$), 2.075-2.072 (m, 3H, RhCC*H*$_3$), 1.22 (s, 9H, *t*Bu-C*H*$_3$), 1.10 (d, $^{3}J_{RhH}$ = 0.7 Hz, 18H, P(C*H*$_3$)$_3$) ppm.

^{13}C{^{1}H} NMR (125.8 MHz, C_6D_6, 298 K): δ = 143.4 (Mes-*C*$_q$, detected by HMBC), 139.7 (dt, $^{2}J_{RhC}$ = 0.5 Hz, $^{3}J_{PC}$ = 4.5 Hz, RhC*C*CH$_3$), 137.9 (t, $^{4}J_{PC}$ = 0.7 Hz, Mes-*C*$_q$), 136.6 (s, Mes-*C*$_q$), 129.8 (dt, $^{1}J_{RhC}$ = 30.7 Hz, $^{2}J_{PC}$ = 11.3 Hz, Rh*C*CH$_3$), 128.3 (s, Mes-*C*H), 55.2 (d, $^{3}J_{RhC}$ = 1.2 Hz, *t*Bu-*C*$_q$), 33.0 (s, *t*Bu-*C*H$_3$), 26.7 (t, $^{5}J_{PC}$ = 0.9 Hz, Mes-*C*H$_3$), 23.8 (dt, $^{2}J_{RhC}$ = 0.5 Hz, $^{3}J_{PC}$ = 3.3 Hz, RhC*C*H$_3$), 21.4 (s, Mes-*C*H$_3$), 17.54-17.50 (m, RhCC*C*H$_3$), 14.7 (vtd, *N* = 28.2 Hz, $^{2}J_{RhC}$ = 1.4 Hz, P(*C*H$_3$)$_3$) ppm.

^{13}C{^{1}H, ^{31}P} NMR (75.5 MHz, C_6D_6, 298 K): δ = 143.3 (br s, Mes-*C*$_q$), 139.7 (d, $^{2}J_{RhC}$ = 0.5 Hz, RhC*C*CH$_3$), 137.9 (s, Mes-*C*$_q$), 136.5 (s, Mes-*C*$_q$), 129.8 (d, $^{1}J_{RhC}$ = 30.7 Hz, Rh*C*CH$_3$), 128.3 (s, Mes-*C*H), 55.2 (d, $^{3}J_{RhC}$ = 1.2 Hz, *t*Bu-*C*$_q$), 33.0 (s, *t*Bu-*C*H$_3$), 26.7 (s, Mes-*C*H$_3$), 23.8 (d, $^{2}J_{RhC}$ = 0.5 Hz, RhC*C*H$_3$), 21.4 (s, Mes-*C*H$_3$), 17.5 (d, $^{3}J_{RhC}$ = 2.0 Hz, RhCC*C*H$_3$), 14.7 (d, $^{2}J_{RhC}$ = 1.4 Hz, P(*C*H$_3$)$_3$) ppm.

^{11}B NMR (160.5 MHz, C_6D_6, 298 K): δ = 68.0 (br s) ppm.

^{31}P{^{1}H} NMR (202.5 MHz, C_6D_6, 298 K): δ = −5.1 (d, $^{1}J_{RhP}$ = 133 Hz, 2 P) ppm.

HRMS (LIFDI, $C_{23}H_{44}BClNP_2Rh$): *calcd*: m/z = 545.1780, *found*: m/z = 545.1757.

UV-vis (hexane): λ_{abs} = 254, 291 (shoulder), 352 nm.

Synthesis of 2e

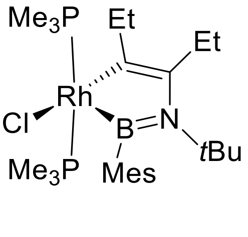

1e (496 mg, 852 μmol) was dissolved in benzene (10 mL) and treated with a stock solution of trimethylphosphine in benzene (2.5 mL, 1.96 mmol, 0.784 M). After stirring the reaction mixture for 4 h, all volatiles were removed *in vacuo*. The residue was washed with pentane (3 x 3 mL) and dried under reduced pressure to yield **2e** as a yellow solid (386 mg, 673 μmol,

79%). Crystals of **2e** suitable for X-ray diffraction were obtained by slow evaporation of a saturated hexane solution at −30 °C.

^{1}H NMR (500.1 MHz, C_6D_6, 298 K): δ = 6.81 (s, 2H, Mes-C*H*), 2.58 (s, 6H, Mes-CH_3), 2.53 (q, $^{3}J_{HH}$ = 7.3 Hz, 2H, Et-CH_2), 2.36 (qd, $^{3}J_{HH}$ = 7.6 Hz, $^{3}J_{RhH}$ = 1.2 Hz, 2H, Et-CH_2), 2.18 (s, 3H, Mes-CH_3), 1.34 (t, $^{3}J_{HH}$ = 7.3 Hz, 3H, Et-CH_3), 1.20 (s, 9H, *t*Bu-CH_3), 1.15 (t, $^{3}J_{HH}$ = 7.3 Hz, 3H, Et-CH_3) overlapping with 1.13 (vtd, *N* = 6.6 Hz, $^{3}J_{RhH}$ = 0.9 Hz, 18H, P(CH_3)$_3$) ppm.

^{1}H{^{31}P} NMR (500.1 MHz, C_6D_6, 298 K): δ= 6.804-6.803 (m, 2H, Mes-C*H*), 2.57 (s, 6H, Mes-CH_3), 2.53 (q, $^{3}J_{HH}$ = 7.3 Hz, 2H, Et-CH_2), 2.35 (qd, $^{3}J_{HH}$ = 7.6 Hz, $^{3}J_{RhH}$ = 1.2 Hz, 2H, Et-CH_2), 2.18 (s, 3H, Mes-CH_3), 1.34 (t, $^{3}J_{HH}$ = 7.6 Hz, 3H, Et-CH_3), 1.20 (s, 9H, *t*Bu-CH_3), 1.15 (t, $^{3}J_{HH}$ = 7.3 Hz, 3H, Et-CH_3) overlapping with 1.13 (d, $^{3}J_{RhH}$ = 0.9 Hz, 18H, P(CH_3)$_3$) ppm.

Comment: The spectrum contains a signal corresponding to residual benzene at 7.156 ppm

^{13}C{^{1}H} NMR (125.8 MHz, C_6D_6, 298 K): δ = 145.4 (t, $^{3}J_{PC}$ = 4.4 Hz, N*C*Et), 143.4 (br s, Mes-*C*$_q$,), 138.37 (dt, $^{1}J_{RhC}$ = 32.3 Hz, $^{2}J_{PC}$ = 10.3 Hz, Rh*C*Et) overlapping with 138.36 (t, $^{4}J_{PC}$ = 0.7 Hz, Mes-*C*$_q$), 136.7 (s, Mes-*C*$_q$), 128.6 (s, Mes-*C*H), 54.9 (d, $^{3}J_{RhC}$ = 1.1 Hz, *t*Bu-*C*$_q$), 32.8 (s, *t*Bu-*C*H$_3$), 30.5 (dt, $^{2}J_{RhC}$ = 0.5 Hz, $^{3}J_{PC}$ = 3.2 Hz, Et-*C*H$_2$), 26.7 (s, Mes-*C*H$_3$), 22.89-22.85 (m, Et-*C*H$_2$), 21.3 (s, Mes-*C*H$_3$), 18.4 (d, $^{3}J_{RhC}$ = 0.9 Hz, Et-*C*H$_3$), 15.7 (vtd, *N* = 28.4 Hz, $^{2}J_{RhC}$ = 1.4 Hz, P(*C*H$_3$)$_3$), 14.8 (dt, $^{4}J_{RhC}$ = 0.4 Hz, $^{5}J_{PC}$ = 2.7 Hz, Et-*C*H$_3$) ppm.

Comment: The spectrum contains signals corresponding to residual hexane from crystallization at 31.97, 23.06 and 14.36 ppm.

^{11}B NMR (160.5 MHz, C_6D_6, 298 K): δ = 69.4 (br s) ppm.

^{31}P{^{1}H} NMR (202.5 MHz, C_6D_6, 298 K): δ = −6.5 (d, $^{1}J_{RhP}$ = 133 Hz, 2 P) ppm.

HRMS (LIFDI, $C_{25}H_{48}BClNP_2Rh$): *calcd*: m/z = 573.2093, *found*: m/z = 573.2082.

UV-vis (hexane): λ_{abs} = 248 (shoulder), 285 (shoulder), 347, 413 (shoulder) nm.

Synthesis of 2f

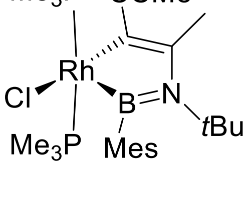

1f (430 mg, 744 μmol) was dissolved in benzene (10 mL) and treated with a stock solution of trimethylphosphine in benzene (2.20 mL, 1.65 mmol, 0.75 M). After stirring the reaction mixture for 1 h, all volatiles were removed *in vacuo* and the residue was dissolved in 5 mL hexane. The yellow solution was filtrated and compound **2f** crystallized at −30 °C. The yellow crystals were washed with hexane (2 x 2 mL) at −30 °C and dried under reduced pressure to yield **2f** as a yellow crystalline solid (352 mg, 618 μmol, 83%). Crystals of **2f** suitable for X-ray diffraction were also obtained by slow evaporation of a saturated benzene solution.

^{1}H NMR (500.1 MHz, C_6D_6, 298 K): δ = 6.80 (s, 2H, Mes-C*H*), 2.52 (t, $^{6}J_{PH}$ = 2.5 Hz, 3H, RhCCCH_3), 2.46 (s, 6H, Mes-CH_3), 2.18 (s, 3H, Mes-CH_3), 1.90 (s, 3H, RhCCCCH_3), 1.25 (vtd, *N* = 7.1 Hz, $^{3}J_{RhH}$ = 0.8 Hz, 18H, P(CH_3)$_3$), 1.17 (s, 9H, *t*Bu-CH_3) ppm.

^{1}H{^{31}P} NMR (500.1 MHz, C_6D_6, 298 K): δ = 6.799-6.796 (m, 2H, Mes-C*H*), 2.51 (s, 3H, RhCCCH_3), 2.46 (s, 6H, Mes-CH_3), 2.18 (s, 3H, Mes-CH_3), 1.90 (s, 3H, RhCCCCH_3), 1.25 (d, $^{3}J_{RhH}$ = 0.8 Hz, 18H, P(CH_3)$_3$), 1.17 (s, 9H, *t*Bu-CH_3) ppm.

Comment: The spectrum contains signals corresponding to residual hexane from crystallization at 1.25 (m) and 0.88 (t) ppm.

^{13}C{^{1}H} NMR (125.8 MHz, C_6D_6, 298 K): δ = 151.8 (dt, $^{2}J_{RhC}$ = 0.7 Hz, $^{3}J_{PC}$ = 4.5 Hz, RhC*C*CH$_3$), 143.0 (br s, Mes-*C*$_q$), 137.6 (t, $^{4}J_{PC}$ = 0.8 Hz, Mes-*C*$_q$), 136.8 (s, Mes-*C*$_q$), 128.3 (s, Mes-*C*H), 112.7 (dt, $^{1}J_{RhC}$ = 30.1 Hz, $^{2}J_{PC}$ = 10.9 Hz, Rh*C*CCCH$_3$), 94.3 (d, $^{3}J_{RhC}$ = 1.0 Hz, RhCC*C*CH$_3$), 84.5 (dt, $^{2}J_{RhC}$ = 1.1 Hz, $^{3}J_{PC}$ = 3.2 Hz, RhC*C*CCH$_3$), 55.8 (d, $^{3}J_{RhC}$ = 1.2 Hz, *t*Bu-*C*$_q$), 32.7 (s, *t*Bu-*C*H$_3$), 26.5 (t, $^{5}J_{PC}$ = 1.1 Hz, Mes-*C*H$_3$), 21.4 (s, Mes-*C*H$_3$), 21.23-21.19 (m, RhCC*C*H$_3$), 13.9 (vtd, *N* = 28.7 Hz, $^{2}J_{RhC}$ = 1.2 Hz, P(*C*H$_3$)$_3$), 5.5 (s, RhCCC*C*H$_3$) ppm.

^{13}C{^{1}H, ^{31}P} NMR (75.5 MHz, C_6D_6, 298 K): δ = 151.8 (d, $^{2}J_{RhC}$ = 0.8 Hz, RhC*C*CH$_3$), 137.6 (Mes-*C*$_q$), 136.8 (s, Mes-*C*$_q$), 128.3 (s, Mes-*C*H), 112.7 (d, $^{1}J_{RhC}$ = 30.1 Hz, Rh*C*CCCH$_3$), 94.4 (d, $^{3}J_{RhC}$ = 1.0 Hz, RhCC*C*CH$_3$), 84.5 (d, $^{2}J_{RhC}$ = 1.1 Hz, RhC*C*CCH$_3$), 55.8 (d, $^{3}J_{RhC}$ = 1.2 Hz, *t*Bu-*C*$_q$), 32.8 (s, *t*Bu-*C*H$_3$), 26.5 (s, Mes-*C*H$_3$), 21.4 (s, Mes-*C*H$_3$), 21.2 (d, $^{3}J_{RhC}$ = 2.2 Hz, RhCC*C*H$_3$), 13.9 (d, $^{2}J_{RhC}$ = 1.3 Hz, P(*C*H$_3$)$_3$), 5.5 (s, RhCCC*C*H$_3$) ppm.

Comment: The spectrum contains signals corresponding to residual hexane from crystallization at 31.97, 23.06 and 14.35 ppm. The broad singlet for the Mes-C_q nucleus bound to the boron atom could not be observed in the ^{13}C{^{1}H, ^{31}P} NMR spectrum.

^{11}B NMR (160.5 MHz, C_6D_6, 298 K): δ = 70.2 (br s) ppm.

^{31}P{^{1}H} NMR (202.5 MHz, C_6D_6, 298 K): δ = −3.7 (d, $^{1}J_{RhP}$ = 128 Hz, 2 P) ppm.

HRMS (LIFDI, $C_{25}H_{44}BClNP_2Rh$): *calcd*: m/z = 569.1780, *found*: m/z = 569.1767.

UV-vis (hexane): λ_{abs} = 260, 345 nm.

Synthesis of 2g

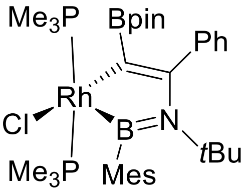

1g (325 mg, 447 μmol) was dissolved in benzene (10 mL) and treated with a stock solution of trimethylphosphine in benzene (2.61 mL, 1.96 mmol, 0.75 M). After stirring the reaction mixture for 4 d, all volatiles were removed *in vacuo* and the residue was dissolved in 10 mL benzene. The orange solution was filtrated and the solvent was removed under reduced pressure. After washing the residue with hexane (3 x 4 mL) all volatiles were removed *in vacuo* to yield **2g** as a yellow solid (114 mg, 158 μmol, 35%). Crystals of **2g** suitable for X-ray diffraction were obtained by slow evaporation of a saturated hexane solution at −30 °C.

^{1}H NMR (500.1 MHz, C_6D_6, 298 K): δ = 7.54-7.52 (m, 2H, Ph-C*H*), 7.19-7.17 (m, 2H, Ph-C*H* overlapping with C_6D_6), 7.13-7.10 (m, 1H, Ph-C*H*), 6.85 (s, 2H, Mes-C*H*), 2.70 (s, 6H, Mes-C*H*$_3$), 2.20 (s, 3H, Mes-C*H*$_3$), 1.37 (vtd, *N* = 7.1 Hz, $^{3}J_{RhH}$ = 0.8 Hz, 18H, P(C*H*$_3$)$_3$), 1.13 (s, 9H, *t*Bu-C*H*$_3$), 0.86 (s, 12H, Bpin-C*H*$_3$) ppm.

^{13}C{^{1}H} NMR (125.8 MHz, C_6D_6, 298 K): δ = 157.1 (br s, N*C*Ph), 143.9-143.8 (m, Ph-C_q), 143.0 (br s, Mes-*C*$_q$), 138.5 (t, $^{4}J_{PC}$ = 0.7 Hz, Mes-*C*$_q$), 137.0 (s, Mes-*C*$_q$), 131.2 (t, $^{5}J_{PC}$ = 1.8 Hz, Ph-*C*H), 128.5 (s, Mes-*C*H), 126.9 (s, Ph-*C*H), 126.2 (s, Ph-*C*H), 81.8 (s, Bpin-*C*$_q$), 56.7 (d, $^{3}J_{RhC}$ = 1.3 Hz, *t*Bu-*C*$_q$), 33.0 (s, *t*Bu-*C*H$_3$), 26.96-26.95 (m, Mes-*C*H$_3$), 25.0 (s, Bpin-*C*H$_3$), 21.4 (s, Mes-*C*H$_3$), 14.7 (vtd, *N* = 28.9 Hz, $^{2}J_{RhC}$ = 1.3 Hz, P(*C*H$_3$)$_3$) ppm.

Comment: The spectrum contains signals corresponding to residual hexane at 31.97, 23.06 and 14.35 ppm. The RhCBpin carbon nucleus was not observed in the ^{13}C{^{1}H} NMR spectrum.

^{11}B NMR (160.5 MHz, C_6D_6, 298 K): δ = 71.3 (br s, Rh*B*Mes), 30.3 (br s, Bpin-*B*) ppm.

^{31}P{^{1}H} NMR (202.5 MHz, C_6D_6, 298 K): δ = −4.9 (d, $^{1}J_{RhP}$ = 128 Hz, 2 P) ppm.

HRMS (LIFDI, $C_{33}H_{55}B_2ClNO_2P_2Rh$): *calcd*: m/z = 719.2632, *found*: m/z = 719.2620.

UV-vis (hexane): λ_{abs} = 255, 291 (shoulder), 362 nm.

Synthesis of 2l

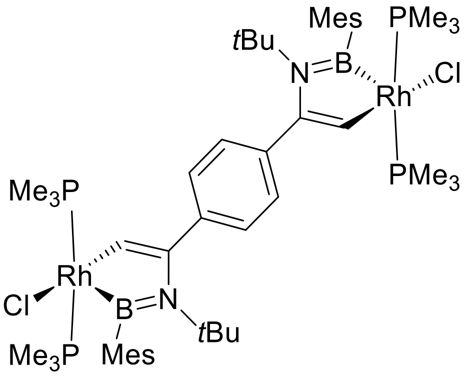

1l (100 mg, 88.8 µmol) was dissolved in benzene (10 mL) and treated with a stock solution of trimethylphosphine in benzene (0.52 mL, 390 µmol, 0.75 M). After stirring the reaction mixture for 15 h, all volatiles were removed *in vacuo*. The residue was first washed with pentane (3 x 2 mL), then with benzene (3 x 2 mL), and then dried under reduced pressure to yield **2l** as a yellow solid (59 mg, 53.2 µmol, 60%). Crystals of **2l** suitable for X-ray diffraction were obtained by evaporation of a saturated benzene solution.

^{1}H NMR (400.6 MHz, C_6D_6, 298 K): δ = 7.43 (s, 4H, C_6H_4-C*H*), 6.88 (s, 4H, Mes-C*H*), 5.92 (t, $^{3}J_{PH}$ = 5.0 Hz, 2H, RhC*H*), 2.67 (s, 12H, Mes-CH_3), 2.21 (s, 6H, Mes-CH_3), 1.24 (s, 18H, *t*Bu-CH_3), 1.17 (vt, *N* = 6.2 Hz, 36H, P(CH_3)$_3$) ppm.

^{13}C{^{1}H} NMR (125.8 MHz, C_6D_6, 298 K): δ = 153.7 (RhC*C*$_q$, detected by HMBC), 142.3 (C_6H_4-C_q, detected by HMBC), 142.3 (Mes-*C*$_q$, detected by HMBC), 138.1 (s, Mes-*C*$_q$), 137.2 (s, Mes-*C*$_q$), 131.3 (Rh*C*H, detected by HSQC), 128.6 (s, Mes-*C*H), 128.2 (C_6H_4-*C*H, detected by HSQC), 56.4 (d, $^{3}J_{RhC}$ = 1.1 Hz, *t*Bu-*C*$_q$), 32.8 (s, *t*Bu-*C*H_3), 26.62-26.60 (m, Mes-*C*H_3), 21.4 (s, Mes-*C*H_3), 14.1 (vtd, *N* = 29.1 Hz, $^{2}J_{RhC}$ = 1.2 Hz, P(*C*H_3)$_3$) ppm.

^{11}B NMR (128.5 MHz, C_6D_6, 298 K): not observed due to poor solubility.

^{31}P{^{1}H} NMR (162.2 MHz, C_6D_6, 298 K): δ = −4.5 (d, $^{1}J_{RhP}$ = 128 Hz, 4 P) ppm.

HRMS (LIFDI, $C_{48}H_{82}B_2Cl_2N_2P_4Rh_2$): *calcd*: m/z = 1108.3096, *found*: m/z = 1108.3095.

UV-vis (THF): λ_{abs} = 255 (shoulder), 299, 344 (shoulder) nm.

Synthesis of 3a^{Me}

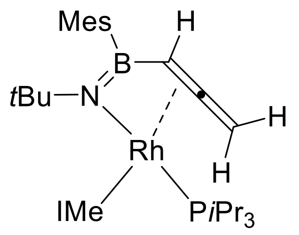

1a (200 mg, 371 μmol) and IMe (106.9 mg, 1.11 mmol) were dissolved in benzene (8 mL). After stirring the reaction mixture for 15 h, the resulting precipitate was filtered off and washed with benzene (3 x 8 mL). After removing all volatiles from the combined benzene fractions *in vacuo,* the residue was washed with pentane (3 x 5 mL) and benzene (2 x 2 mL), and dried under reduced pressure to yield **3a^{Me}** as a yellow solid (89 mg, 148 μmol, 40%). Crystals of **3a^{Me}** suitable for X-ray diffraction were obtained by evaporation of a saturated benzene-d_6 solution.

Comment: The residue of the filtration/extraction was identified as 1,3-dimethylimidazolium chloride by ^{1}H NMR spectroscopy.

^{1}H NMR (500.1 MHz, C_6D_6, 298 K): δ = 7.06 (s, 1H, Mes-C*H*), 7.02 (s, 1H, Mes-C*H*), 6.12 (d, $^3J_{HH}$ = 1.8 Hz, 1H, IMe-NC*H*), 6.05 (d, $^3J_{HH}$ = 1.9 Hz, 1H, IMe-NC*H*), 5.34-5.32 (m, 1H, CCH_2), 4.85-4.84 (m, 1H, CCH_2), 3.91 (s, 3H, IMe-CH_3), 3.22 (s, 3H, IMe-CH_3), 3.05 (s, 3H, Mes-CH_3), 2.81 (s, 3H, Mes-CH_3), 2.52-2.50 (m, 1H, C*H*CCH_2), 2.34 (s, 3H, Mes-CH_3), 1.88-1.81 (m, 3H, *i*Pr-C*H*), 1.18 (dd, $^3J_{HH}$ = 7.3 Hz, $^3J_{PH}$ = 12.6 Hz, 9H, *i*Pr-CH_3), 1.04 (s, 9H, *t*Bu-CH_3), 0.77 (dd, $^3J_{HH}$ = 7.3 Hz, $^3J_{PH}$ = 11.4 Hz, 9H, *i*Pr-CH_3) ppm.

Comment: The spectrum contains signals corresponding to residual pentane at 1.25 (m) and 0.87 (t) ppm

^{1}H{^{31}P} NMR (500.1 MHz, C_6D_6, 298 K): δ = 7.06 (s, 1H, Mes-C*H*), 7.02 (s, 1H, Mes-C*H*), 6.12 (d, $^3J_{HH}$ = 1.8 Hz, 1H, IMe-NC*H*), 6.05 (d, $^3J_{HH}$ = 1.8 Hz, 1H, IMe-NC*H*), 5.34-5.32 (m, 1H, CCH_2), 4.85-4.83 (m, 1H, CCH_2), 3.91 (s, 3H, IMe-CH_3), 3.22 (s, 3H, IMe-CH_3), 3.05 (s, 3H, Mes-CH_3), 2.81 (s, 3H, Mes-CH_3), 2.52-2.50 (m, 1H, C*H*CCH_2), 2.34 (s, 3H, Mes-CH_3), 1.84 (sept, $^3J_{HH}$ = 7.2 Hz, 3H, *i*Pr-C*H*), 1.18 (d, $^3J_{HH}$ = 7.2 Hz, 9H, *i*Pr-CH_3), 1.04 (s, 9H, *t*Bu-CH_3), 0.77 (d, $^3J_{HH}$ = 7.2 Hz, 9H, *i*Pr-CH_3) ppm.

^{13}C{^{1}H} NMR (125.8 MHz, C_6D_6, 298 K): δ = 192.8 (dd, $^1J_{RhC}$ = 60.7 Hz, $^2J_{PC}$ = 16.0 Hz, IMe-C_q), 188.8 (dd, $^1J_{RhC}$ = 20.5 Hz, $^2J_{PC}$ = 4.8 Hz, *C*CH_2), 145.7 (Mes-C_q, detected by

HMBC), 140.1 (s, Mes-C_q), 137.4 (s, Mes-C_q), 134.6 (s, Mes-C_q), 128.0 (Mes-*C*H, detected by HSQC), 127.1 (s, Mes-*C*H), 122.2 (d, $^3J_{RhC}$ = 1.4 Hz, IMe-N*C*H), 121.2 (d, $^3J_{RhC}$ = 1.3 Hz, IMe-N*C*H), 85.90-85.88 (m, C*C*H_2), 54.4 (dd, $^2J_{RhC}$ = 0.9 Hz, $^3J_{PC}$ = 3.6 Hz, *t*Bu-C_q), 41.6 (br s, *C*HCCH_2), 39.1 (d, $^3J_{RhC}$ = 1.2 Hz, IMe-*C*H_3), 38.7 (d, $^3J_{RhC}$ = 0.6 Hz, IMe-*C*H_3), 34.4-34.3 (m, *t*Bu-*C*H_3), 25.9 (dd, $^1J_{PC}$ = 17.4 Hz, $^2J_{RhC}$ = 0.8 Hz, *i*Pr-*C*H), 24.1 (s, Mes-*C*H_3), 23.6 (s, Mes-*C*H_3), 21.6 (s, Mes-*C*H_3), 20.9 (d, $^2J_{PC}$ = 1.9 Hz, *i*Pr-*C*H_3), 19.0 (s, *i*Pr-*C*H_3) ppm.

Comment: The spectrum contains signals corresponding to residual pentane at 34.45, 22.72 and 14.28 ppm.

^{13}C{^{1}H, ^{31}P} NMR (75.5 MHz, C_6D_6, 298 K): δ = 192.8 (d, $^1J_{RhC}$ = 60.6 Hz, IMe-C_q), 188.8 (d, $^1J_{RhC}$ = 20.5 Hz, *C*CH_2), 140.1 (s, Mes-C_q), 137.4 (s, Mes-C_q), 134.6 (s, Mes-C_q), 128.0 (s, Mes-*C*H), 127.1 (s, Mes-*C*H), 122.2 (d, $^3J_{RhC}$ = 1.6 Hz, IMe-N*C*H), 121.2 (d, $^3J_{RhC}$ = 1.5 Hz, IMe-N*C*H), 85.9 (d, $^2J_{RhC}$ = 1.0 Hz, C*C*H_2), 54.4 (d, $^2J_{RhC}$ = 0.9 Hz, *t*Bu-C_q), 39.1 (d, $^3J_{RhC}$ = 1.3 Hz, IMe-*C*H_3), 38.7 (d, $^3J_{RhC}$ = 0.7 Hz, IMe-*C*H_3), 34.4 (d, $^3J_{RhC}$ = 0.8 Hz, *t*Bu-*C*H_3), 25.9 (d, $^2J_{RhC}$ = 1.0 Hz, *i*Pr-*C*H), 24.1 (s, Mes-*C*H_3), 23.6 (s, Mes-*C*H_3), 21.6 (s, Mes-*C*H_3), 20.9 (d, $^3J_{RhC}$ = 0.4 Hz, *i*Pr-*C*H_3), 19.0 (s, *i*Pr-*C*H_3) ppm.

Comment: The signals corresponding to the Mes-C_q and CHCCH_2 *nuclei bound to the boron atom could not be observed in the ^{13}C{^{1}H, ^{31}P} NMR spectrum.*

^{11}B NMR (128.5 MHz, C_6D_6, 298 K): δ = 33.6 (br s) ppm.

^{31}P{^{1}H} NMR (162.2 MHz, C_6D_6, 298 K): δ = 48.8 (d, $^1J_{RhP}$ = 140 Hz) ppm.

HRMS (LIFDI, $C_{30}H_{52}BN_3PRh$): *calcd*: m/z = 599.3041, *found*: m/z = 599.3032.

UV-vis (THF): λ_{abs} = 396 nm.

Synthesis of 3e^{Me}

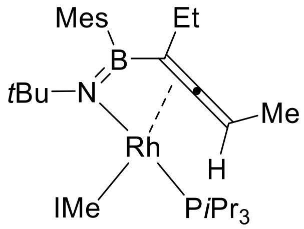

1e (25.0 mg, 43.0 μmol) and IMe (9.9 mg, 103 μmol) were dissolved in benzene (0.6 mL). After 30 mins at room temperature, the suspension was filtered, and all volatiles of the filtrate were removed *in vacuo*. The residue of the filtrate was treated with hexane (0.6 mL) and the resulting suspension was filtered. After storing the filtrate at −30 °C for 8 d a yellow crystalline solid formed. The solid was washed with benzene-d_6 and dried under reduced pressure to yield

3e^{Me}. Crystals of **3e^{Me}** suitable for X-ray diffraction were obtained by slow evaporation of a saturated hexane solution at −30 °C.

^{1}H NMR (500.1 MHz, C_6D_6, 298 K): δ = 7.073-7.070 (m, 1H, Mes-C*H*), 7.014-7.010 (m, 1H, Mes-C*H*), 6.09 (d, $^3J_{HH}$ = 1.9 Hz, 1H, IMe-NC*H*), 6.03 (d, $^3J_{HH}$ = 1.9 Hz, 1H, IMe-NC*H*), 5.32 (q, $^3J_{HH}$ = 6.5 Hz, 1H, CC*H*CH_3), 3.80 (s, 3H, IMe-CH_3), 3.51 (s, 3H, IMe-CH_3), 3.03 (s, 3H, Mes-CH_3), 2.78 (s, 3H, Mes-CH_3), 2.35 (s, 3H, Mes-CH_3), 2.27 (d, $^3J_{HH}$ = 6.5 Hz, 3H, CCHCH_3), 2.13-2.06 (m, 1H, CC$H_2$$CH_3$), 1.80-1.73 (m, 3H, *i*Pr-C*H*), 1.71-1.64 (m, 1H, CC$H_2$$CH_3$), 1.26-1.20 (m, 12H, *i*Pr-CH_3 overlapping with CCH_2CH_3), 1.01-0.97 (m, 9H, *i*Pr-CH_3) overlapping with 0.98 (s, 9H, *t*Bu-CH_3) ppm.

^{13}C{^{1}H} NMR (125.8 MHz, C_6D_6, 298 K): δ = 193.4 (dd, $^1J_{RhC}$ = 59.0 Hz, $^2J_{PC}$ = 17.6 Hz, IMe-*C*$_q$), 183.0 (dd, $^1J_{RhC}$ = 17.4 Hz, $^2J_{PC}$ = 4.2 Hz, *C*CHCH_3), 145.5 (Mes-*C*$_q$, detected by HMBC), 139.7 (s, Mes-*C*$_q$), 138.2 (s, Mes-*C*$_q$), 134.7 (s, Mes-*C*$_q$), 127.9 (Mes-*C*H, detected by HSQC), 127.2 (s, Mes-*C*H), 122.0 (d, $^3J_{RhC}$ = 1.4 Hz, IMe-N*C*H), 121.1 (d, $^3J_{RhC}$ = 1.4 Hz, IMe-N*C*H), 96.04-96.03 (m, C*C*HCH_3), 54.4 (dd, $^2J_{RhC}$ = 1.2 Hz, $^3J_{PC}$ = 3.5 Hz, *t*Bu-*C*$_q$), 39.5 (d, $^3J_{RhC}$ = 0.8 Hz, IMe-*C*H_3), 38.8 (d, $^3J_{RhC}$ = 1.3 Hz, IMe-*C*H_3), 34.1 (dd, ($^2J_{RhC}$ or $^3J_{PC}$) = 0.6 Hz, ($^3J_{PC}$ or $^2J_{RhC}$) = 1.8 Hz, *t*Bu-*C*H_3), 27.6 (s, C*C*$H_2$$CH_3$), 27.4 (dd, $^1J_{PC}$ = 15.9 Hz, $^2J_{RhC}$ = 0.6 Hz, *i*Pr-*C*H), 24.5 (s, Mes-*C*H_3), 24.4 (s, Mes-*C*H_3), 21.6 (s, Mes-*C*H_3), 21.4 (d, $^2J_{PC}$ = 2.8 Hz, *i*Pr-*C*H_3), 19.5 (d, ($^3J_{RhC}$ or $^2J_{PC}$) = 0.7 Hz, *i*Pr-*C*H_3), 18.2 (d, ($^3J_{RhC}$ or $^4J_{PC}$) = 1.6 Hz, CCH*C*H_3), 15.4 (d, ($^3J_{RhC}$ or $^4J_{PC}$) = 0.9 Hz, CCH_2*C*H_3) ppm.

^{11}B NMR (160.5 MHz, C_6D_6, 298 K): δ = 34.7 (br s) ppm.

^{31}P{^{1}H} NMR (162.2 MHz, C_6D_6, 298 K): δ = 46.6 (d, $^1J_{RhP}$ = 144 Hz) ppm.

HRMS (LIFDI, $C_{33}H_{58}BN_3PRh$): *calcd*: m/z = 641.3511, *found*: m/z = 641.3498.

Synthesis of 4a^{iPr}

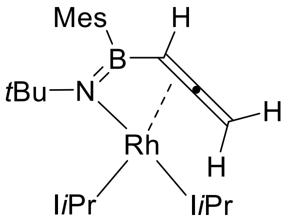

1a (30.0 mg, 55.6 μmol) and I*i*Pr (28.0 mg, 184 μmol) were dissolved in benzene (0.6 mL). After 6 d at room temperature the suspension was filtered. The filtrate was treated with benzene (0.2 mL), leading to precipitation of a yellow solid. The supernatant solution was removed by Pasteur pipette and the residue was washed with benzene (2 x 1 mL) and hexane (1 x 2 mL).

The residual solvent was removed *in vacuo* to yield **4a^{iPr}** as a yellow solid (10.0 mg, 15.4 µmol, 28%). Crystals of **4a^{iPr}** suitable for X-ray diffraction were obtained by evaporation of a saturated benzene solution.

Comment: The residue of the filtration was identified as 1,3-diisopropylimidazolium chloride by ^{1}H NMR spectroscopy.

^{1}H NMR (500.1 MHz, C_6D_6, 298 K): δ = 7.11-7.10 (m, 1H, Mes-C*H*), 7.021-7.017 (m, 1H, Mes-C*H*), 6.73 (sept, $^3J_{HH}$ = 6.7 Hz, 1H, *i*Pr-C*H*), 6.50 (d, $^3J_{HH}$ = 2.0 Hz, 1H, I*i*Pr-NC*H*), 6.44 (sept, $^3J_{HH}$ = 6.6 Hz, 1H, *i*Pr-C*H*), 6.28 (d, $^3J_{HH}$ = 2.2 Hz, 1H, I*i*Pr-NC*H*), 6.24 (d, $^3J_{HH}$ = 2.1 Hz, 1H, I*i*Pr-NC*H*), 6.16 (d, $^3J_{HH}$ = 2.1 Hz, 1H, I*i*Pr-NC*H*), 5.40-5.31 (m, 2H, *i*Pr-C*H* overlapping with CC*H*$_2$), 4.71-4.70 (m, 1H, CC*H*$_2$), 4.54 (sept, $^3J_{HH}$ = 6.8 Hz, 1H, *i*Pr-C*H*), 3.08 (s, 3H, Mes-C*H*$_3$), 2.89 (s, 3H, Mes-C*H*$_3$), 2.37 (s, 3H, Mes-C*H*$_3$), 1.86 (t, $^4J_{HH}$ = 3.5 Hz, 1H, C*H*CCH$_2$), 1.43 (d, $^3J_{HH}$ = 6.5 Hz, 3H, *i*Pr-C*H*$_3$), 1.30 (d, $^3J_{HH}$ = 6.7 Hz, 3H, *i*Pr-C*H*$_3$), 1.26 (s, 9H, *t*Bu-C*H*$_3$), 1.20 (d, $^3J_{HH}$ = 6.8 Hz, 3H, *i*Pr-C*H*$_3$), 1.14 (d, $^3J_{HH}$ = 6.9 Hz, 3H, *i*Pr-C*H*$_3$), 1.02 (d, $^3J_{HH}$ = 6.9 Hz, 3H, *i*Pr-C*H*$_3$), 0.98 (d, $^3J_{HH}$ = 6.7 Hz, 3H, *i*Pr-C*H*$_3$), 0.40 (d, $^3J_{HH}$ = 6.8 Hz, 3H, *i*Pr-C*H*$_3$), 0.35 (d, $^3J_{HH}$ = 6.7 Hz, 3H, *i*Pr-C*H*$_3$) ppm.

^{13}C{^{1}H} NMR (125.8 MHz, C_6D_6, 298 K): δ = 192.4 (d, $^1J_{RhC}$ = 65.7 Hz, I*i*Pr-*C*$_q$), 186.9 (d, $^1J_{RhC}$ = 24.5 Hz, *C*CH$_2$), 183.8 (d, $^1J_{RhC}$ = 48.3 Hz, I*i*Pr-*C*$_q$), 147.7 (Mes-*C*$_q$, detected by HMBC), 140.2 (s, Mes-*C*$_q$), 136.8 (s, Mes-*C*$_q$), 133.9 (s, Mes-*C*$_q$), 127.9 (Mes-*C*H, detected by HSQC), 127.0 (s, Mes-*C*H), 117.4 (d, $^3J_{RhC}$ = 1.8 Hz, I*i*Pr-N*C*H), 116.4 (d, $^3J_{RhC}$ = 0.8 Hz, I*i*Pr-N*C*H), 115.3 (d, $^3J_{RhC}$ = 1.1 Hz, I*i*Pr-N*C*H), 114.8 (d, $^3J_{RhC}$ = 1.4 Hz,, I*i*Pr-N*C*H), 85.9 (s, C*C*H$_2$), 54.6 (d, $^2J_{RhC}$ = 0.7 Hz, *t*Bu-*C*$_q$), 52.8 (s, *i*Pr-*C*H), 50.9 (s, *i*Pr-*C*H), 50.497 (s, *i*Pr-*C*H) overlapping with 50.488 (s, *i*Pr-*C*H), 36.1 (s, *t*Bu-*C*H$_3$), 34.0 (br s, *C*HCCH$_2$), 25.0 (s, *i*Pr-*C*H$_3$), 24.9 (s, *i*Pr-*C*H$_3$), 24.1 (s, *i*Pr-*C*H$_3$), 23.8 (s, Mes-*C*H$_3$), 23.5 (s, *i*Pr-*C*H$_3$), 23.4 (s, Mes-*C*H$_3$), 22.8 (s, *i*Pr-*C*H$_3$), 22.6 (s, *i*Pr-*C*H$_3$), 22.1 (s, *i*Pr-*C*H$_3$), 22.0 (s, *i*Pr-*C*H$_3$), 21.6 (s, Mes-*C*H$_3$) ppm.

^{11}B NMR (160.5 MHz, C_6D_6, 298 K): δ = 31.7 (br s) ppm.

HRMS (LIFDI, $C_{34}H_{55}BN_5Rh$): *calcd*: m/z = 647.3600, *found*: m/z = 647.3594.

UV-vis (THF): λ_{abs} = 380, 430 nm.

Synthesis of 4e^{iPr}

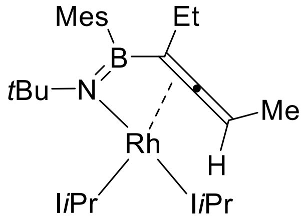

1e (30.0 mg, 51.6 μmol) and I*i*Pr (25.9 mg, 170 μmol) were dissolved in benzene (0.6 mL). After 4 d at room temperature, the suspension was filtered, and all volatiles of the filtrate were removed *in vacuo*. The residue of the filtrate was washed with hexane (3 x 1 mL) and dried under reduced pressure to yield **4e^{iPr}** as an orange solid (9.0 mg, 13.1 μmol, 25%). Crystals of **4e^{iPr}** suitable for X-ray diffraction were obtained by evaporation of a saturated hexane solution.

^{1}H NMR (500.1 MHz, C_6D_6, 298 K): δ = 7.122-7.119 (m, 1H, Mes-C*H*), 7.061-7.059 (m, 1H, Mes-C*H*), 6.58-6.50 (m, 2H, *i*Pr-C*H* overlapping with I*i*Pr-NC*H*), 6.36-6.28 (m, 2H, *i*Pr-C*H* overlapping with I*i*Pr-NC*H*), 6.26 (d, $^3J_{HH}$ = 2.0 Hz, 1H, I*i*Pr-NC*H*), 6.16 (d, $^3J_{HH}$ = 2.1 Hz, 1H, I*i*Pr-NC*H*), 5.40 (sept, $^3J_{HH}$ = 6.8 Hz, 1H, *i*Pr-C*H*), 4.94 (qd, $^3J_{HH}$ = 6.3 Hz, $^3J_{RhH}$ = 1.6 Hz, 1H, CC*H*CH$_3$), 4.50 (sept, $^3J_{HH}$ = 6.8 Hz, 1H, *i*Pr-C*H*), 3.08 (s, 3H, Mes-C*H*$_3$), 2.88 (s, 3H, Mes-C*H*$_3$), 2.39 (s, 3H, Mes-C*H*$_3$), 2.30 (d, $^3J_{HH}$ = 6.3 Hz, 3H, CCHC*H*$_3$), 1.94-1.86 (m, 1H, CC*H*$_2$CH$_3$), 1.48 (d, $^3J_{HH}$ = 6.5 Hz, 3H, *i*Pr-C*H*$_3$), 1.34-1.26 (m, 7H, CC*H*$_2$CH$_3$ overlapping with two *i*Pr-C*H*$_3$), 1.24 (s, 9H, *t*Bu-C*H*$_3$), 1.17-1.14 (m, 6H, *i*Pr-C*H*$_3$ overlapping with another *i*Pr-C*H*$_3$), 1.00 (d, $^3J_{HH}$ = 6.9 Hz, 3H, *i*Pr-C*H*$_3$), 0.73 (t, $^3J_{HH}$ = 7.4 Hz, 3H, CCH$_2$C*H*$_3$), 0.43 (d, $^3J_{HH}$ = 6.8 Hz, 3H, *i*Pr-C*H*$_3$), 0.32 (d, $^3J_{HH}$ = 6.7 Hz, 3H, *i*Pr-C*H*$_3$) ppm.

^{13}C{^{1}H} NMR (125.8 MHz, C_6D_6, 298 K): δ = 192.3 (d, $^1J_{RhC}$ = 67.2 Hz, I*i*Pr-*C*$_q$), 184.7 (d, $^1J_{RhC}$ = 48.4 Hz, I*i*Pr-*C*$_q$), 179.2 (d, $^1J_{RhC}$ = 26.5 Hz, *C*CHCH$_3$), 146.8 (br s, Mes-*C*$_q$), 139.7 (s, Mes-*C*$_q$), 137.3 (s, Mes-*C*$_q$), 133.8 (s, Mes-*C*$_q$), 127.7 (s, Mes-*C*H), 127.2 (s, Mes-*C*H), 117.6 (d, $^3J_{RhC}$ = 1.9 Hz, I*i*Pr-N*C*H), 116.6 (d, $^3J_{RhC}$ = 0.8 Hz, I*i*Pr-N*C*H), 115.1 (d, $^3J_{RhC}$ = 0.9 Hz, I*i*Pr-N*C*H), 114.6 (d, $^3J_{RhC}$ = 1.4 Hz, I*i*Pr-N*C*H), 95.2 (s, C*C*HCH$_3$), 54.6 (d, $^2J_{RhC}$ = 0.7 Hz, *t*Bu-*C*$_q$), 52.6 (s, *i*Pr-*C*H), 50.6 (d, $^3J_{RhC}$ = 0.8 Hz, *i*Pr-*C*H), 50.2 (s, *i*Pr-*C*H), 49.7 (d, $^3J_{RhC}$ = 1.3 Hz, *i*Pr-*C*H), 49.3 (br s, *C*CH$_2$CH$_3$), 36.2 (s, *t*Bu-*C*H$_3$), 27.4 (s, C*C*H$_2$CH$_3$), 24.9 (s, *i*Pr-*C*H$_3$), 24.6 (s, *i*Pr-*C*H$_3$), 24.5 (s, *i*Pr-*C*H$_3$), 24.3 (s, *i*Pr-*C*H$_3$), 23.70 (s, Mes-*C*H$_3$), 23.68 (s, Mes-*C*H$_3$), 23.4 (s, *i*Pr-*C*H$_3$), 22.5 (s, *i*Pr-*C*H$_3$), 22.1 (s, *i*Pr-*C*H$_3$), 21.75 (s, *i*Pr-*C*H$_3$), 21.66 (s, Mes-*C*H$_3$), 18.3 (d, $^3J_{RhC}$ = 3.5 Hz, CCH*C*H$_3$), 13.5 (s, CCH$_2$*C*H$_3$) ppm.

Comment: The spectrum contains signals corresponding to residual hexane at 31.97, 23.06 and 14.35 and residual benzene at 128.59 ppm.

^{11}B NMR (160.5 MHz, C_6D_6, 298 K): δ = 32.3 (br s) ppm.

HRMS (LIFDI, $C_{37}H_{61}BN_5Rh$): *calcd*: m/z = 689.4070, *found*: m/z = 689.4058.

UV-vis (THF): λ_{abs} = 351 (shoulder), 449 nm.

Synthesis of 5

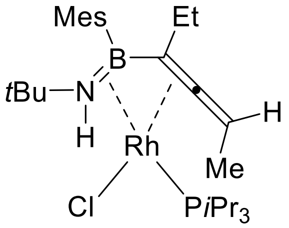

1e (30.0 mg, 51.6 μmol) was dissolved in benzene-d_6 (0.6 mL). After 4 d at 80 °C, all volatiles were removed *in vacuo* and the residue was washed with hexane (3 x 1 mL). Drying under reduced pressure yielded **5** as a yellow solid. Crystals of **5** suitable for X-ray diffraction were obtained by slow evaporation of a saturated pentane solution at −30 °C.

Comment: Crystals of ***6*** *suitable for X-ray diffraction were obtained by evaporation of the hexane filtrate/washing solution.*

^{1}H NMR (500.1 MHz, C_6D_6, 298 K): δ = 6.95 (s, 1H, Mes-C*H*), 6.87 (s, 1H, Mes-C*H*), 5.10 (q, $^3J_{HH}$ = 6.7 Hz, 1H, CC*H*CH$_3$), 3.36 (s, 3H, Mes-C*H*$_3$), 2.33 (s, 3H, Mes-C*H*$_3$) overlapping with 2.32 (d, $^3J_{HH}$ = 6.8 Hz, 3H, CCHC*H*$_3$), 2.28-2.20 (m, 3H, *i*Pr-C*H*) overlapping with 2.22 (s, 3H, Mes-C*H*$_3$), 2.11-2.10 (m, 1H, N*H*), 1.95-1.88 (m, 1H, Et-C*H*$_2$), 1.38 (s, 9H, *t*Bu-C*H*$_3$), 1.21-1.16 (m, 19H, *i*Pr-C*H*$_3$ overlapping with Et-C*H*$_2$), 1.03 (t, $^3J_{HH}$ = 7.5 Hz, 3H, Et-C*H*$_3$) ppm.

^{13}C{^{1}H} NMR (125.8 MHz, C_6D_6, 298 K): δ = 168.2 (dd, $^1J_{RhC}$ = 20.5 Hz, $^2J_{PC}$ = 4.7 Hz, *C*CHCH$_3$), 140.4 (s, Mes-*C*$_q$), 139.0 (s, Mes-*C*$_q$), 137.1 (s, Mes-*C*$_q$), 135.3 (Mes-*C*$_q$, detected by HMBC), 128.3 (Mes-*C*H, detected by HSQC), 127.8 (Mes-*C*H, detected by HSQC), 101.97-101.95 (m, C*C*HCH$_3$), 58.2 (B*C*Et, detected by HMBC), 54.7 (d, $^2J_{RhC}$ = 0.5 Hz, *t*Bu-*C*$_q$), 30.3 (d, ($^3J_{RhC}$ or $^4J_{PC}$) = 2.1 Hz, *t*Bu-*C*H$_3$), 28.1 (s, Mes-*C*H$_3$), 25.1 (dd, $^1J_{PC}$ = 23.2 Hz, $^2J_{RhC}$ = 1.1 Hz, *i*Pr-*C*H), 25.0 (s, Et-*C*H$_2$), 23.6 (s, Mes-*C*H$_3$), 21.3 (s, Mes-*C*H$_3$), 20.8 (s, *i*Pr-*C*H$_3$), 19.2 (($^3J_{RhC}$ or $^2J_{PC}$) = 1.3 Hz, *i*Pr-*C*H$_3$), 16.5 (d, ($^3J_{RhC}$ or $^4J_{PC}$) = 1.1 Hz, CCH*C*H$_3$), 13.3 (dd, ($^3J_{RhC}$ or $^4J_{PC}$) = 0.7 Hz, ($^4J_{PC}$ or $^3J_{RhC}$) = 1.6 Hz, Et-*C*H$_3$) ppm.

^{11}**B NMR** (128.5 MHz, C_6D_6, 298 K): δ = 29.3 (br s) ppm.
^{31}**P{^{1}H} NMR** (202.5 MHz, C_6D_6, 298 K): δ = 53.9 (d, $^1J_{RhP}$ = 181 Hz) ppm.

Irradiation of 5

The irradiation of a solution of **5** in benzene-d_6 with a mercury-xenon vapor lamp for 2 h led to complete conversion to a compound with a major signal in the ^{11}B NMR spectrum at 40.4 ppm and a doublet at 47.6 ppm ($^1J_{RhP}$ = 169 Hz) in the ^{31}P NMR spectrum, in addition to traces of side products. After removing all volatiles *in vacuo* the residue was dissolved in pentane. Crystals of **7** suitable for X-ray diffraction were obtained by slow evaporation of this solution at −30 °C.

Synthesis of 1b(IMe)

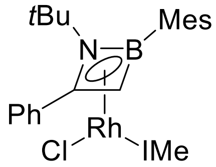

1b (30.0 mg, 49.8 µmol) and IMe (4.8 mg, 49.9 µmol) were dissolved in benzene (0.6 mL). After 2 h at room temperature, all volatiles were removed *in vacuo*. The residue was washed with hexane (3 x 1.5 mL), dried under reduced pressure and dissolved in benzene-d_6 (0.6 mL). After 6 d at room temperature, an orange solid precipitated. The supernatant solution was removed by Pasteur pipette. The residue was washed with benzene (ca. 0.3 mL) and dried under reduced pressure to yield **1b(IMe)**.

^{1}**H NMR** (500.1 MHz, C_6D_6, 298 K): δ = 8.14-8.12 (m, 2H, Ph-C*H*), 7.13-7.06 (m, 3H, Ph-C*H*), 6.92 (s, 2H, Mes-C*H*), 5.78 (s, 2H, IMe-NC*H*), 3.60 (s, 3H, Mes-CH_3), 3.38 (s, 6H, IMe-CH_3), 3.02 (s, 1H, Aza-C*H*), 2.75 (s, 3H, Mes-CH_3), 2.21 (s, 3H, Mes-CH_3), 1.33 (s, 9H, *t*Bu-CH_3) ppm.
^{13}**C{^{1}H} NMR** (125.8 MHz, C_6D_6, 298 K): δ = 181.7 (d, $^1J_{RhC}$ = 68.1 Hz, IMe-C_q), 141.8 (s, Mes-C_q), 140.9 (s, Mes-C_q), 138.1 (s, Mes-C_q), 135.5 (s, Ph-C_q), 132.2 (Mes-C_q, detected by HMBC), 131.1 (s, Ph-*C*H), 129.1 (s, Ph-*C*H), 128.7 (s, Mes-*C*H), 127.9 (Ph-*C*H, detected by HSQC), 127.7 (s, Mes-*C*H), 121.4 (s, IMe-N*C*H), 103.5 (d, $^1J_{RhC}$ = 14.6 Hz, Aza-C_q), 56.4 (s,

*t*Bu-*C*$_q$), 47.7 (br s, Aza-*C*H), 37.8 (s, IMe-*C*H$_3$), 29.6 (s, *t*Bu-*C*H$_3$), 26.7 (s, Mes-*C*H$_3$), 24.0 (s, Mes-*C*H$_3$), 21.3 (s, Mes-*C*H$_3$) ppm.

^{11}B NMR (128.5 MHz, C_6D_6, 298 K): δ = 20.3 (br s) ppm.

Reaction of 1a with PEt_3

1a (21.0 mg, 38.9 µmol) was dissolved in benzene-d$_6$ (0.5 mL) and PEt_3 (0.03 mL, 207 µmol) was added. After 30 mins at room temperature, complete conversion to compound **2a(PEt_3)** was observed by ^{11}B and ^{31}P NMR spectroscopy (assigned on the basis of similarity to **2a**).

^{11}B NMR (128.5 MHz, C_6D_6, 298 K): δ = 68.1 (br s) ppm.

^{31}P{^{1}H} NMR (162.2 MHz, C_6D_6, 298 K): δ = 12.5 (d, $^1J_{RhP}$ = 127 Hz, 2P) ppm.

Comment: The ^{31}P{^{1}H} NMR spectrum contains signals corresponding to P^iPr_3 at 19.5 ppm and PEt_3 at −19.7 ppm.

Reaction of 1a with PCy_3

1a (20.0 mg, 37.1 µmol) and PCy_3 (21.3 mg, 76.0 µmol) were dissolved in toluene (0.6 mL). The reaction mixture was heated at 80 °C for 2 d and then all volatiles were removed *in vacuo*. The residue was dissolved again in toluene (0.6 mL) and heated at 80 °C for 1 d. Again all volatiles were removed *in vacuo*, the residue was dissolved in toluene (0.6 mL) and heated at 80 °C for 8 h. Almost complete conversion to compound **1a(PCy_3)** was observed (assigned by ^{11}B and ^{31}P NMR spectroscopy on the basis of similarity to **1a**).

^{11}B NMR (128.5 MHz, toluene, 298 K): δ = 20.0 (br s) ppm.

^{31}P{^{1}H} NMR (162.2 MHz, toluene, 298 K): δ = 49.0 (d, $^1J_{RhP}$ = 197 Hz) ppm.

*Comment: The ^{31}P{^{1}H} NMR spectrum contains signals corresponding to starting material **1a** at 60.4 (d, $^1J_{RhP}$ = 197 Hz) ppm, P^iPr_3 at 18.8 ppm and PCy_3 at 9.0 ppm.*

NMR spectra

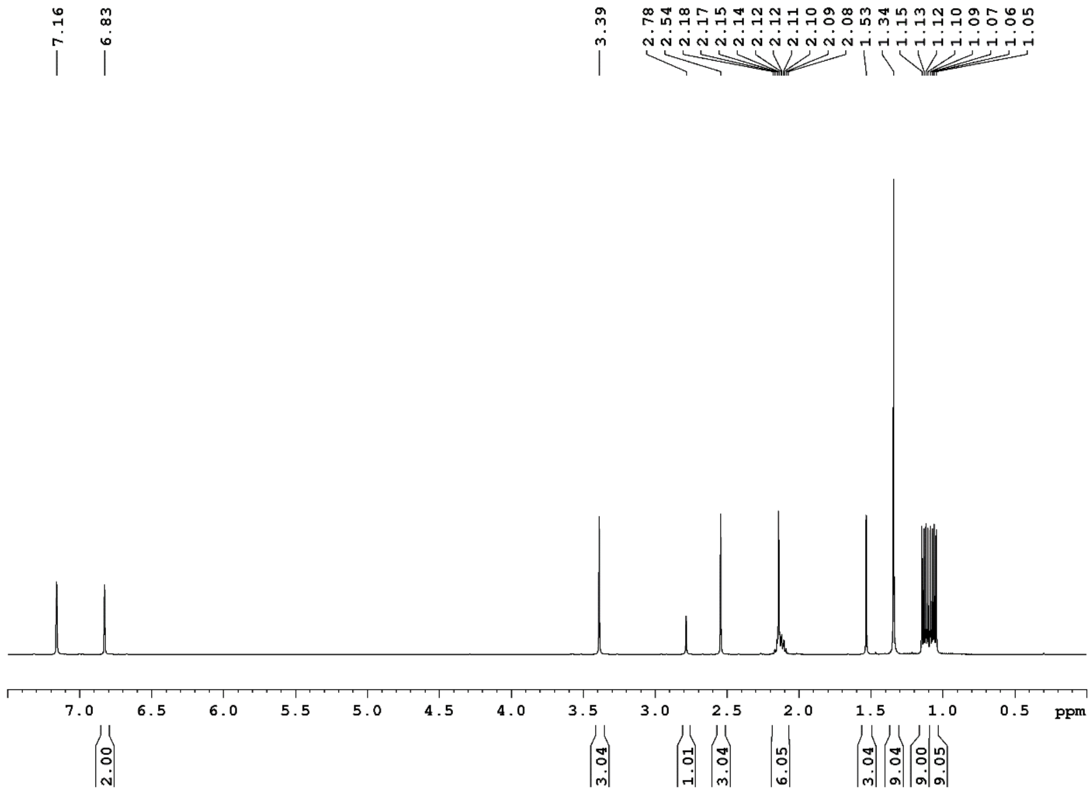

Figure S1. ^{1}H NMR (500.1 MHz, C_6D_6, 298 K) spectrum of **1a**

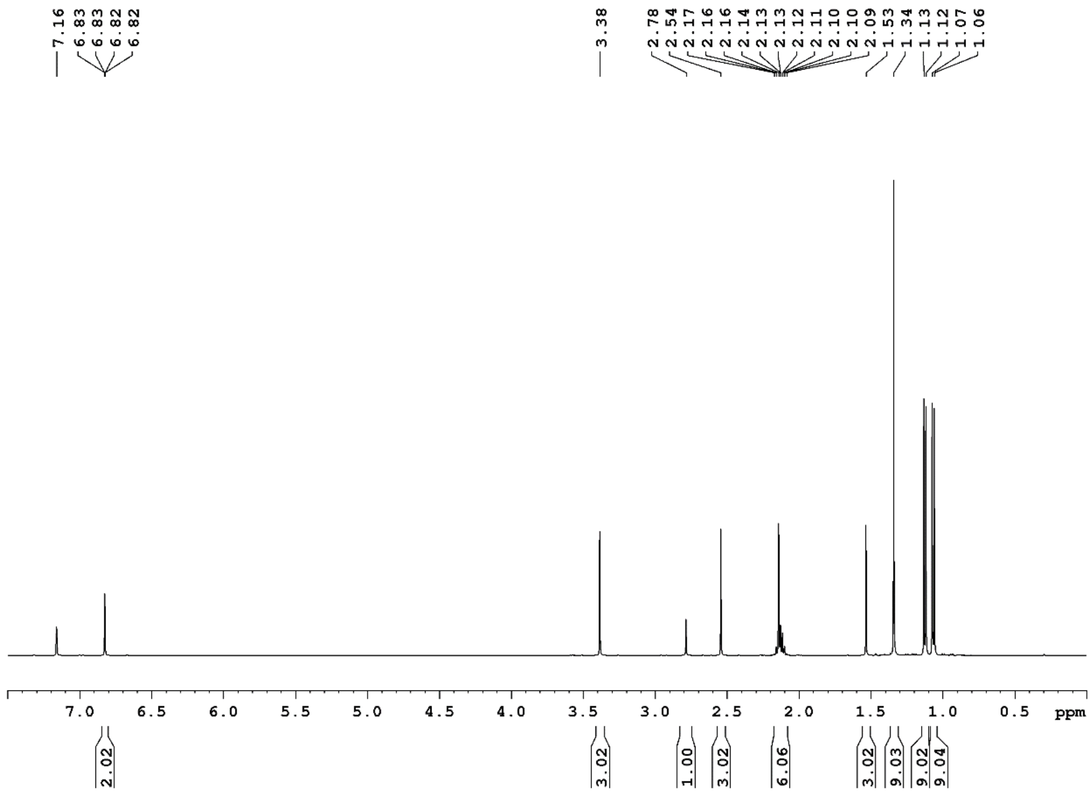

Figure S2. $^{1}H\{^{31}P\}$ NMR (500.1 MHz, C_6D_6, 298 K) spectrum of **1a**.

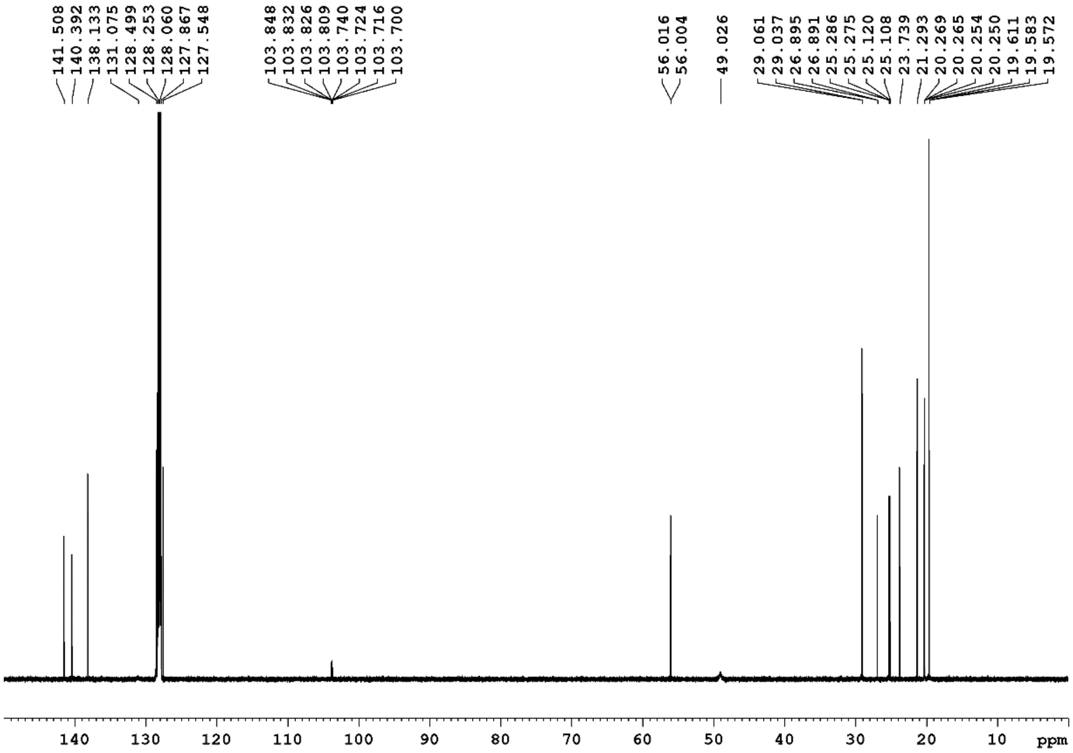

Figure S3. $^{13}C\{^{1}H\}$ NMR (125.8 MHz, C_6D_6, 298 K) spectrum of **1a**.

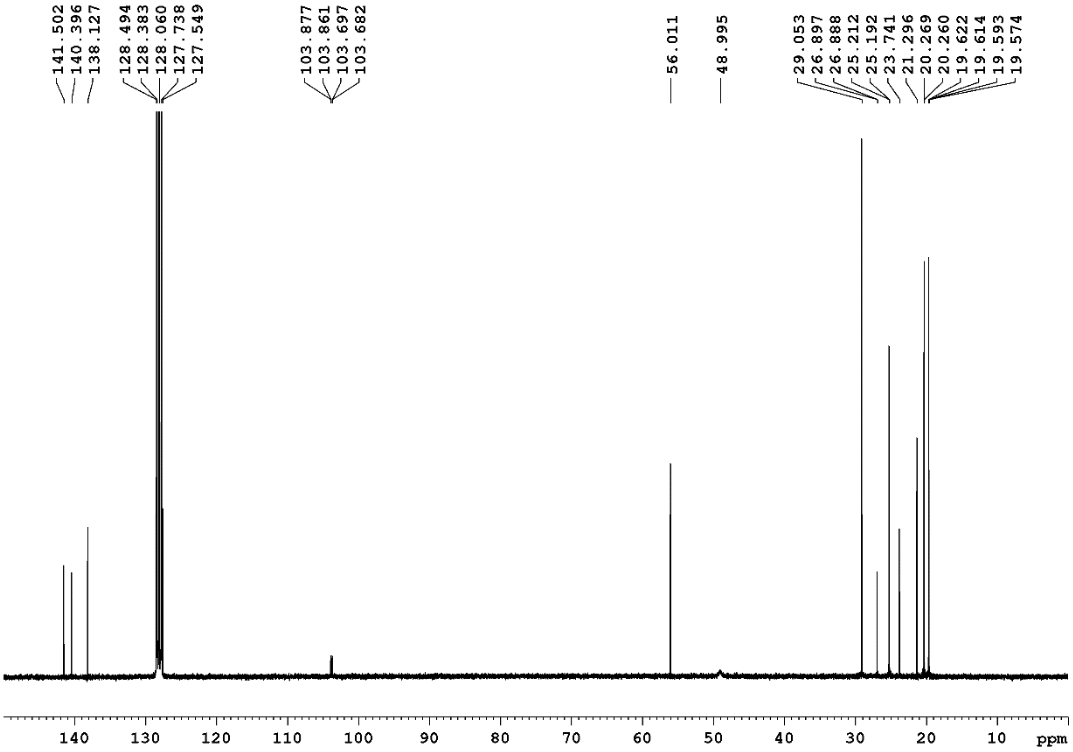

Figure S4. $^{13}C\{^{1}H, ^{31}P\}$ NMR (75.5 MHz, C_6D_6, 298 K) spectrum of **1a**.

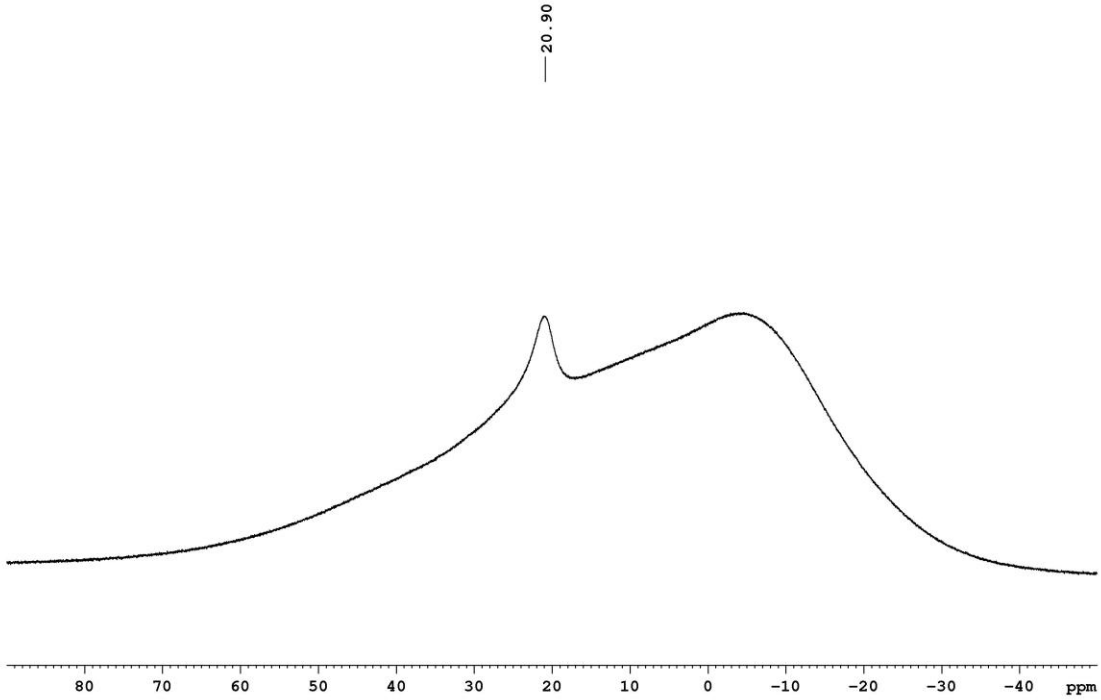

Figure S5. ^{11}B NMR (160.5 MHz, C_6D_6, 298 K) spectrum of **1a**.

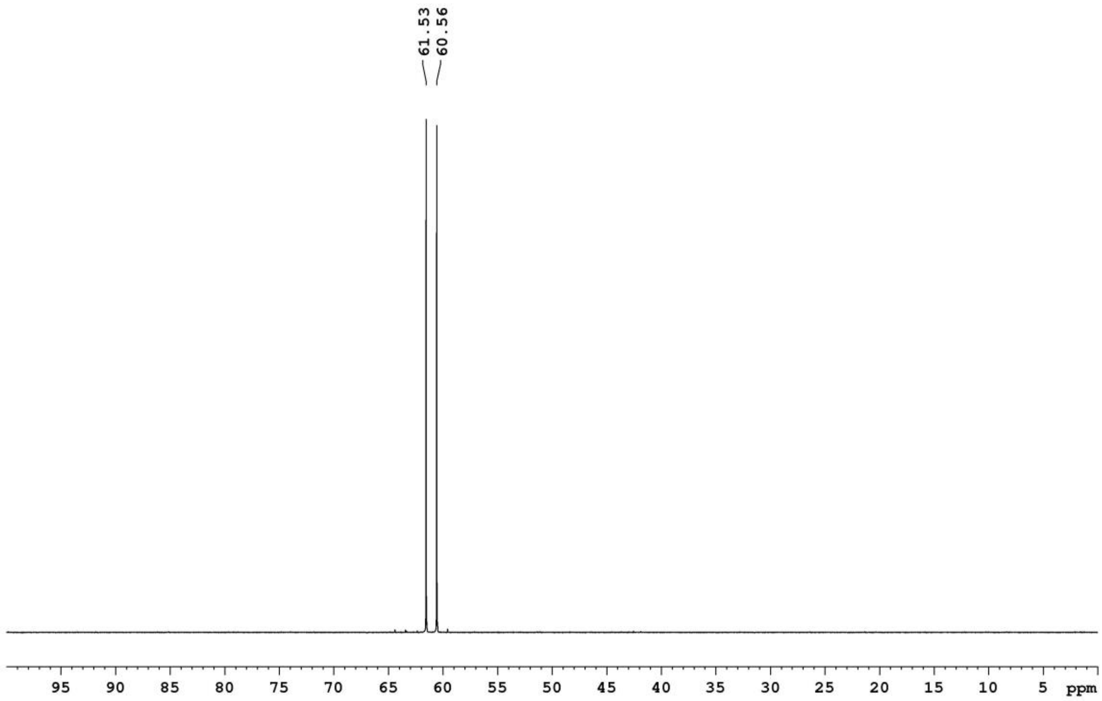

Figure S6. ^{31}P{^{1}H} NMR (202.5 MHz, C_6D_6, 298 K) spectrum of **1a**.

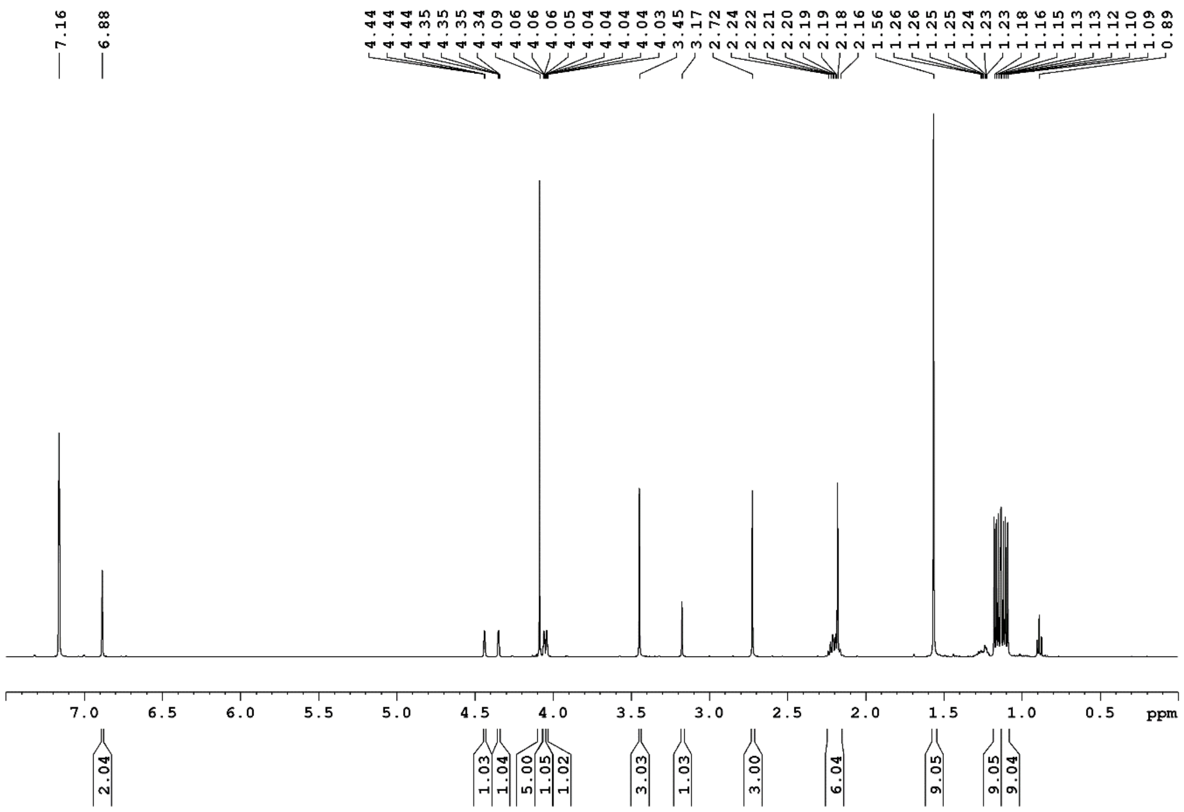

Figure S7. ^{1}H NMR (500.1 MHz, C_6D_6, 298 K) spectrum of **1c**.

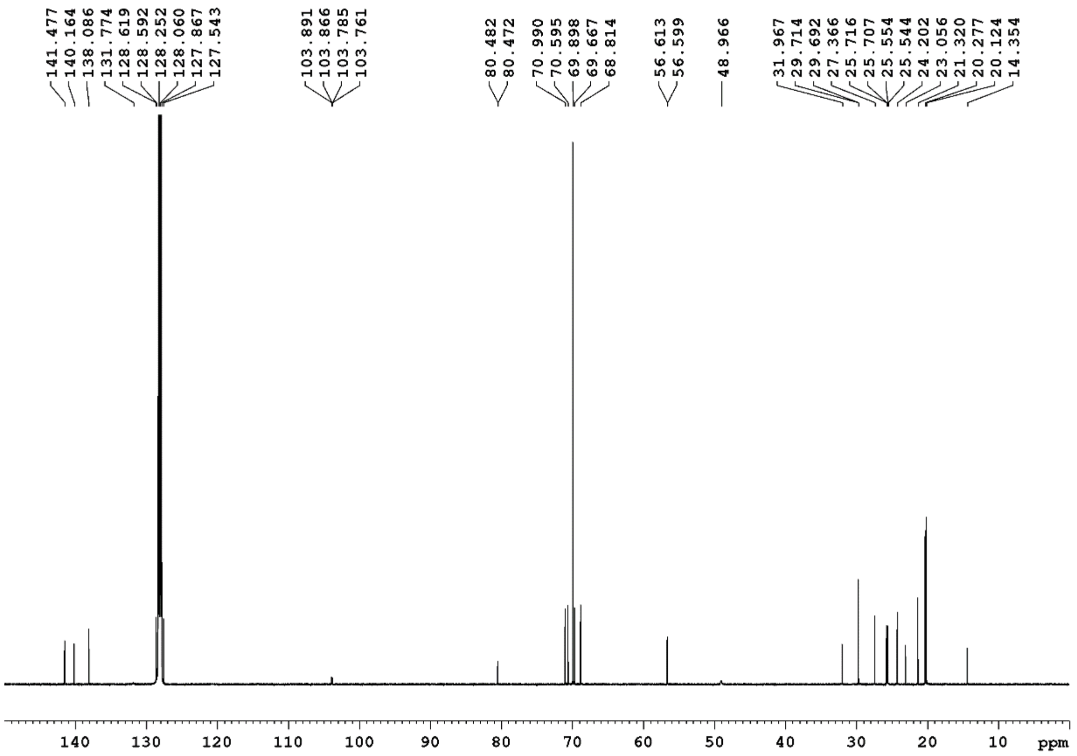

Figure S8. $^{13}C\{^{1}H\}$ NMR (125.8 MHz, C_6D_6, 298 K) spectrum of **1c**.

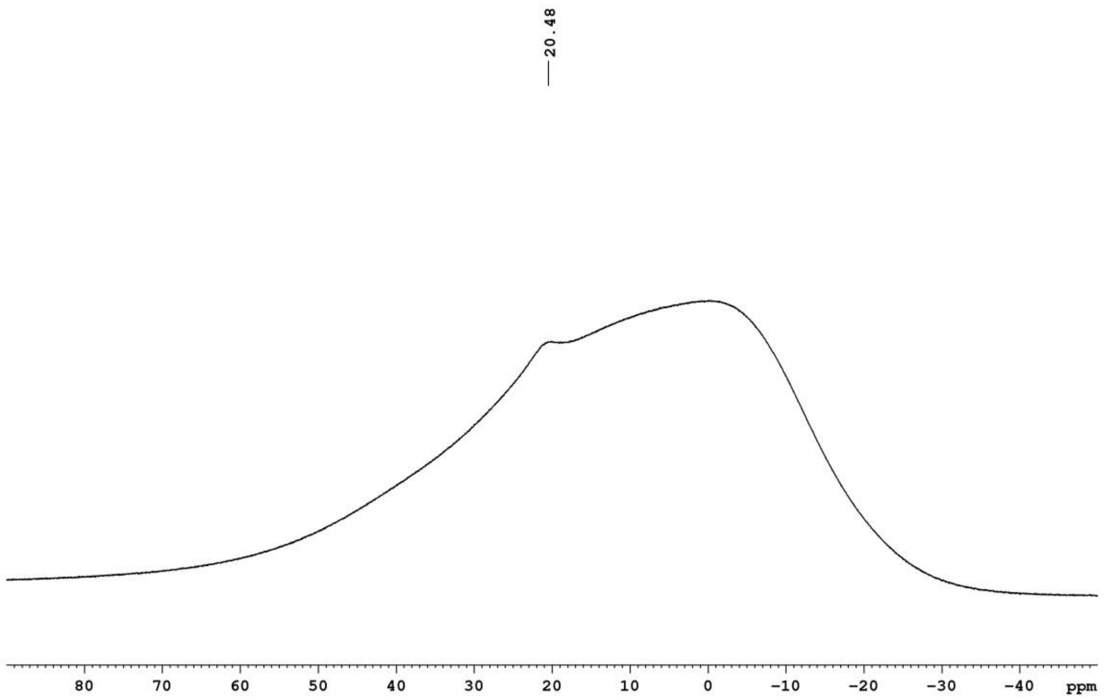

Figure S9. ^{11}B NMR (160.5 MHz, C_6D_6, 298 K) spectrum of **1c**.

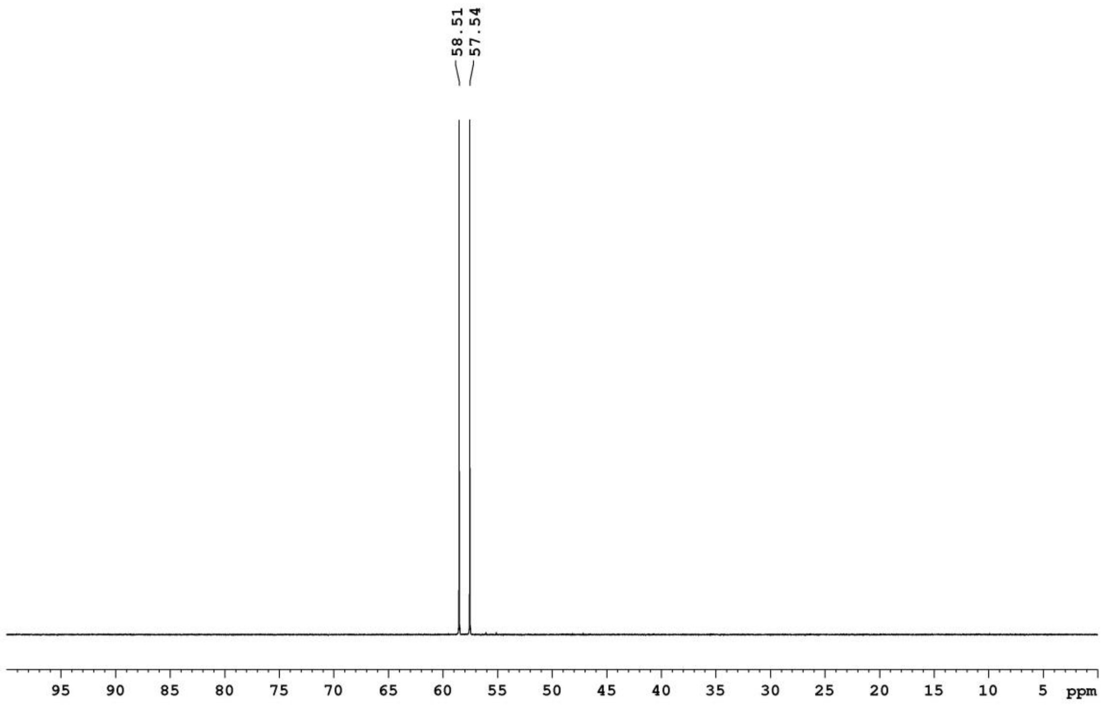

Figure S10. $^{31}P\{^1H\}$ NMR (202.5 MHz, C_6D_6, 298 K) spectrum of **1c**.

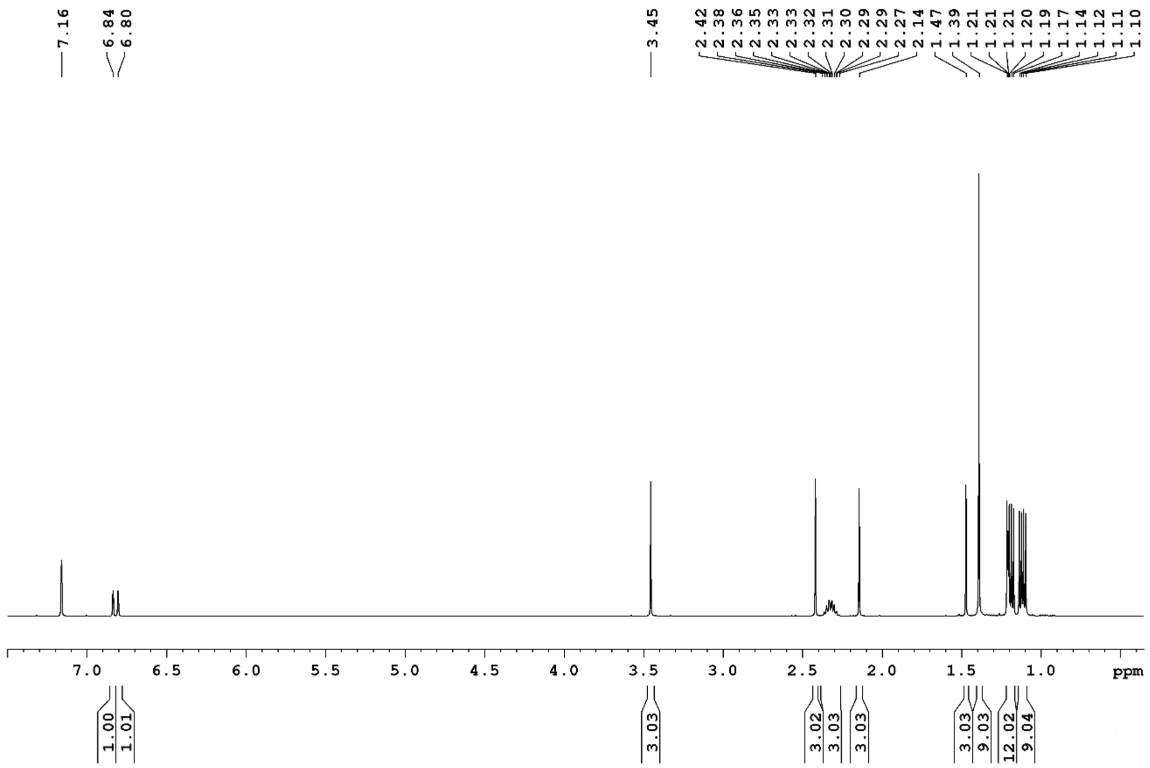

Figure S11. ^{1}H NMR (500.1 MHz, C_6D_6, 298 K) spectrum of **1d**.

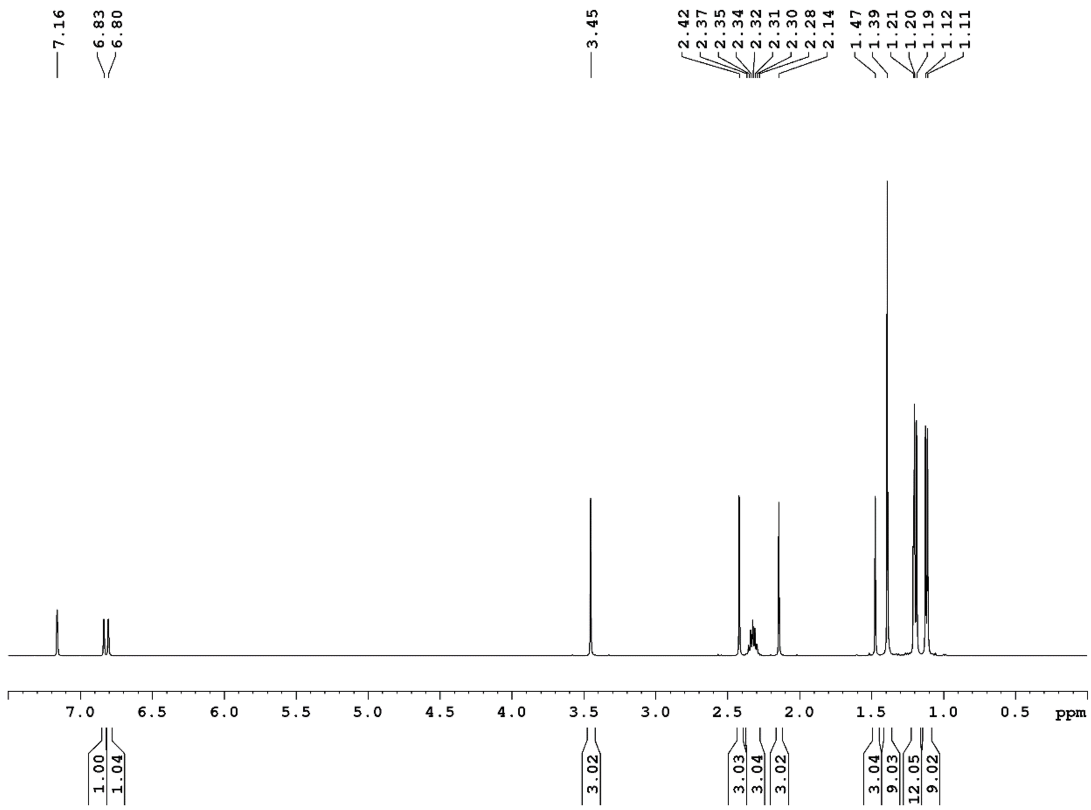

Figure S12. ^{1}H{^{31}P} NMR (500.1 MHz, C_6D_6, 298 K) spectrum of **1d**.

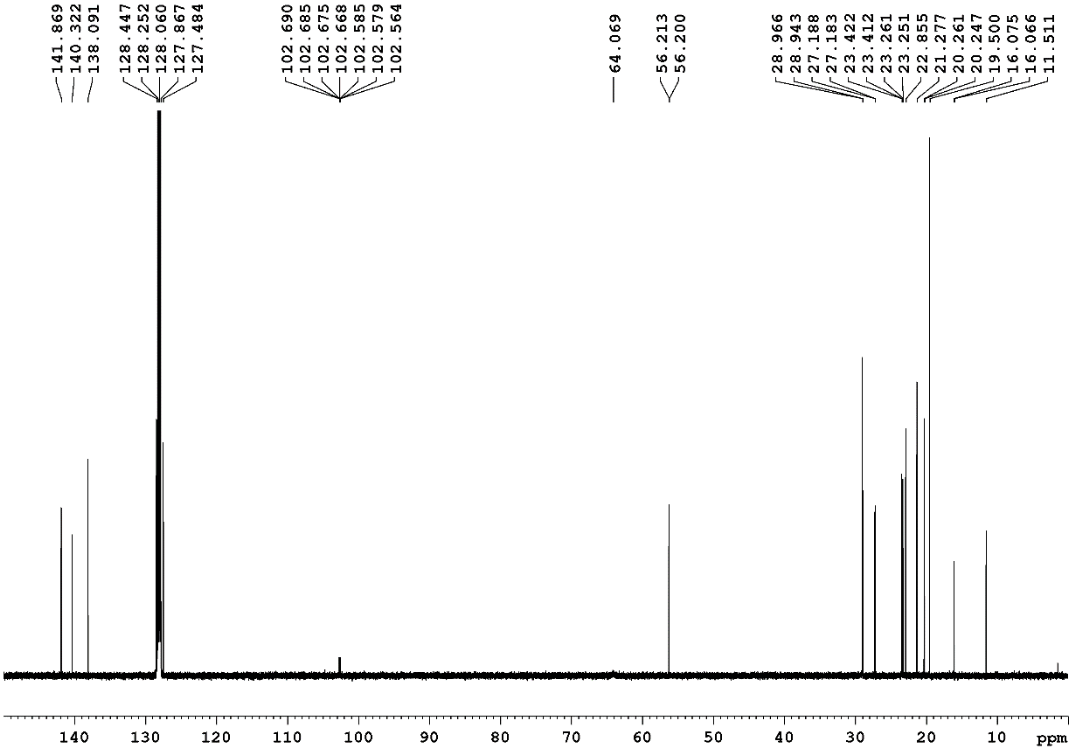

Figure S13. $^{13}C\{^{1}H\}$ NMR (125.8 MHz, C_6D_6, 298 K) spectrum of **1d**.

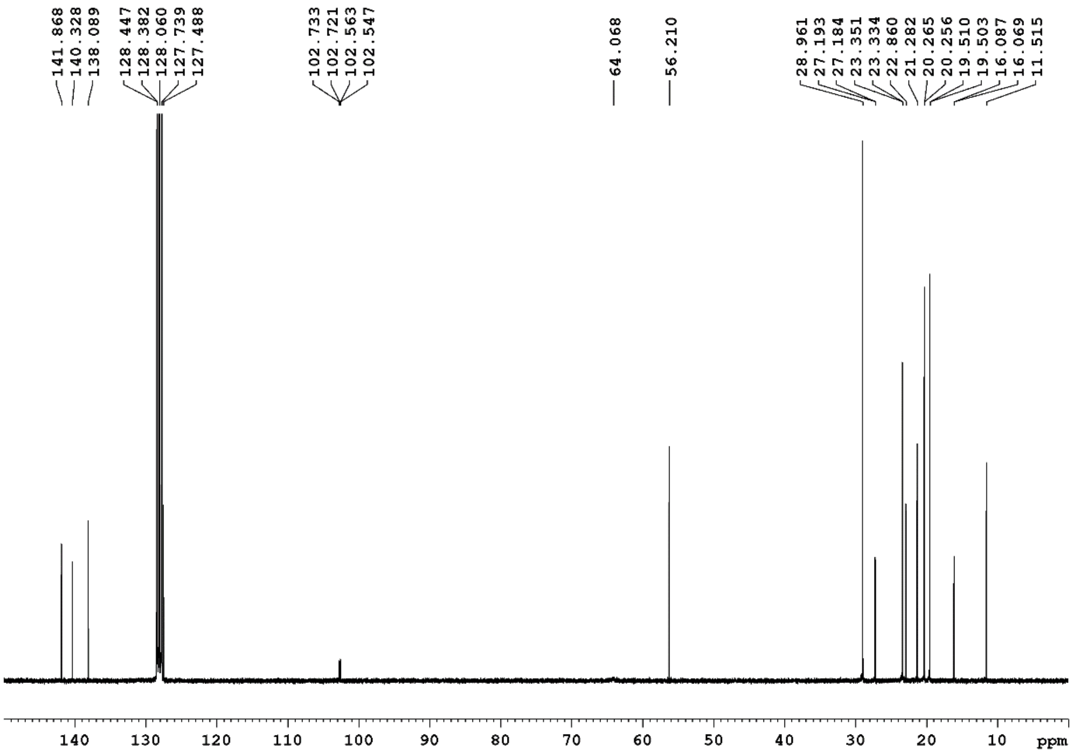

Figure S14. $^{13}C\{^{1}H, ^{31}P\}$ NMR (75.5 MHz, C_6D_6, 298 K) spectrum of **1d**.

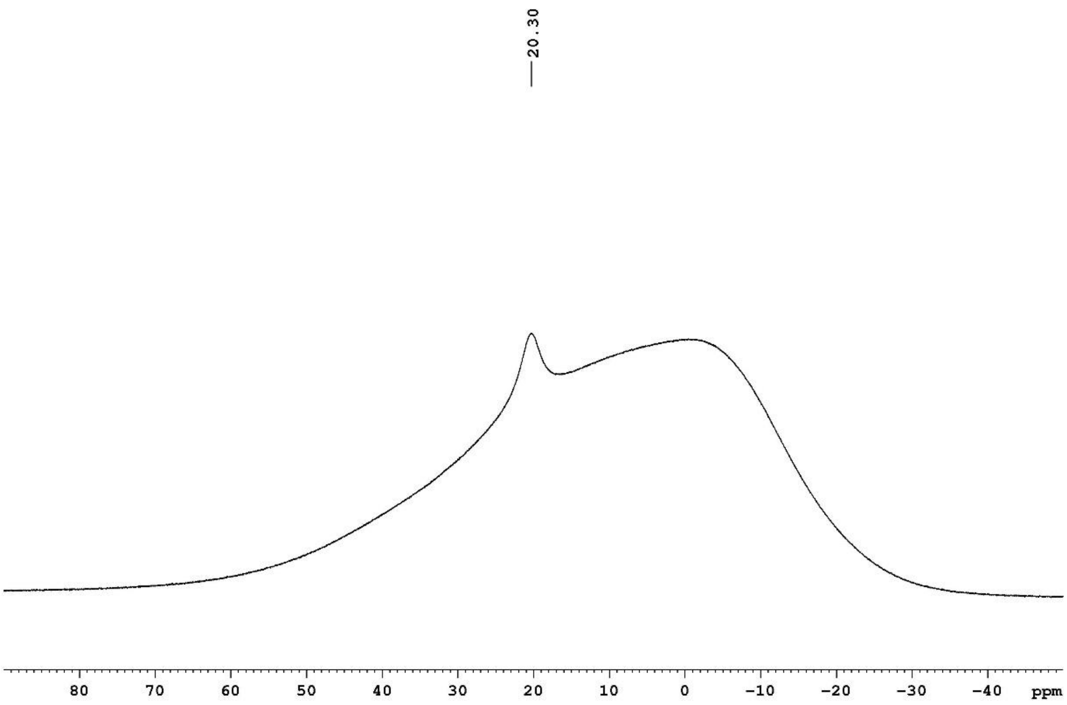

Figure S15. ^{11}B NMR (160.5 MHz, C_6D_6, 298 K) spectrum of **1d**.

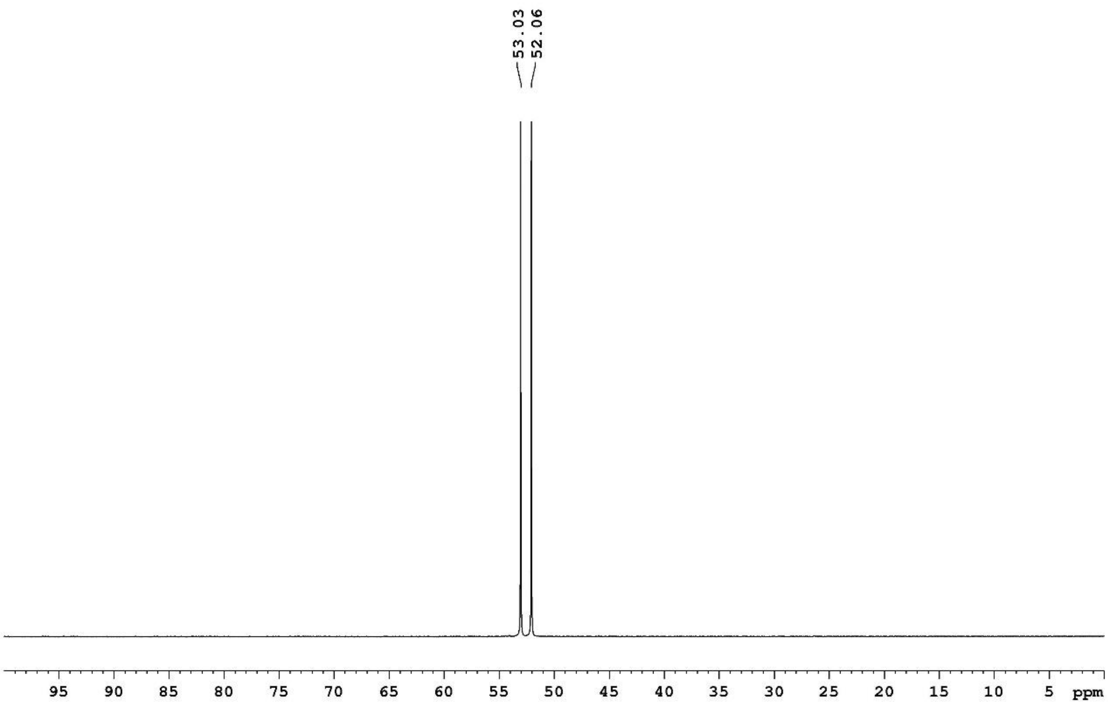

Figure S16. $^{31}P\{^1H\}$ NMR (202.5 MHz, C_6D_6, 298 K) spectrum of **1d**.

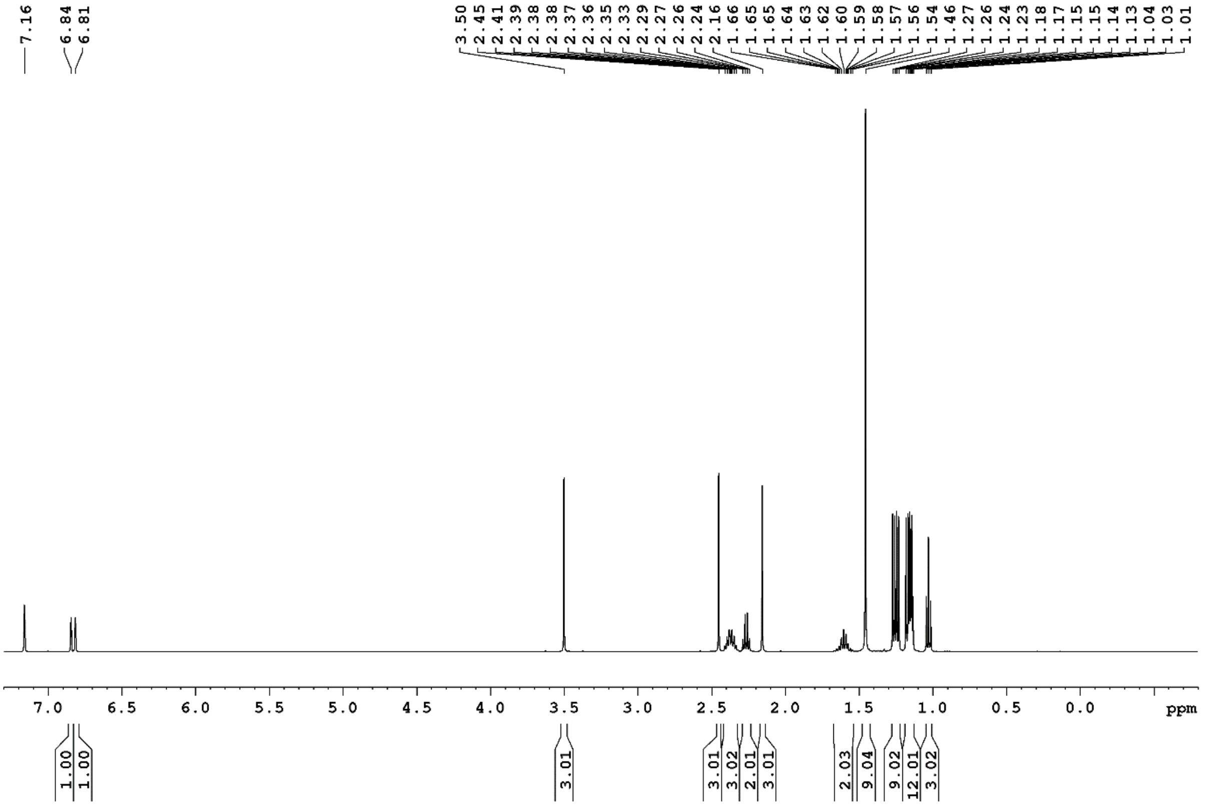

Figure S17. ^{1}H NMR (500.1 MHz, C_6D_6, 298 K) spectrum of **1e**.

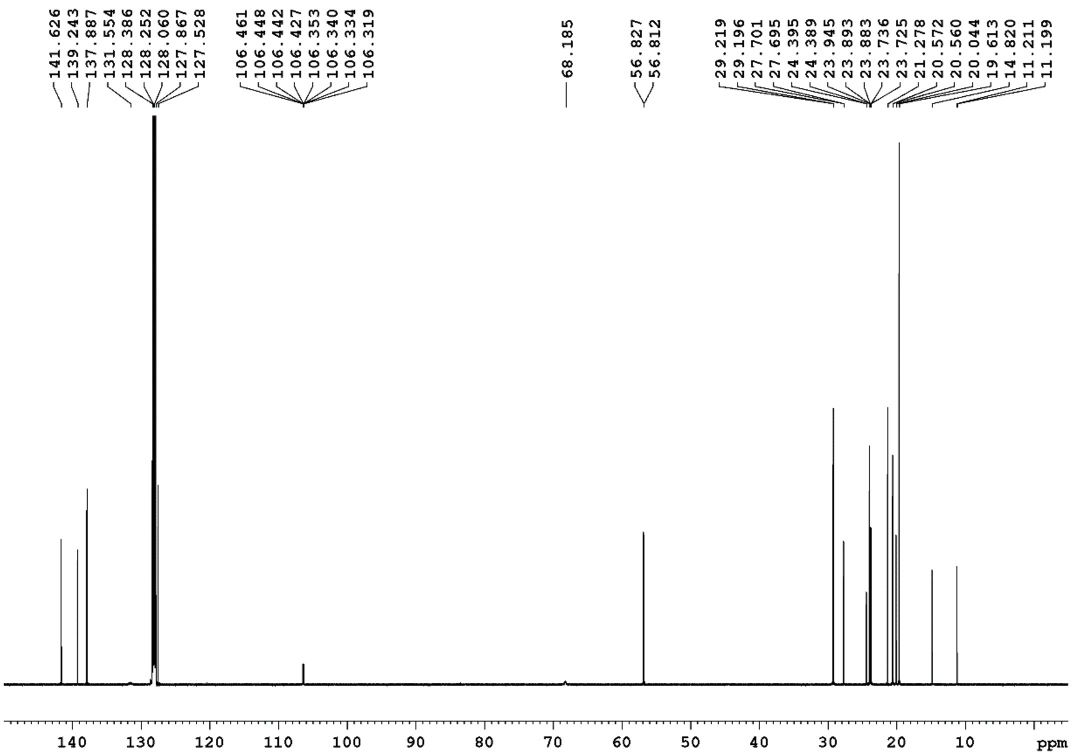

Figure S18. $^{13}C\{^{1}H\}$ NMR (125.8 MHz, C_6D_6, 298 K) spectrum of **1e**.

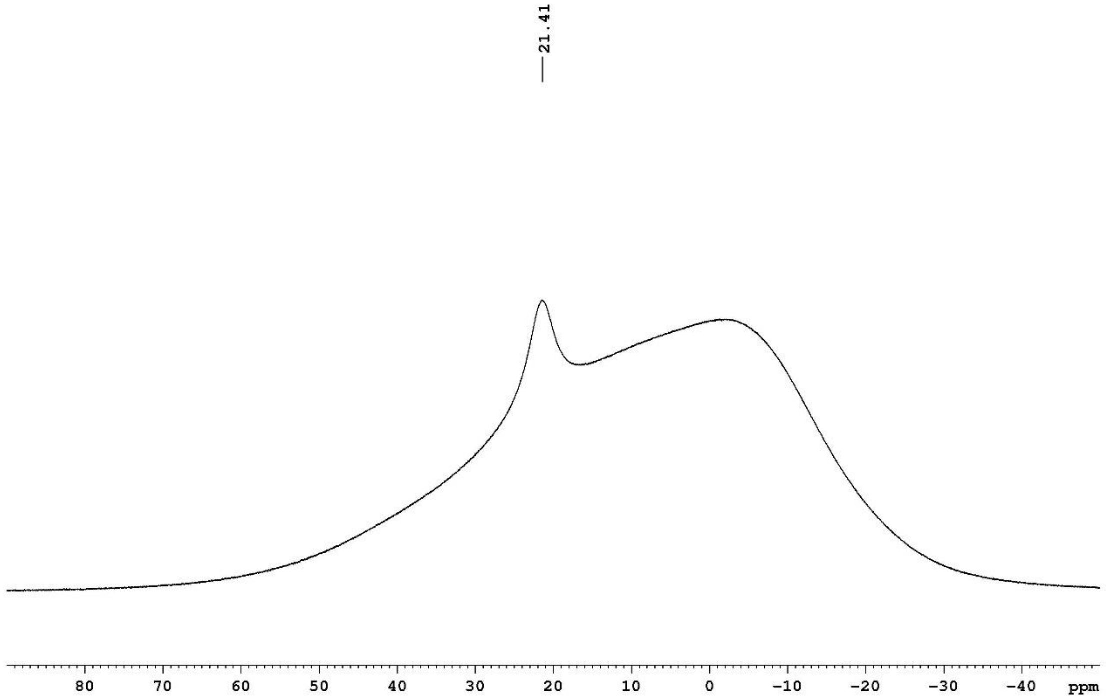

Figure S19. ^{11}B NMR (160.5 MHz, C_6D_6, 298 K) spectrum of **1e**.

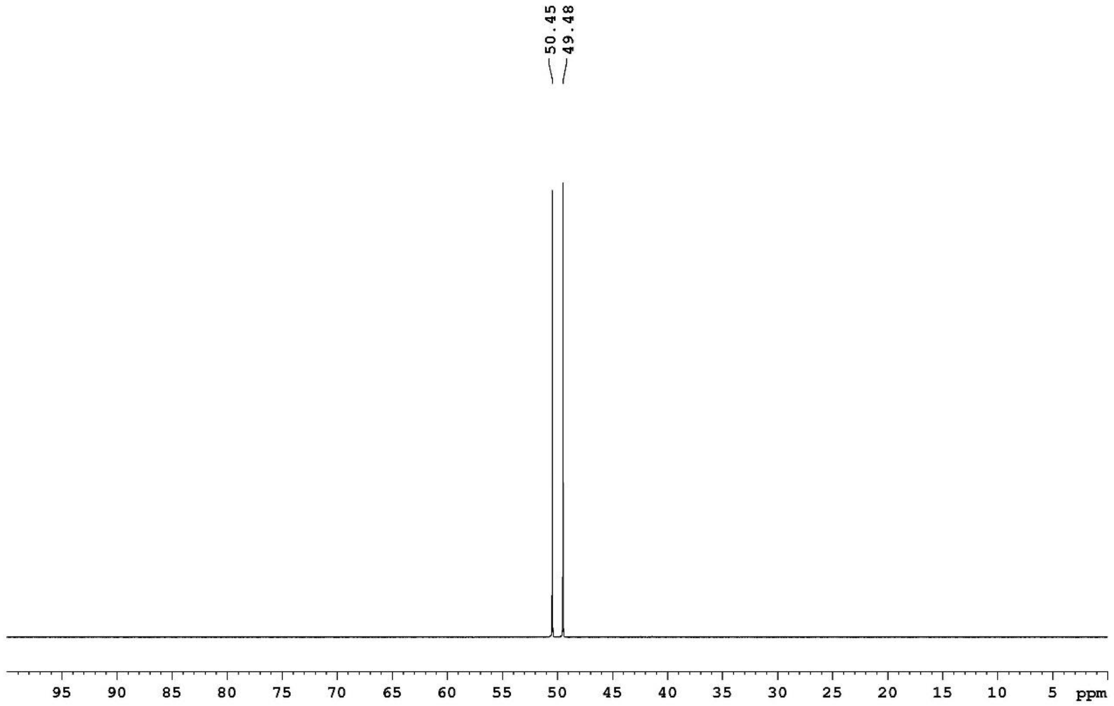

Figure S20. $^{31}P\{^1H\}$ NMR (202.5 MHz, C_6D_6, 298 K) spectrum of **1e**.

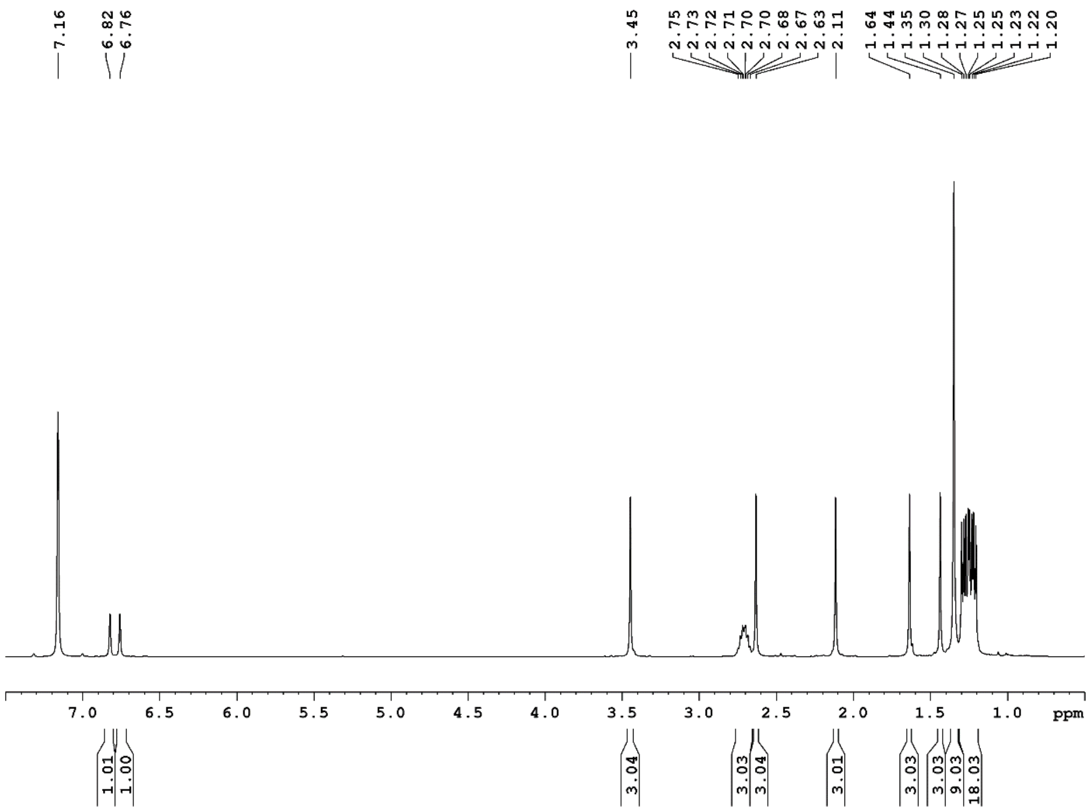

Figure S21. ^{1}H NMR (500.1 MHz, C_6D_6, 298 K) spectrum of **1f**.

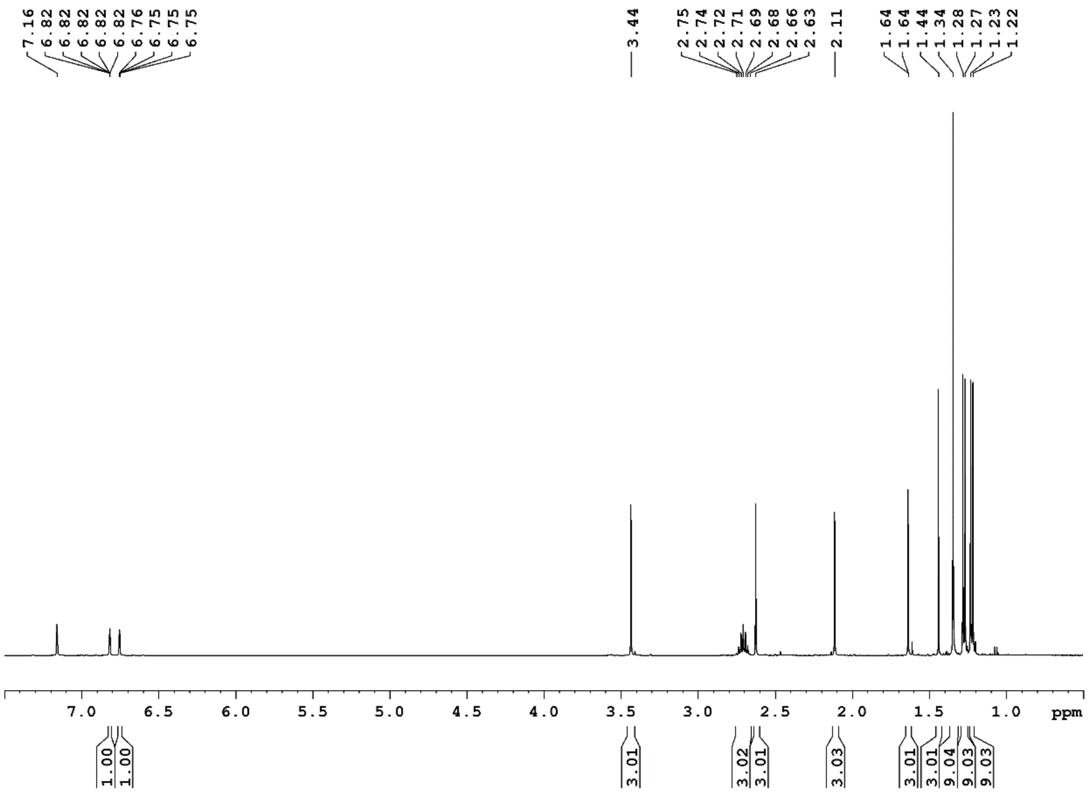

Figure S22. ^{1}H{^{31}P} NMR (500.1 MHz, C_6D_6, 298 K) spectrum of **1f**.

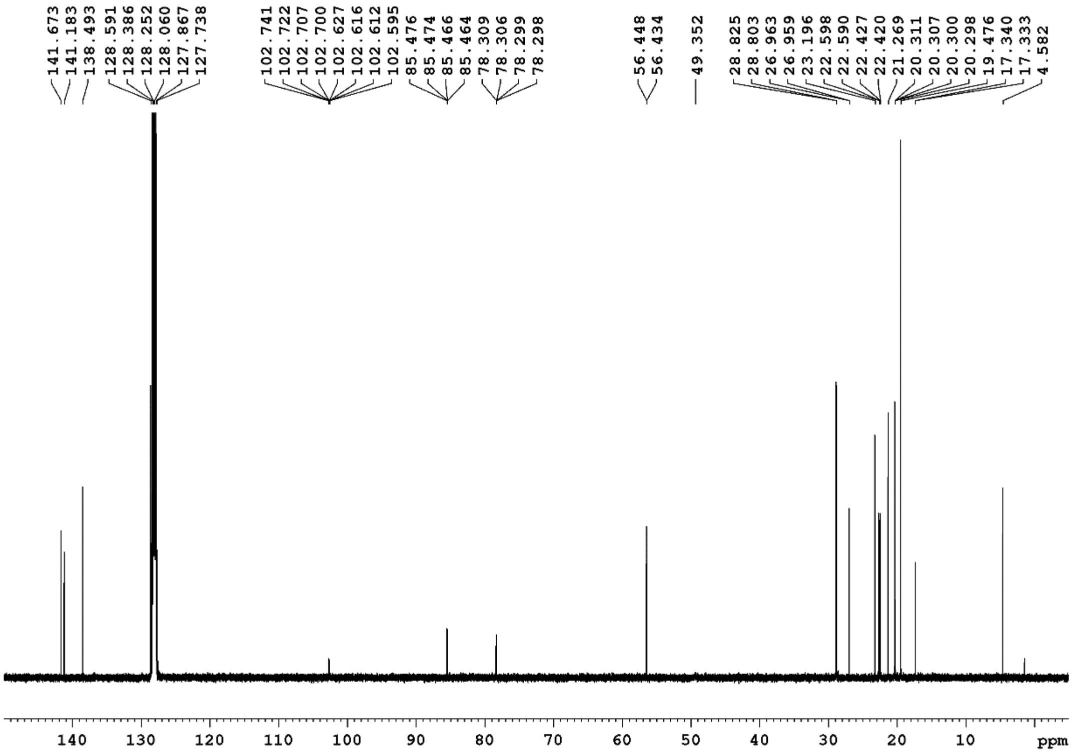

Figure S23. $^{13}C\{^1H\}$ NMR (125.8 MHz, C_6D_6, 298 K) spectrum of **1f**.

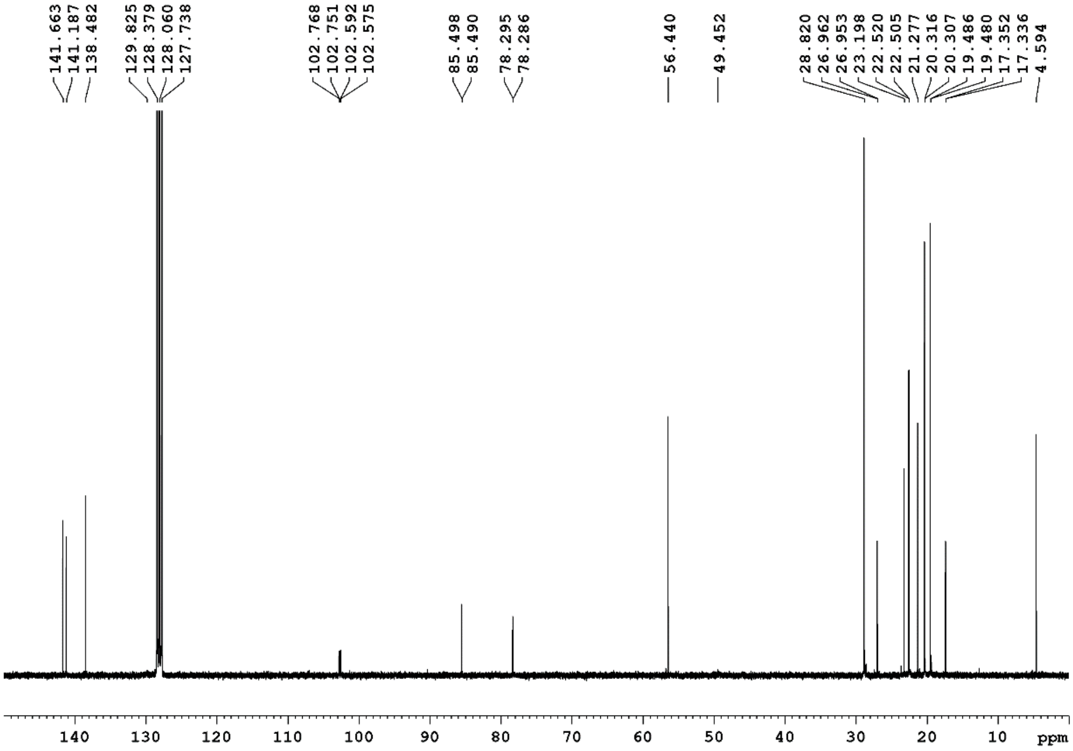

Figure S24. $^{13}C\{^1H, ^{31}P\}$ NMR (75.5 MHz, C_6D_6, 298 K) spectrum of **1f**.

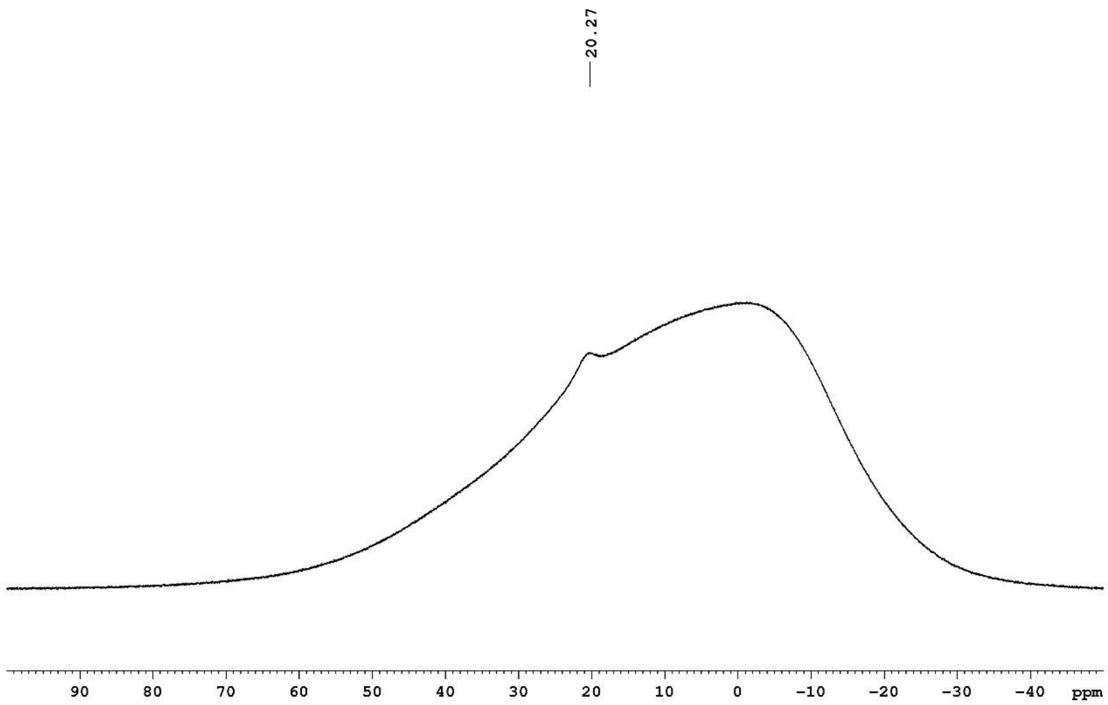

Figure S25. ^{11}B NMR (160.5 MHz, C_6D_6, 298 K) spectrum of **1f**.

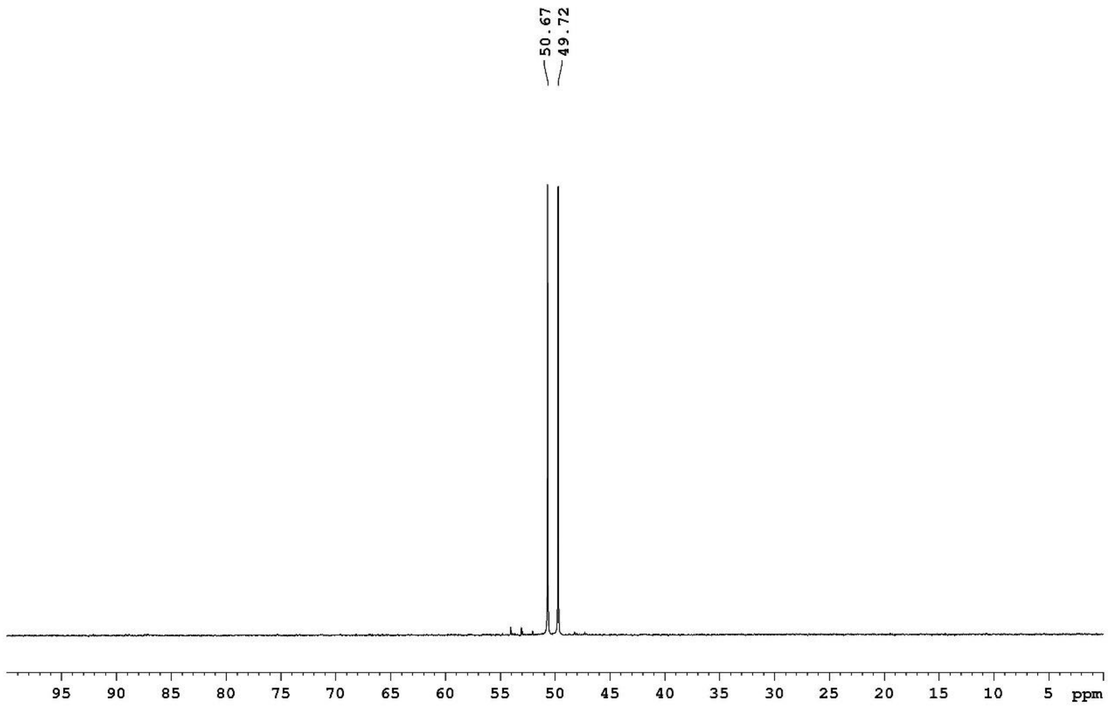

Figure S26. $^{31}P\{^1H\}$ NMR (202.5 MHz, C_6D_6, 298 K) spectrum of **1f**.

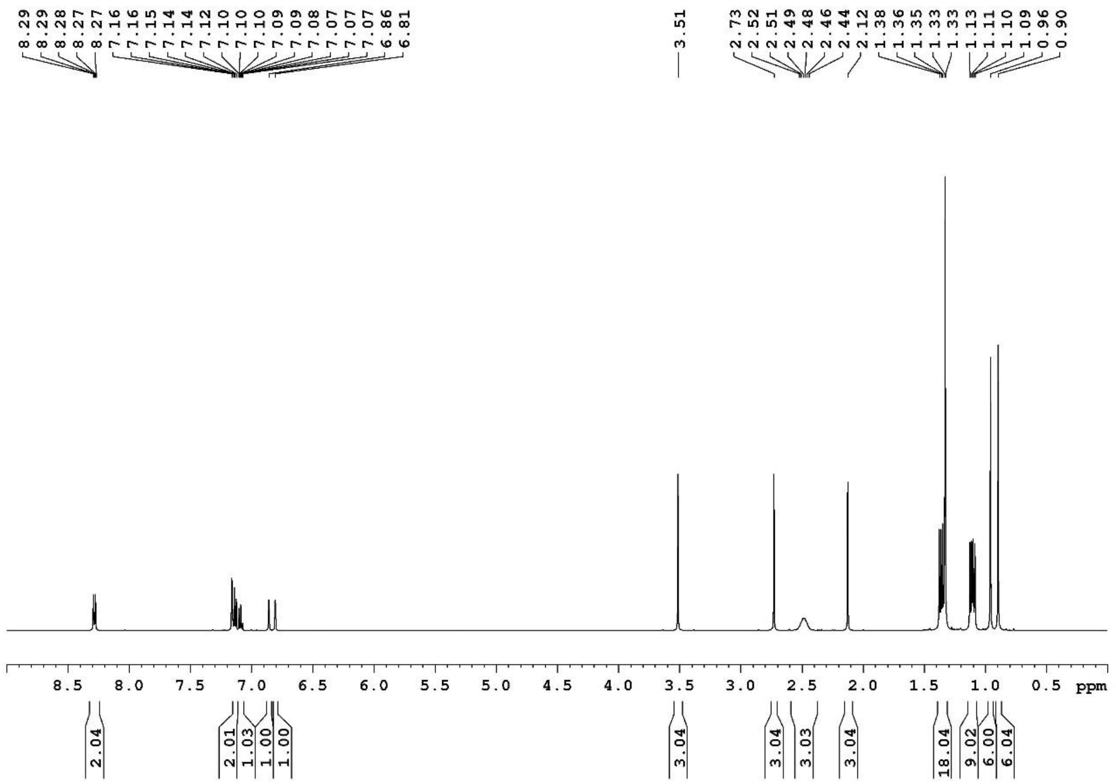

Figure S27. ^{1}H NMR (500.1 MHz, C_6D_6, 298 K) spectrum of **1g**.

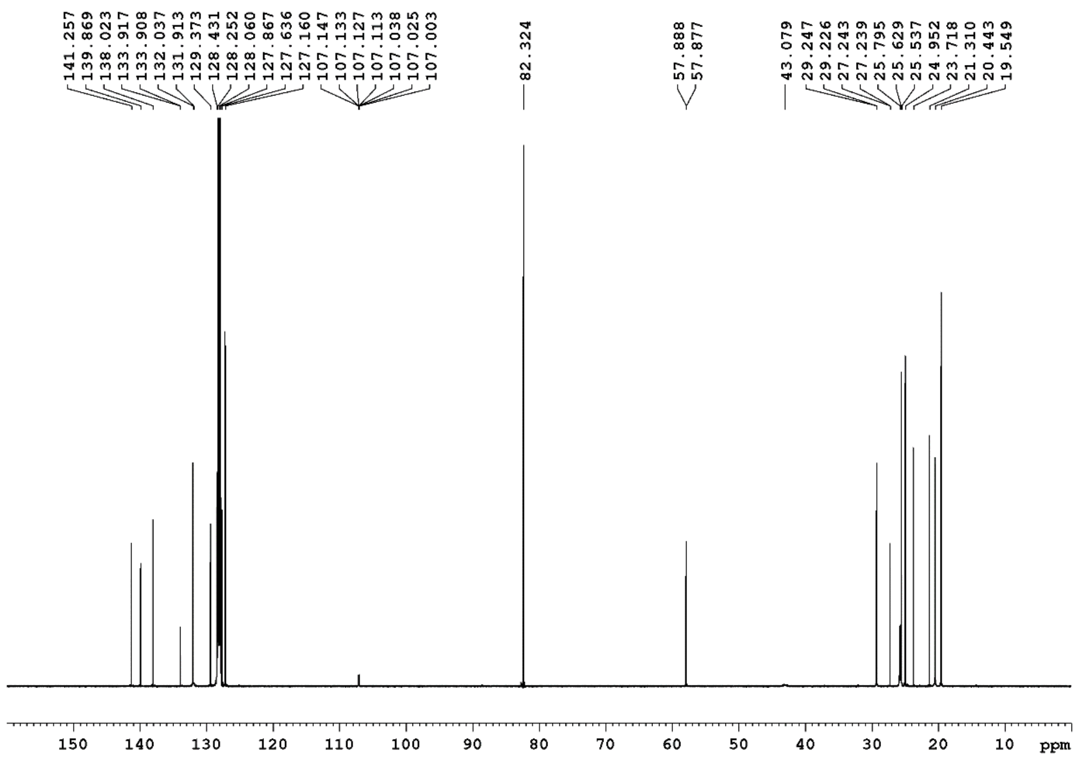

Figure S28. $^{13}C\{^{1}H\}$ NMR (125.8 MHz, C_6D_6, 298 K) spectrum of **1g**.

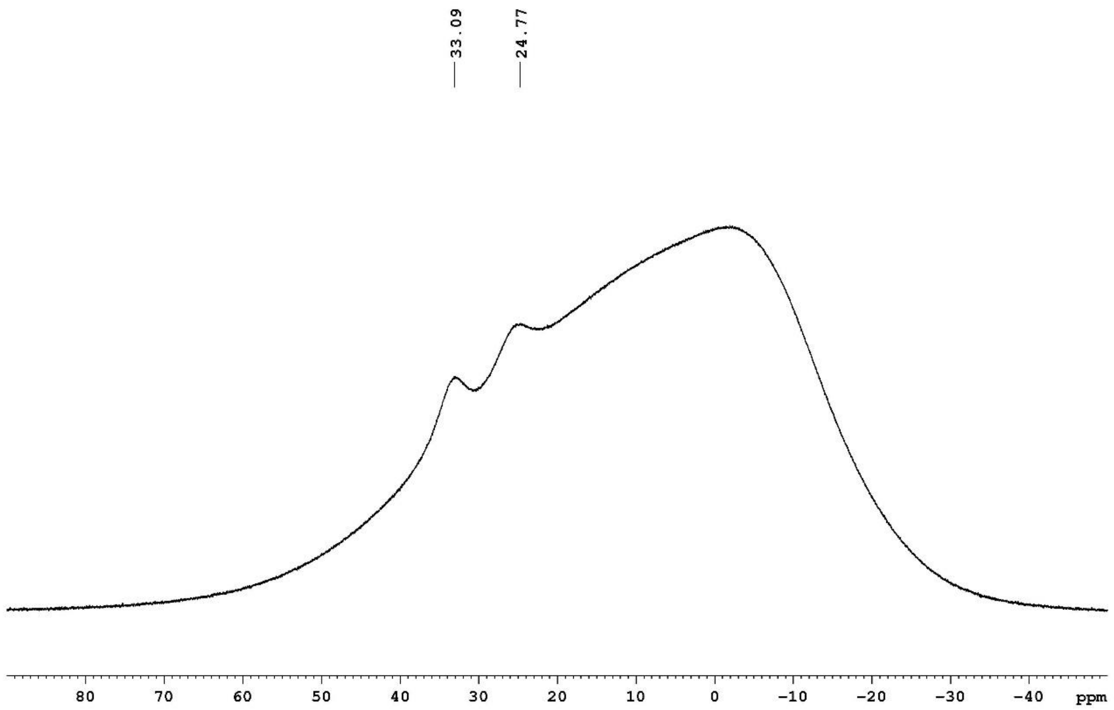

Figure S29. ^{11}B NMR (160.5 MHz, C_6D_6, 298 K) spectrum of **1g**.

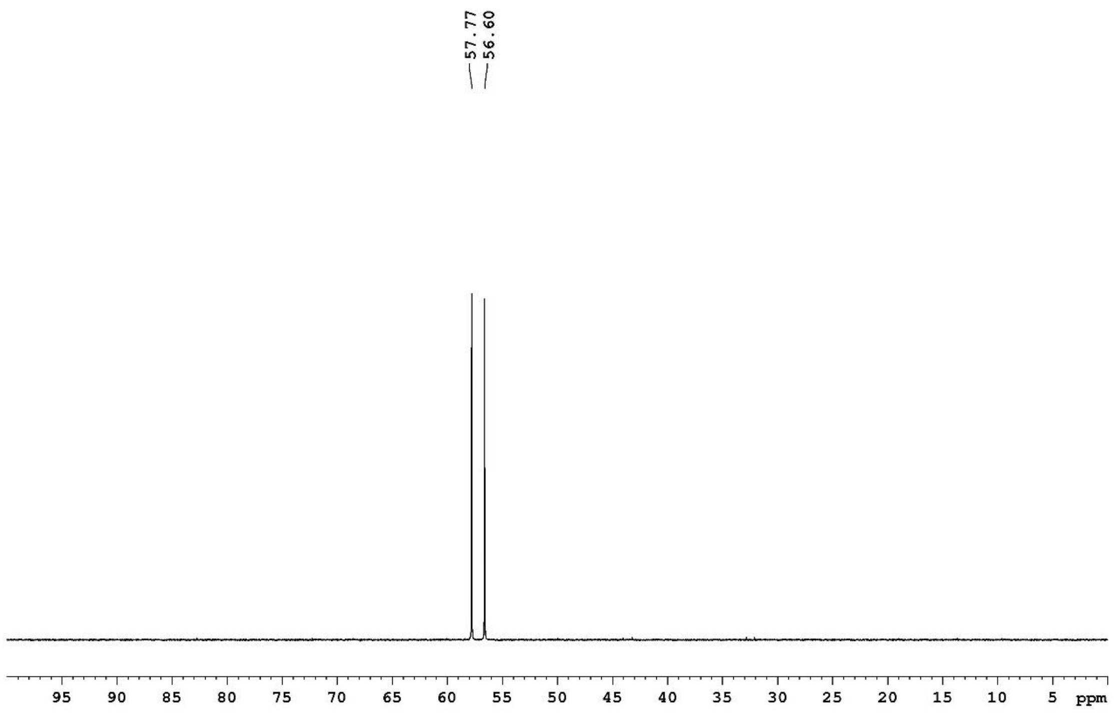

Figure S30. $^{31}P\{^1H\}$ NMR (162.2 MHz, C_6D_6, 298 K) spectrum of **1g**.

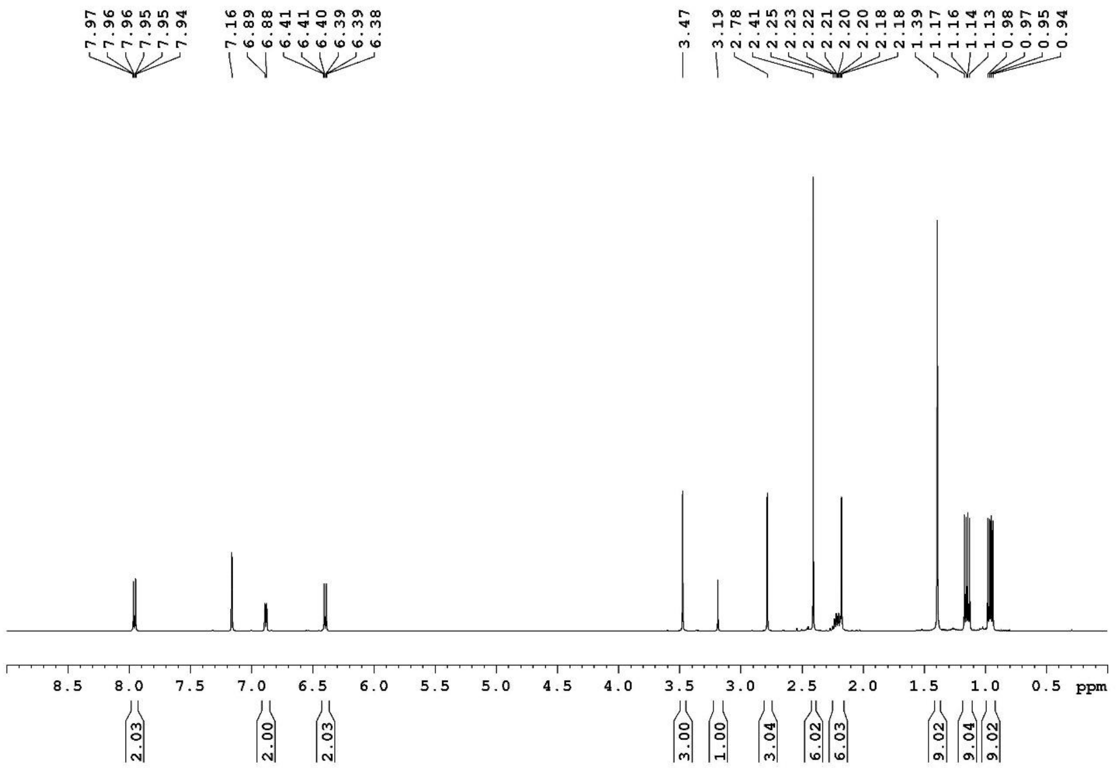

Figure S31. ^{1}H NMR (500.1 MHz, C_6D_6, 298 K) spectrum of **1h**.

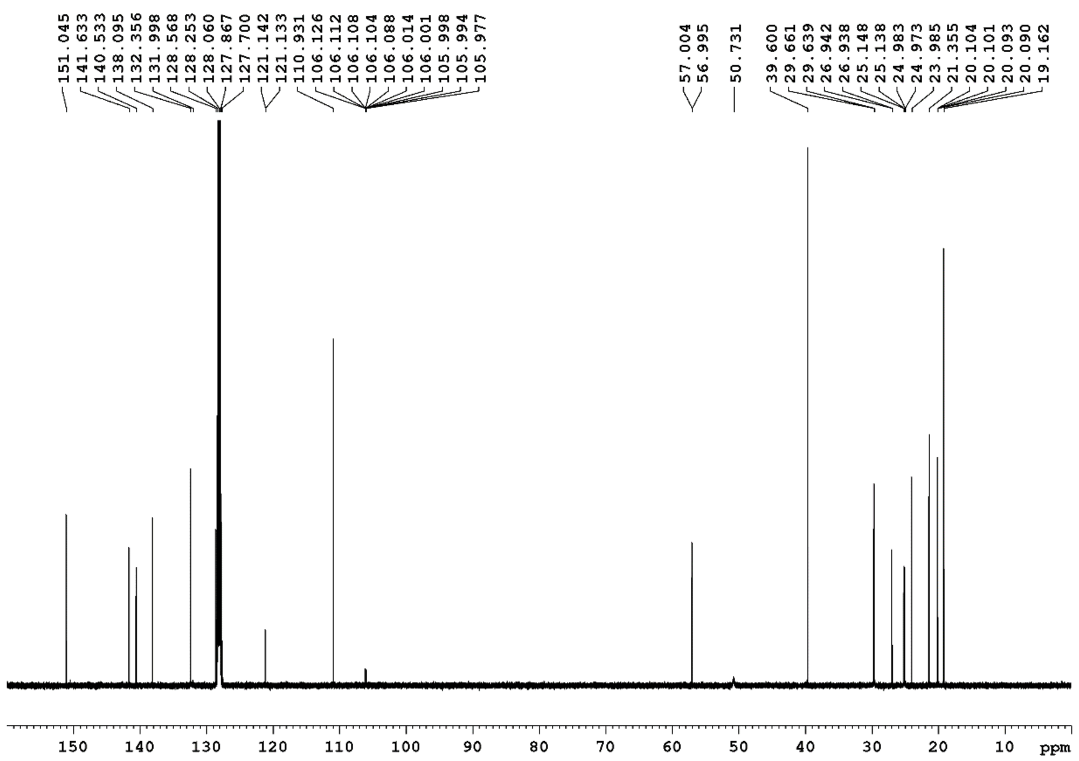

Figure S32. $^{13}C\{^{1}H\}$ NMR (125.8 MHz, C_6D_6, 298 K) spectrum of **1h**.

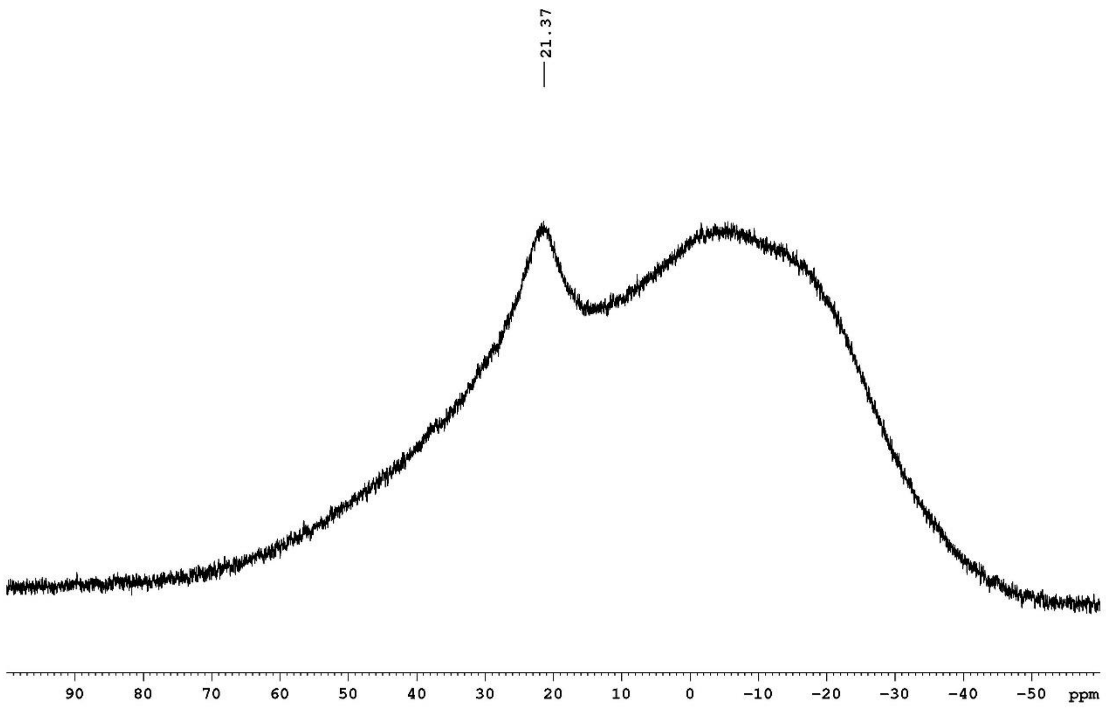

Figure S33. ^{11}B NMR (128.5 MHz, C_6D_6, 298 K) spectrum of **1h**.

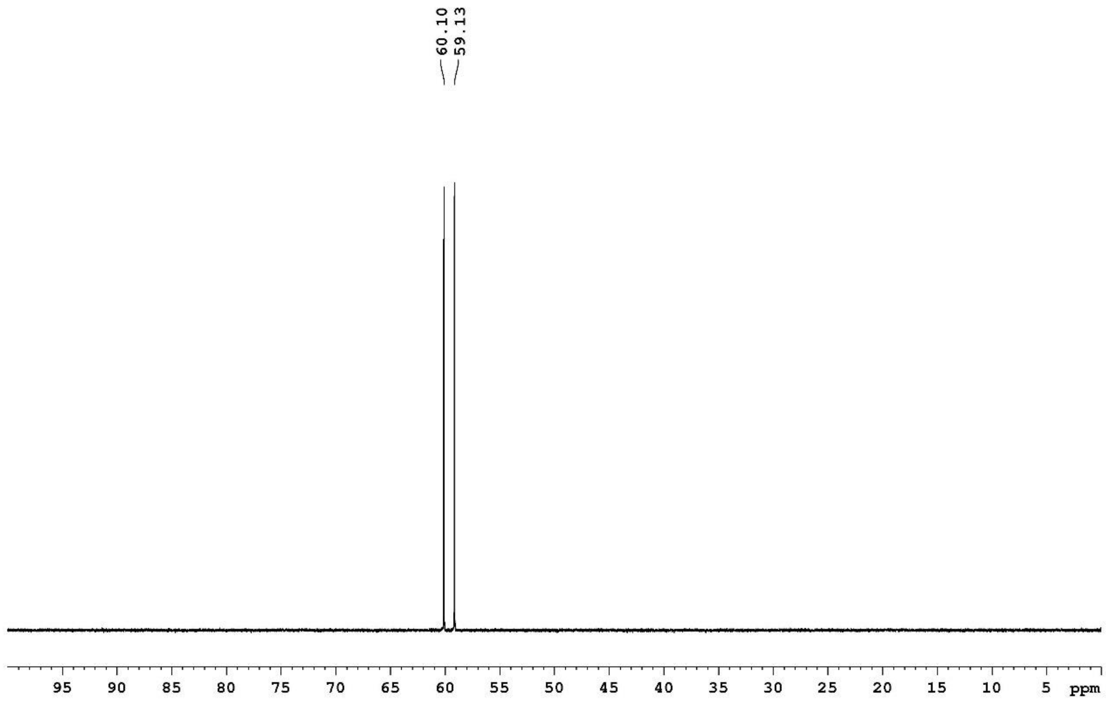

Figure S34 $^{31}P\{^1H\}$ NMR (202.5 MHz, C_6D_6, 298 K) spectrum of **1h**.

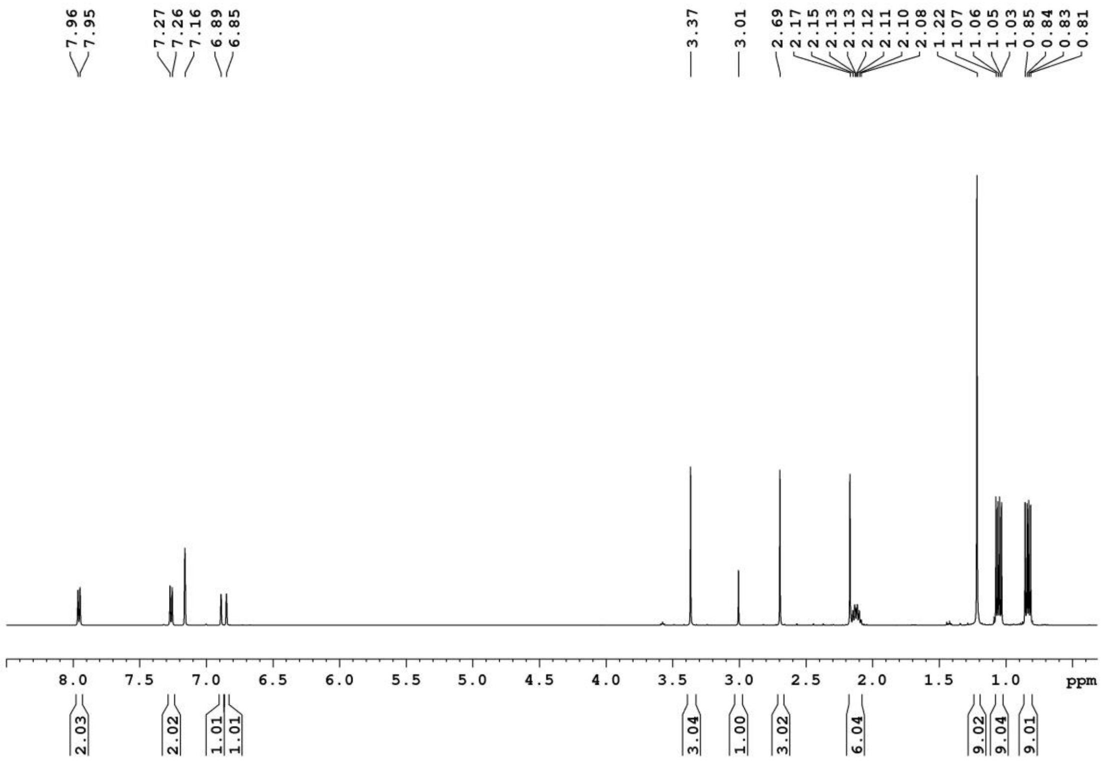

Figure S35. ^{1}H NMR (500.1 MHz, C_6D_6, 298 K) spectrum of **1i**.

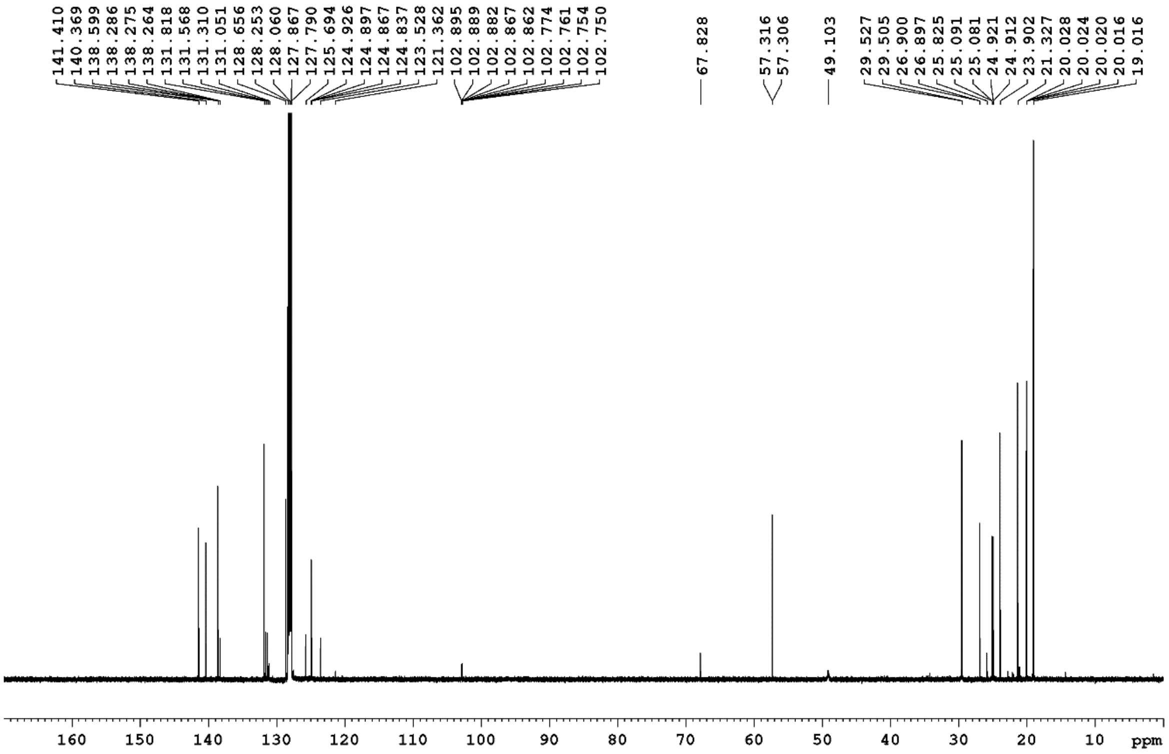

Figure S36. $^{13}C\{^{1}H\}$ NMR (125.8 MHz, C_6D_6, 298 K) spectrum of **1i**.

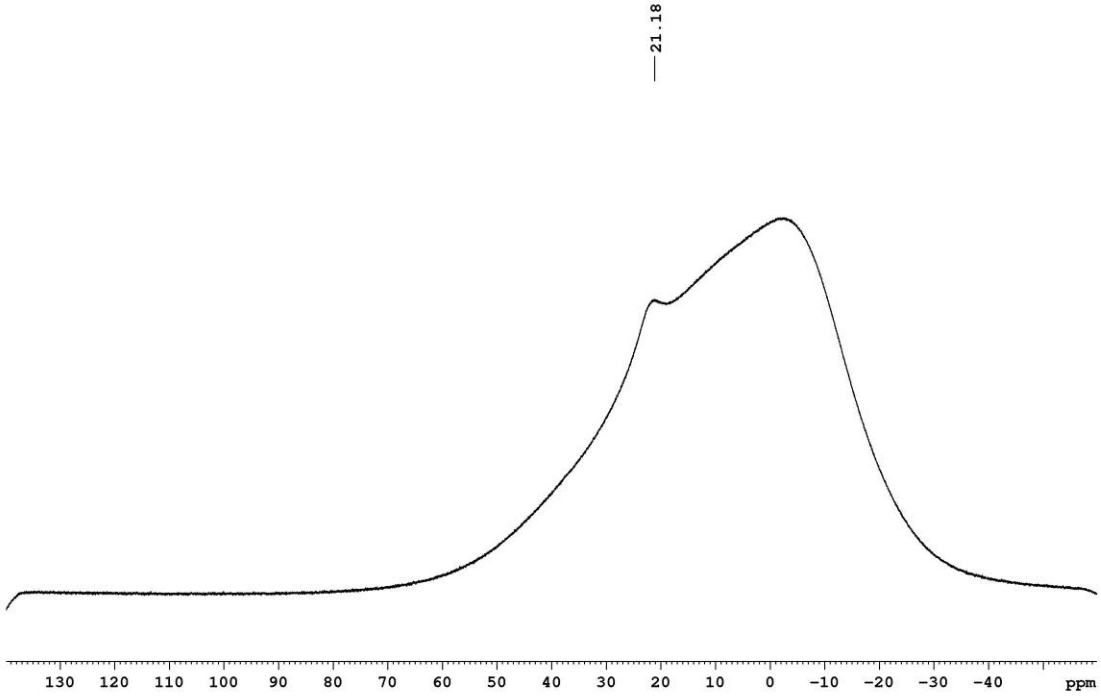

Figure S37. ^{11}B NMR (160.5 MHz, C_6D_6, 298 K) spectrum of **1i**.

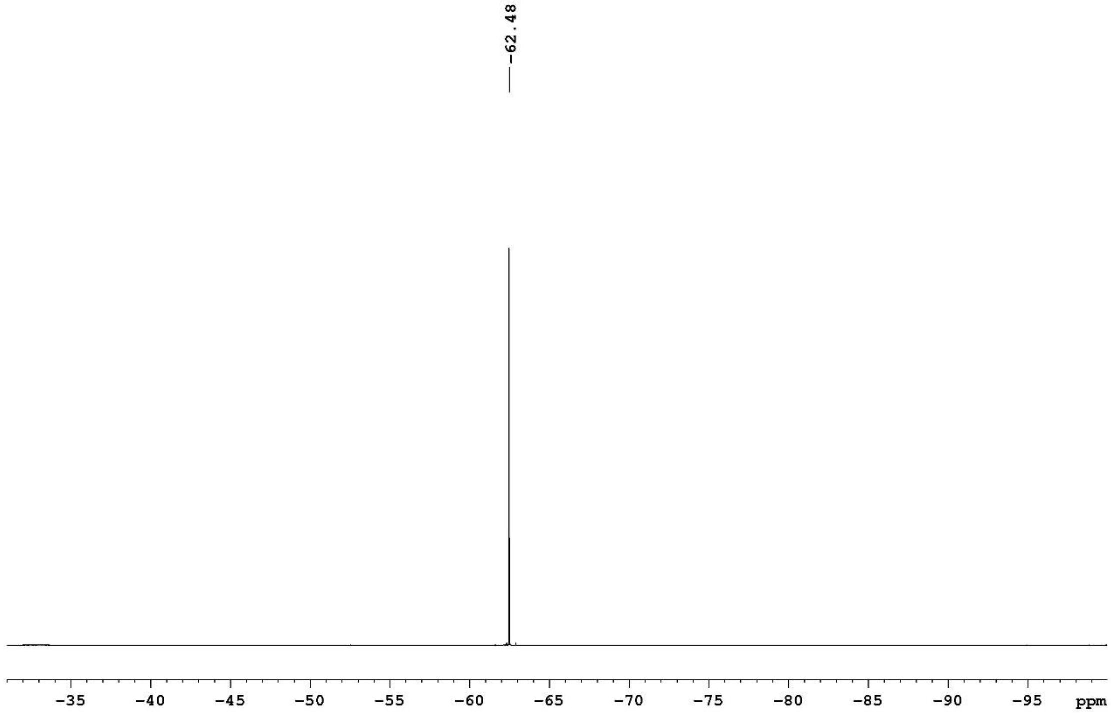

Figure S38. ^{19}F NMR (470.6 MHz, C_6D_6, 298 K) spectrum of **1i**.

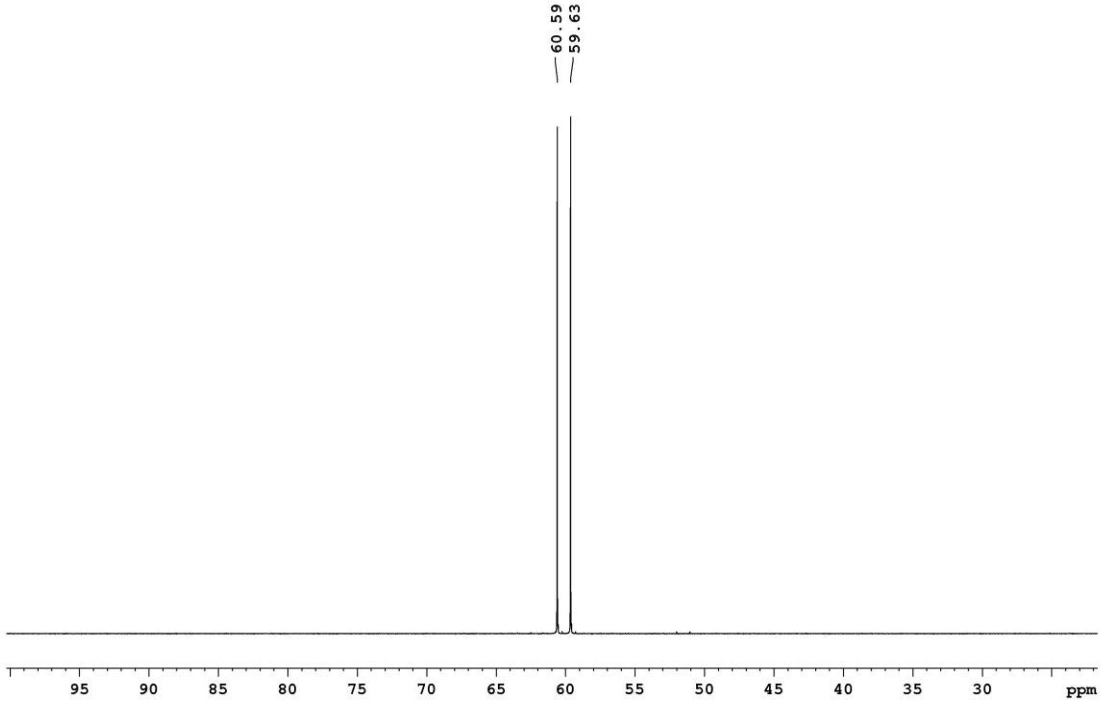

Figure S39. $^{31}P\{^{1}H\}$ NMR (202.5 MHz, C_6D_6, 298 K) spectrum of **1i**.

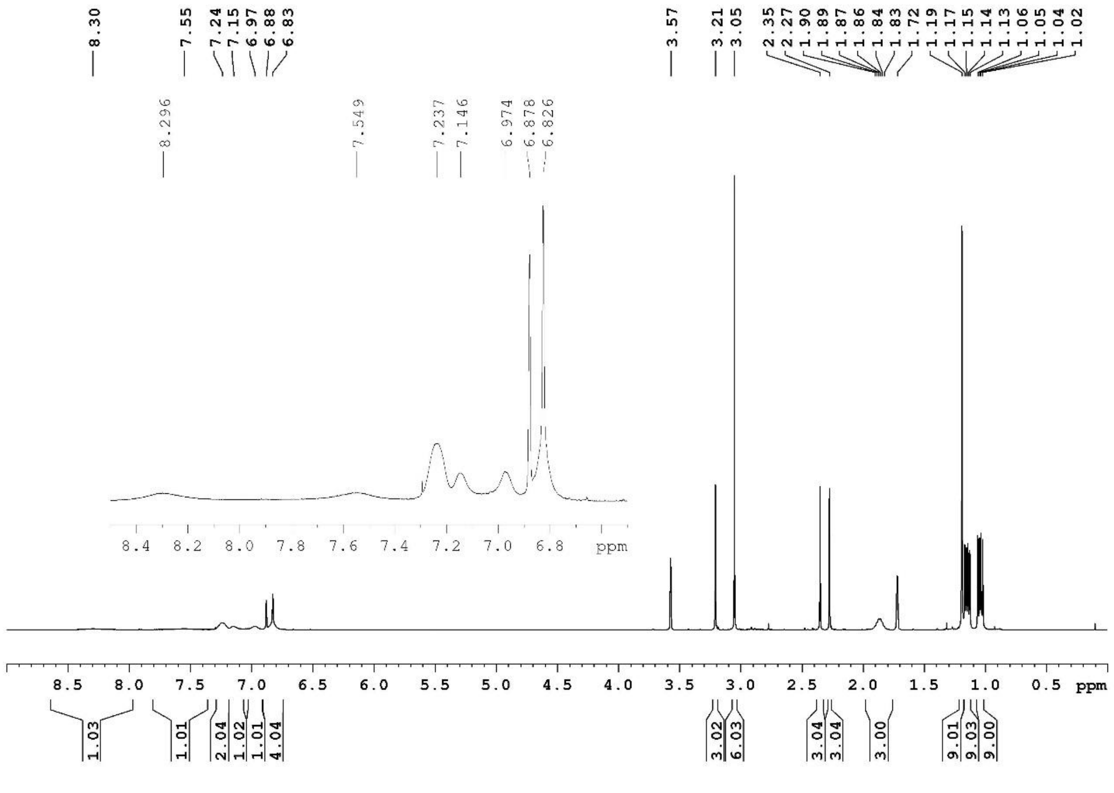

Figure S40. ^{1}H NMR (500.1 MHz, d_8-THF, 298 K) spectrum of **1j**.

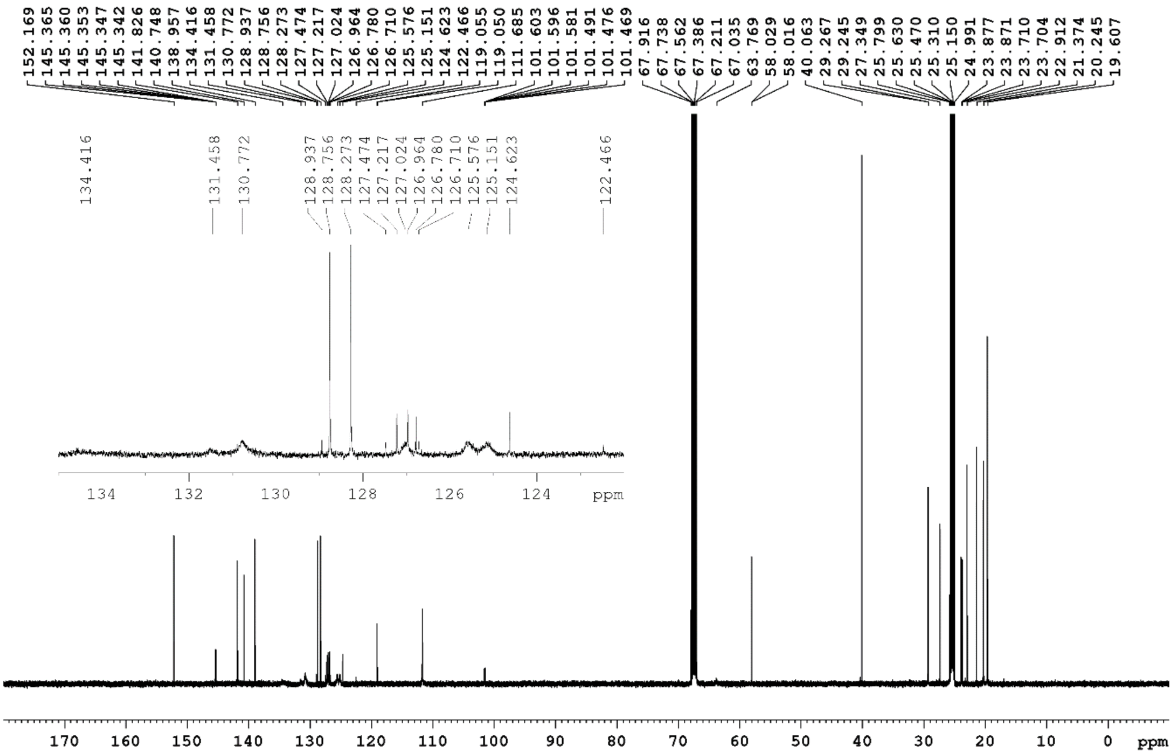

Figure S41. $^{13}C\{^{1}H\}$ NMR (125.8 MHz, d_8-THF, 298 K) spectrum of **1j**.

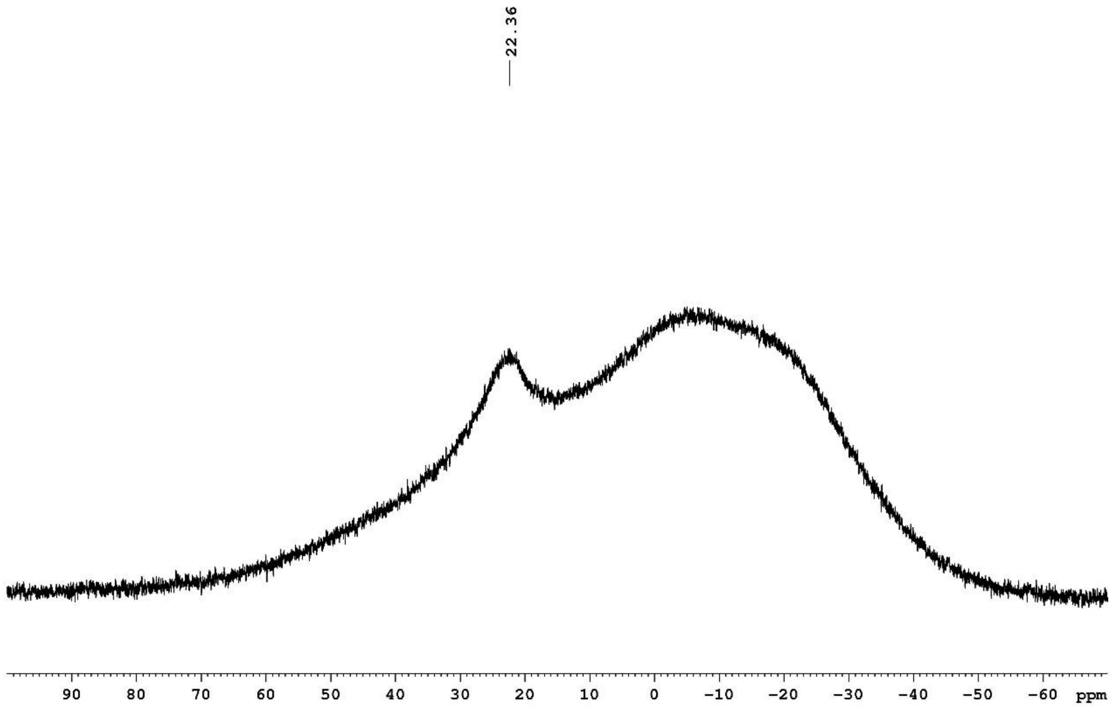

Figure S42. ^{11}B NMR (128.5 MHz, d_8-THF, 298 K) spectrum of **1j**.

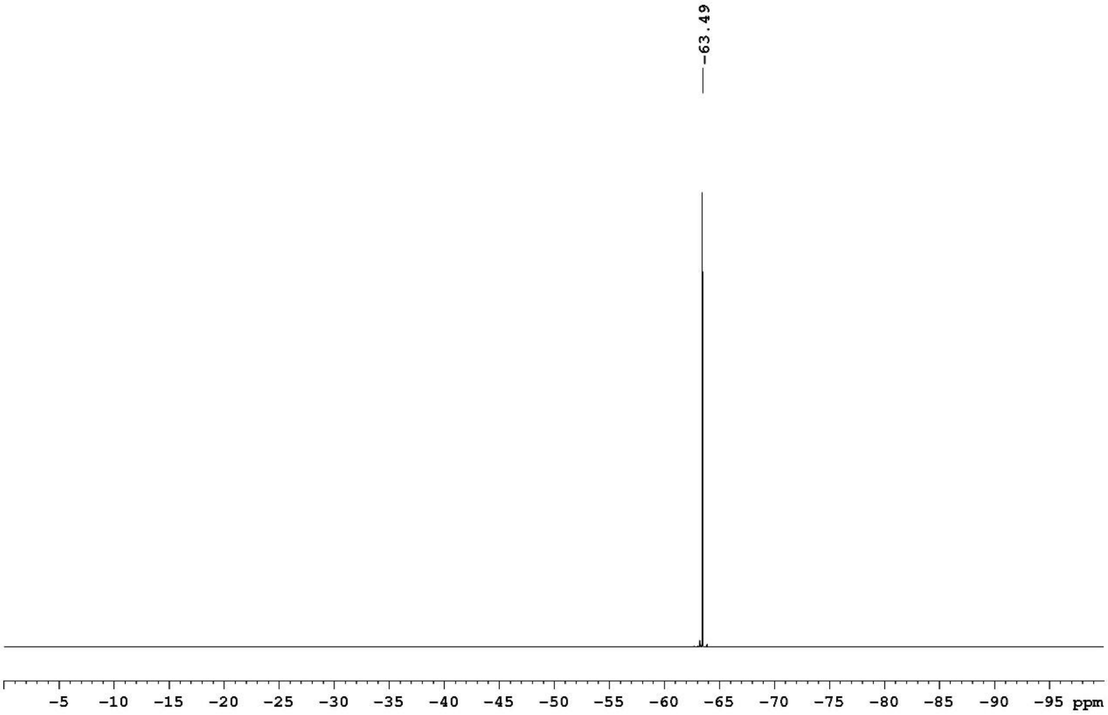

Figure S43. ^{19}F NMR (470.6 MHz, d_8-THF, 298 K) spectrum of **1j**.

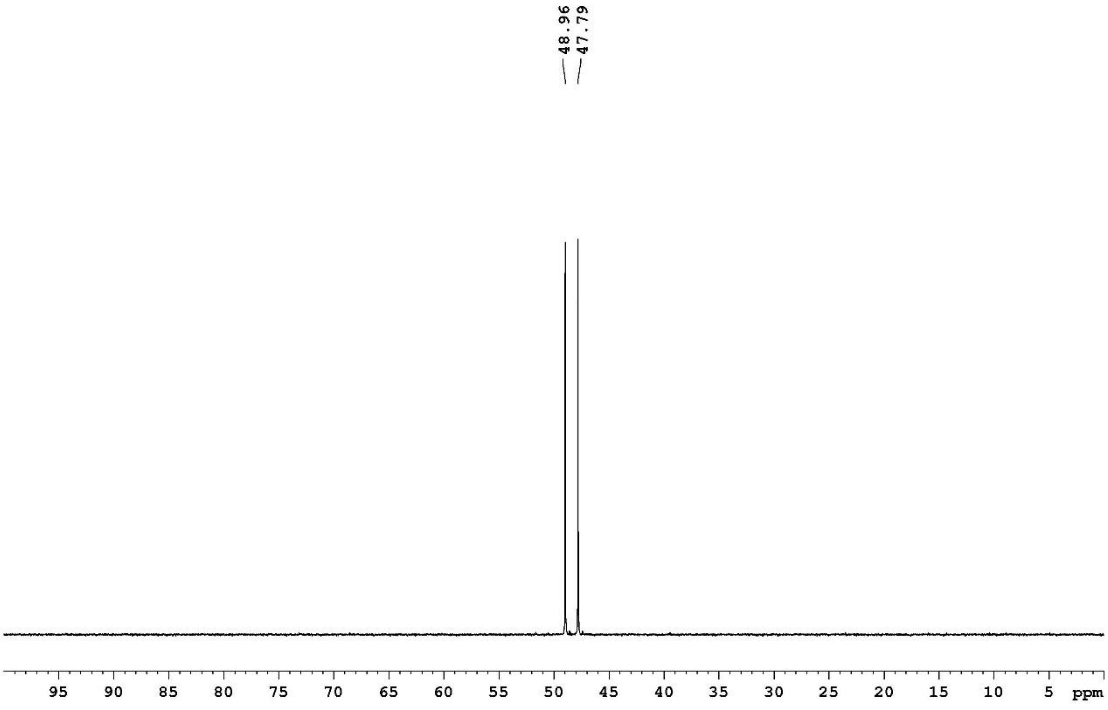

Figure S44. ^{31}P{^{1}H} NMR (162.0 MHz, d_8-THF, 298 K) spectrum of **1j**.

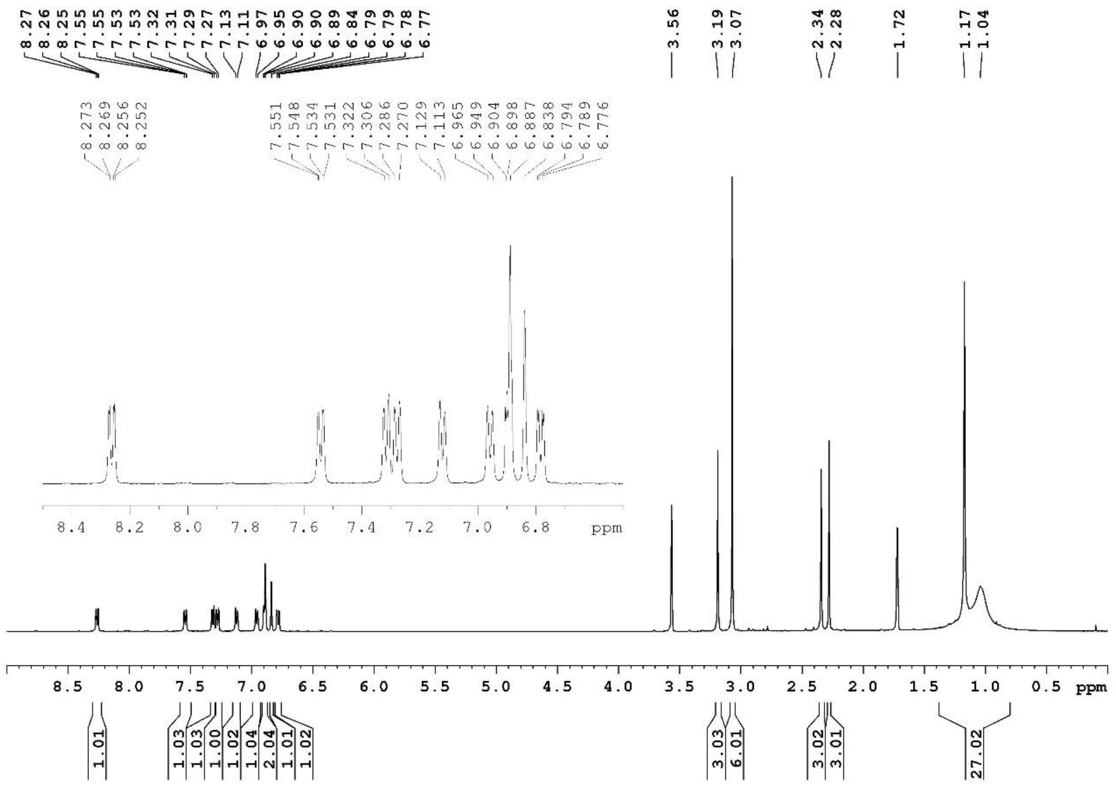

Figure S45. ^{1}H NMR (500.1 MHz, d_8-THF, 233 K) spectrum of **1j**.

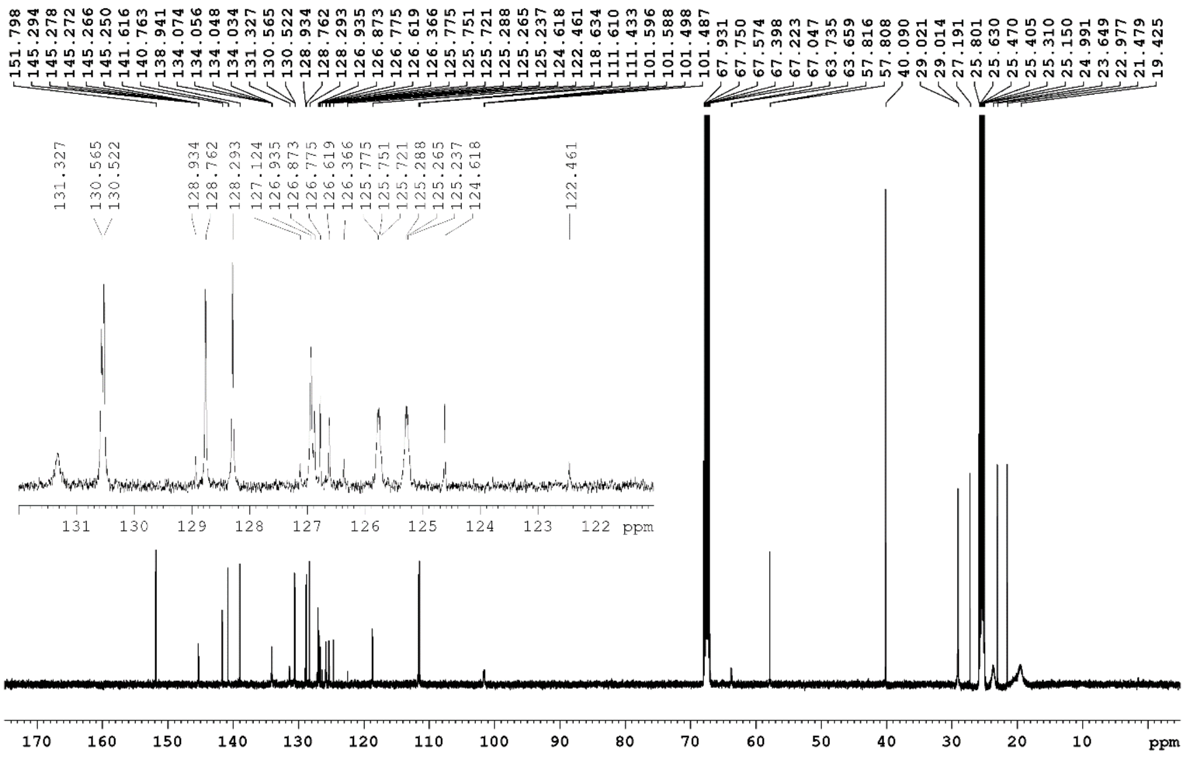

Figure S46. ^{13}C{^{1}H} NMR (125.8 MHz, d_8-THF, 233 K) spectrum of **1j**.

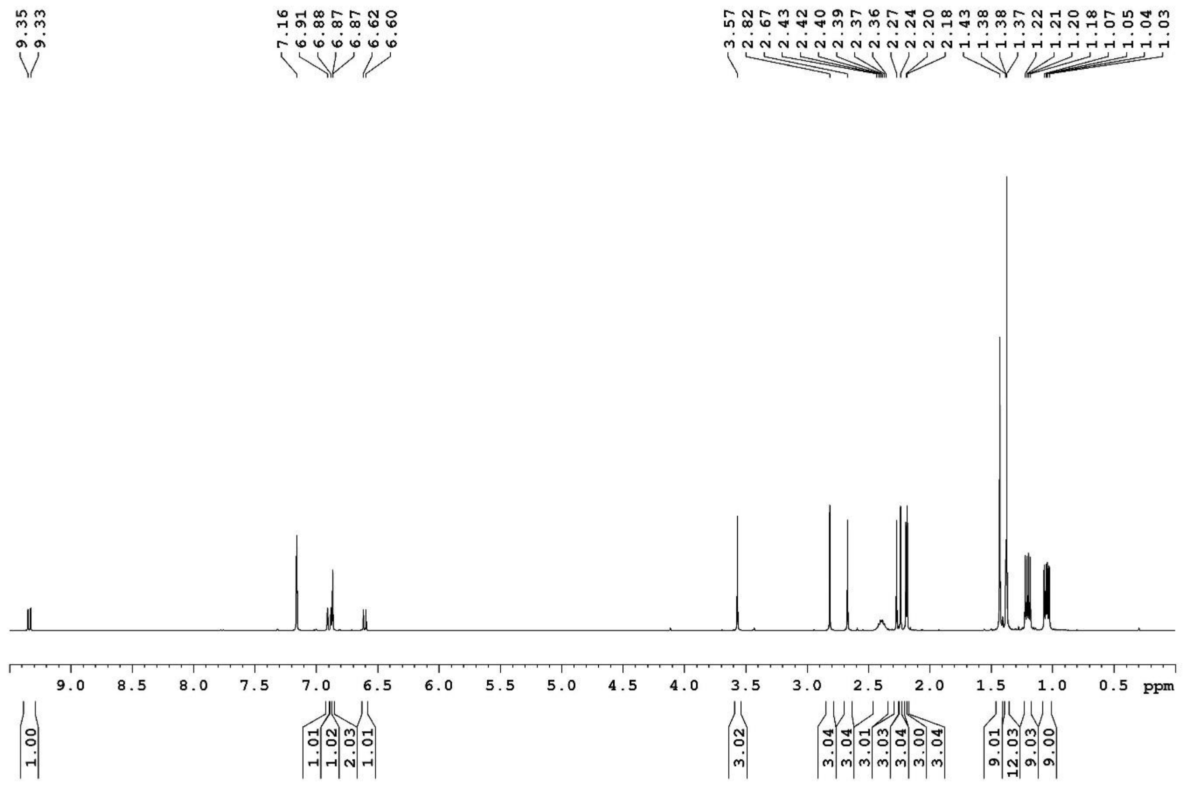

Figure S47. ^{1}H NMR (500.1 MHz, C_6D_6, 298 K) spectrum of **1k**.

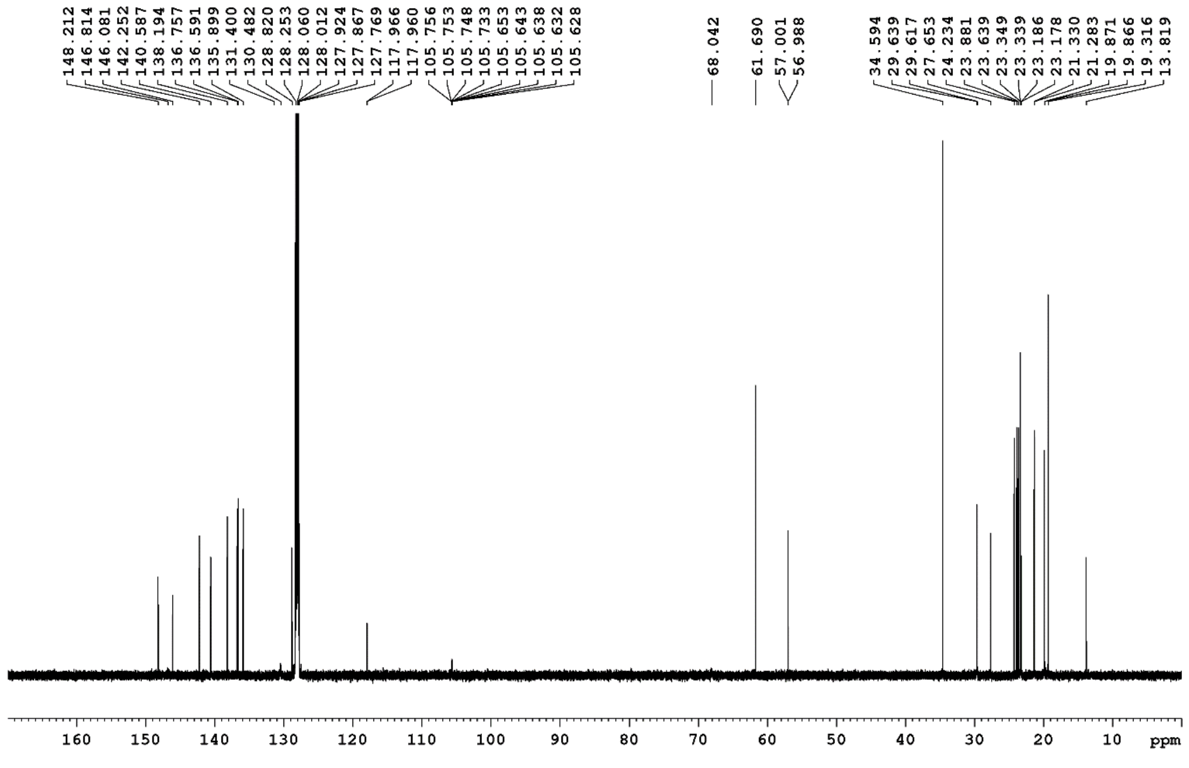

Figure S48. $^{13}C\{^{1}H\}$ NMR (125.8 MHz, C_6D_6, 298 K) spectrum of **1k**.

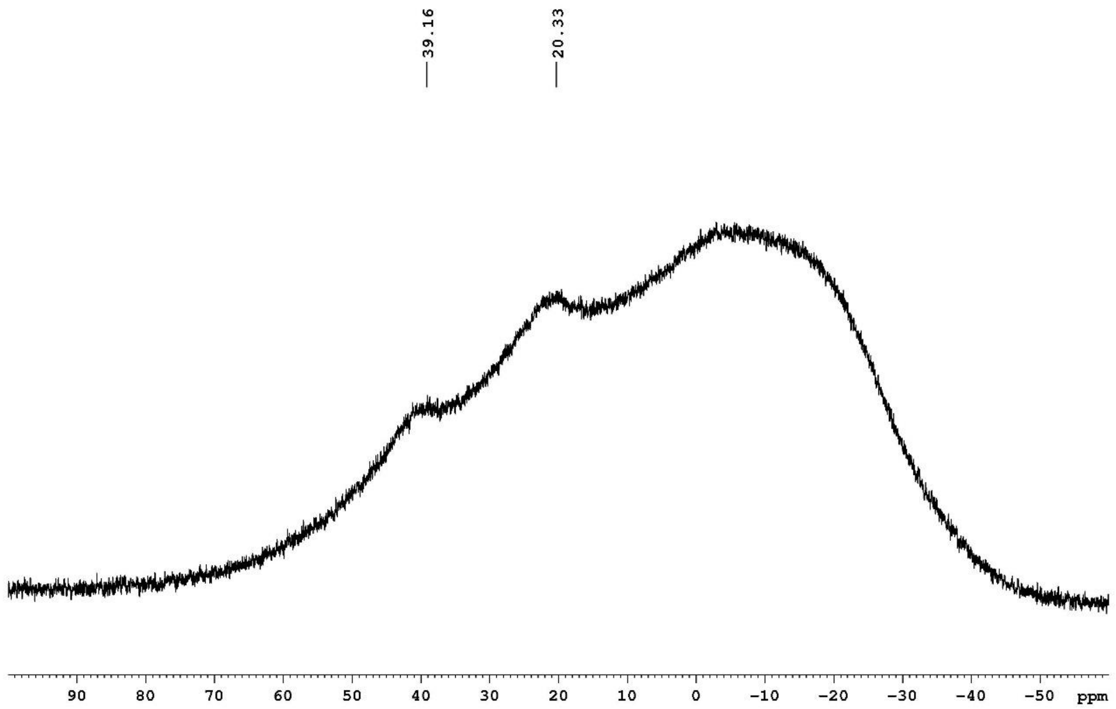

Figure S49. ^{11}B NMR (128.5 MHz, C_6D_6, 298 K) spectrum of **1k**.

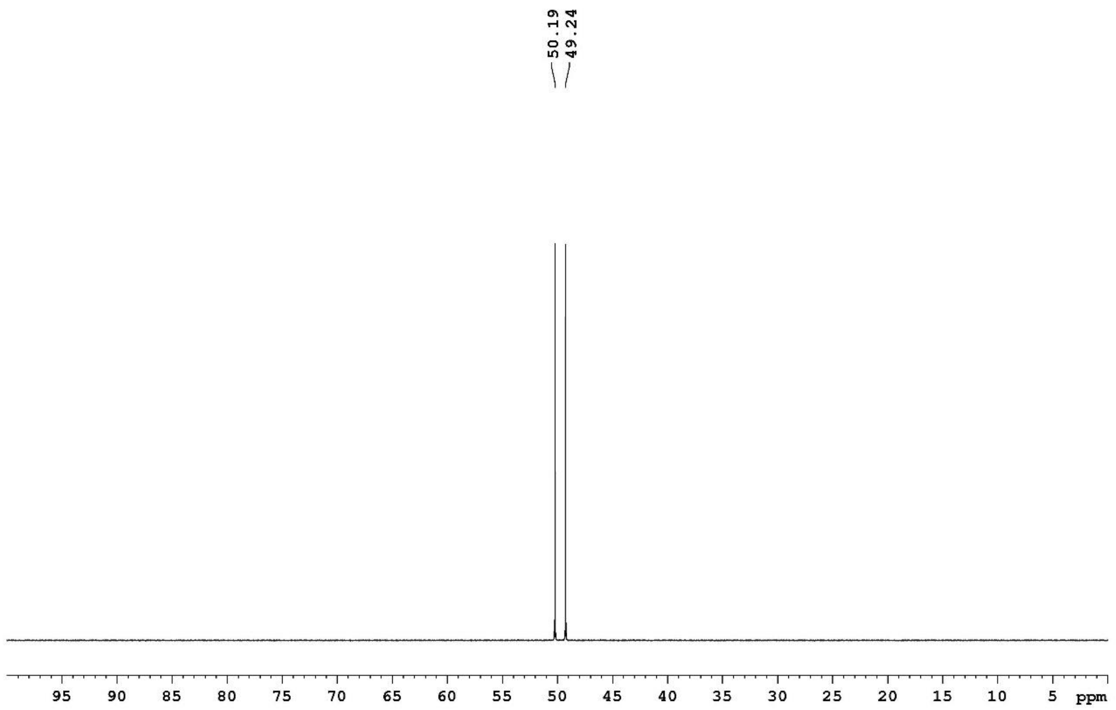

Figure S50. $^{31}P\{^1H\}$ NMR (202.5 MHz, C_6D_6, 298 K) spectrum of **1k**.

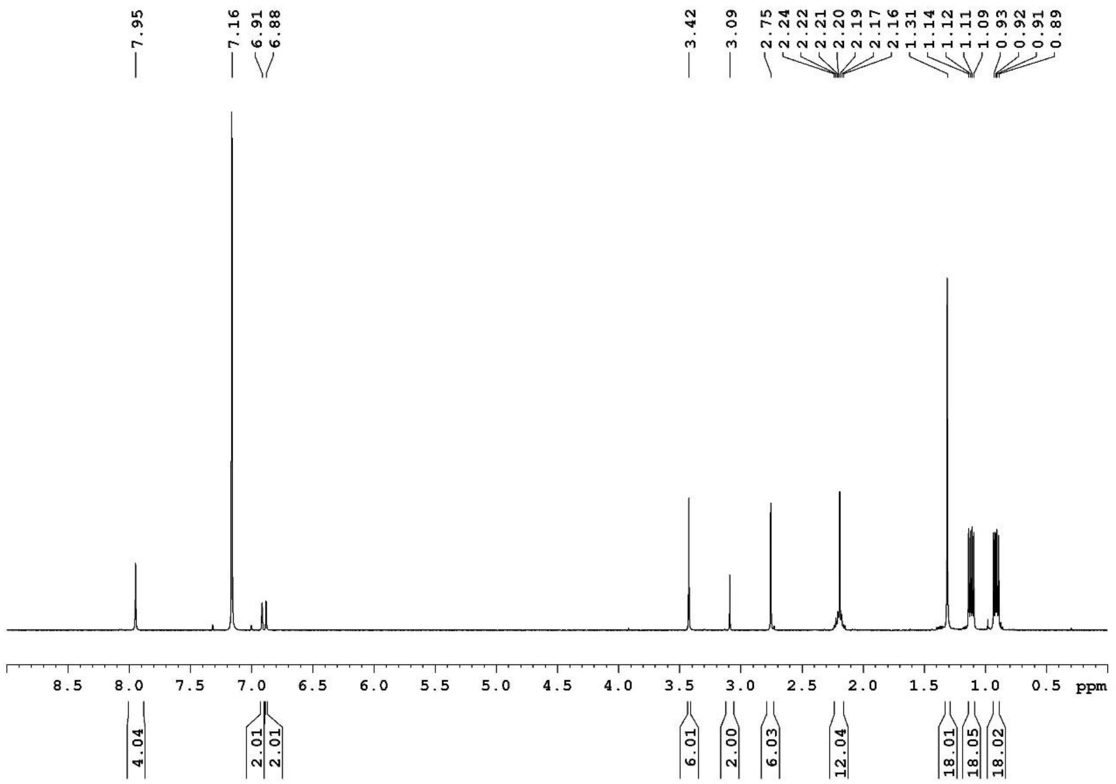

Figure S51. ^{1}H NMR (500.1 MHz, C_6D_6, 298 K) spectrum of **1l**.

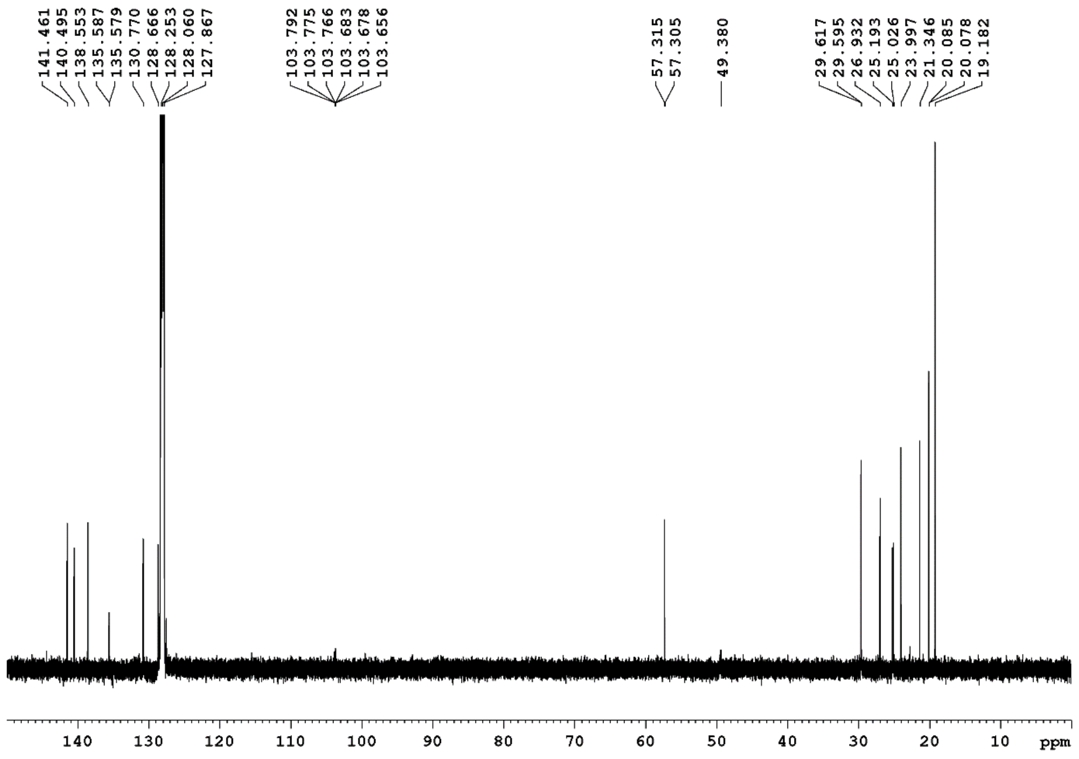

Figure S52. $^{13}C\{^{1}H\}$ NMR (125.8 MHz, C_6D_6, 298 K) spectrum of **1l**.

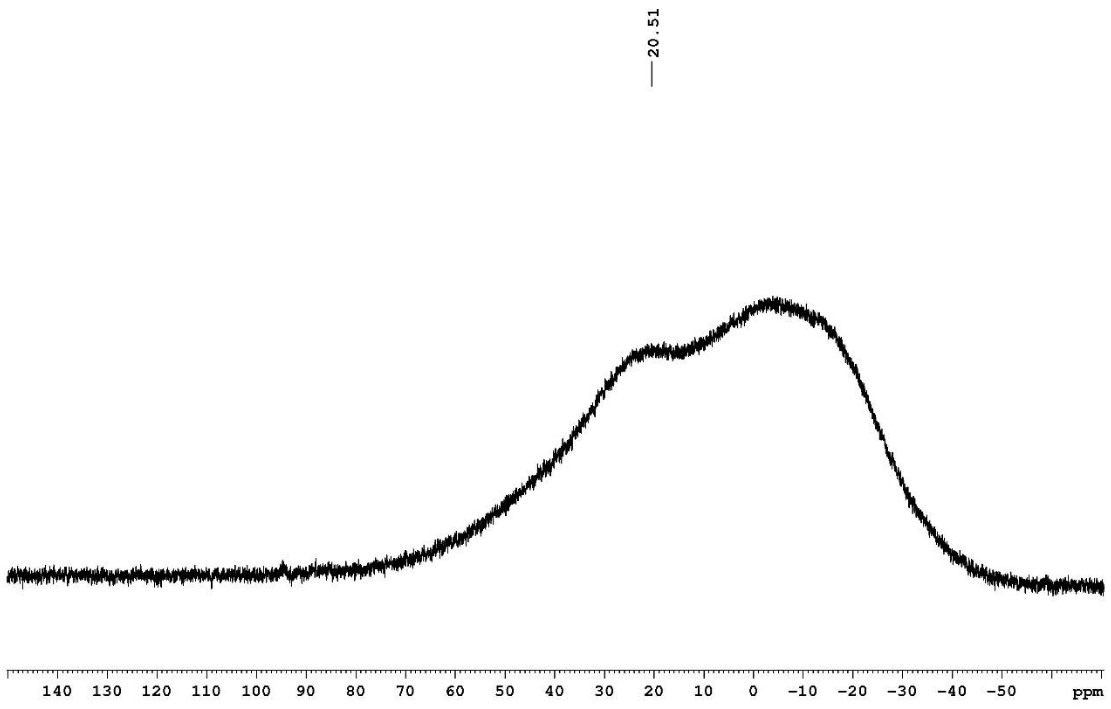

Figure S53. ^{11}B NMR (128.4 MHz, C_6D_6, 298 K) spectrum of **1l**.

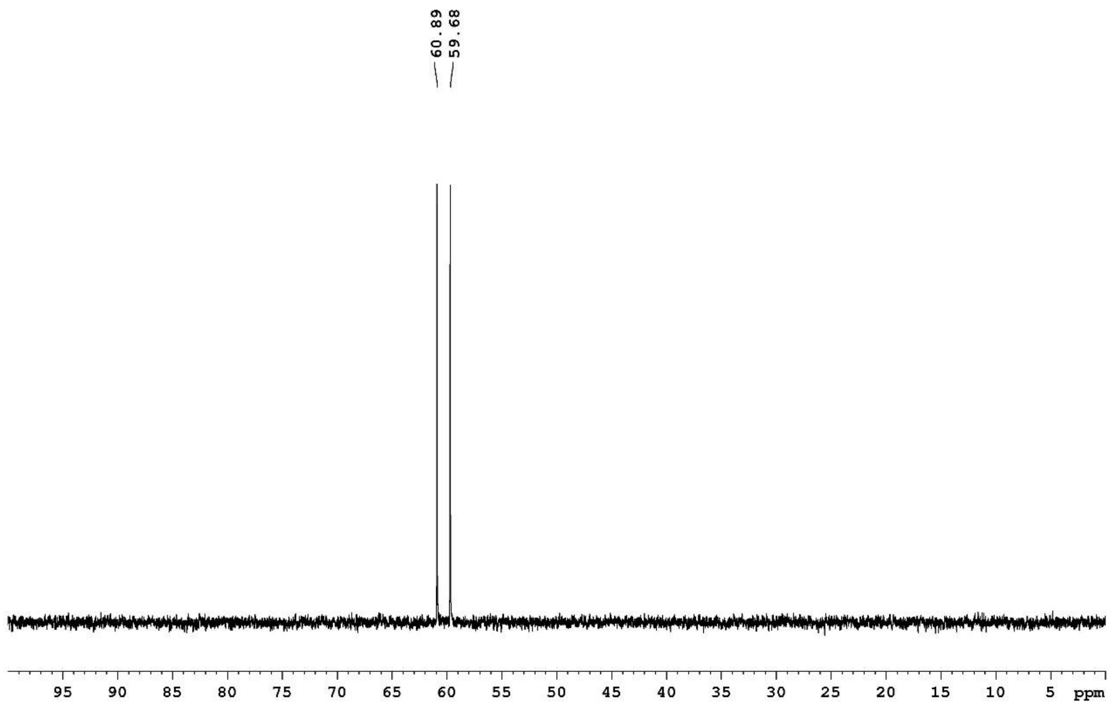

Figure S54. $^{31}P\{^1H\}$ NMR (162.0 MHz, C_6D_6, 298 K) spectrum of **1l**.

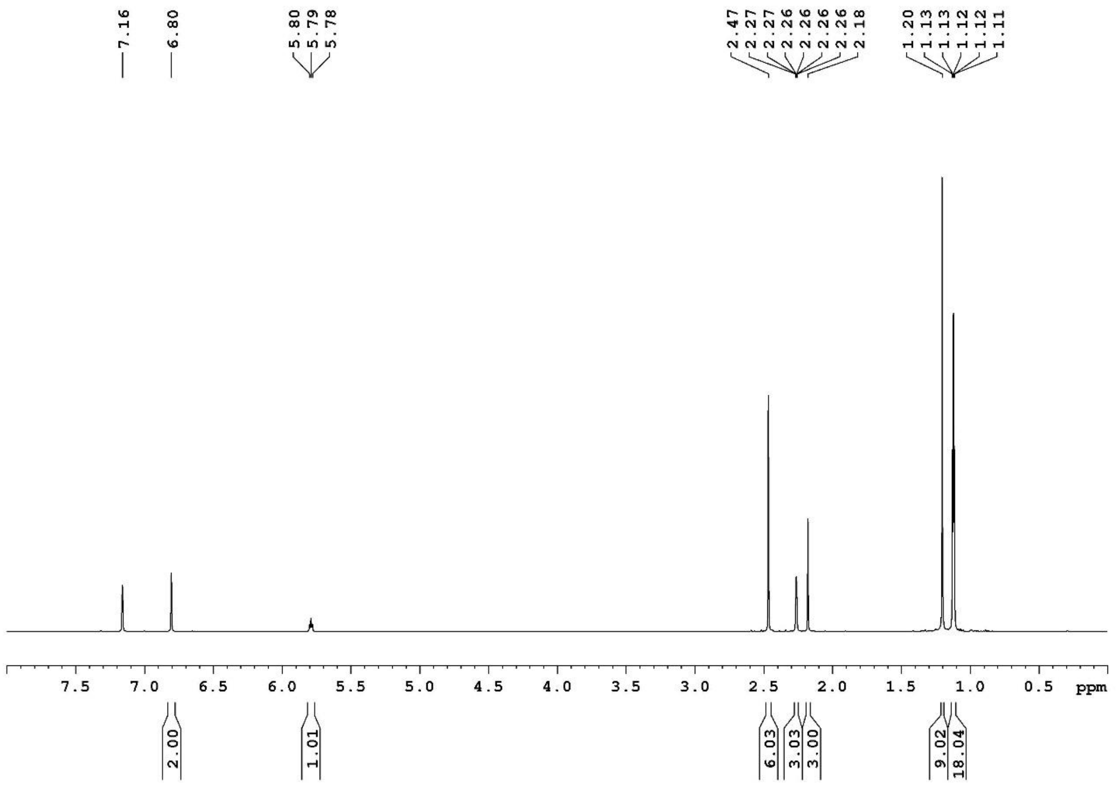

Figure S55. ^{1}H NMR (500.1 MHz, C_6D_6, 298 K) spectrum of **2a**.

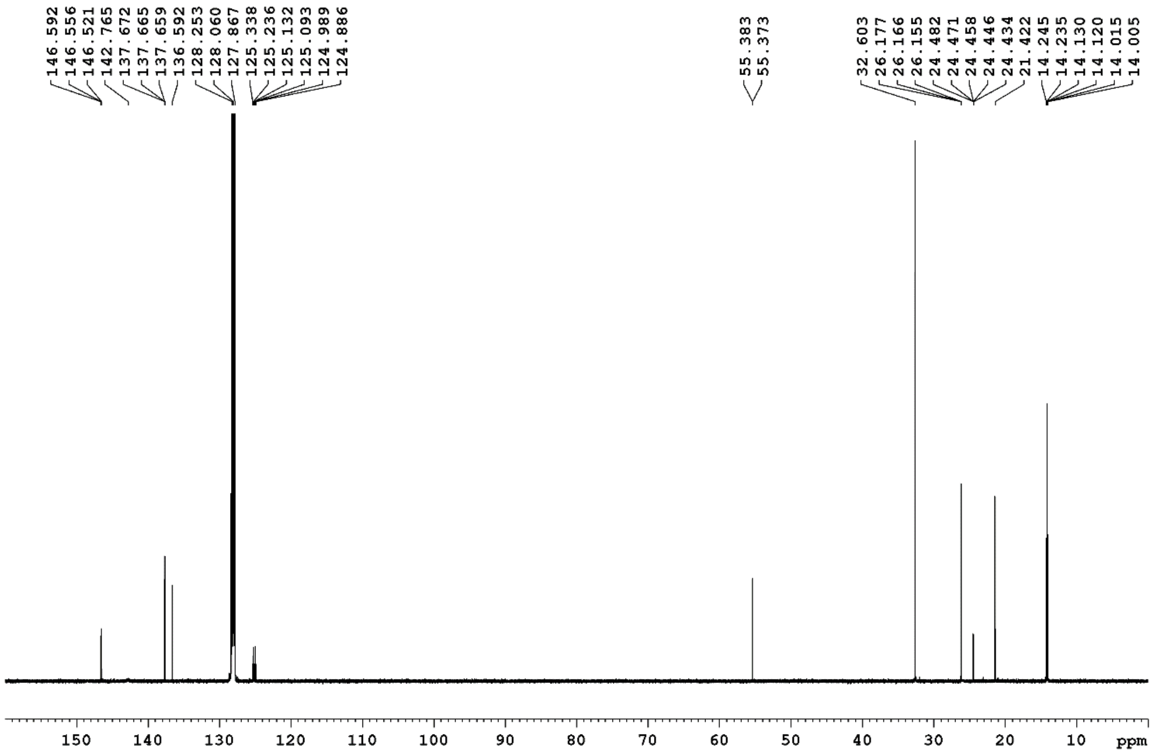

Figure S56. $^{13}C\{^{1}H\}$ NMR (125.8 MHz, C_6D_6, 298 K) spectrum of **2a**.

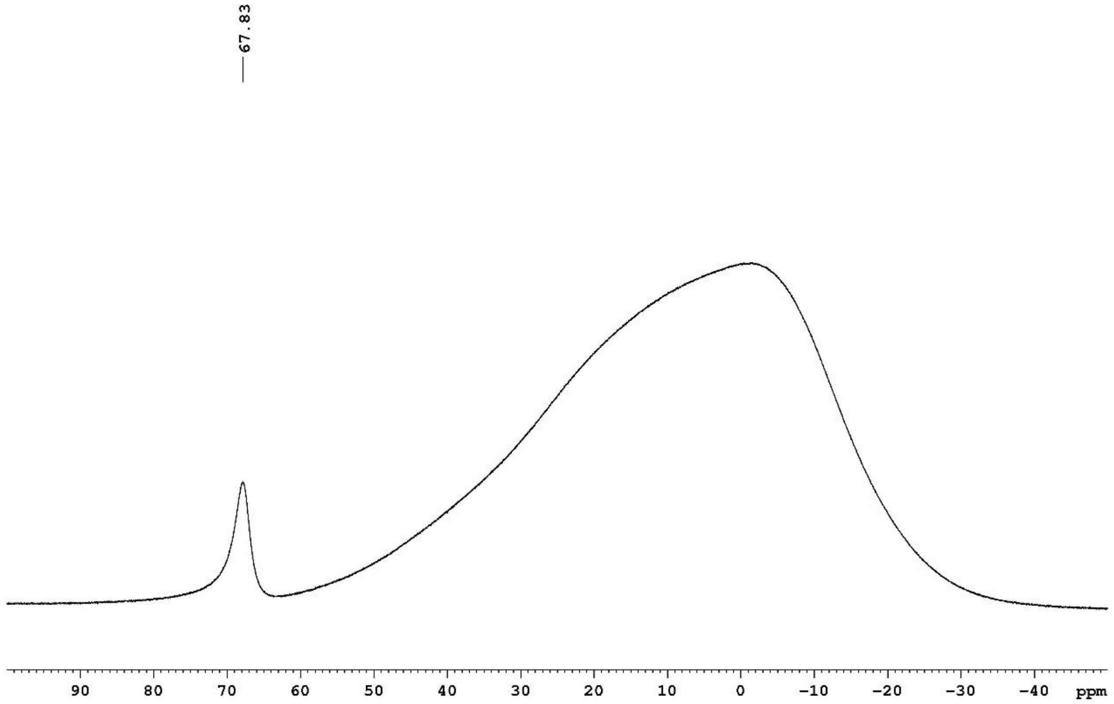

Figure S57. ^{11}B NMR (160.5 MHz, C_6D_6, 298 K) spectrum of **2a**.

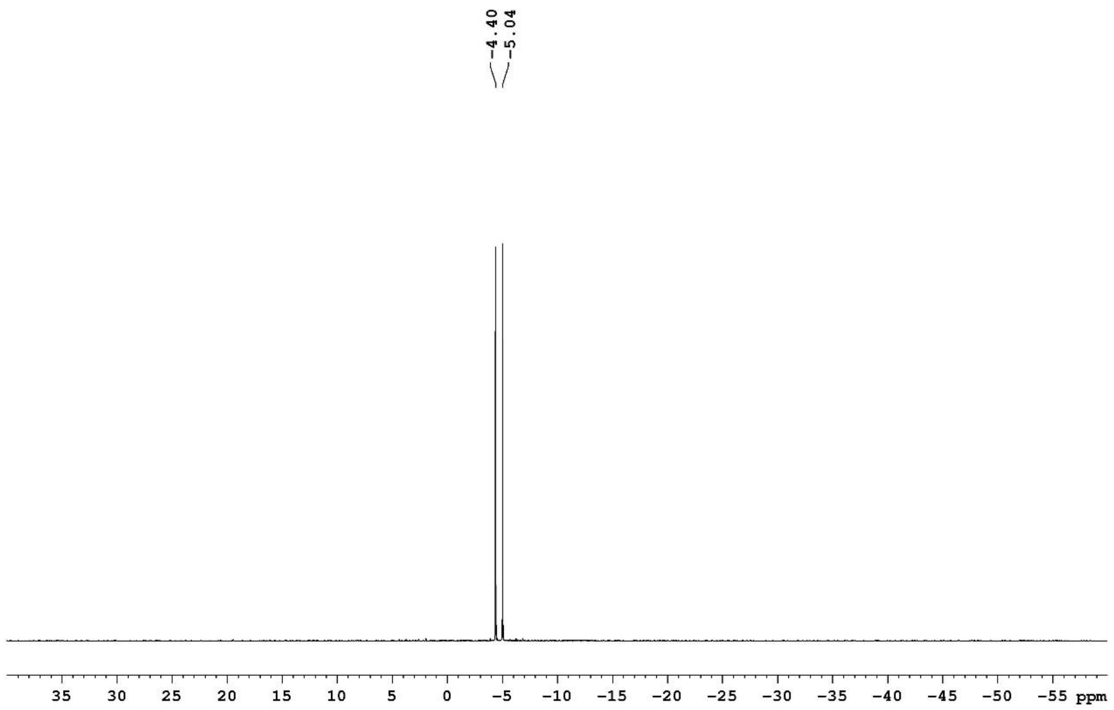

Figure S58. $^{31}P\{^1H\}$ NMR (202.5 MHz, C_6D_6, 298 K) spectrum of **2a**.

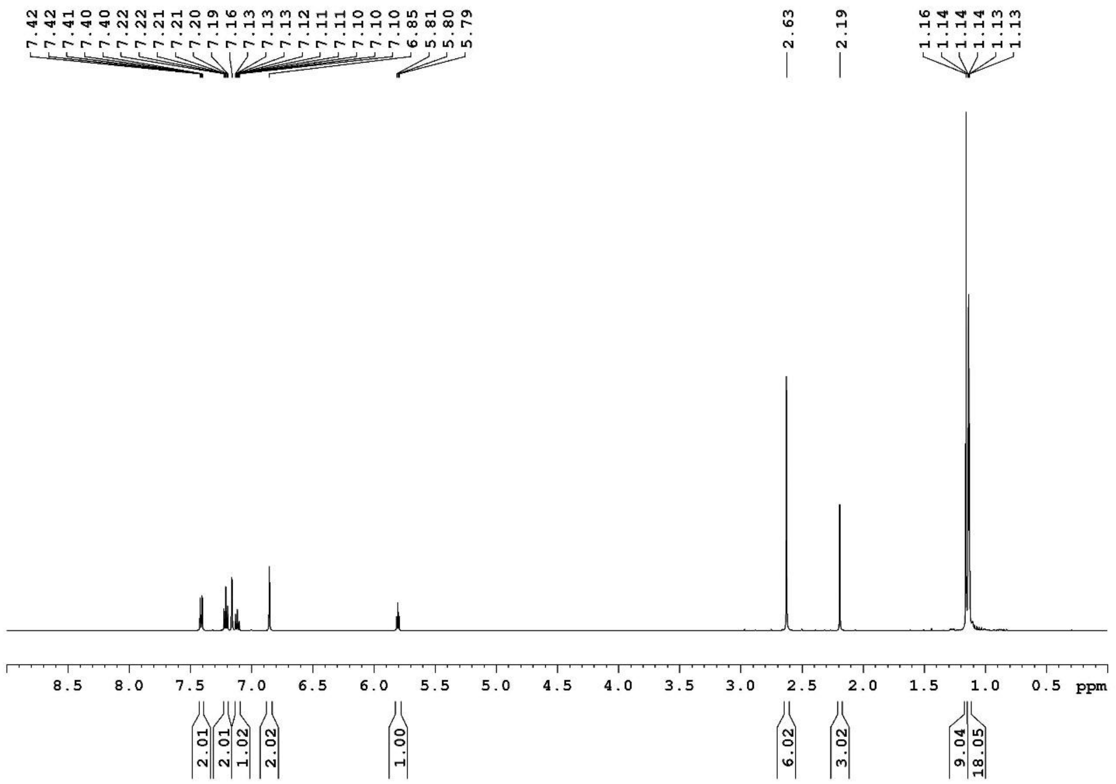

Figure S59. ^{1}H NMR (500.1 MHz, C_6D_6, 298 K) spectrum of **2b**.

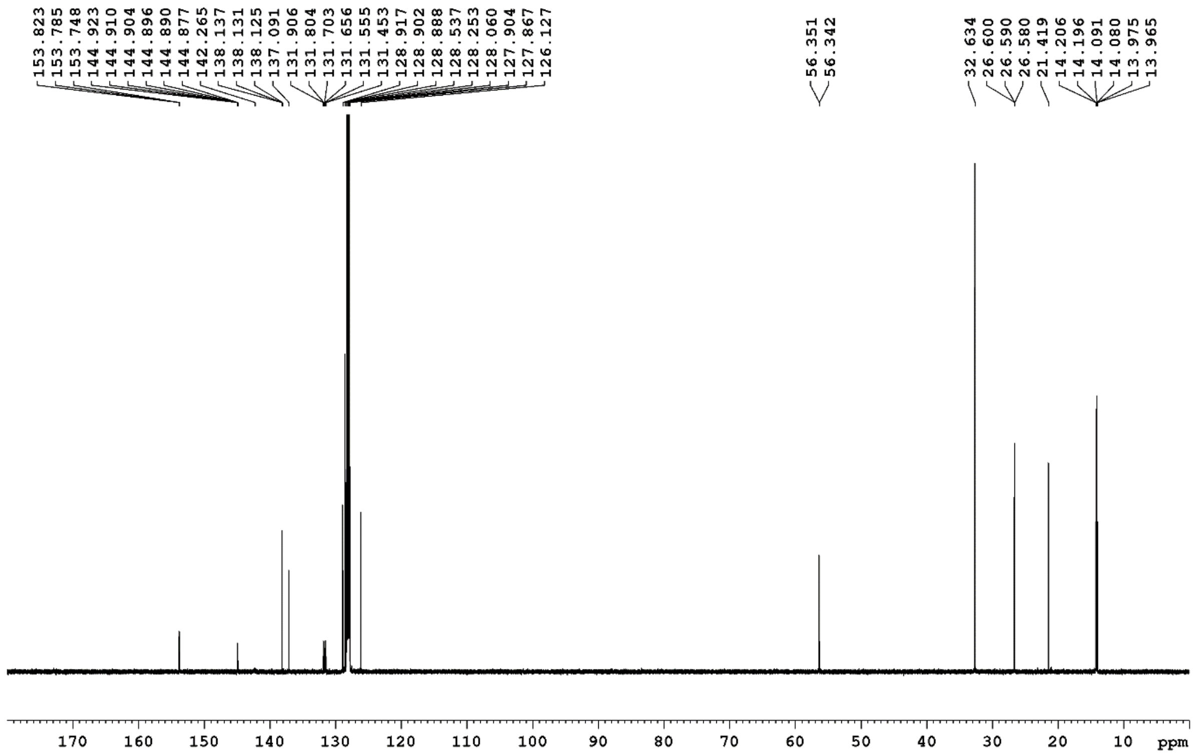

Figure S60. $^{13}C\{^{1}H\}$ NMR (125.8 MHz, C_6D_6, 298 K) spectrum of **2b**.

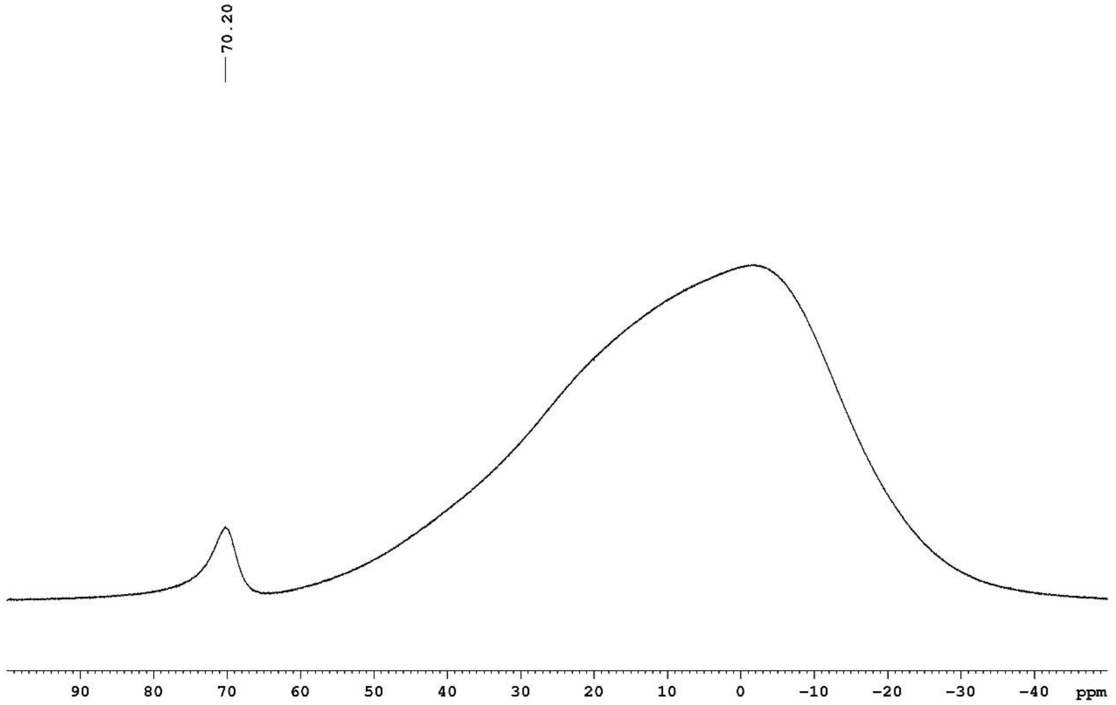

Figure S61. ^{11}B NMR (160.5 MHz, C_6D_6, 298 K) spectrum of **2b**.

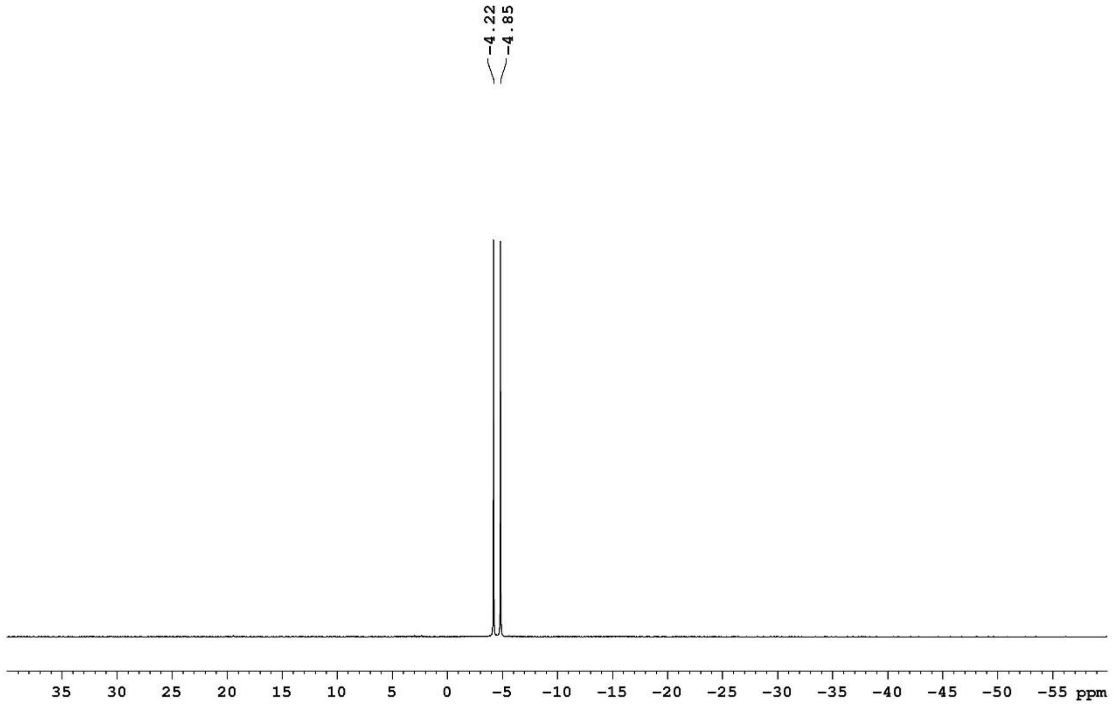

Figure S62. $^{31}P\{^1H\}$ NMR (202.5 MHz, C_6D_6, 298 K) spectrum of **2b**.

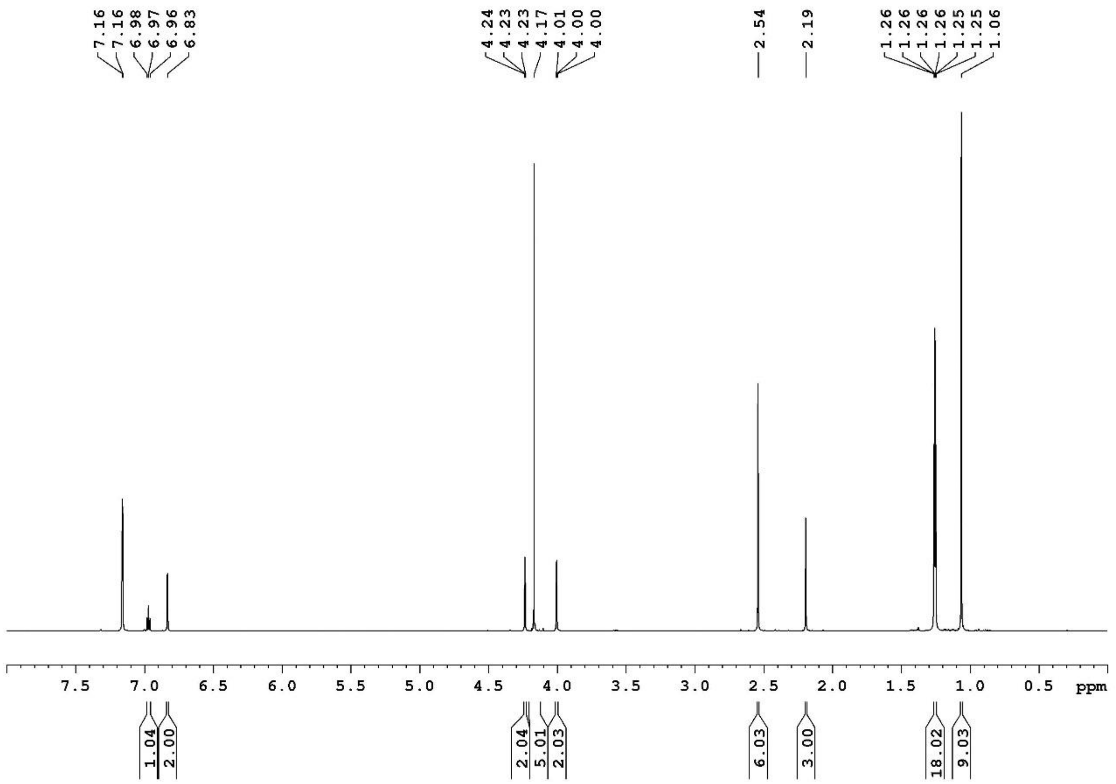

Figure S63. ^{1}H NMR (500.1 MHz, C_6D_6, 298 K) spectrum of **2c**.

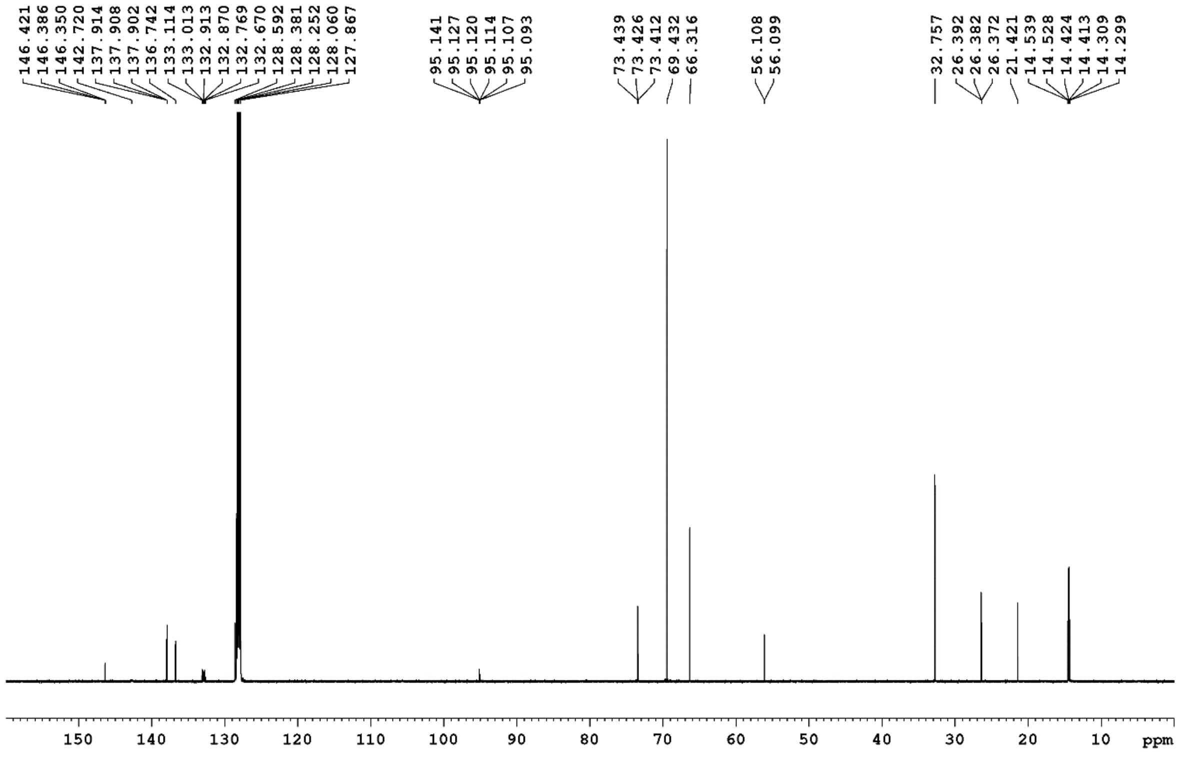

Figure S64. $^{13}C\{^{1}H\}$ NMR (125.8 MHz, C_6D_6, 298 K) spectrum of **2c**.

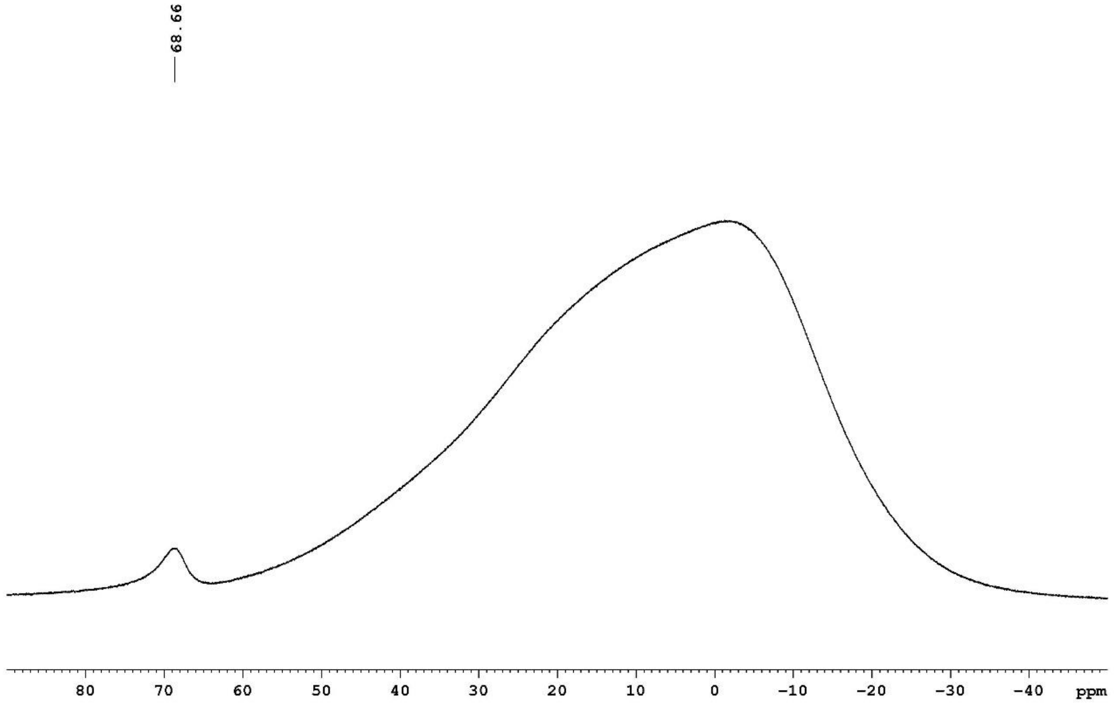

Figure S65. ^{11}B NMR (160.5 MHz, C_6D_6, 298 K) spectrum of **2c**.

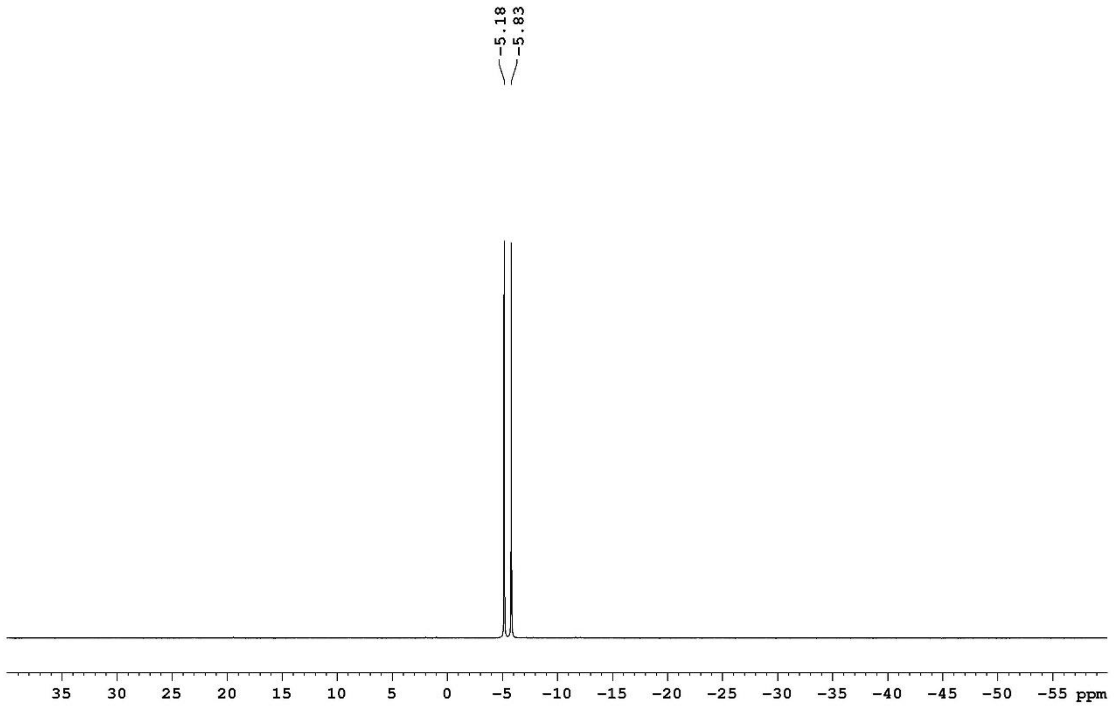

Figure S66. $^{31}P\{^1H\}$ NMR (202.5 MHz, C_6D_6, 298 K) spectrum of **2c**.

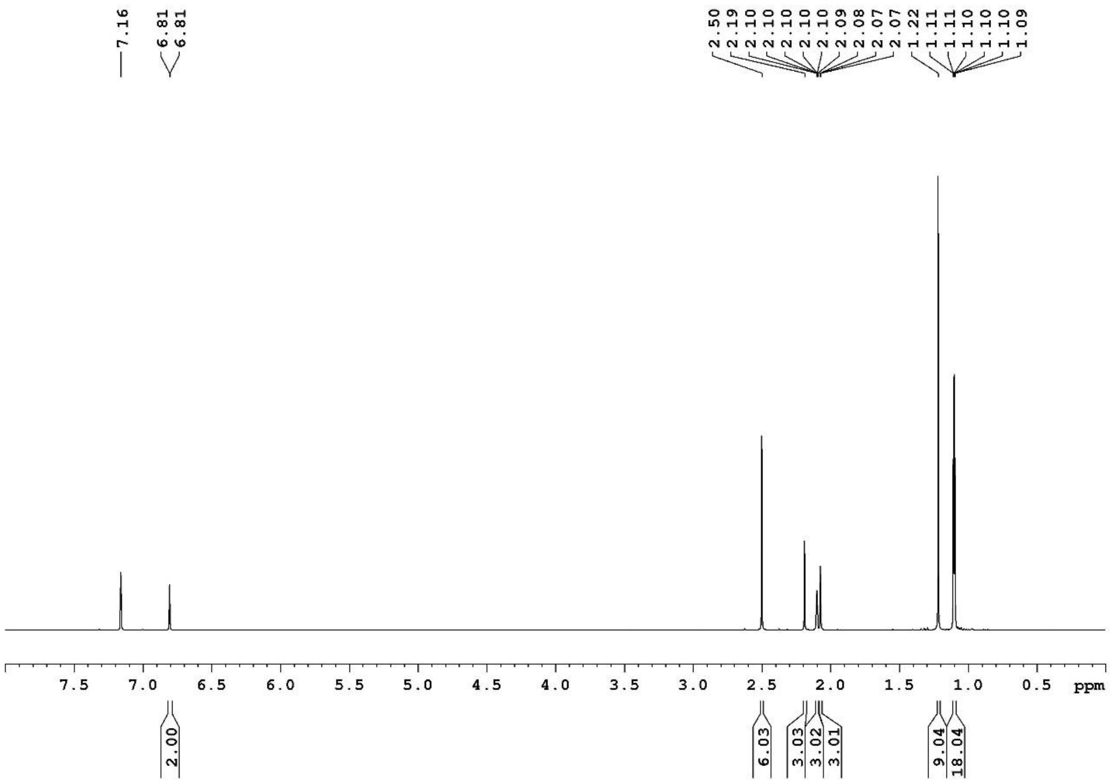

Figure S67. ^{1}H NMR (500.1 MHz, C_6D_6, 298 K) spectrum of **2d**.

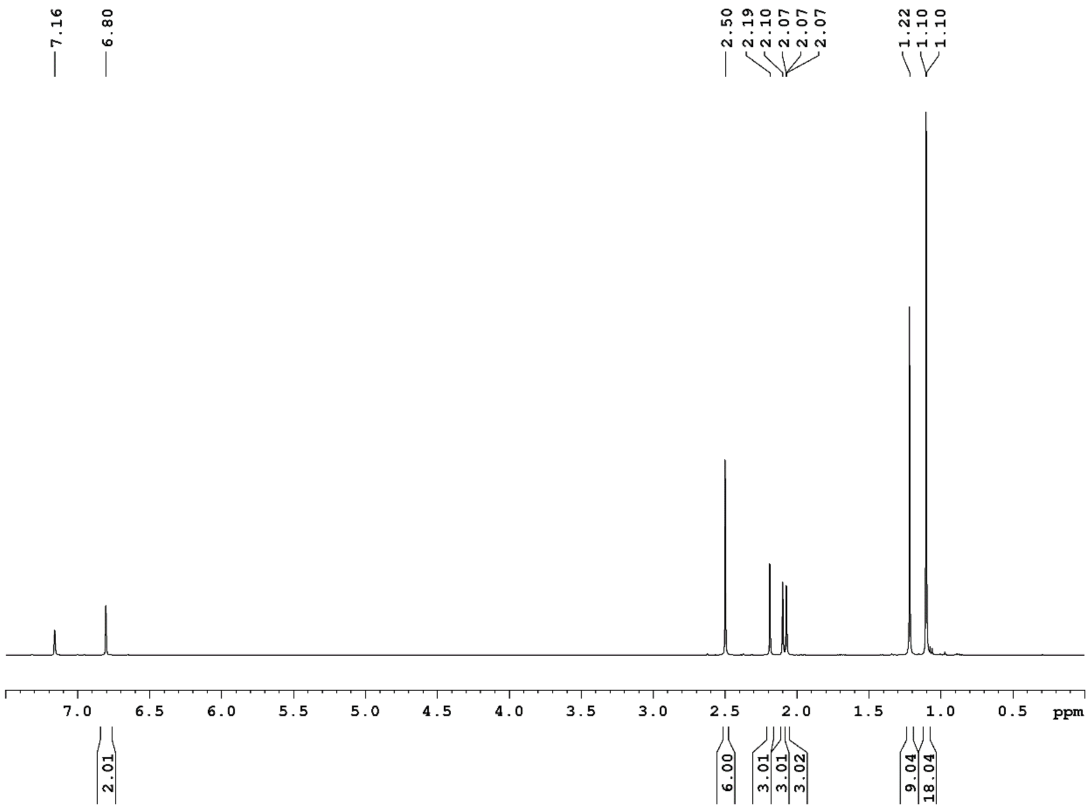

Figure S68. ^{1}H{^{31}P} NMR (500.1 MHz, C_6D_6, 298 K) spectrum of **2d**.

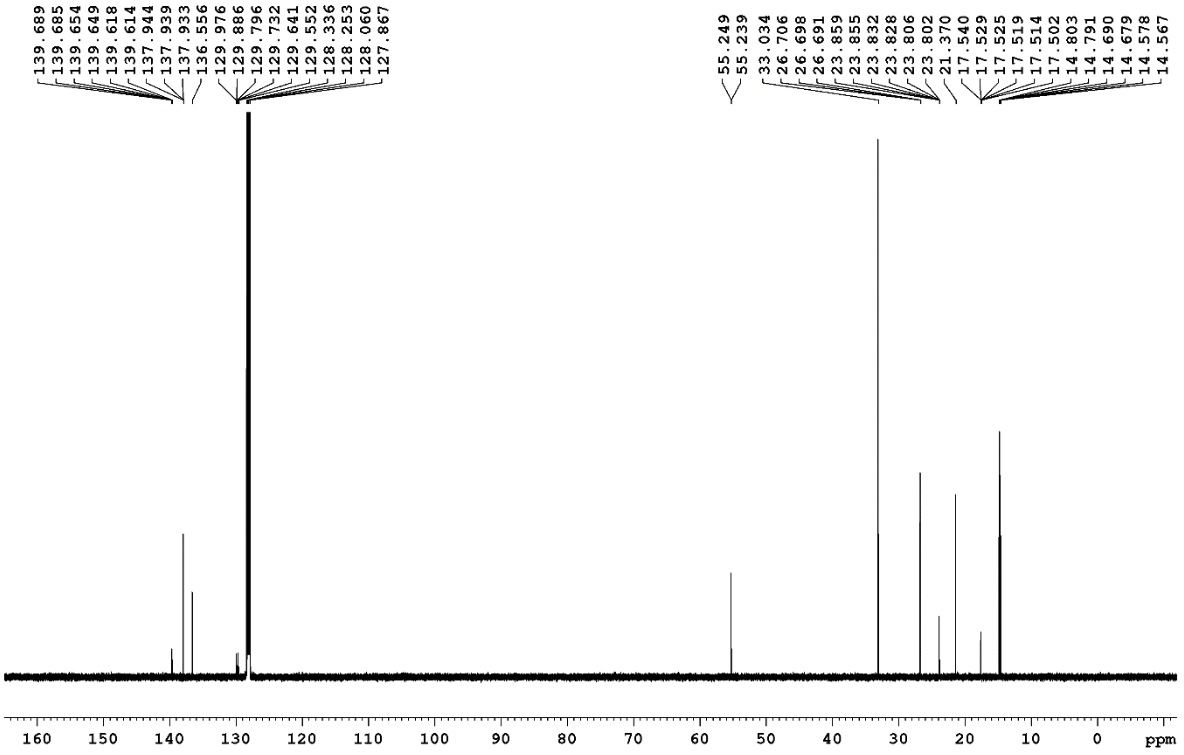

Figure S69. $^{13}C\{^{1}H\}$ NMR (125.8 MHz, C_6D_6, 298 K) spectrum of **2d**.

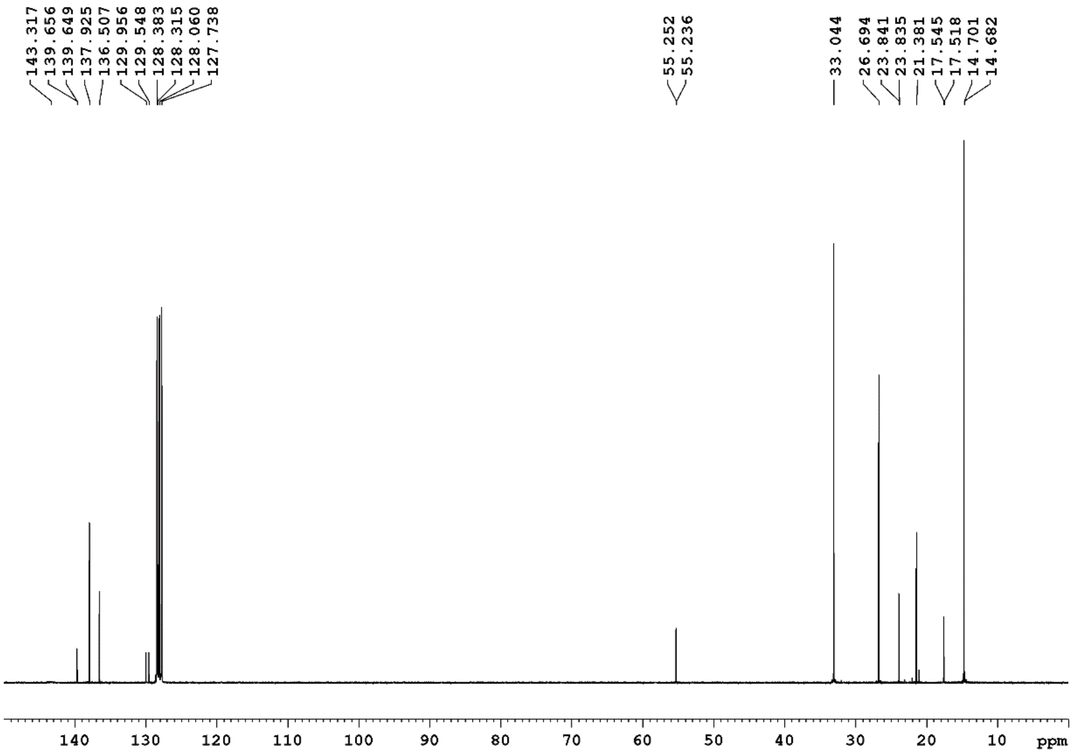

Figure S70. $^{13}C\{^{1}H, ^{31}P\}$ NMR (75.5 MHz, C_6D_6, 298 K) spectrum of **2d**.

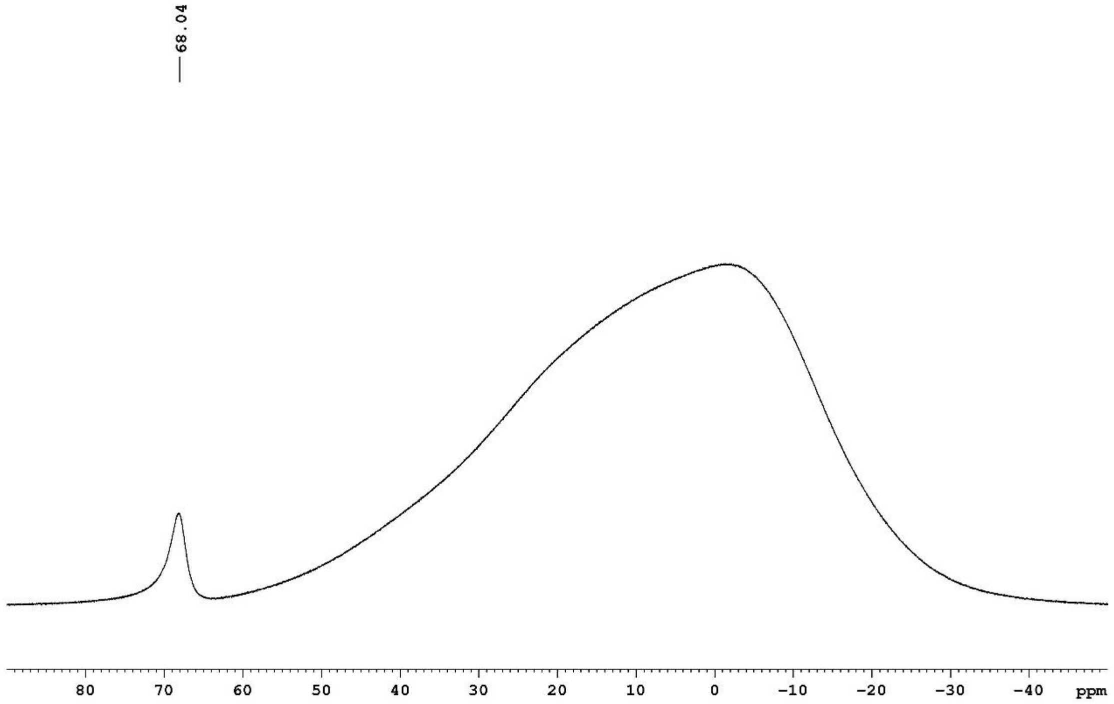

Figure S71. ^{11}B NMR (160.5 MHz, C_6D_6, 298 K) spectrum of **2d**.

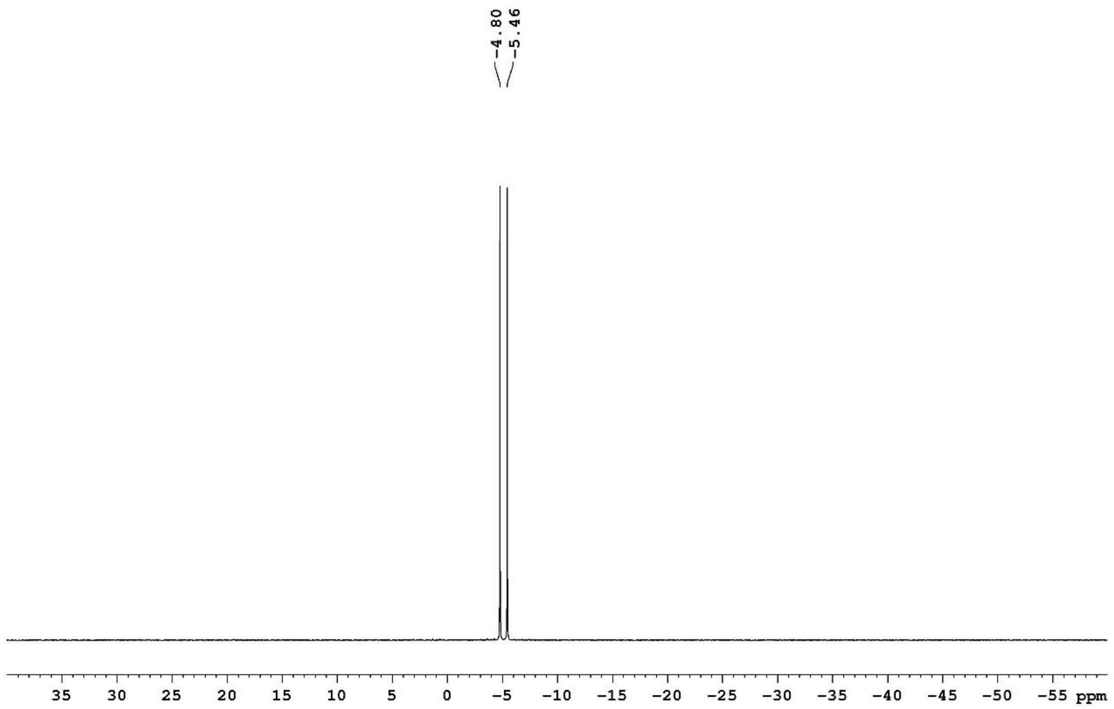

Figure S72. $^{31}P\{^{1}H\}$ NMR (202.5 MHz, C_6D_6, 298 K) spectrum of **2d**.

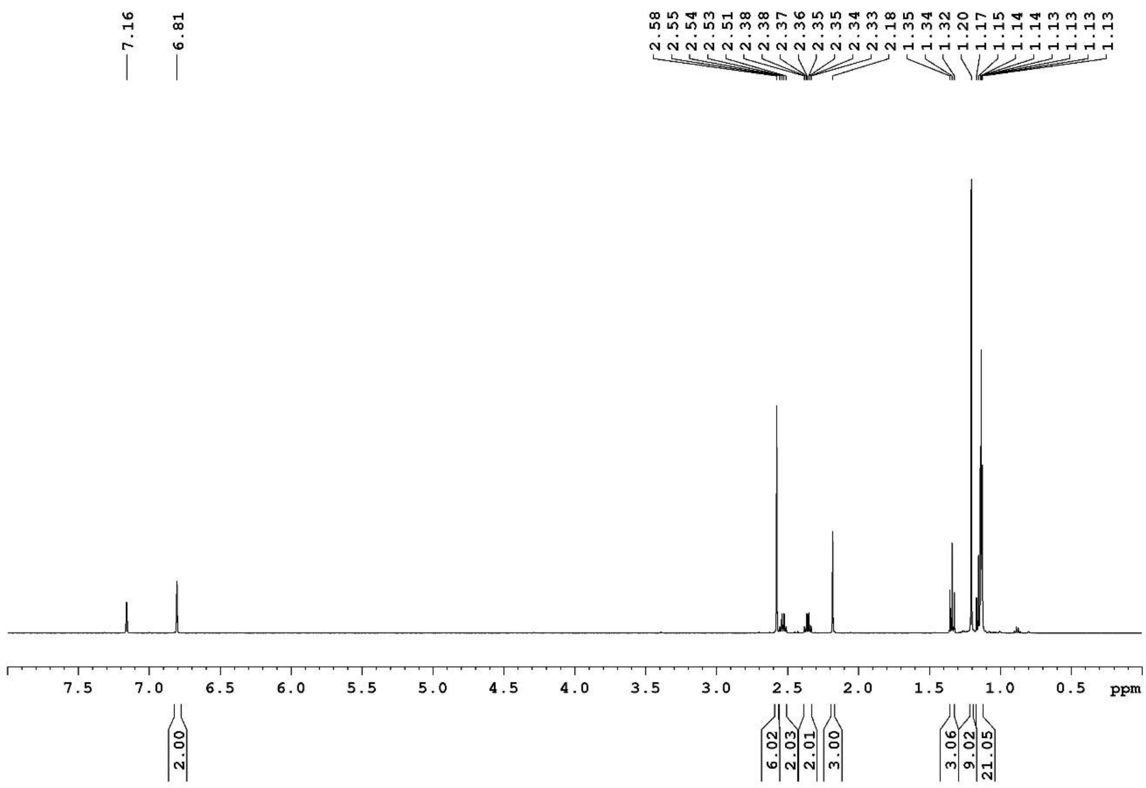

Figure S73. ^{1}H NMR (500.1 MHz, C_6D_6, 298 K) spectrum of **2e**.

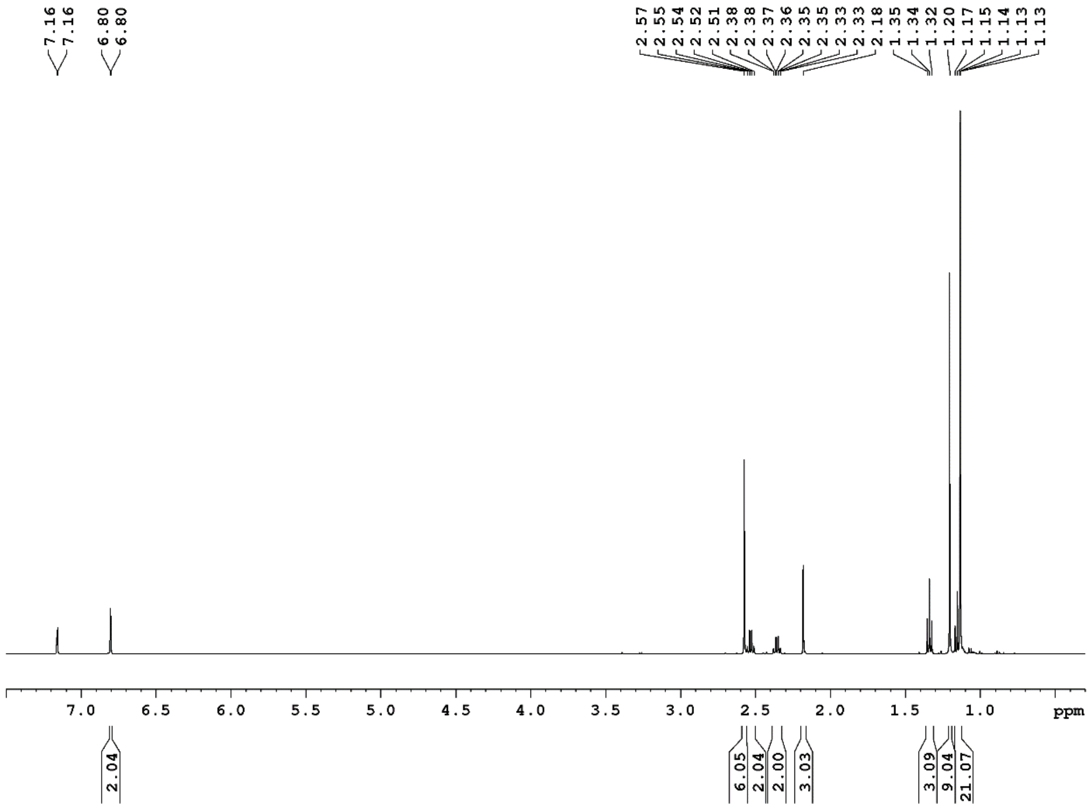

Figure S74. $^{1}H\{^{31}P\}$ NMR (500.1 MHz, C_6D_6, 298 K) spectrum of **2e**.

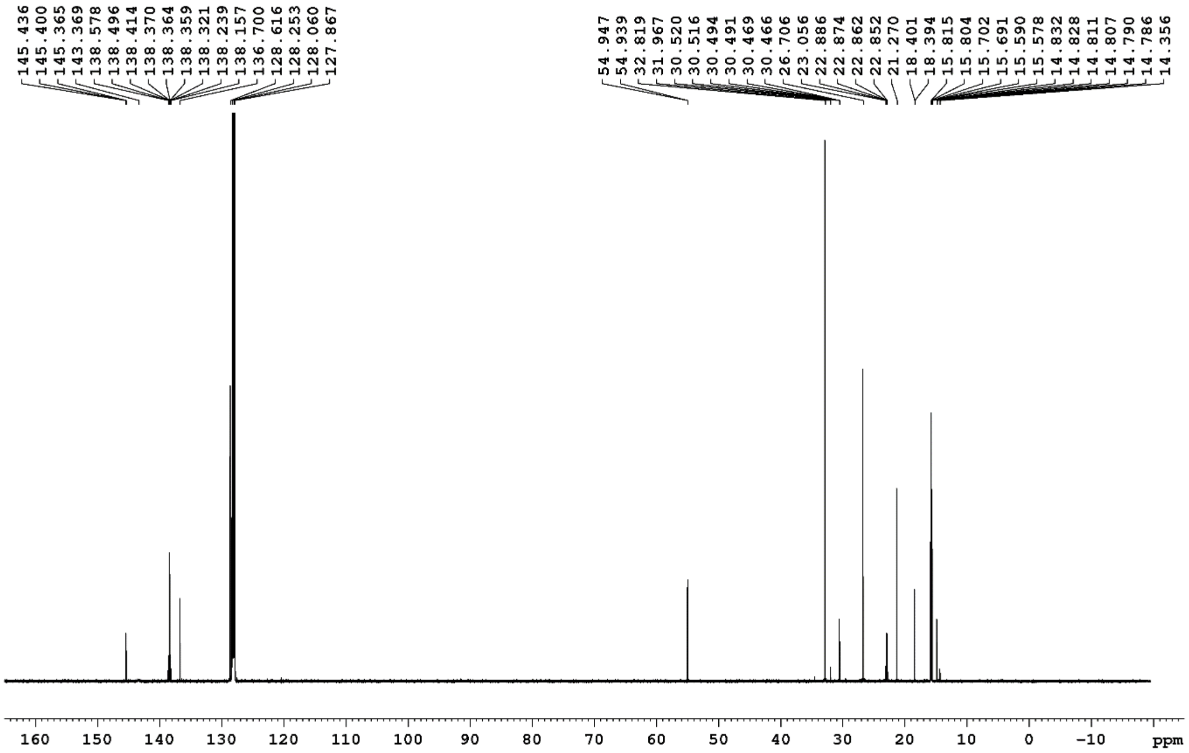

Figure S75. $^{13}C\{^{1}H\}$ NMR (125.8 MHz, C_6D_6, 298 K) spectrum of **2e**.

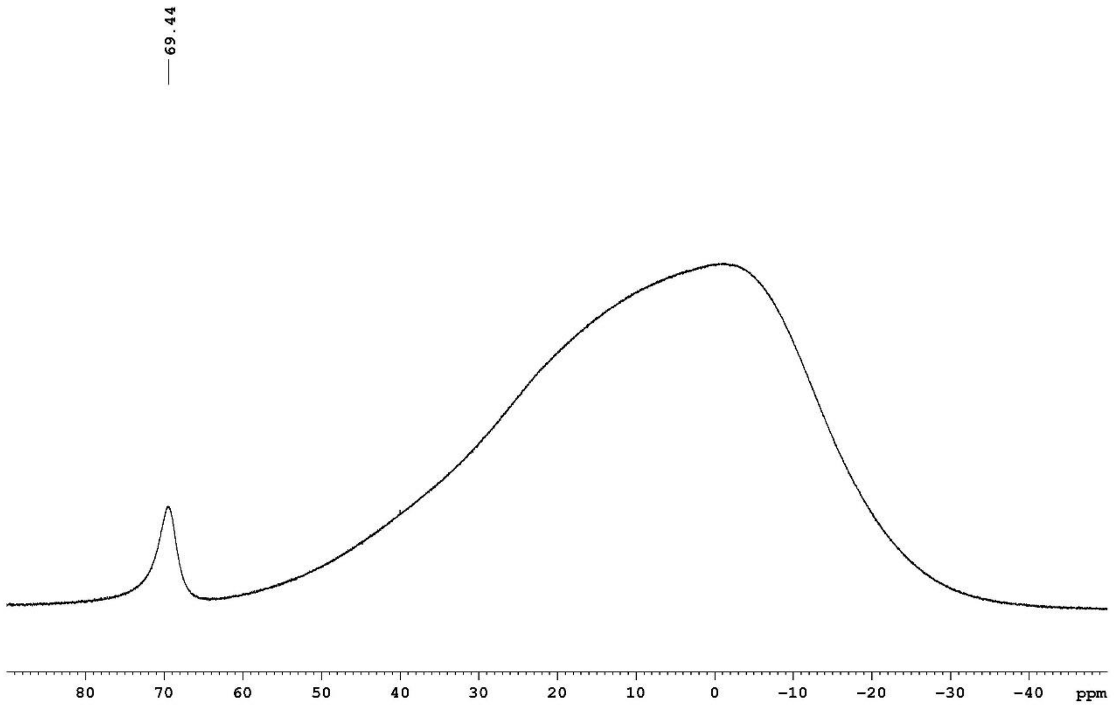

Figure S76. ^{11}B NMR (160.5 MHz, C_6D_6, 298 K) spectrum of **2e**.

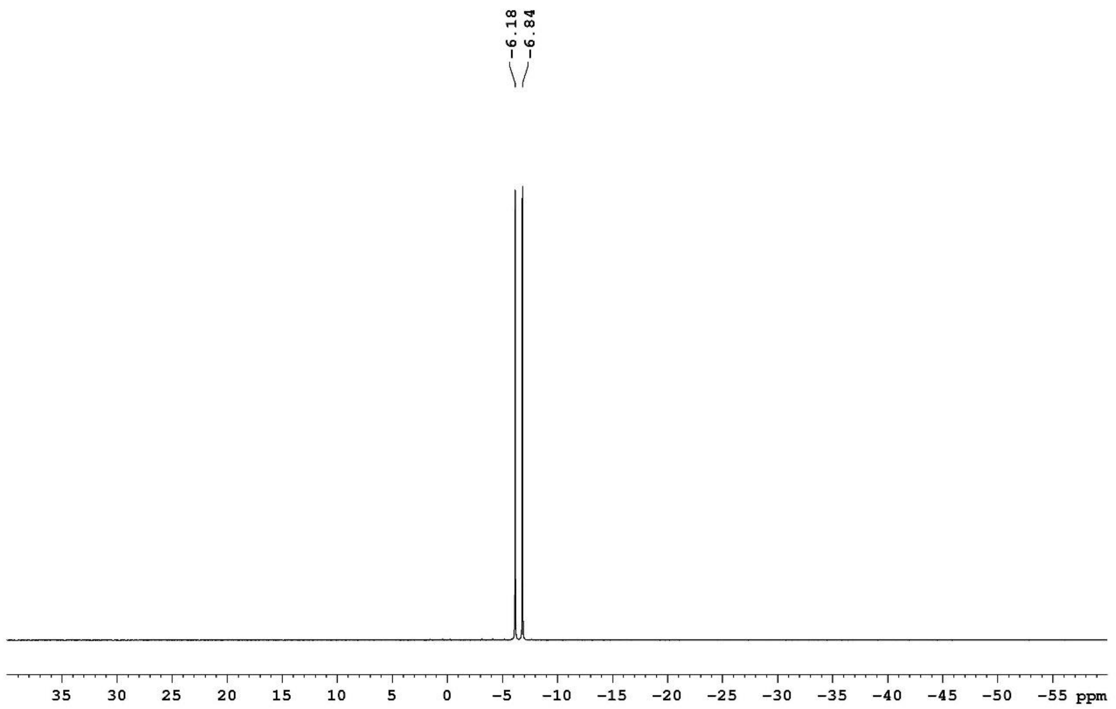

Figure S77. $^{31}P\{^{1}H\}$ NMR (202.5 MHz, C_6D_6, 298 K) spectrum of **2e**.

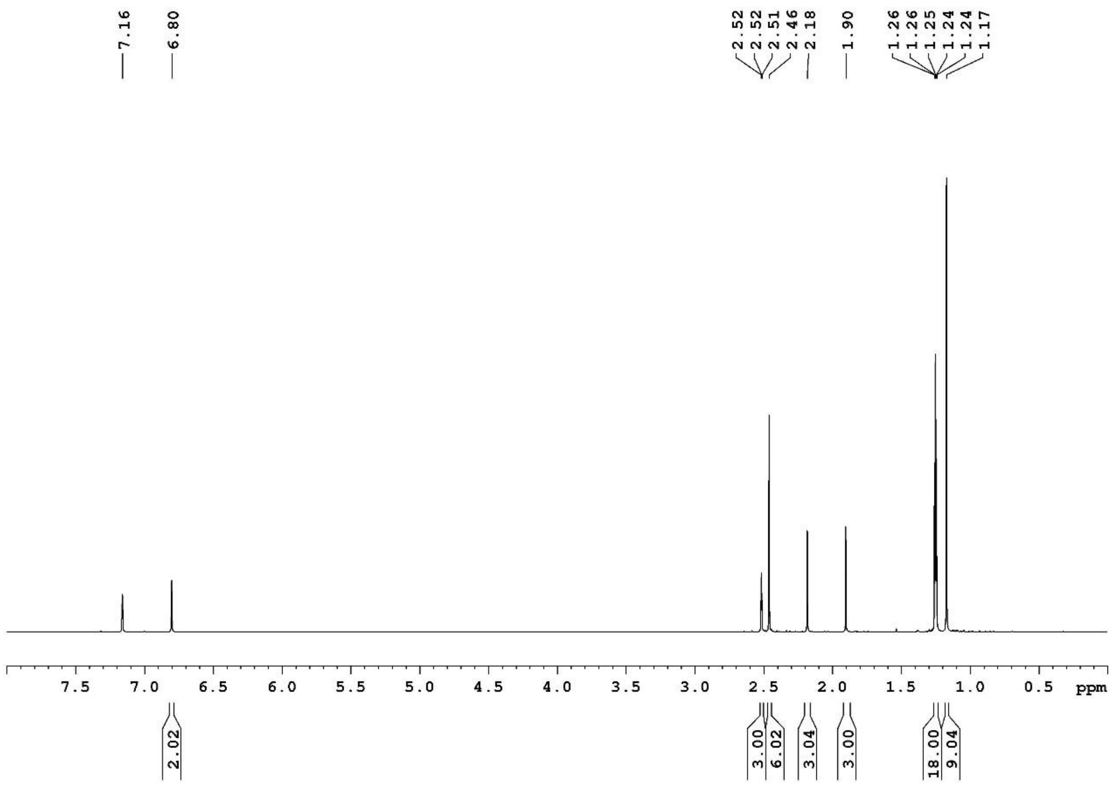

Figure S78. ^{1}H NMR (500.1 MHz, C_6D_6, 298 K) spectrum of **2f**.

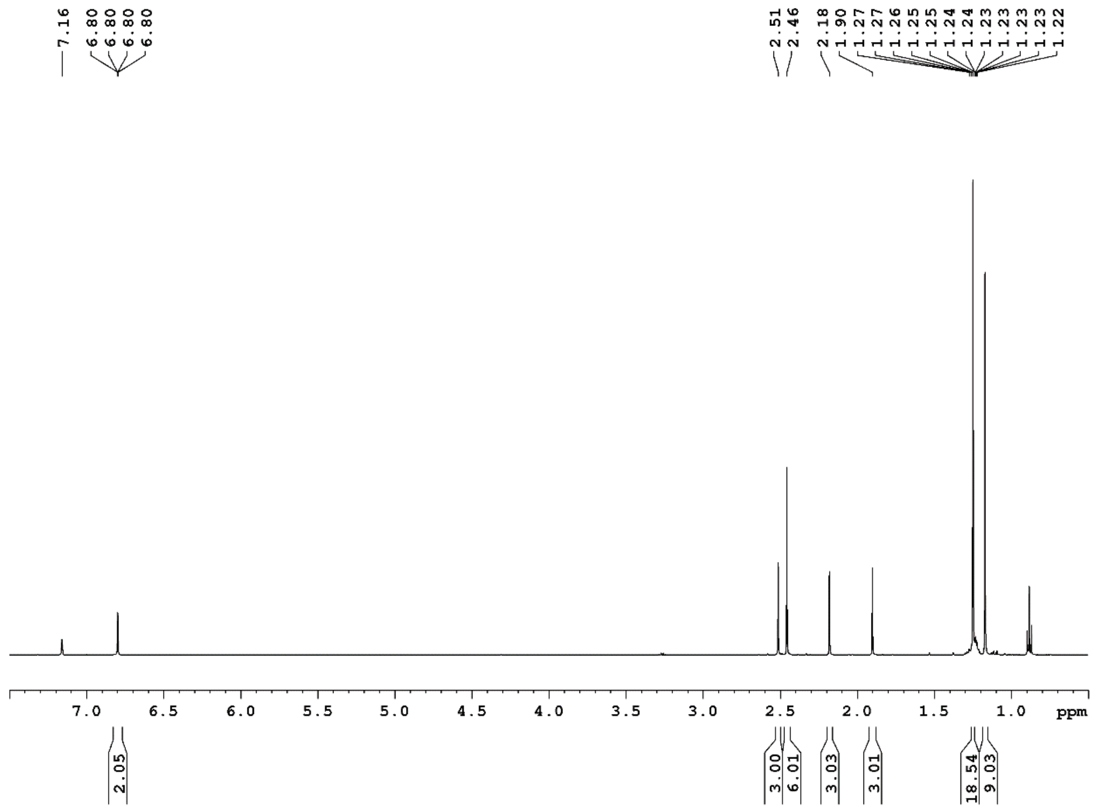

Figure S79. $^{1}H\{^{31}P\}$ NMR (500.1 MHz, C_6D_6, 298 K) spectrum of **2f**.

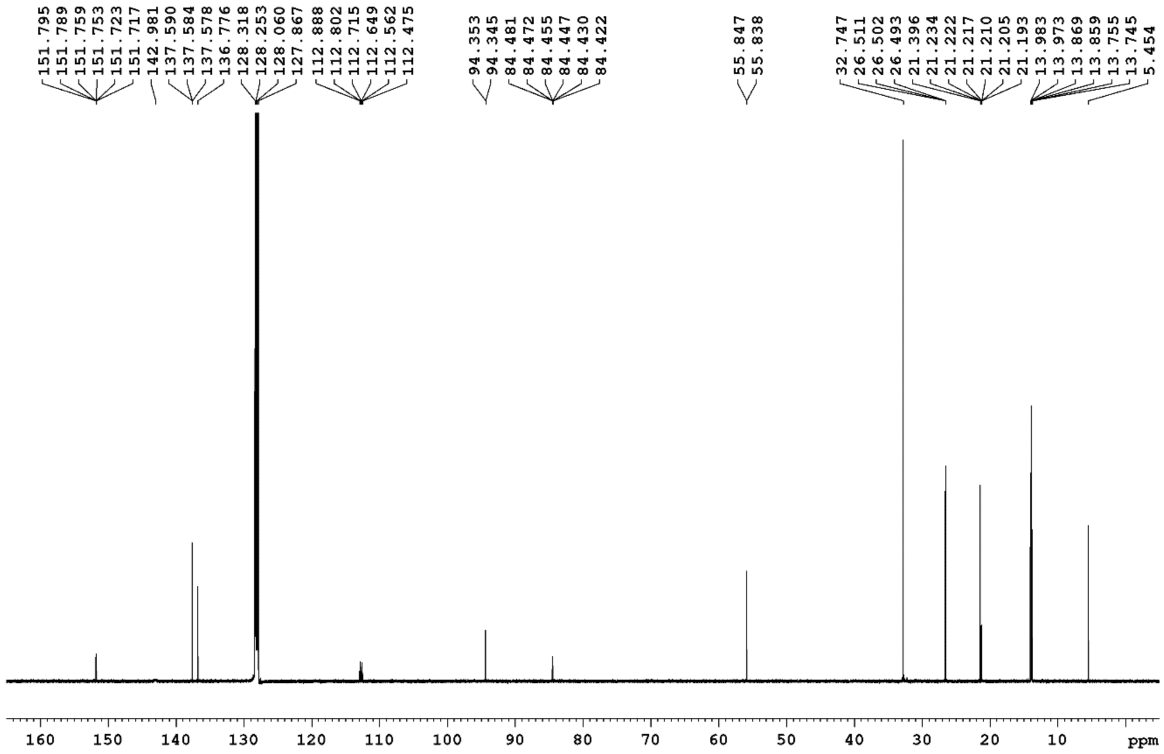

Figure S80. $^{13}C\{^{1}H\}$ NMR (125.8 MHz, C_6D_6, 298 K) spectrum of **2f**.

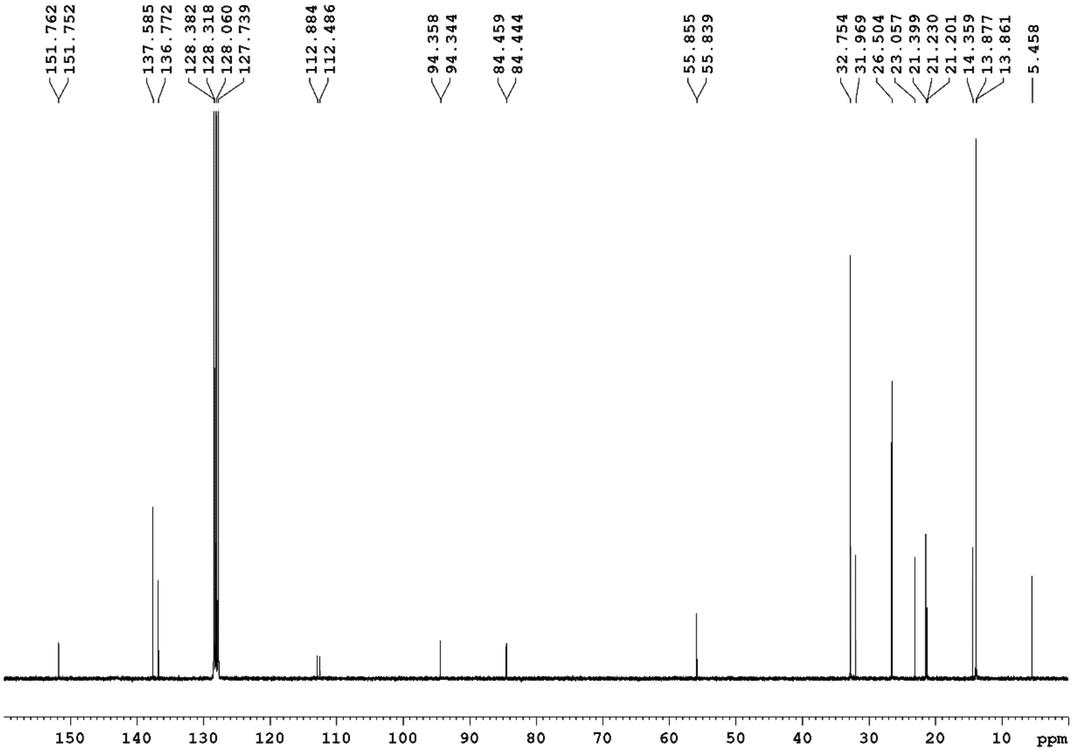

Figure S81. $^{13}C\{^{1}H, ^{31}P\}$ NMR (75.5 MHz, C_6D_6, 298 K) spectrum of **2f**.

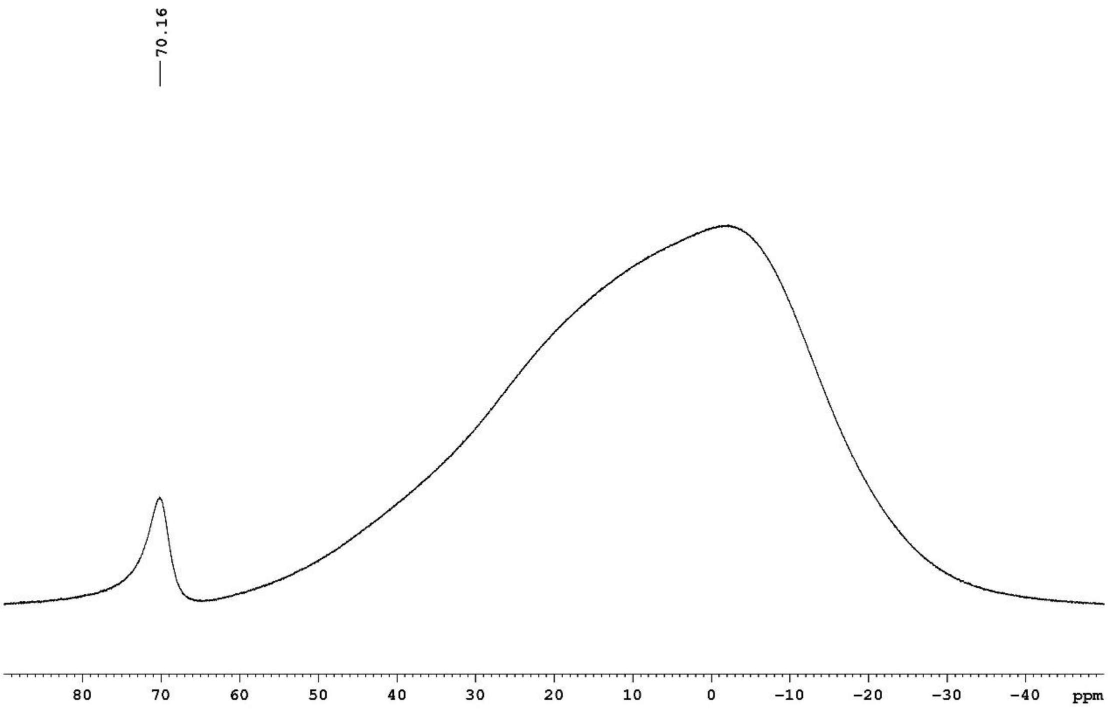

Figure S82. ^{11}B NMR (160.5 MHz, C_6D_6, 298 K) spectrum of **2f**.

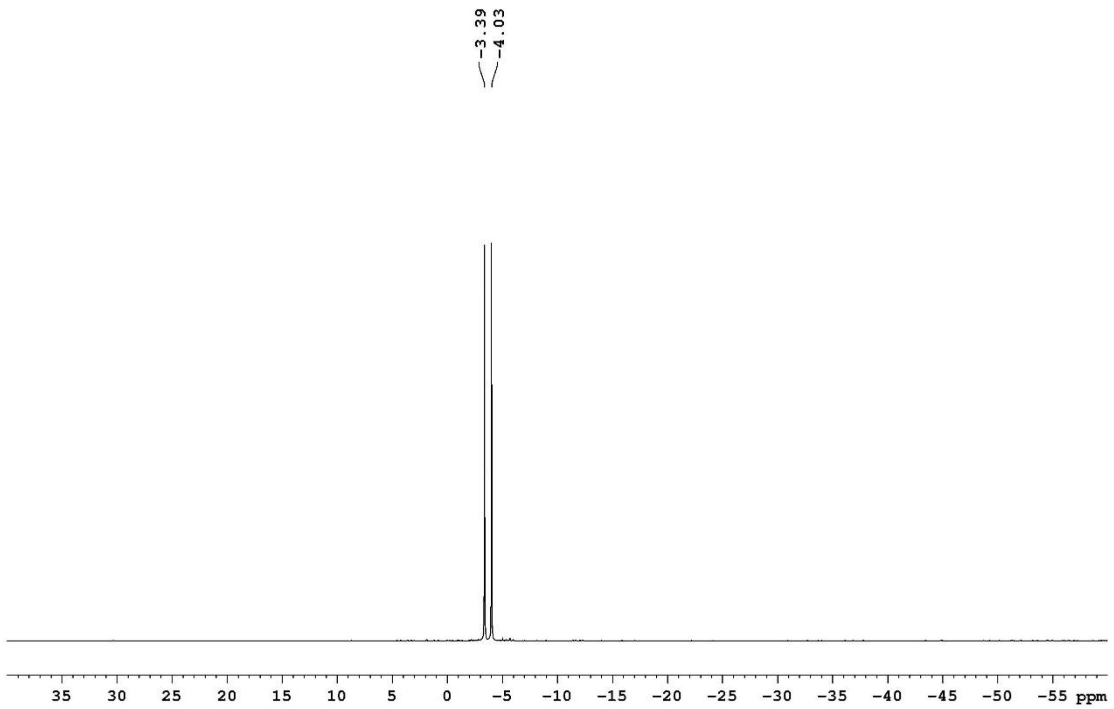

Figure S83. $^{31}P\{^{1}H\}$ NMR (202.5 MHz, C_6D_6, 298 K) spectrum of **2f**.

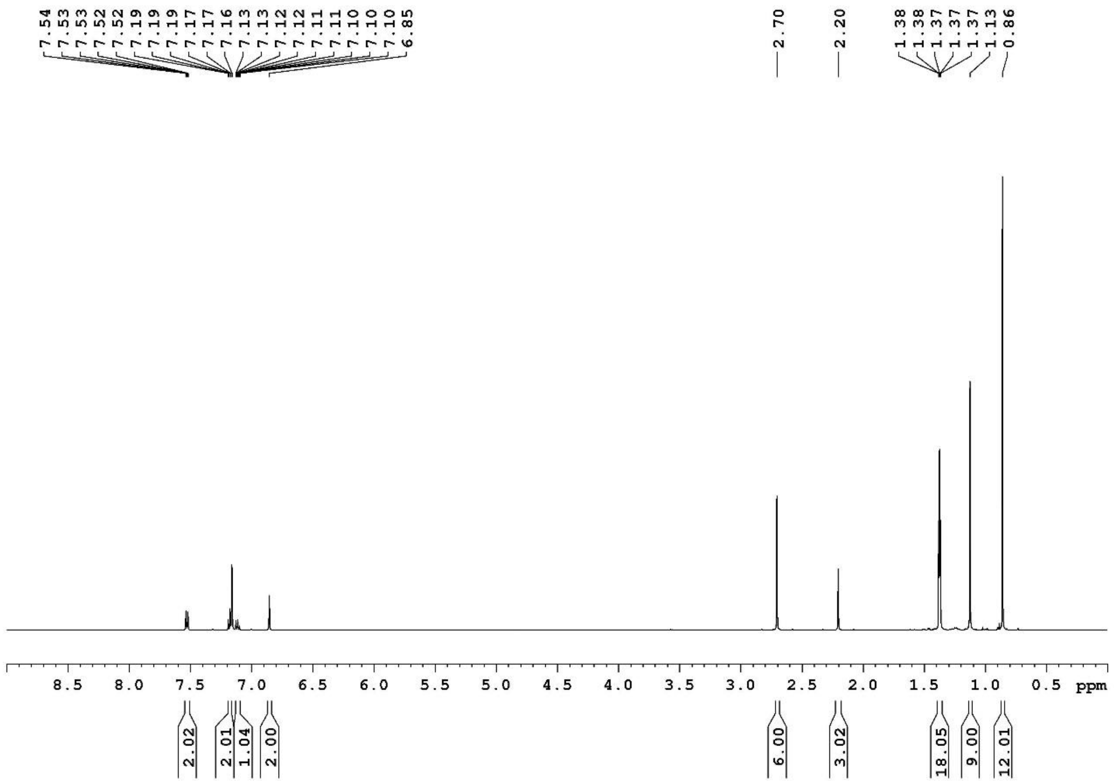

Figure S84. ^{1}H NMR (500.1 MHz, C_6D_6, 298 K) spectrum of **2g**.

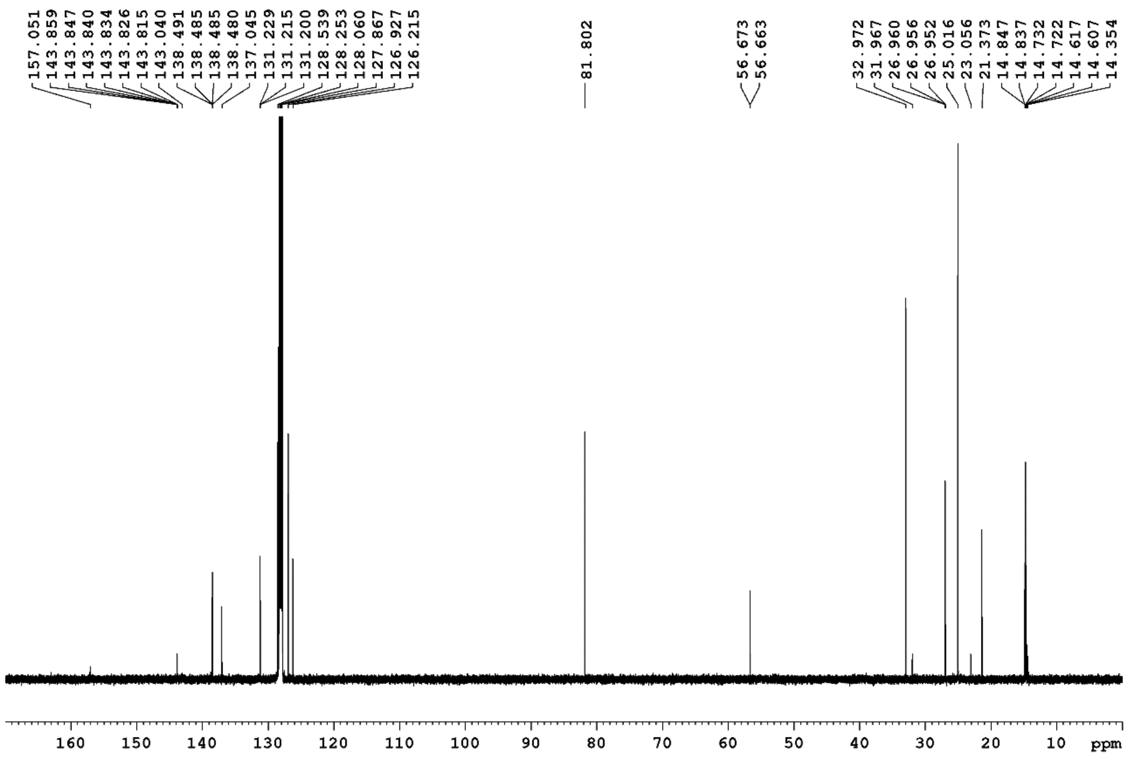

Figure S85. $^{13}C\{^{1}H\}$ NMR (125.8 MHz, C_6D_6, 298 K) spectrum of **2g**.

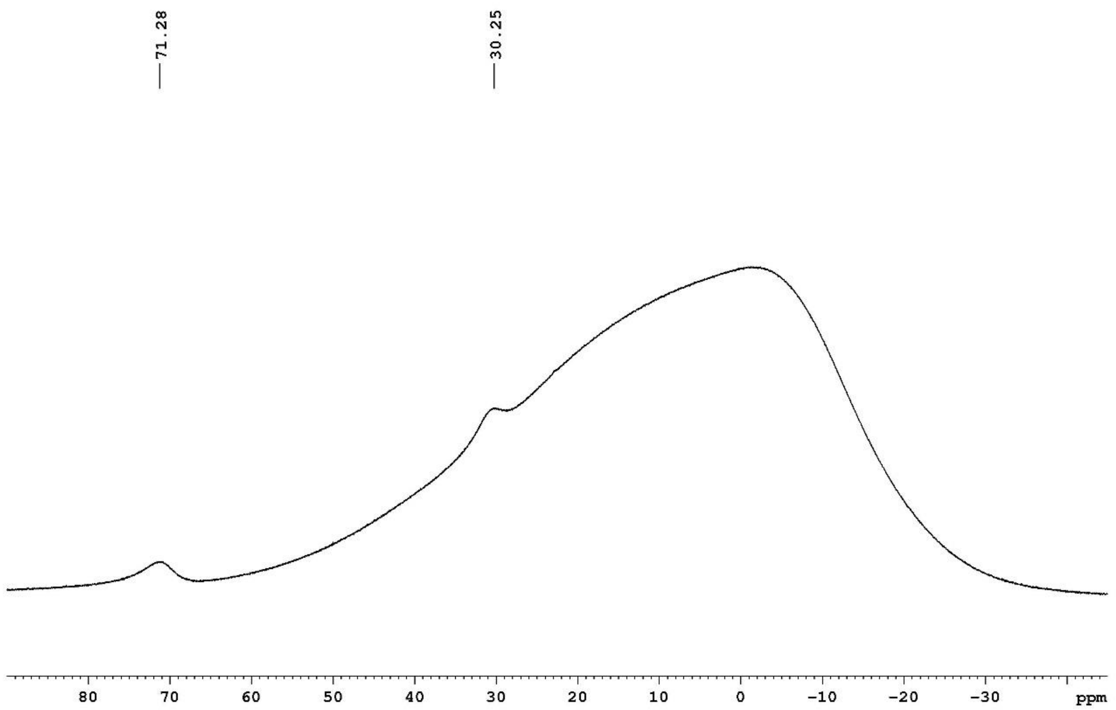

Figure S86. ^{11}B NMR (160.5 MHz, C_6D_6, 298 K) spectrum of **2g**.

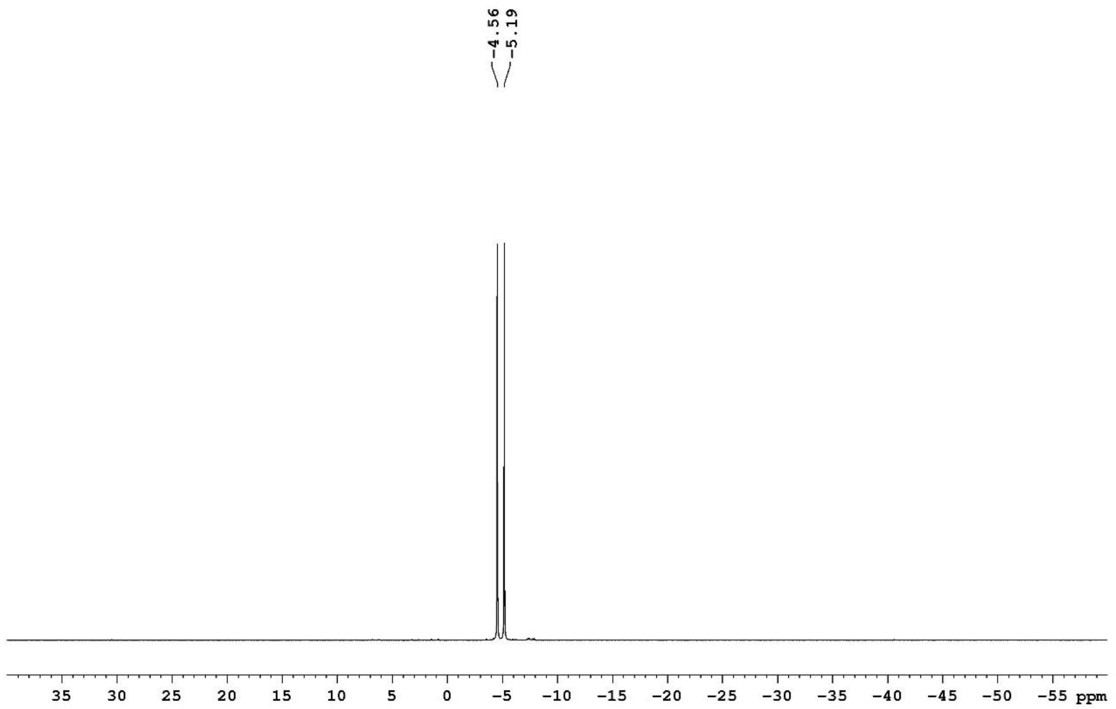

Figure S87. $^{31}P\{^{1}H\}$ NMR (202.5 MHz, C_6D_6, 298 K) spectrum of **2g**.

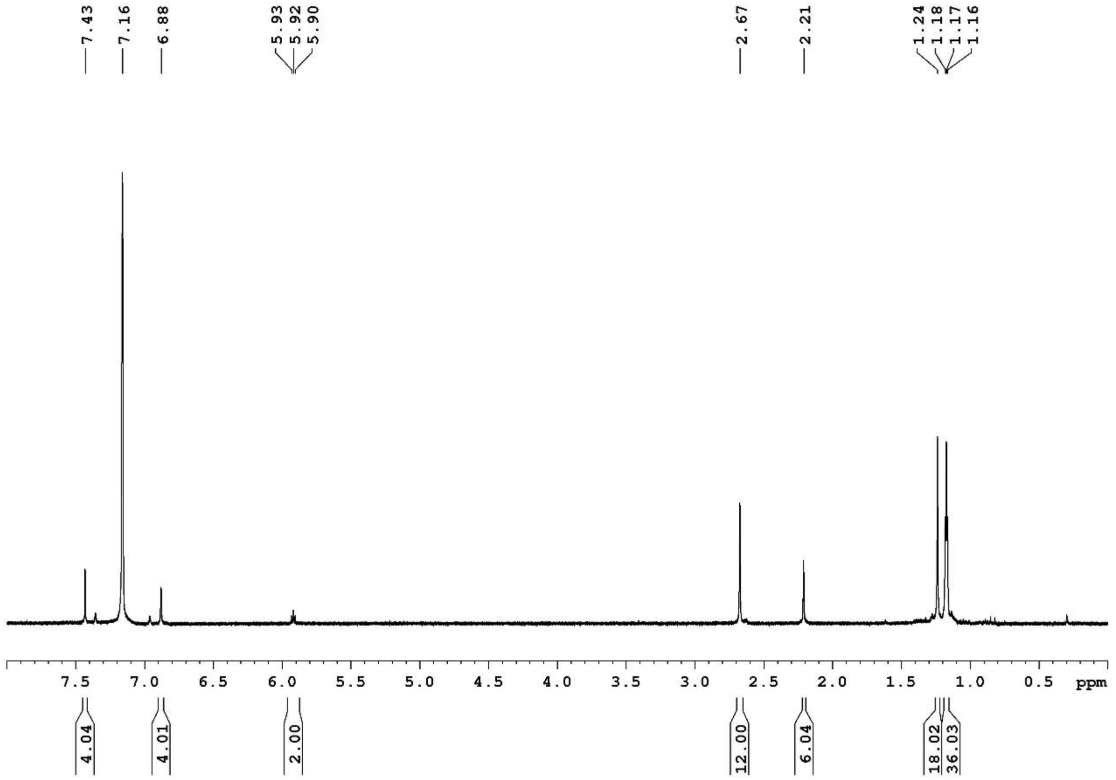

Figure S88. ^{1}H NMR (400.6 MHz, C_6D_6, 298 K) spectrum of **2l**.

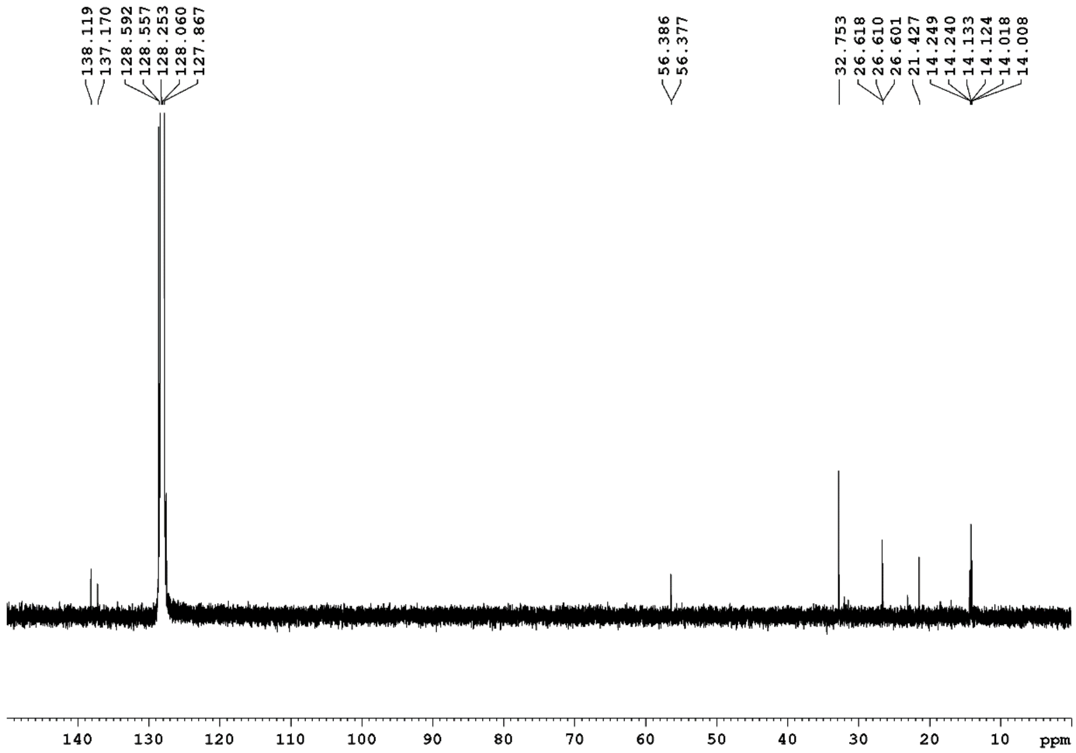

Figure S89. $^{13}C\{^{1}H\}$ NMR (125.8 MHz, C_6D_6, 298 K) spectrum of **2l**.

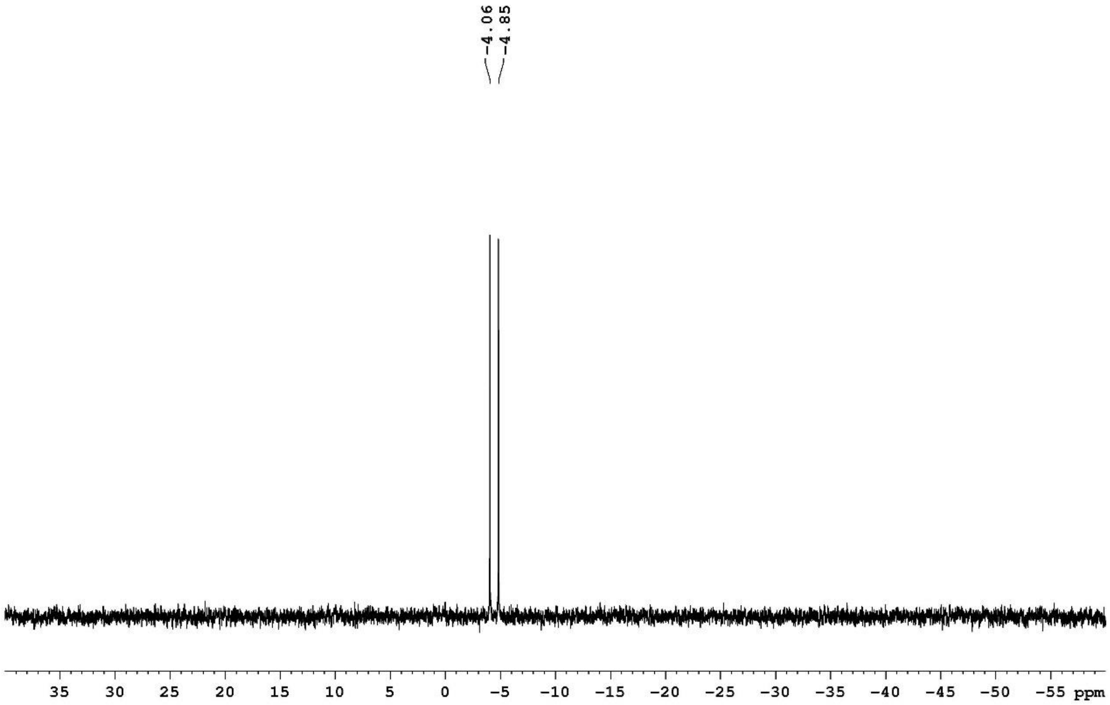

Figure S90. $^{31}P\{^{1}H\}$ NMR (162.2 MHz, C_6D_6, 298 K) spectrum of **2l**.

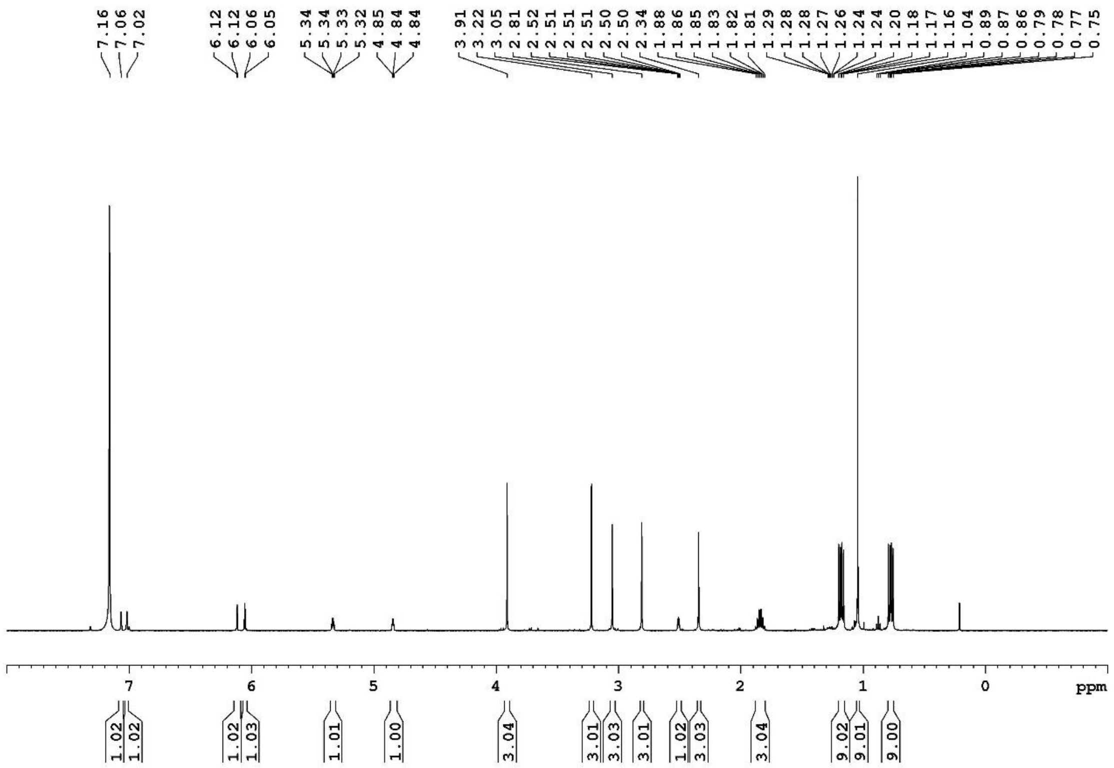

Figure S91. ^{1}H NMR (500.1 MHz, C_6D_6, 298 K) spectrum of **3a^{Me}**.

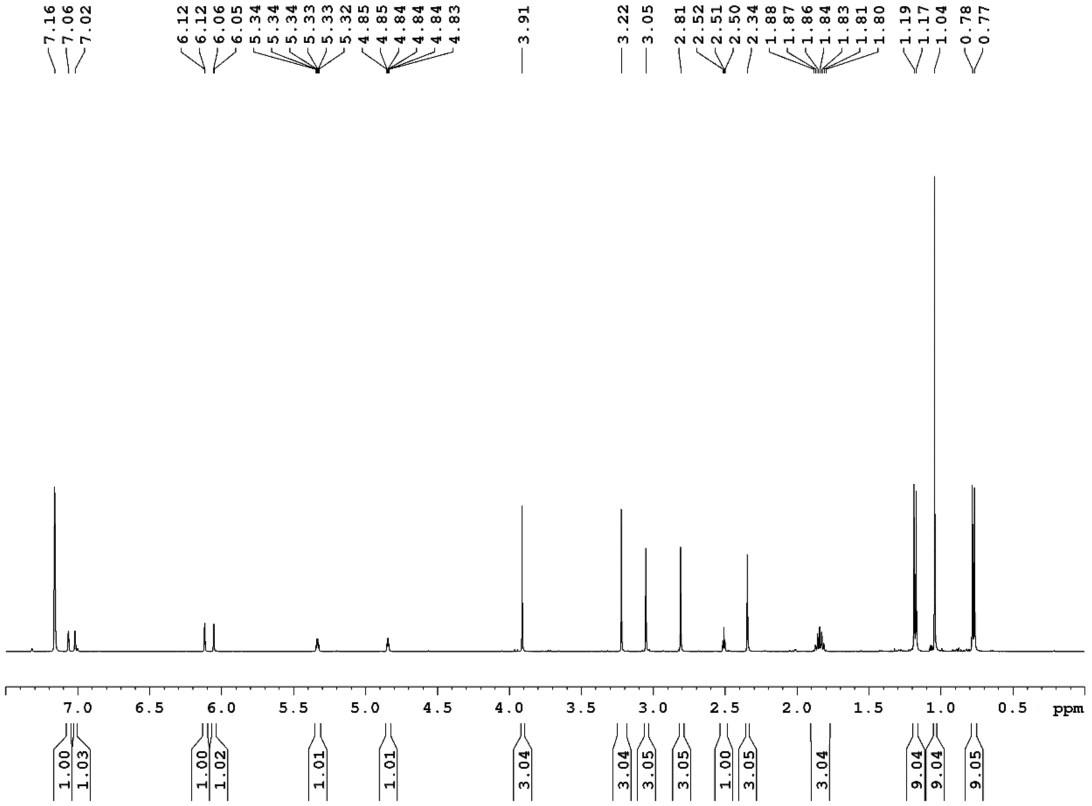

Figure S92. ^{1}H{^{31}P} NMR (500.1 MHz, C_6D_6, 298 K) spectrum of **3a^{Me}**.

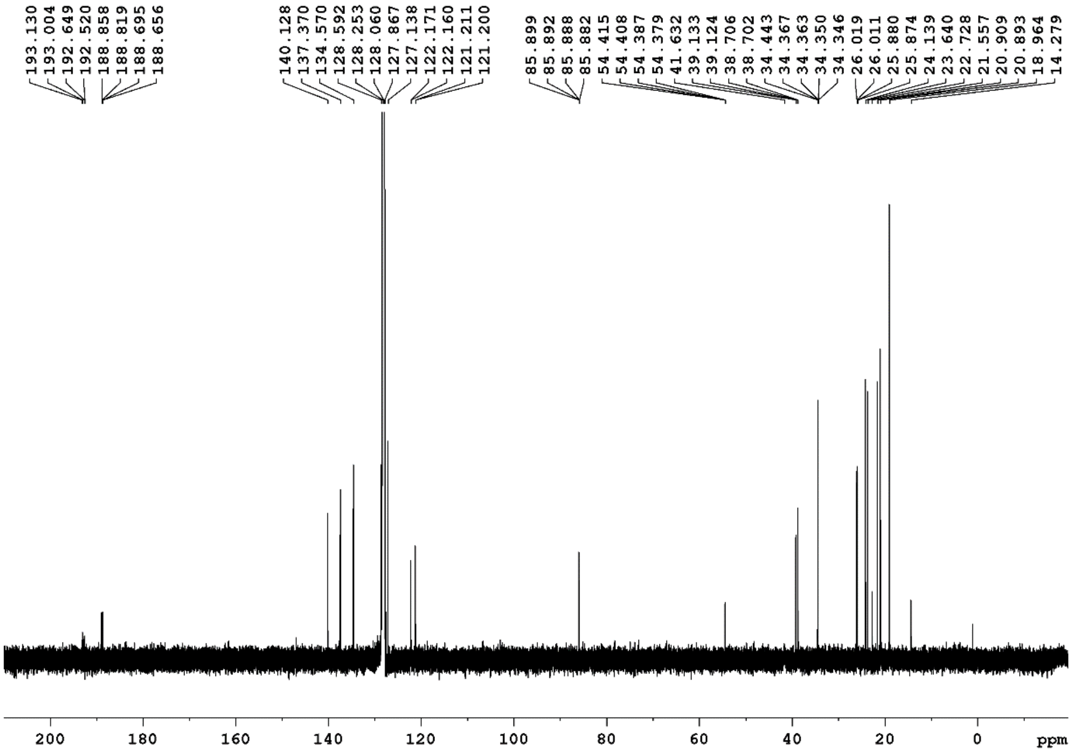

Figure S93. $^{13}C\{^{1}H\}$ NMR (125.8 MHz, C_6D_6, 298 K) spectrum of $\mathbf{3a^{Me}}$.

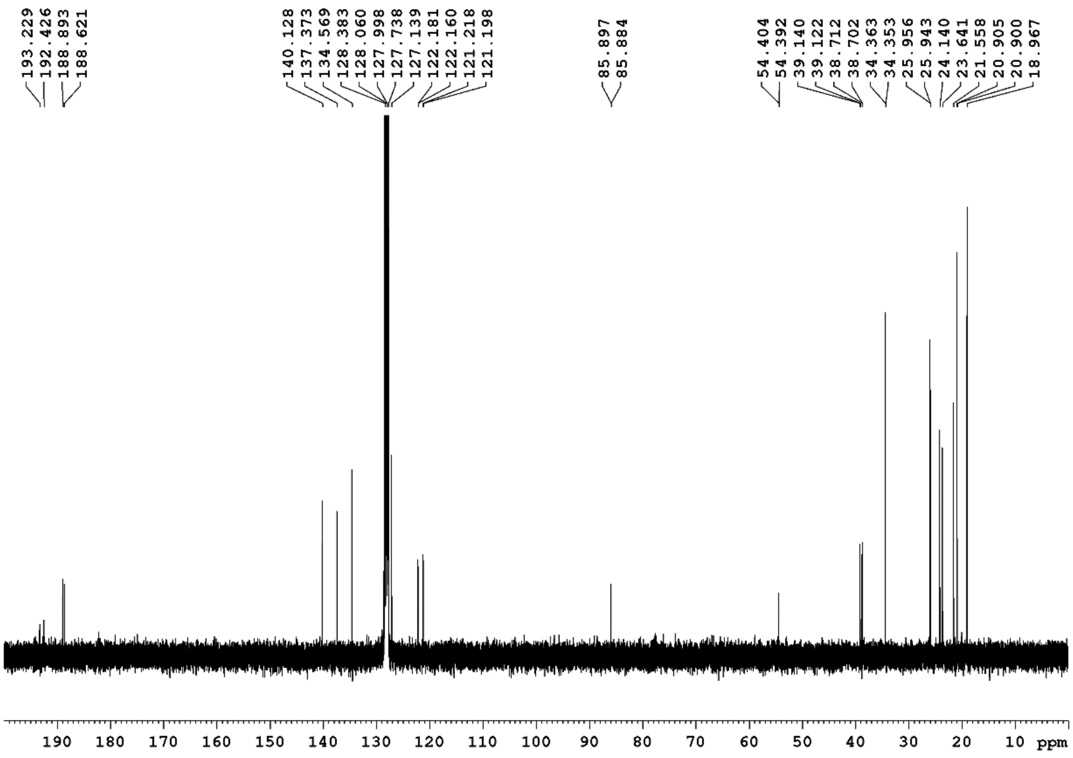

Figure S94. $^{13}C\{^{1}H, ^{31}P\}$ NMR (75.5 MHz, C_6D_6, 298 K) spectrum of $\mathbf{3a^{Me}}$.

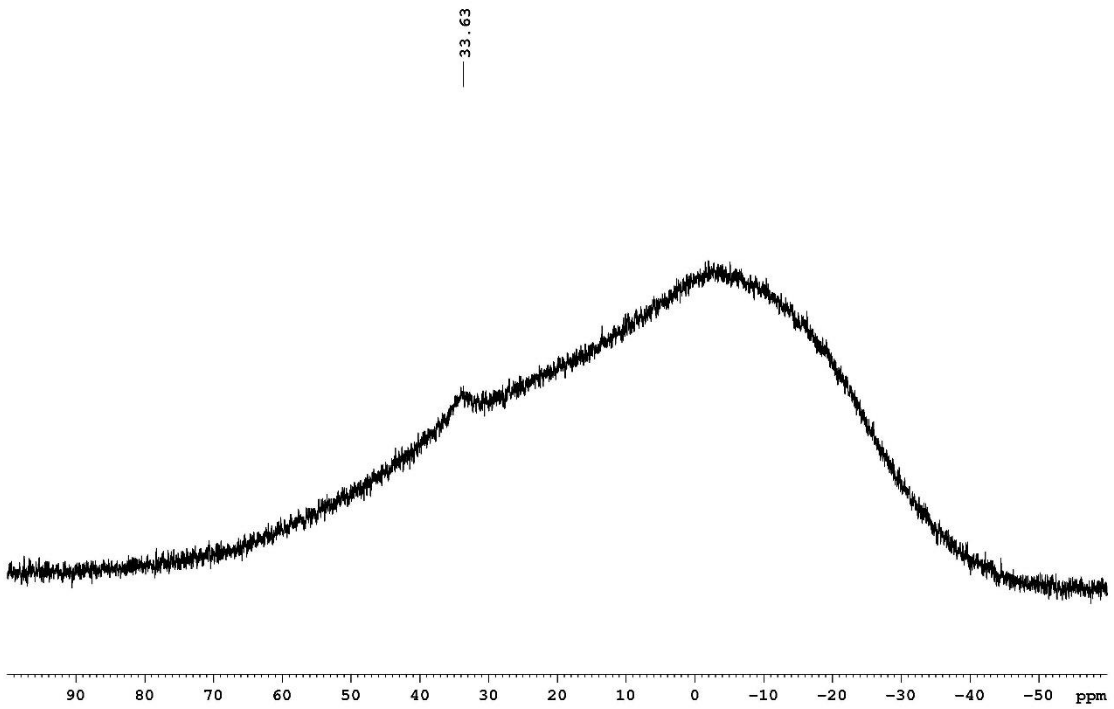

Figure S95. ^{11}B NMR (128.5 MHz, C_6D_6, 298 K) spectrum of **3a^{Me}**.

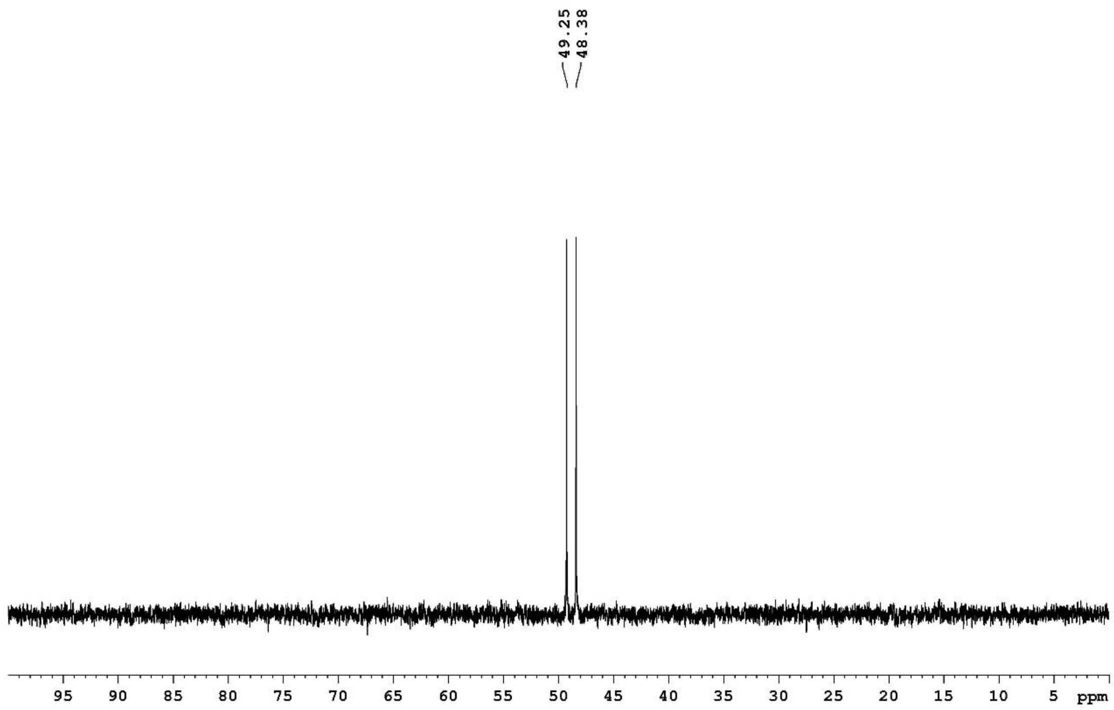

Figure S96. $^{31}P\{^1H\}$ NMR (162.2 MHz, C_6D_6, 298 K) spectrum of **3a^{Me}**.

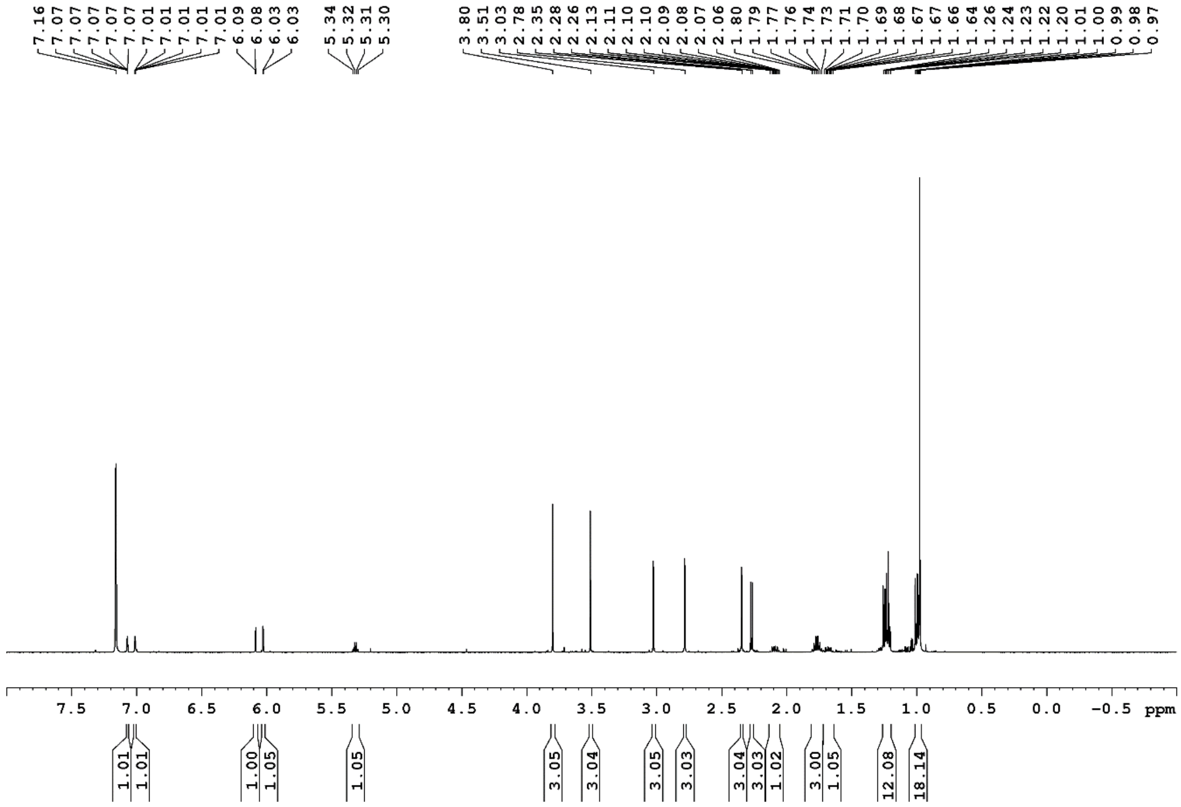

Figure S97. ^{1}H NMR (500.1 MHz, C_6D_6, 298 K) spectrum of **3e^{Me}**.

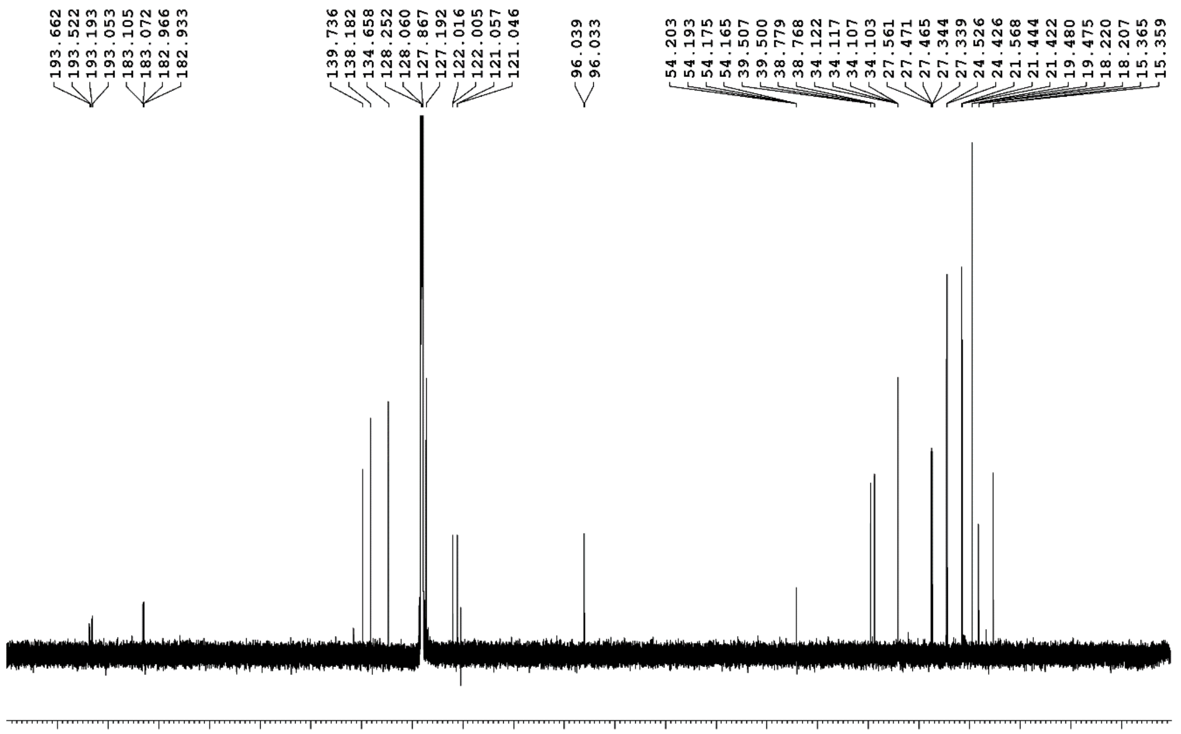

Figure S98. ^{13}C{^{1}H} NMR (125.8 MHz, C_6D_6, 298 K) spectrum of **3e^{Me}**.

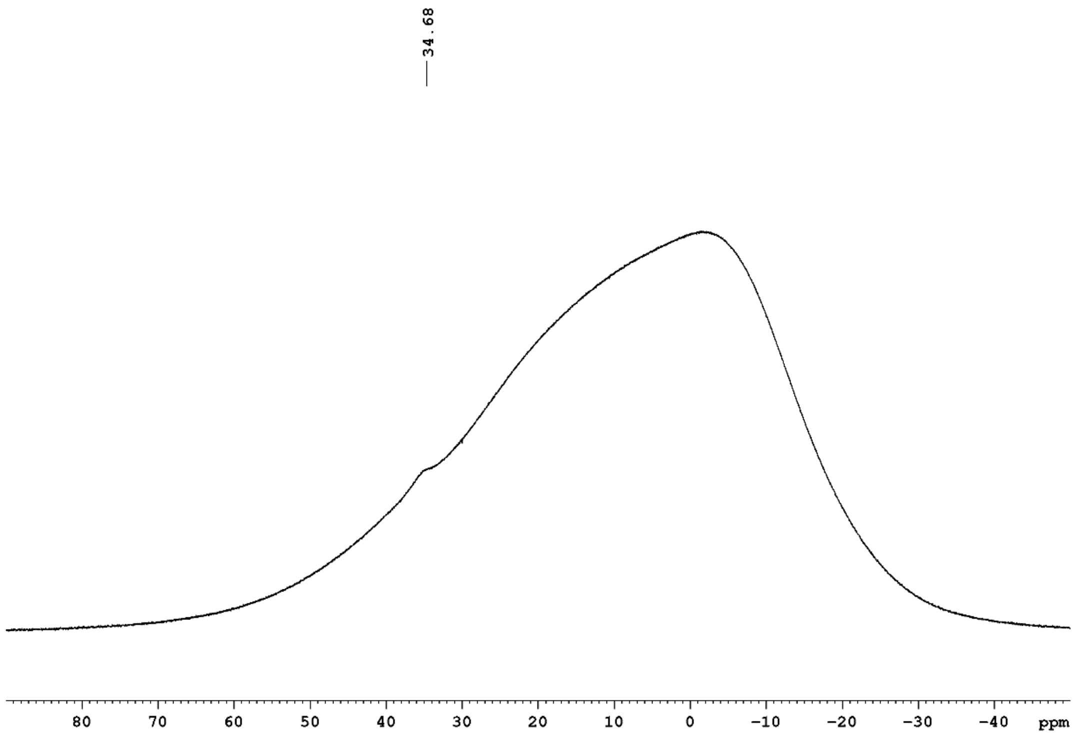

Figure S99. ^{11}B NMR (160.5 MHz, C_6D_6, 298 K) spectrum of $\mathbf{3e^{Me}}$.

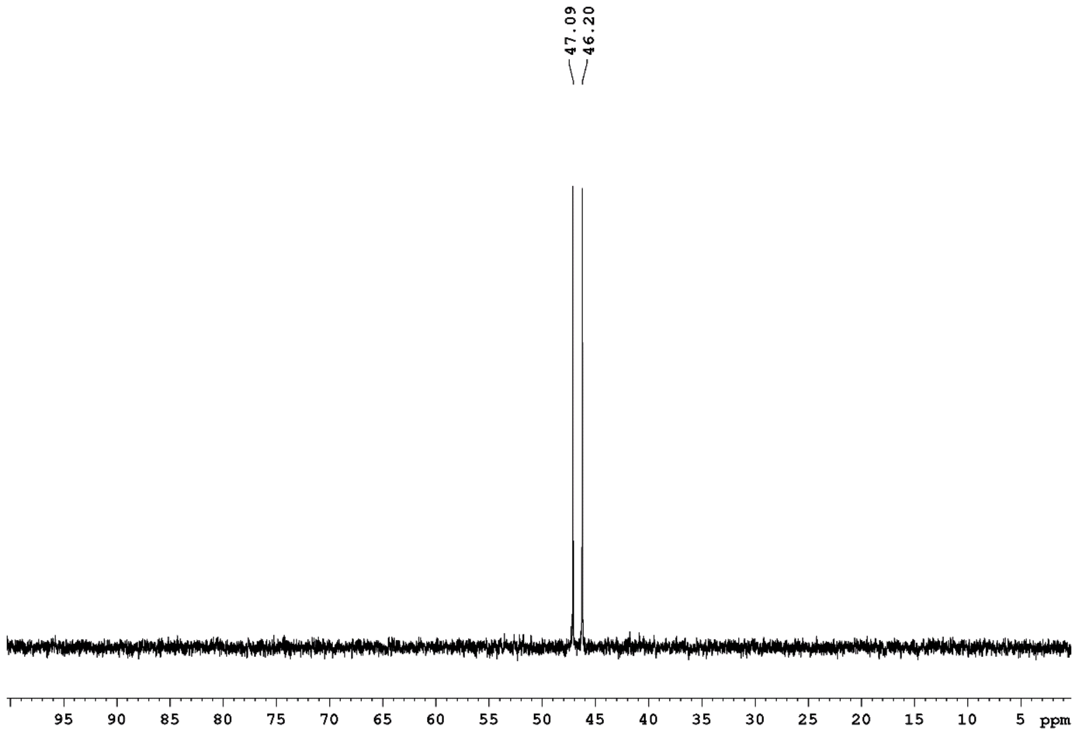

Figure S100. $^{31}P\{^1H\}$ NMR (162.2 MHz, C_6D_6, 298 K) spectrum of $\mathbf{3e^{Me}}$.

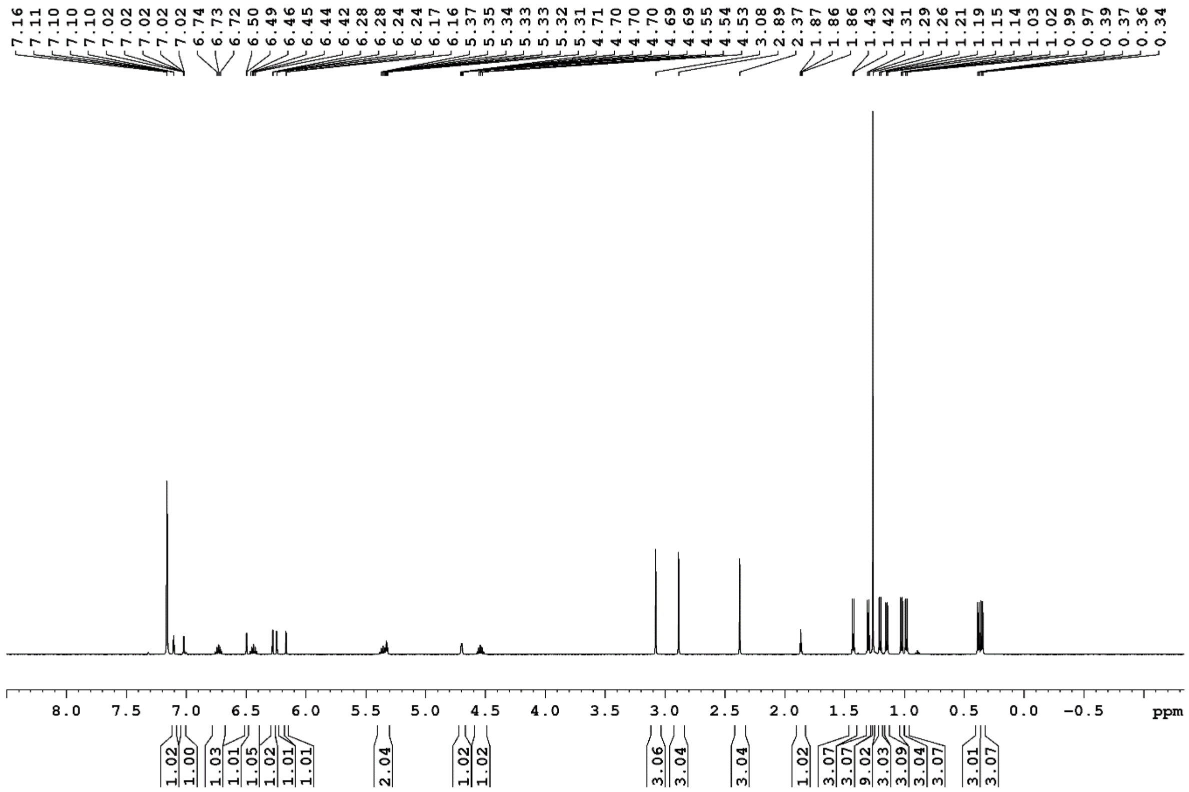

Figure S101. ^{1}H NMR (500.1 MHz, C_6D_6, 298 K) spectrum of **4a*iPr***.

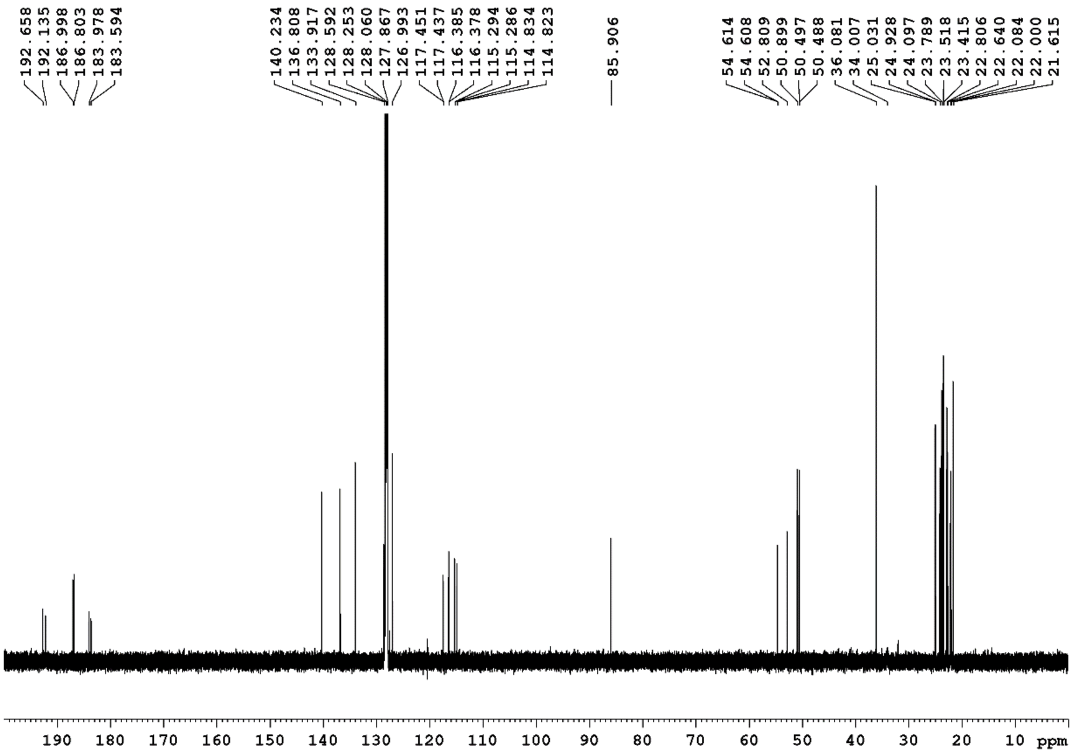

Figure S102. $^{13}C\{^{1}H\}$ NMR (125.8 MHz, C_6D_6, 298 K) spectrum of **4a*iPr***.

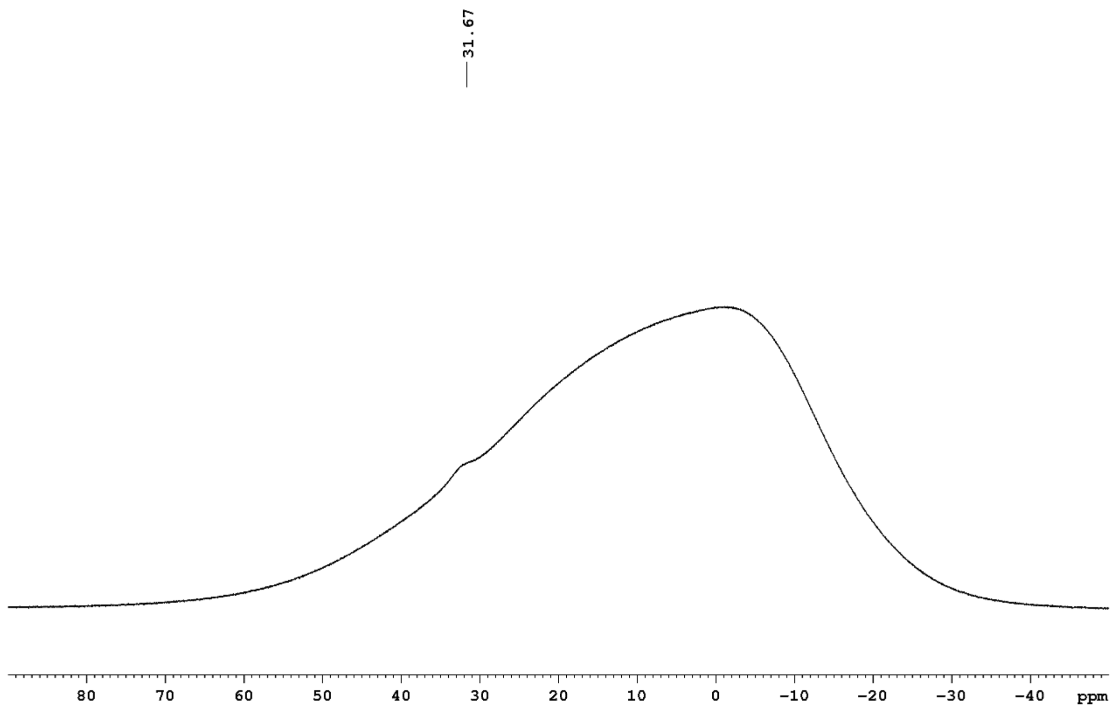

Figure S103. ^{11}B NMR (160.5 MHz, C_6D_6, 298 K) spectrum of **4a^{iPr}**.

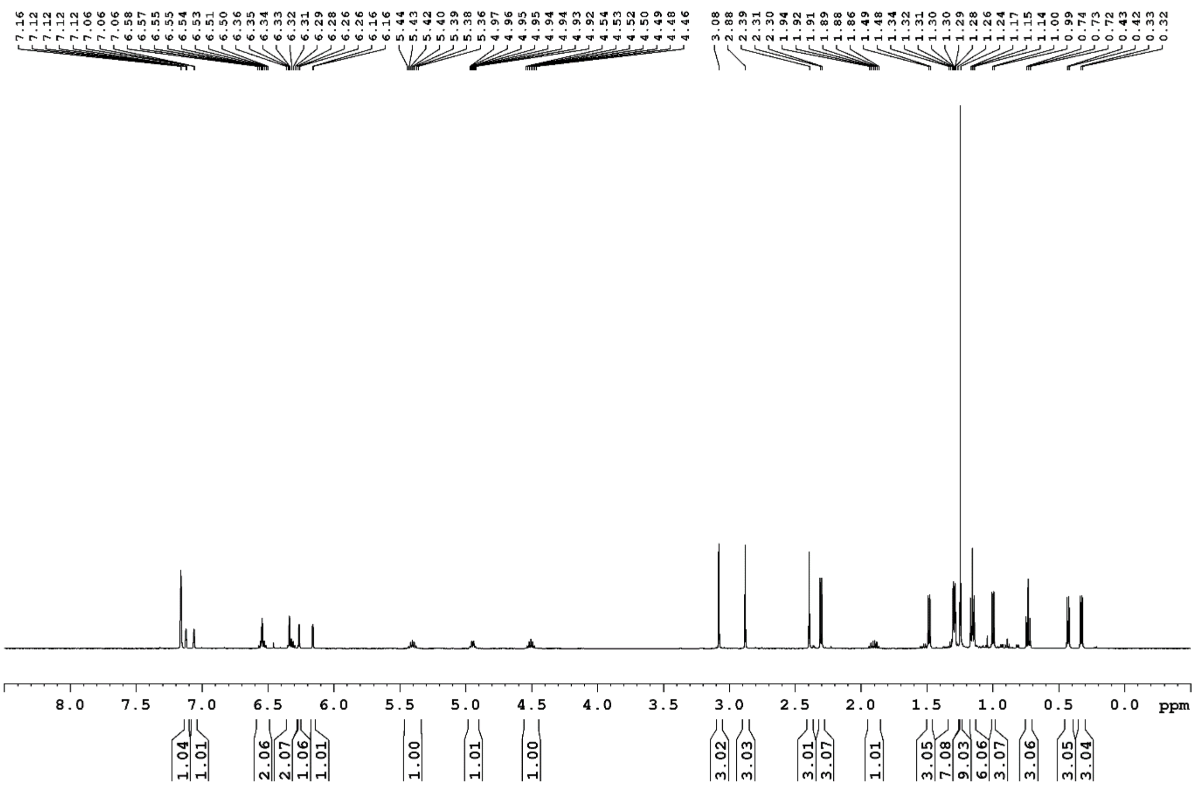

Figure S104. ^{1}H NMR (500.1 MHz, C_6D_6, 298 K) spectrum of **4e^{iPr}**.

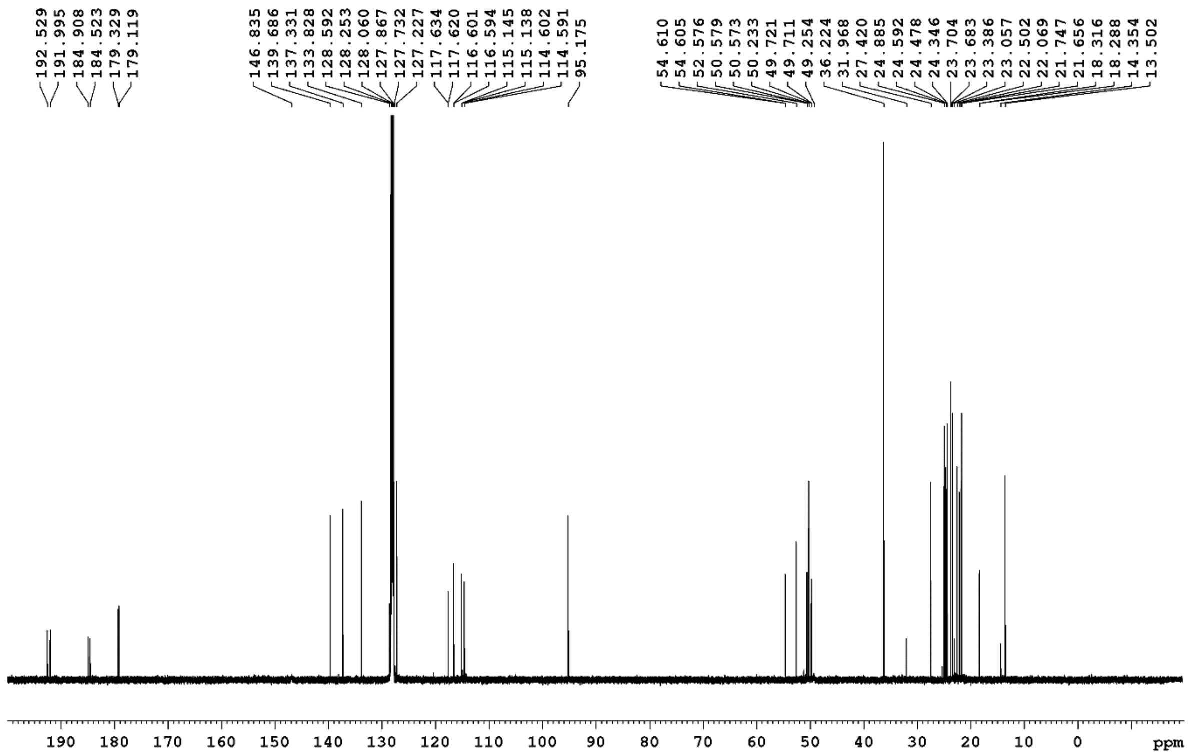

Figure S105. $^{13}C\{^{1}H\}$ NMR (125.8 MHz, C_6D_6, 298 K) spectrum of **4e**$^{\mathbf{\mathit{i}Pr}}$.

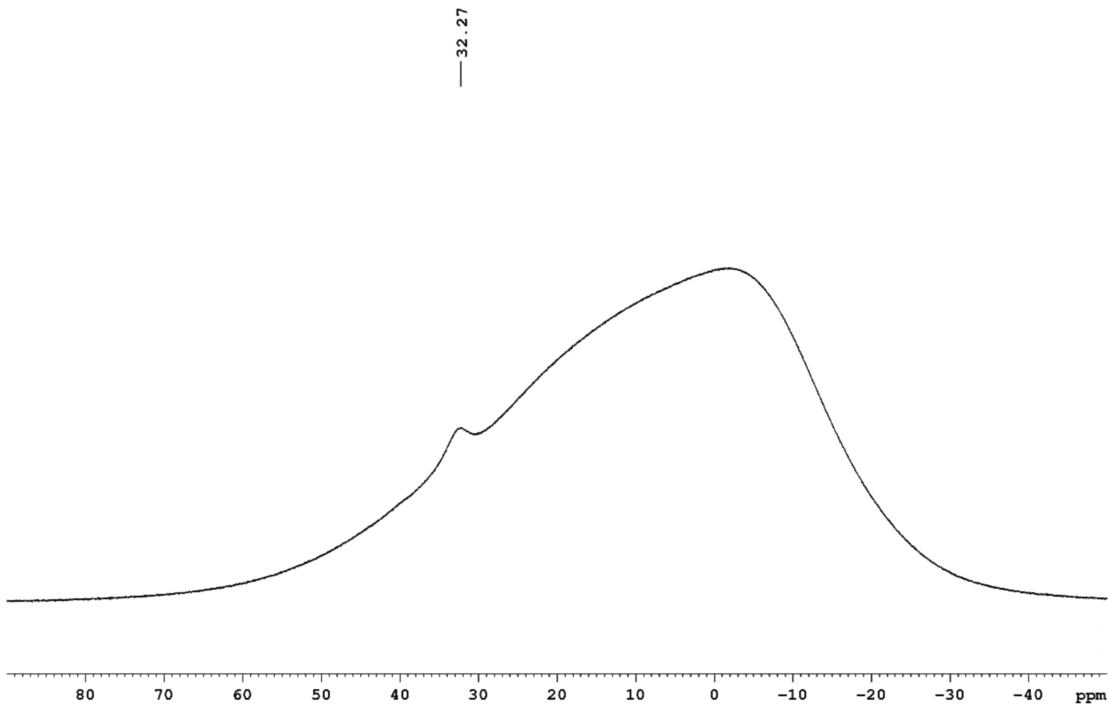

Figure S106. ^{11}B NMR (160.5 MHz, C_6D_6, 298 K) spectrum of **4e**$^{\mathbf{\mathit{i}Pr}}$.

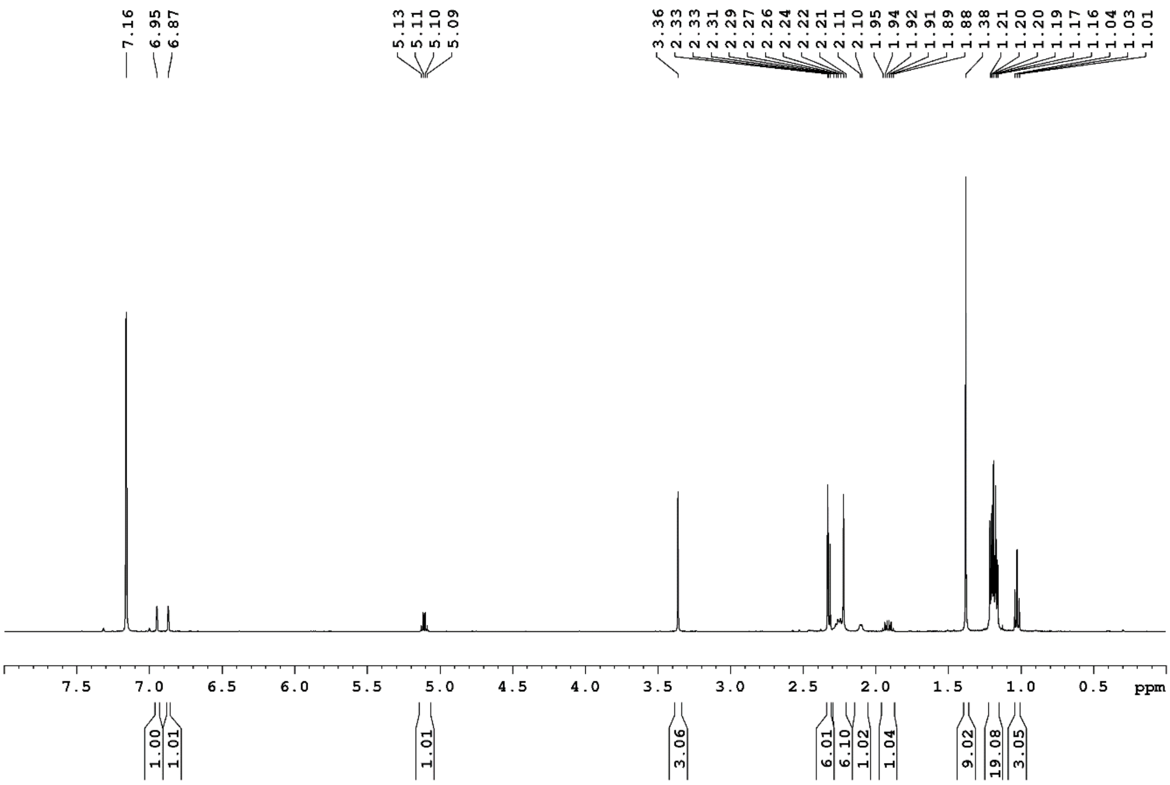

Figure S107. ^{1}H NMR (500.1 MHz, C_6D_6, 298 K) spectrum of **5**.

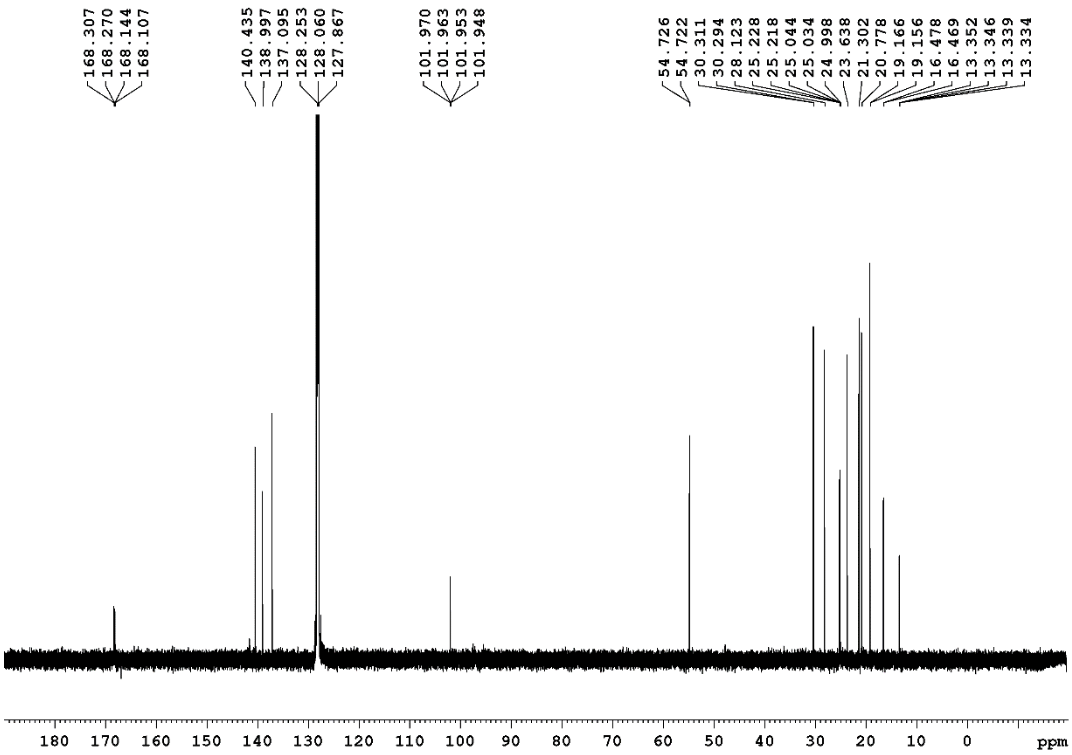

Figure S108. $^{13}C\{^{1}H\}$ NMR (125.8 MHz, C_6D_6, 298 K) spectrum of **5**.

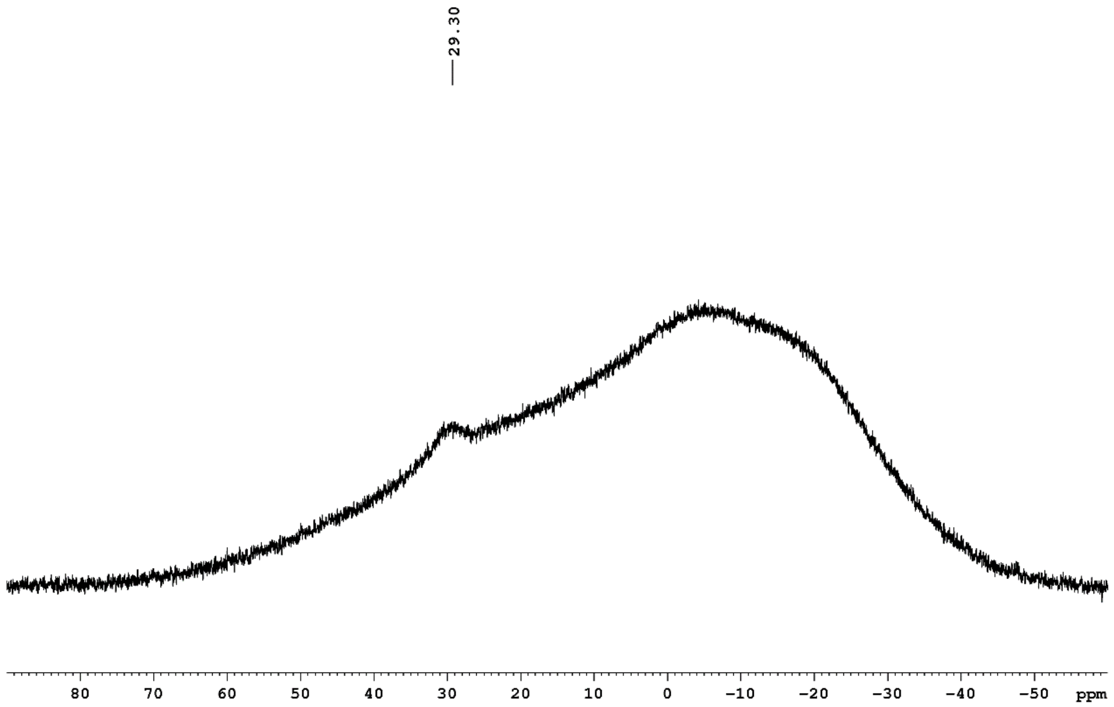

Figure S109. ^{11}B NMR (128.5 MHz, C_6D_6, 298 K) spectrum of **5**.

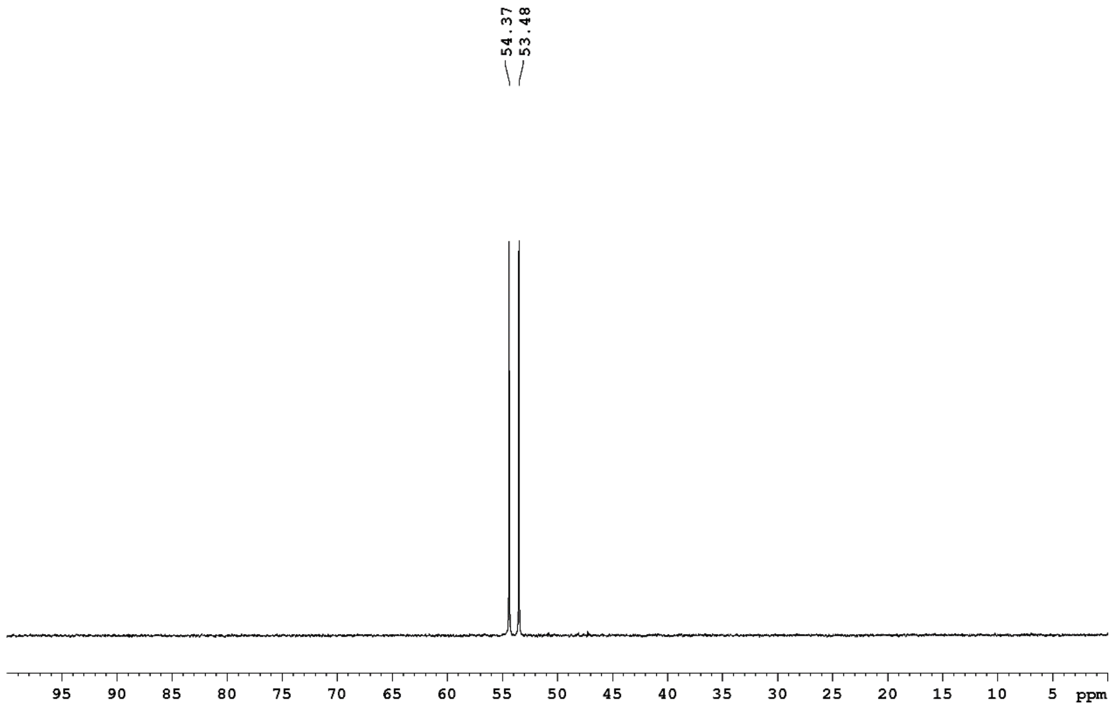

Figure S110. $^{31}P\{^1H\}$ NMR (202.5 MHz, C_6D_6, 298 K) spectrum of **5**.

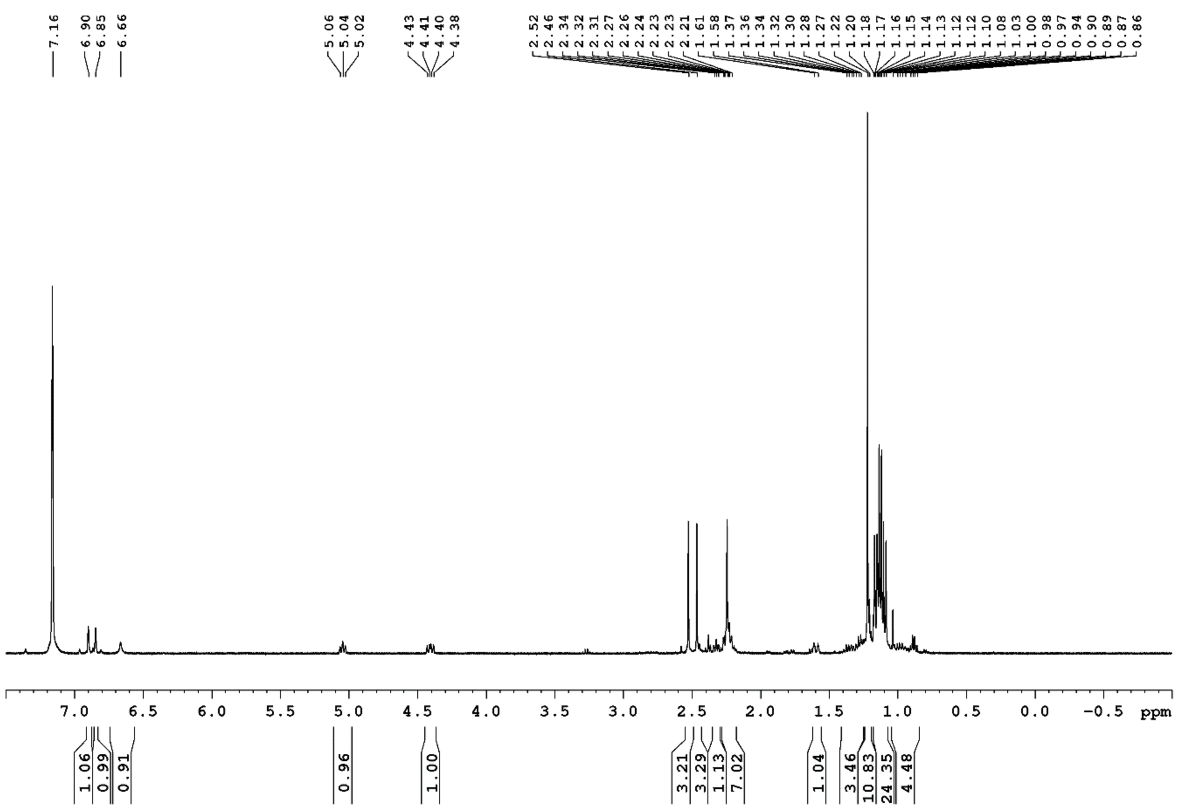

Figure S111. ^{1}H NMR (400.6 MHz, C_6D_6, 298 K) spectrum of **7** with impurities.

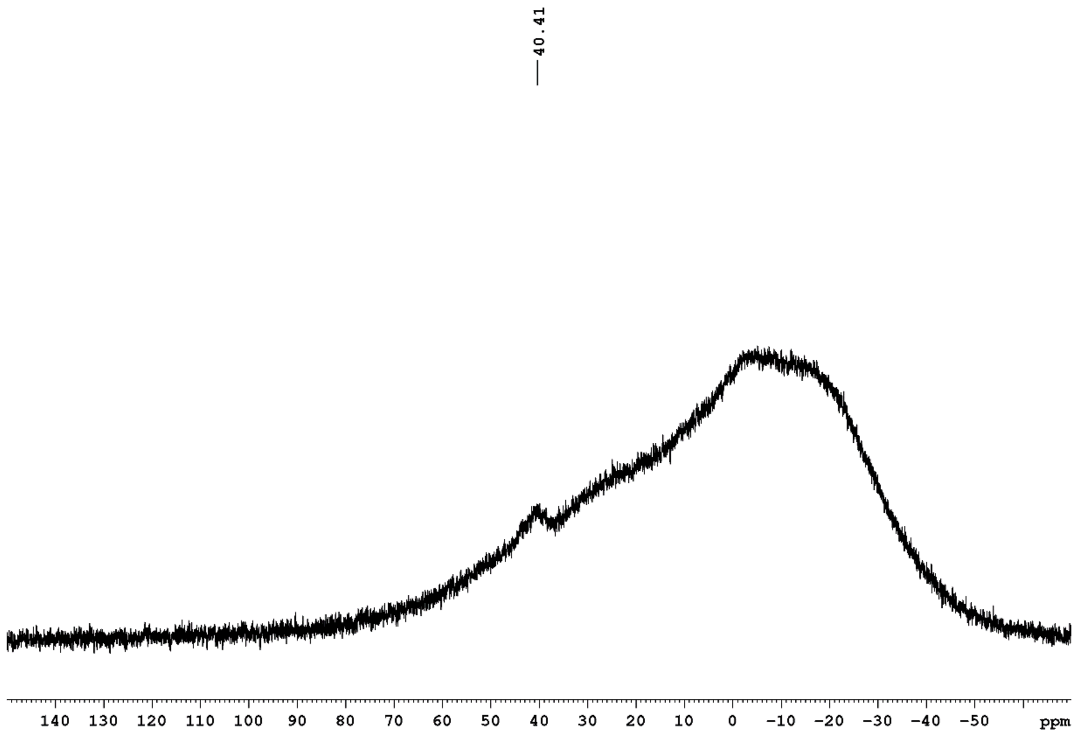

Figure S 112. ^{11}B NMR (128.5 MHz, C_6D_6, 298 K) spectrum of **7**.

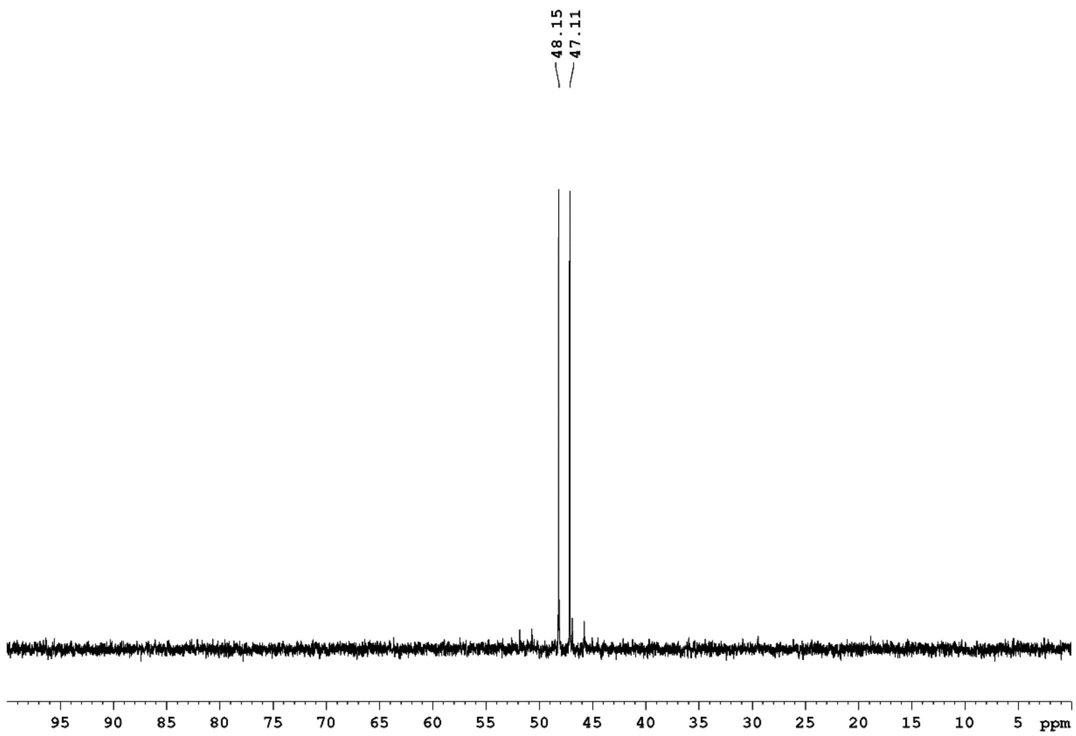

Figure S113. $^{31}P\{^{1}H\}$ NMR (162.2 MHz, C_6D_6, 298 K) spectrum of **7**.

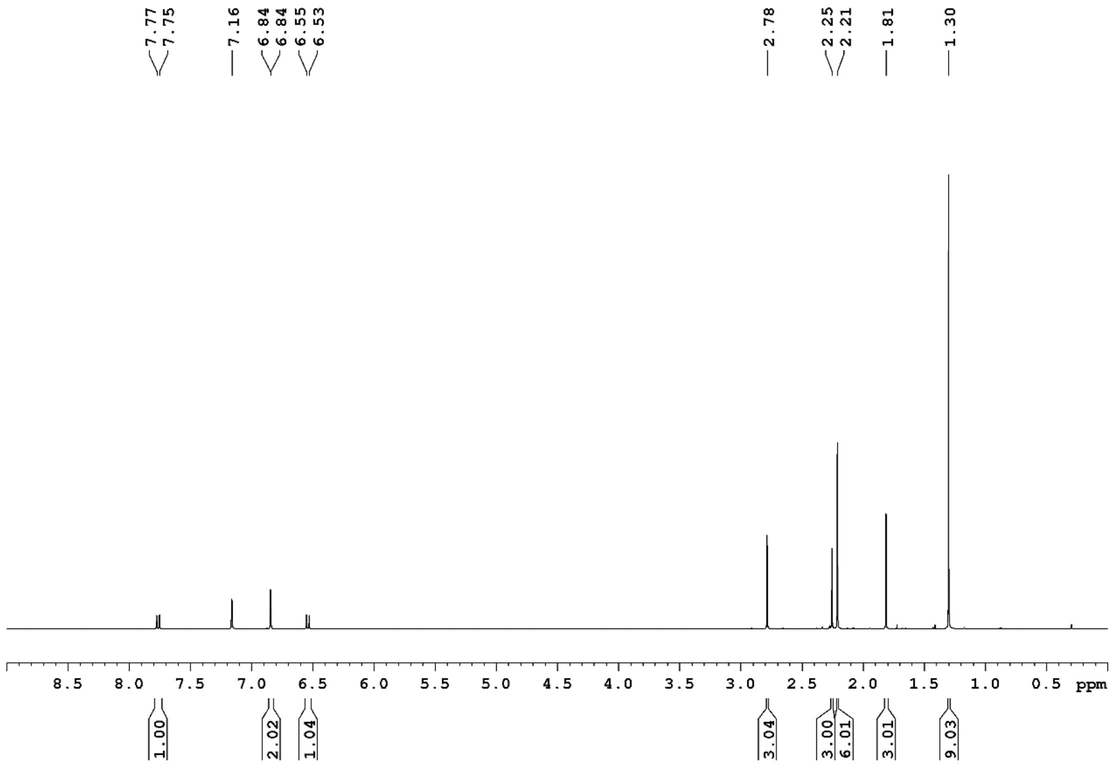

Figure S114. ^{1}H NMR (500.1 MHz, C_6D_6, 298 K) spectrum of **I**.

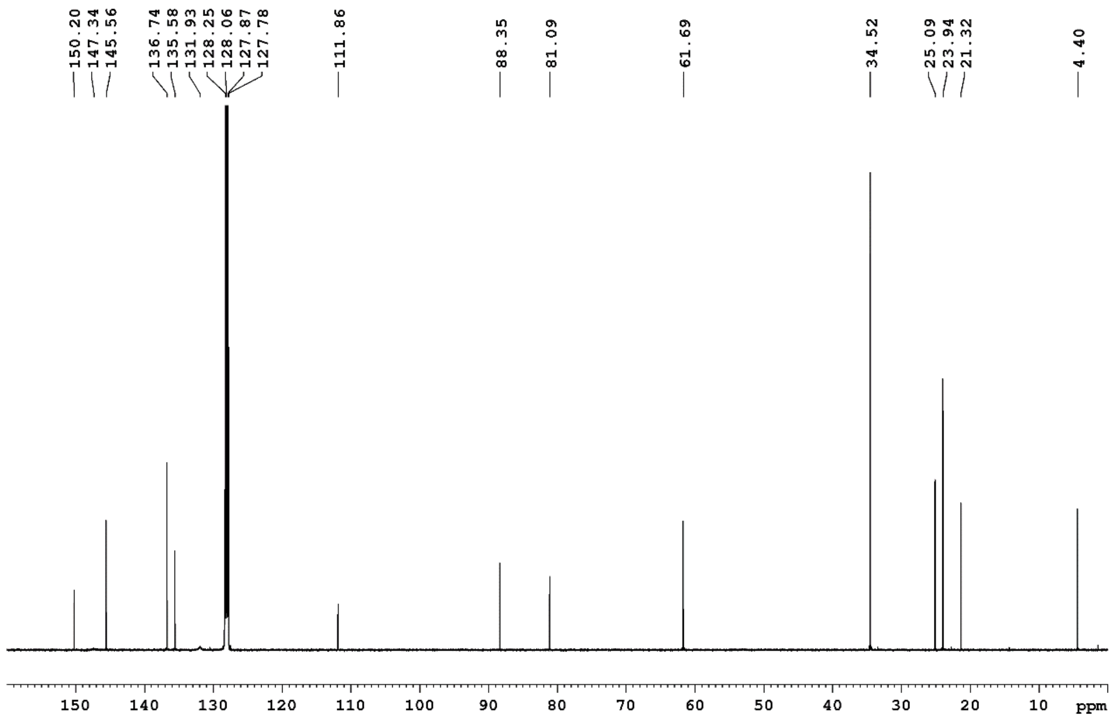

Figure S115. $^{13}C\{^{1}H\}$ NMR (125.8 MHz, C_6D_6, 298 K) spectrum of **I**.

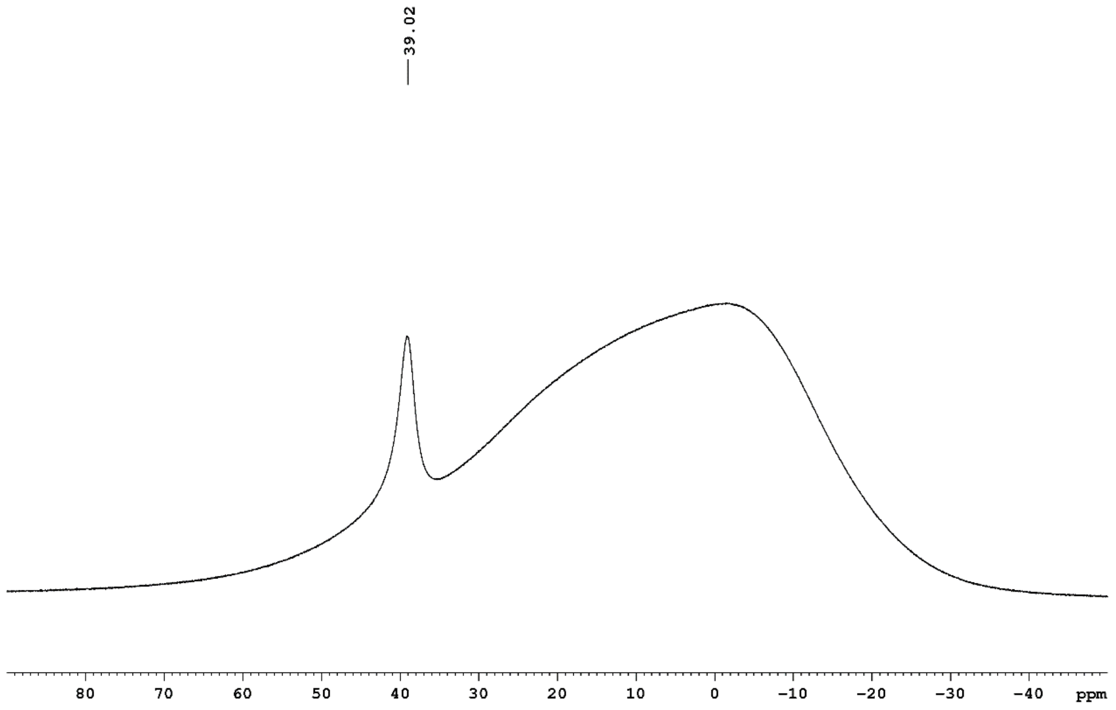

Figure S116. ^{11}B NMR (160.5 MHz, C_6D_6, 298 K) spectrum of **I**.

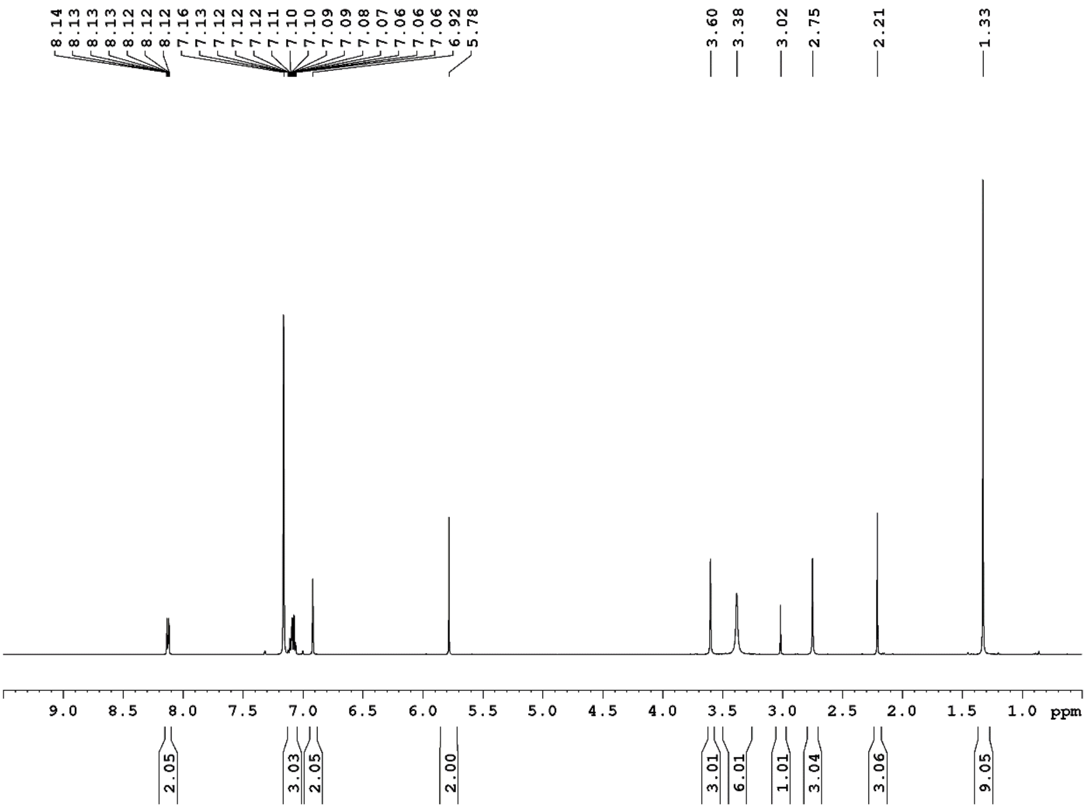

Figure S117. ^{1}H NMR (500.1 MHz, C_6D_6, 298 K) spectrum of **II**.

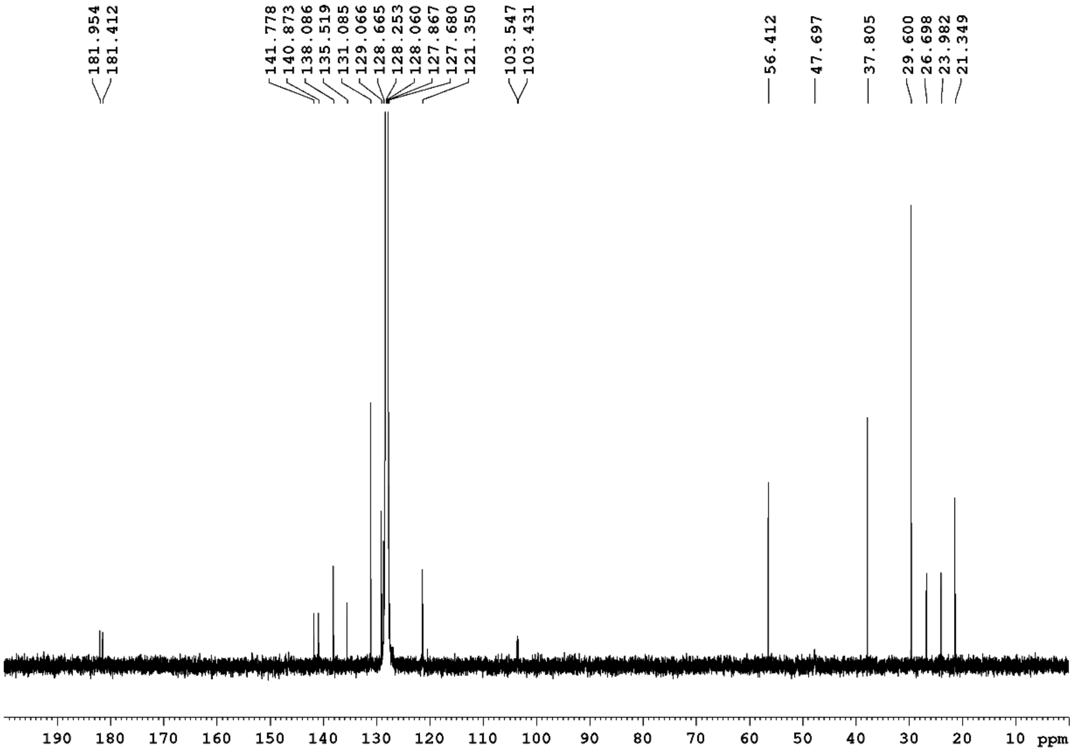

Figure S118. $^{13}C\{^{1}H\}$ NMR (125.8 MHz, C_6D_6, 298 K) spectrum of **II**.

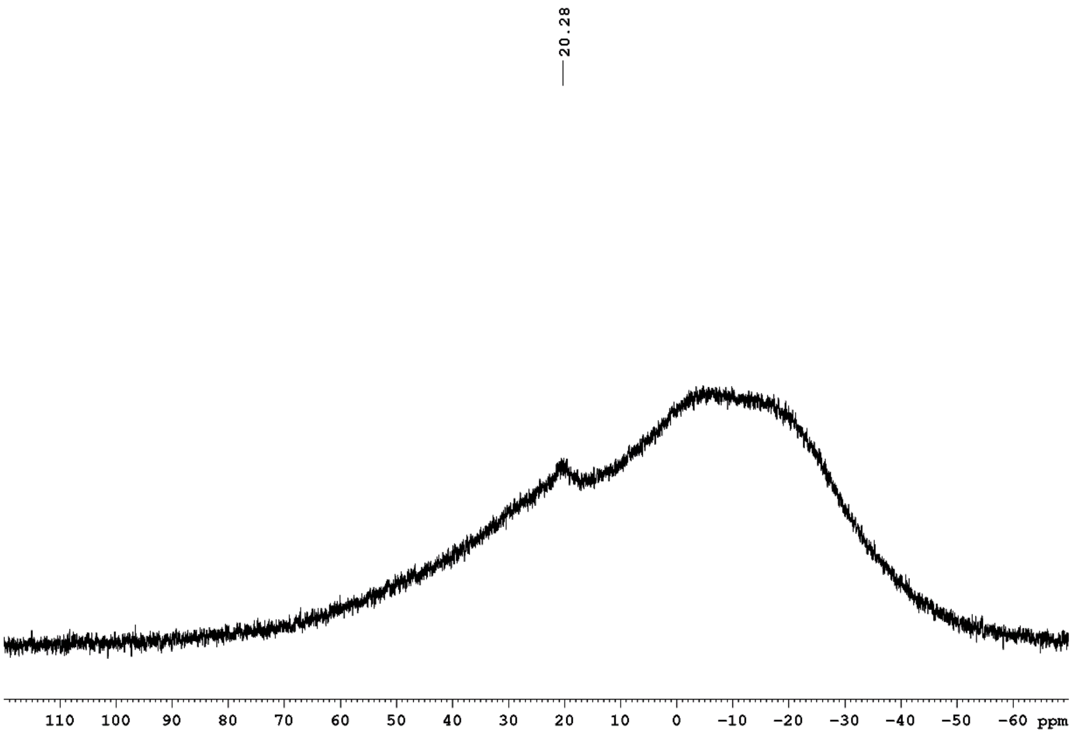

Figure S119. ^{11}B NMR (128.5 MHz, C_6D_6, 298 K) spectrum of **II**.

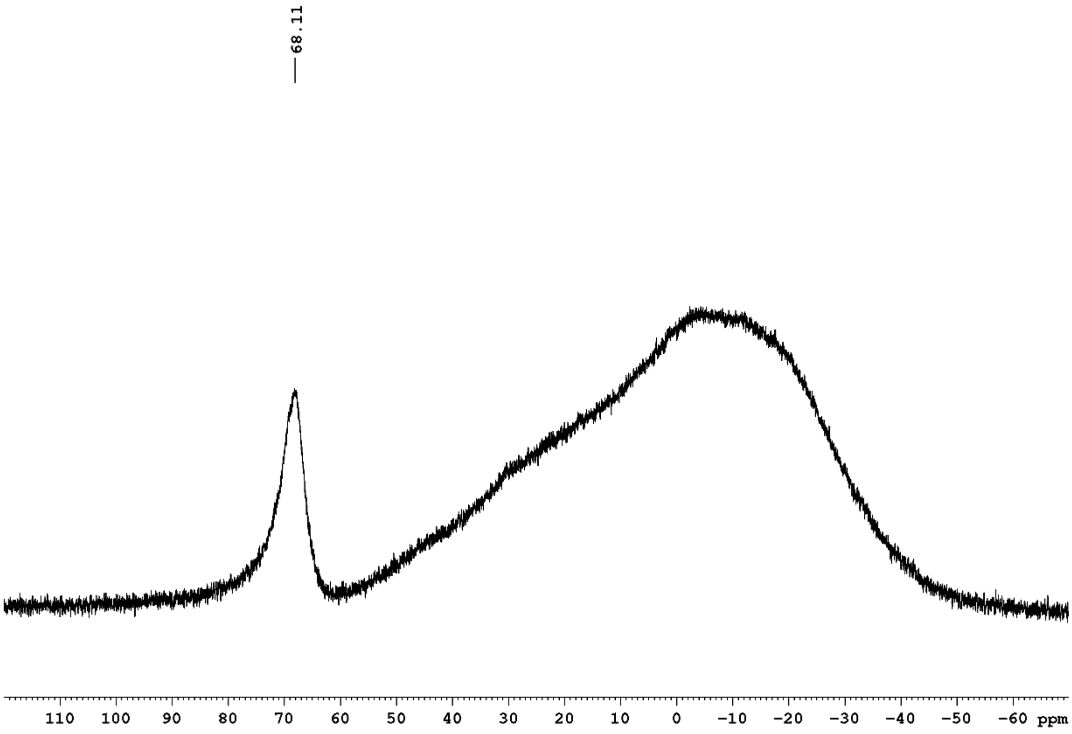

Figure S120. ^{11}B NMR (128.5 MHz, C_6D_6, 298 K) spectrum of the reaction of **1a** with PEt_3.

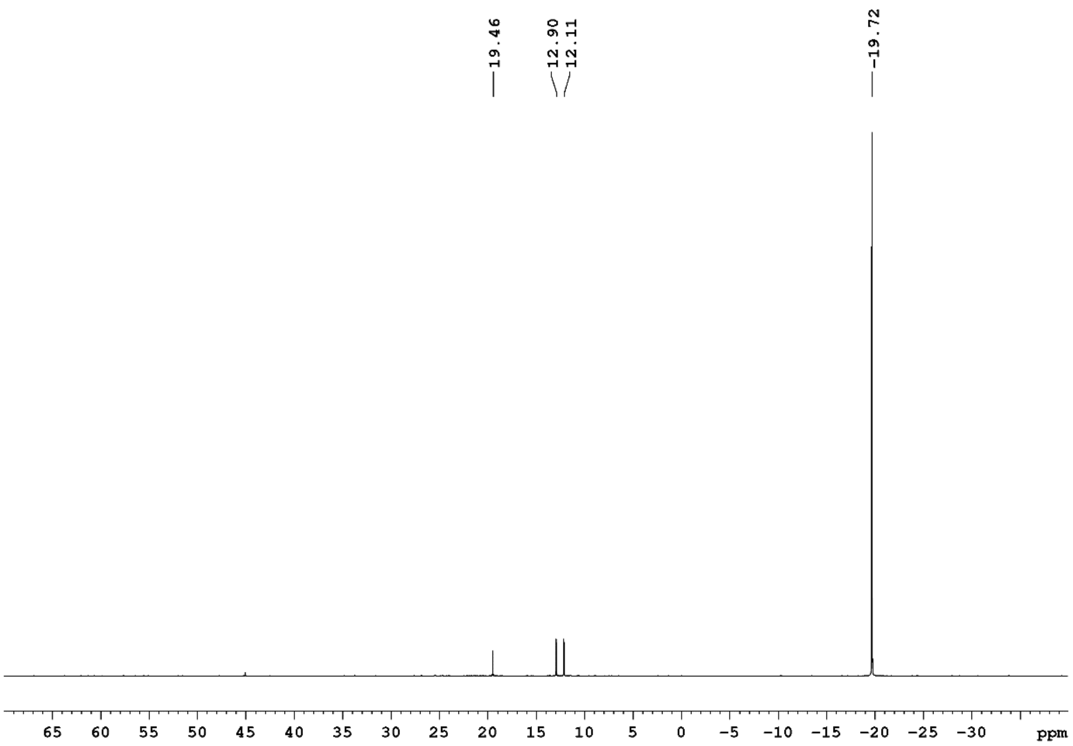

Figure S121. $^{31}P\{^{1}H\}$ NMR (162.2 MHz, C_6D_6, 298 K) spectrum of the reaction of **1a** with PEt_3.

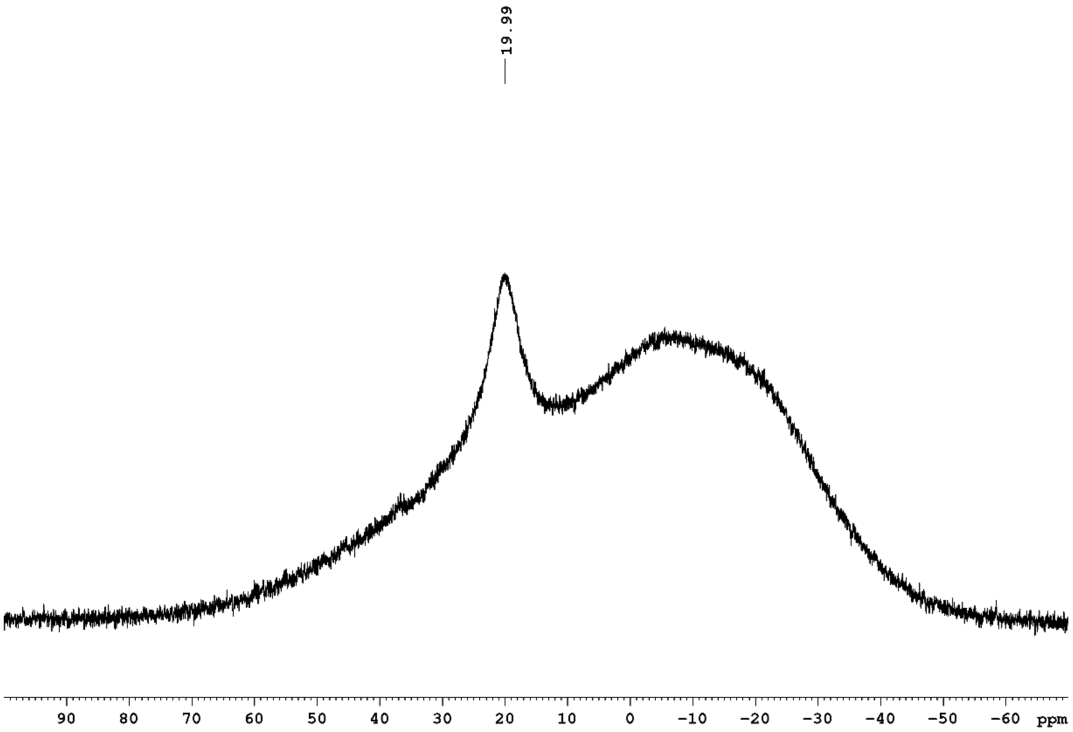

Figure S122. ^{11}B NMR (128.5 MHz, toluene, 298 K) spectrum of the reaction of **1a** with PCy_3.

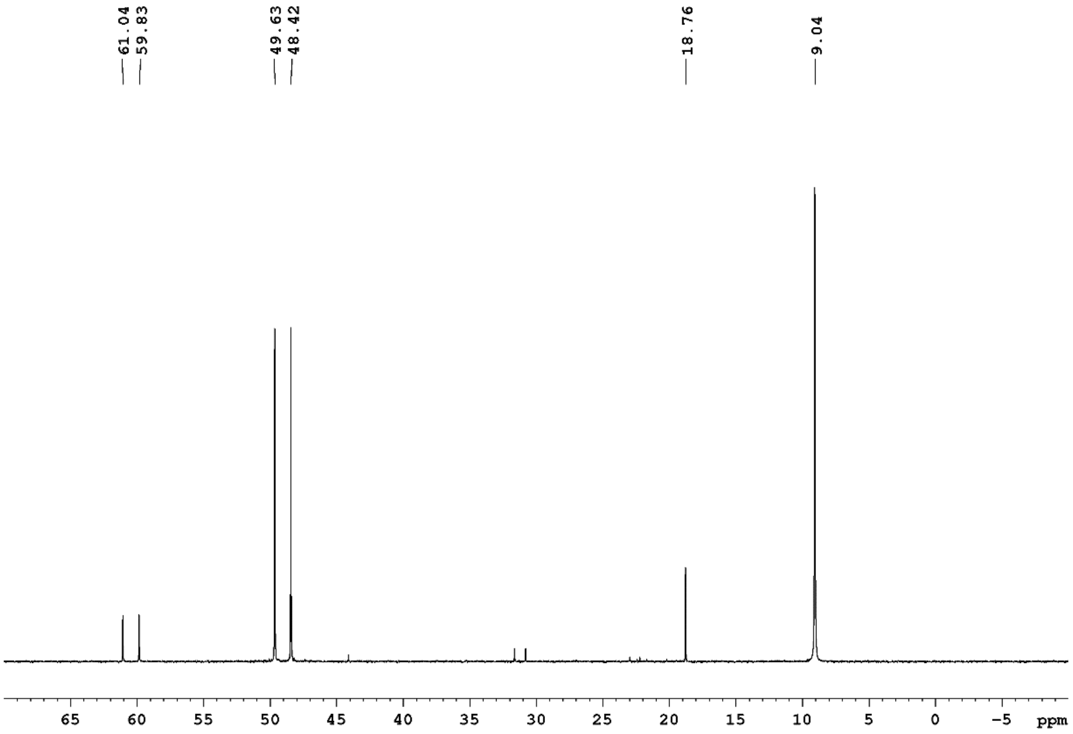

Figure S123. $^{31}P\{^{1}H\}$ NMR (162.2 MHz, toluene, 298 K) spectrum of the reaction of **1a** with PCy_3.

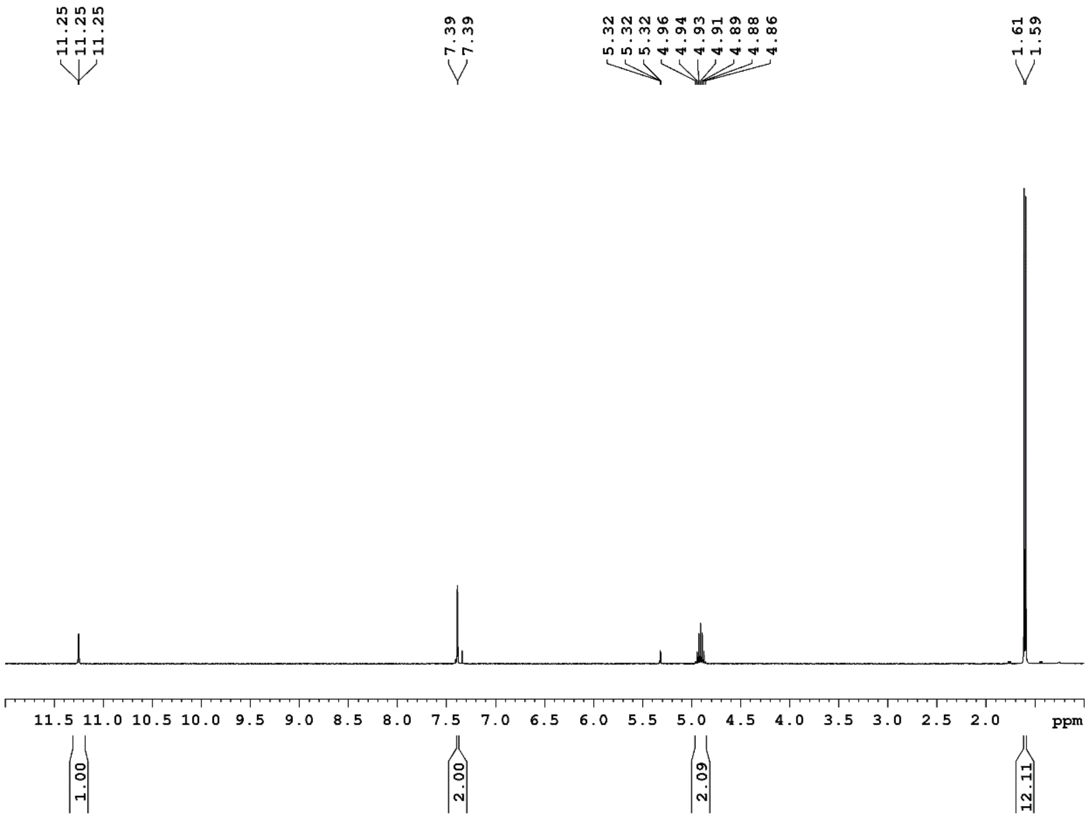

Figure S124. ^{1}H NMR (400.1 MHz, CD_2Cl_2, 298 K) spectrum of 1,3-di*iso*propylimidazolium chloride.

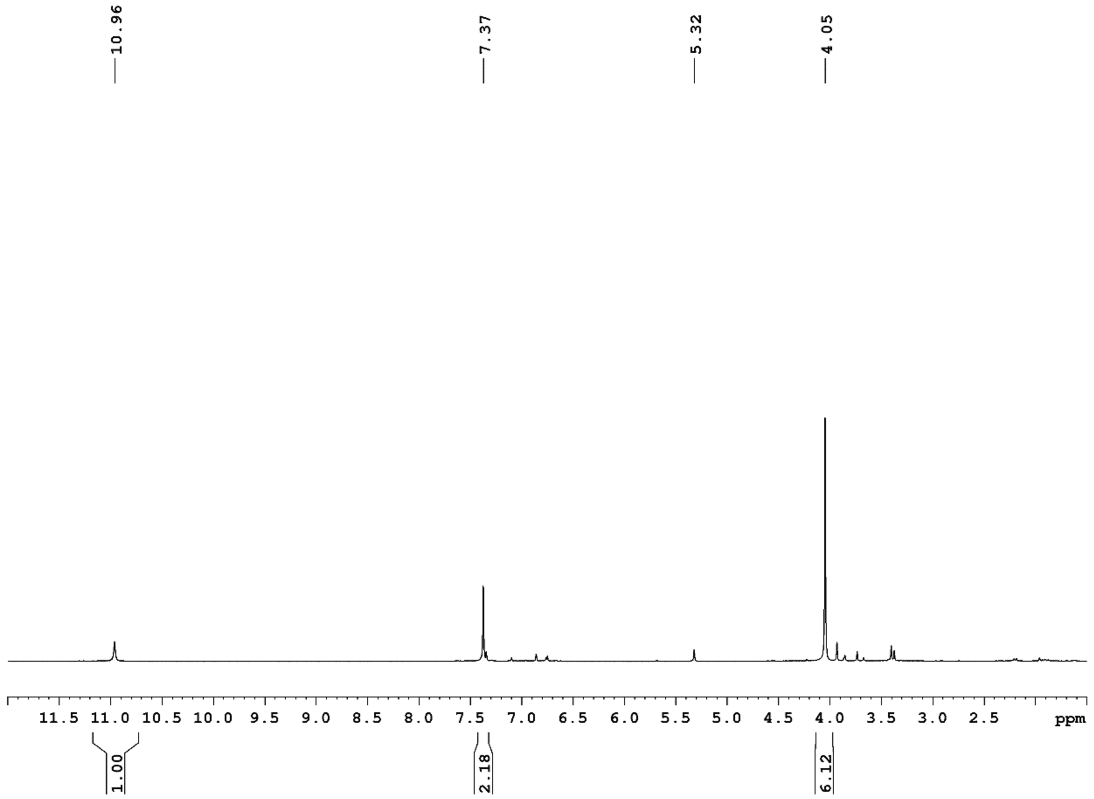

Figure S125. ^{1}H NMR (400.3 MHz, CD_2Cl_2, 298 K) spectrum of 1,3-dimethylimidazolium chloride.

UV-Vis spectra

The UV-vis absorption spectra of **1a**, **1c**, **1d**, **1e**, **1f**, **1g**, **1h**, **1i**, **1j**, **1k**, **1l**, **2a**, **2b**, **2c**, **2d**, **2e**, **2f**, **2g**, **2l** and **3a**$^{\mathbf{Me}}$ were measured on a JASCO V-660 UV-vis spectrometer. The UV-vis absorption spectra of **4a**$^{\mathbf{iPr}}$ and **4e**$^{\mathbf{iPr}}$ were measured on a METTLER TOLEDO UV-vis Excellence UV5 spectrophotometer.

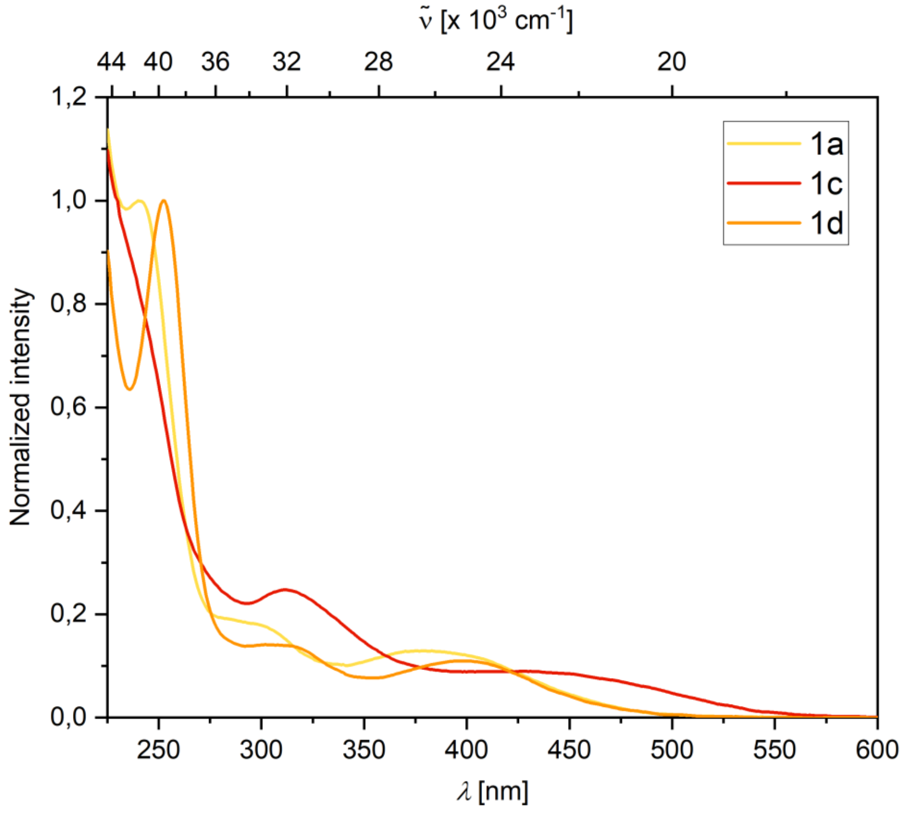

Figure S126. UV-vis absorption spectra of **1a**, **1c** and **1d** in hexane.

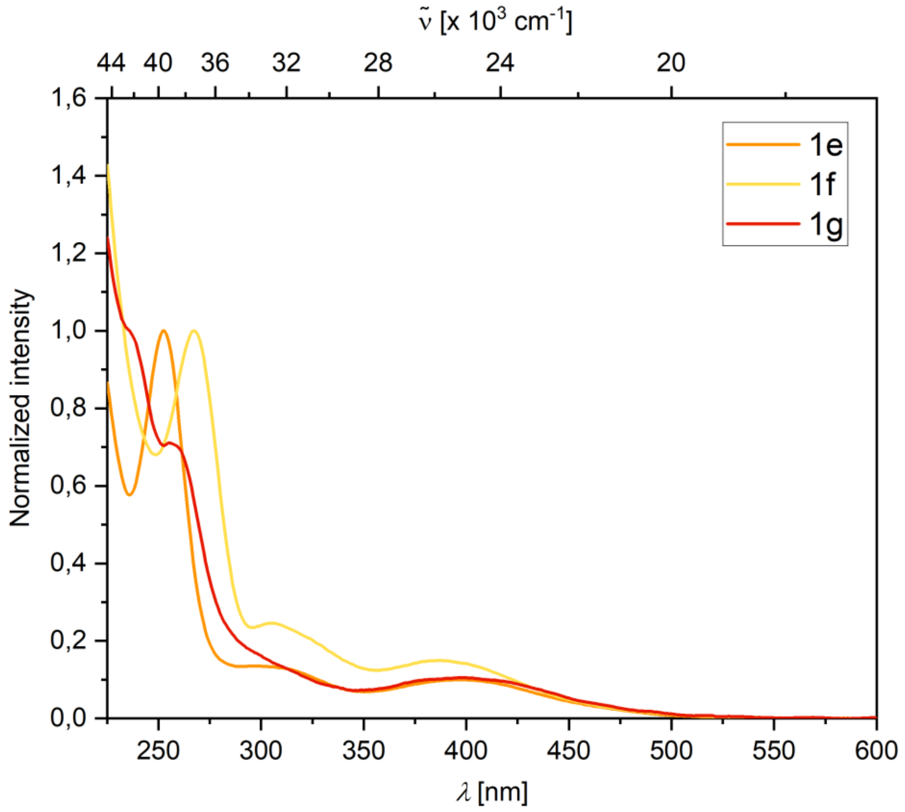

Figure S127. UV-vis absorption spectra of **1e**, **1f** and **1g** in hexane.

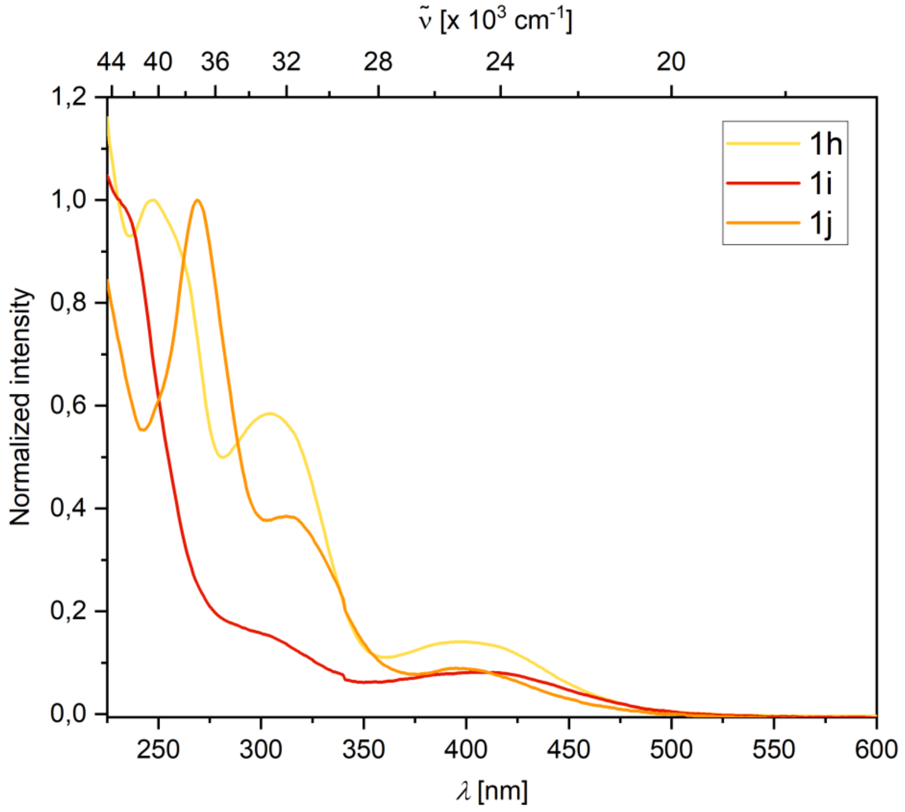

Figure S128. UV-vis absorption spectra of **1h**, **1i** and **1j** in hexane.

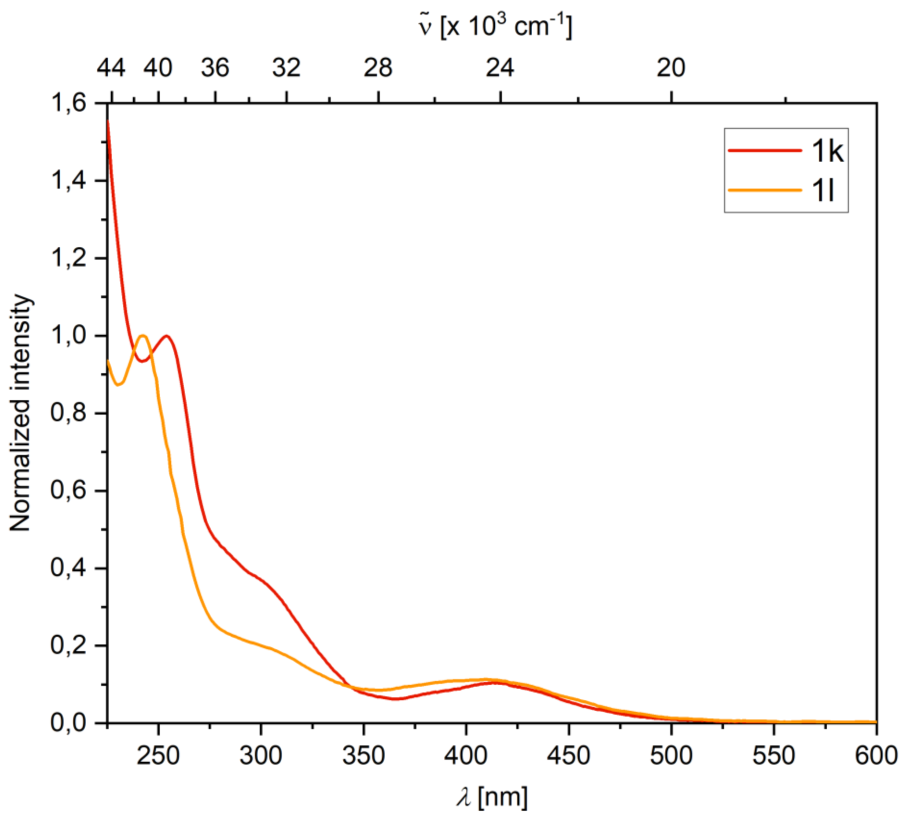

Figure S129. UV-vis absorption spectra of **1k** in hexane and of **1l** in THF.

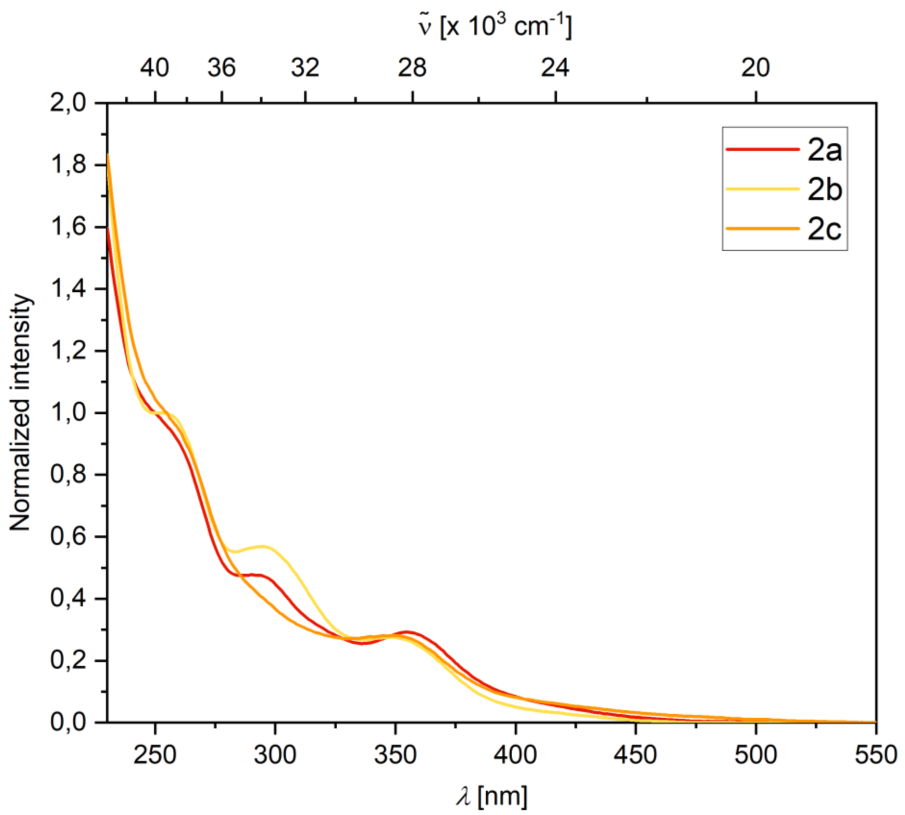

Figure S130. UV-vis absorption spectra of **2a** and **2b** in hexane and of **2c** in THF.

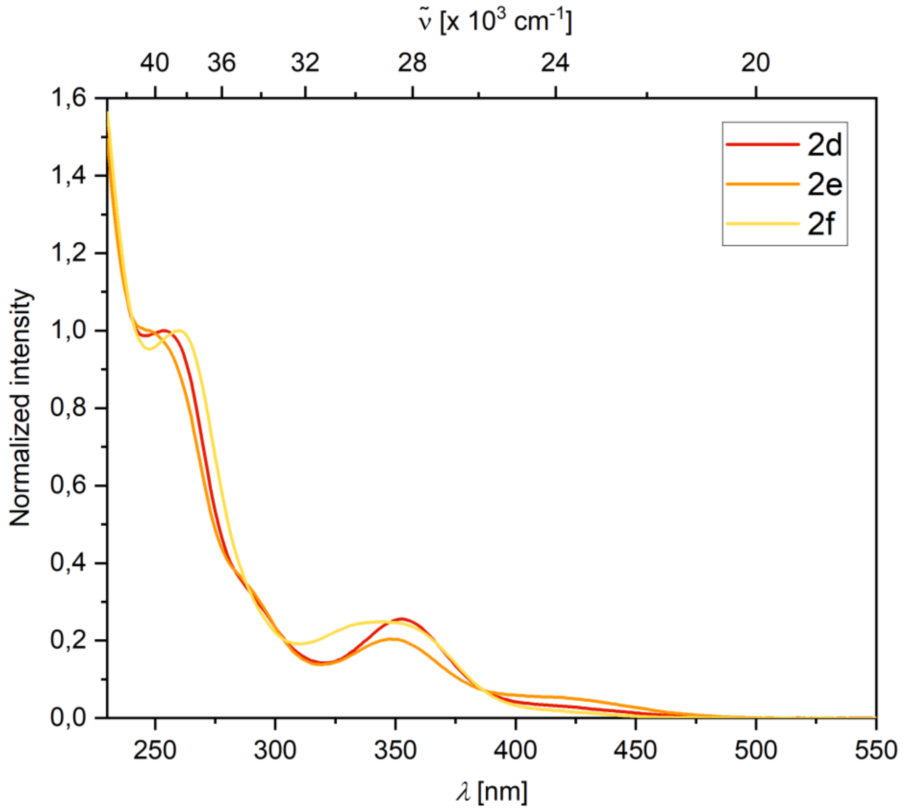

Figure S 131. UV-vis absorption spectra of **2d**, **2e** and **2f** in hexane.

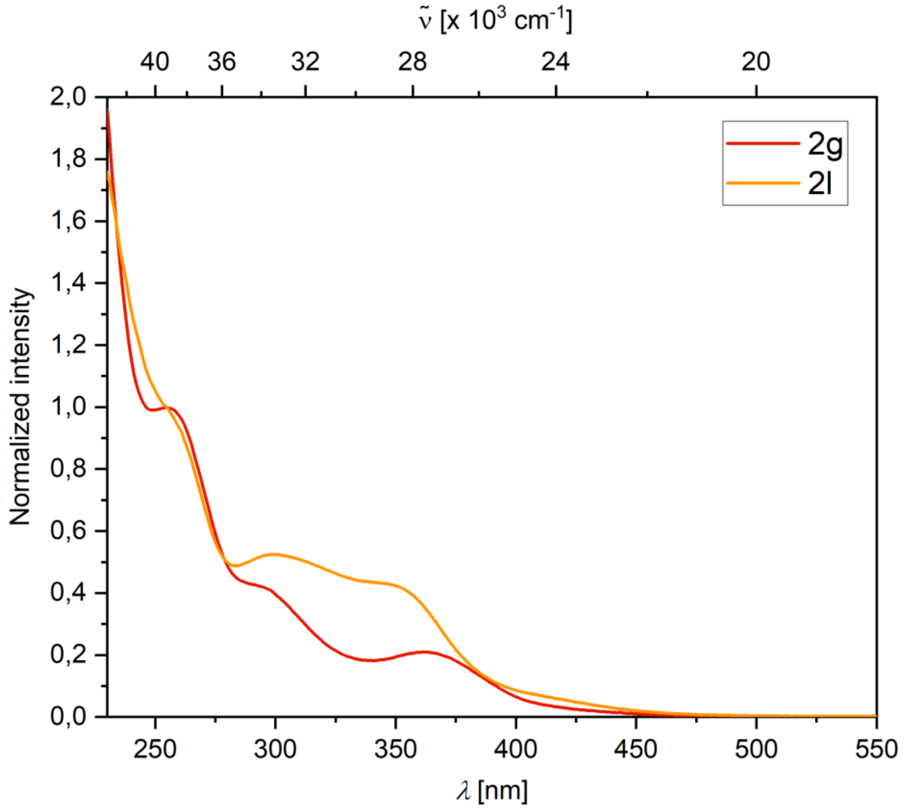

Figure S132. UV-vis absorption spectra of **2g** in hexane and of **2l** in THF.

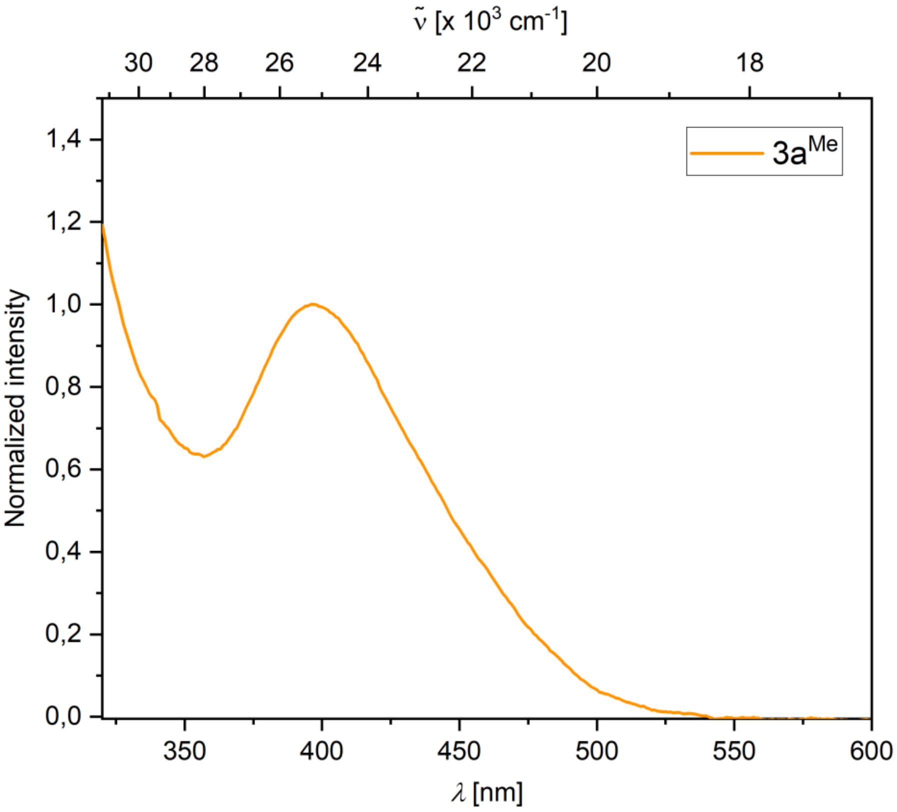

Figure S133. UV-vis absorption spectrum of **3a**$^{\mathbf{Me}}$ in THF.

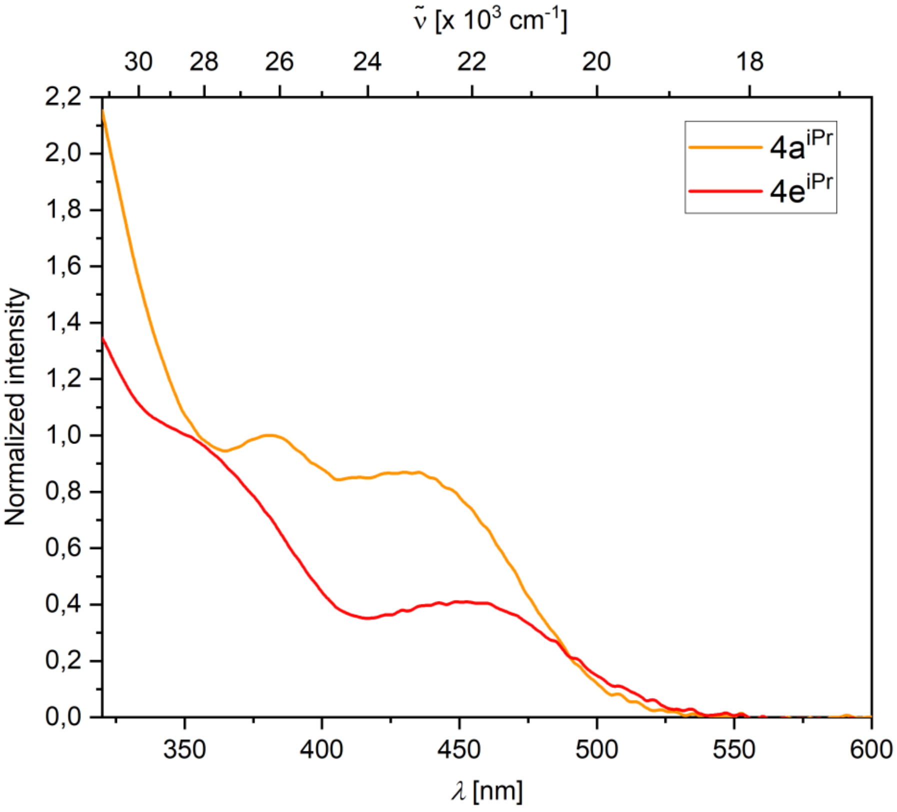

Figure S134. UV-vis absorption spectra of **4a**$^{\mathbf{iPr}}$ and **4e**$^{\mathbf{iPr}}$ in THF.

Crystal structure determination

The crystal data of **1c**, **1d**, **1g**, **1h**, **1i**, **1j**, **1k**, **2b**, **2c**, **2e**, **2f**, **2g**, **2l**, **3a**$^{\boldsymbol{i}\mathbf{Pr}}$, **3e**$^{\mathbf{Me}}$, **4a**$^{\boldsymbol{i}\mathbf{Pr}}$, **4e**$^{\boldsymbol{i}\mathbf{Pr}}$, **5**, **6**, **7** and **I (Ia)** were collected on a BRUKER D8 QUEST diffractometer with a CMOS area detector and multi-layer mirror monochromated $Mo_{K\alpha}$ radiation. The crystal data of **1a**, **1e**, **1f**, **1l**, **2a**, **2d**, **3a**$^{\mathbf{Me}}$ and **I(Ib)** were collected on a BRUKER X8-APEX II diffractometer with a CCD area detector and multi-layer mirror monochromated $Mo_{K\alpha}$ radiation. The structures were solved using the intrinsic phasing method,[11] refined with the SHELXL program[12] and expanded using Fourier techniques. All non-hydrogen atoms were refined anisotropically. Hydrogen atoms were included in structure factor calculations. All hydrogen atoms were assigned to idealized geometric positions unless otherwise stated.

Crystallographic data have been deposited with the Cambridge Crystallographic Data Center as supplementary publication no. CCDC 1997139-1997165, 1997341 and 2012450. These data can be obtained free of charge from The Cambridge Crystallographic Data Centre *via* www.ccdc.cam.ac.uk/data_request/cif

The hydrogen atoms of the azaborete four-membered ring system CH moieties of the compounds **1a**, **1c**, **1h**, **1i** and **1l** were refined with different refinement options (HFIX 43, HFIX 13 and freely refined). The different options showed no significant variations (less than double the standard deviations) concerning the bond distances and angles of the corresponding *C*H carbon atom to the surrounding heavier atoms (heavier than H). These hydrogen atoms were refined using the HFIX 13 command.

The hydrogen atoms of the allene CH moieties of the compounds **3a**$^{\mathbf{Me}}$, **3a**$^{\boldsymbol{i}\mathbf{Pr}}$, **3e**$^{\mathbf{Me}}$ and **4a**$^{\boldsymbol{i}\mathbf{Pr}}$ were refined with different refinement options (HFIX 43, HFIX 13 and freely refined). The different options showed no significant differences (less than the standard deviations) among each other concerning the bond distances and angles of the corresponding *C*H carbon atom to the surrounding heavier atoms (heavier than H). These hydrogen atoms were refined using the HFIX 43 command.

Refinement details for 1d: The two most disagreeable reflections were omitted.

Refinement details for 1e: The structure was refined using the TWIN keyword. The BASF parameter was refined to 46.9%.

Refinement details for 1g: The most disagreeable reflection was omitted. The displacement parameters of atoms B1_1 and B1_10 of the residues RESI 1 and RESI 10 were constrained to the same value with the EADP keyword. The displacement parameters of atoms B1_1 > C6_10 of the residues RESI 1 and RESI 10 were restrained to the same value with the similarity restraint SIMU. The Uii displacement parameters of atoms B1_1 > C6_10 of the residues RESI 1 and RESI 10 were restrained with the ISOR keyword to approximate isotropic behavior. The atomic displacement parameters of atoms B1_1 > C6_10 were restrained with the RIGU keyword in the ShelXL input ('enhanced rigid bond' restraint for all bonds in the connectivity list. A standard value of 0.003 was used). The 1-2 and 1-3 distances in the residues RESI 1 and RESI 10 were restrained to the same values with the SAME keyword.

Refinement details for 1h: The most disagreeable reflection was omitted.

Refinement details for 1i: The most disagreeable reflection was omitted.

Refinement details for 2d: The displacement parameters of atoms C1_1 > C4_10 of the residues RESI 1 and RESI 10 were restrained to the same value with the similarity restraint SIMU. The Uii displacement parameters of atoms C1_1 > C4_10 were restrained with the ISOR keyword to approximate isotropic behavior. The atomic displacement parameters of atoms C1_1 > C4_10 were restrained with the RIGU keyword in the ShelXL input ('enhanced rigid bond' restraint for all bonds in the connectivity list. Standard values of 0.002 for both parameters s1 and s2 were used). The 1-2 and 1-3 distances in RESI 1 and RESI 10 residues were restrained to the same values with the SAME keyword. The BUMP command was used to avoid short intramolecular H-H contacts.

Refinement details for 2f: The most disagreeable reflection was omitted. The displacement parameters of atoms C1_3 > C4_10 of the residues RESI 1, RESI 3, RESI 4, RESI 5 and RESI 10 were restrained to the same value with the similarity restraint SIMU. The Uii displacement parameters of atoms C1_3 > C4_10 were restrained with the ISOR keyword to approximate isotropic behavior. The atomic displacement parameters of atoms C1_3 > C4_10 were restrained with the RIGU keyword in the ShelXL input ('enhanced rigid bond' restraint for all

bonds in the connectivity list. Standard values of 0.002 for both parameters s1 and s2 were used). The 1-2 and 1-3 distances in RESI 1 and RESI 10 residues were restrained to the same values with the SAME keyword.

Refinement details for 2g: The most disagreeable reflection was omitted.

Refinement details for 2l: The displacement parameters of atoms C1_1 > C3_2 of the residues RESI 1 and RESI 2 were restrained to the same value with the similarity restraint SIMU. The 1-2 and 1-3 distances in RESI 1 and RESI 2 residues were restrained to the same values with the SAME keyword.

Refinement details for $3a^{iPr}$: The most disagreeable reflection was omitted.

Refinement details for $4a^{iPr}$: The two most disagreeable reflections were omitted.

Refinement details for $4e^{iPr}$: The displacement parameters of atoms C1_1 > C4_10 of the residues RESI 1 and RESI 10 were restrained to the same value with the similarity restraint SIMU. The Uii displacement parameters of atoms C1_1 > C4_10 were restrained with the ISOR keyword to approximate isotropic behavior. The atomic displacement parameters of atoms C1_1 > C4_10 were restrained with the RIGU keyword in the ShelXL input ('enhanced rigid bond' restraint for all bonds in the connectivity list. Standard values of 0.004 for both parameters s1 and s2 were used).

Refinement details for 5: The most disagreeable reflection was omitted. All hydrogen atoms except H1 were assigned to idealized positions. The coordinates of H1 were refined freely.

Refinement details for 6: All hydrogen atoms except H2, H3 and H4 were assigned to idealized positions. The coordinates of H2, H3 and H4 were refined freely.

Refinement details for 7: All hydrogen atoms except H2, H4, H30 and H32 were assigned to idealized positions. The coordinates of H2, H4, H30 and H32 were refined freely. The BUMP instruction was used to avoid short intramolecular H-H contacts. The structure was refined using the TWIN keyword. The BASF parameter was refined to 12.6%. The four most disagreeable reflections were omitted.

Refinement details for Ia: The four most disagreeable reflections were omitted.

Crystal data for **1a**: $C_{25}H_{45}BClNPRh$, M_r = 539.76, yellow block, 0.156×0.122×0.069 mm^3, triclinic space group $P\bar{1}$, a = 7.2623(5) Å, b = 8.3682(6) Å, c = 23.6179(17) Å, α = 81.735(2)°, β = 87.228(2)°, γ = 72.334(2)°, V = 1353.41(17) Å^3, Z = 2, ρ_{calcd} = 1.324 g·cm^{-3}, μ = 0.801 mm^{-1}, F(000) = 568, T = 100(2) K, R_1 = 0.0494, wR^2 = 0.0733, 5548 independent reflections [2θ≤52.794°] and 284 parameters.

Crystal data for **1c**: $C_{34}H_{51}BClFeNPRh$, M_r = 709.74, orange block, 0.37×0.314×0.295 mm^3, monoclinic space group $P2_1/c$, a = 18.500(3) Å, b = 10.9446(14) Å, c = 16.701(5) Å, β = 99.868(18)°, V = 3331.5(13) Å^3, Z = 4, ρ_{calcd} = 1.415 g·cm^{-3}, μ = 1.083 mm^{-1}, F(000) = 1480, T = 100(2) K, R_1 = 0.0189, wR^2 = 0.0443, 6810 independent reflections [2θ≤52.744°] and 373 parameters.

Crystal data for **1d**: $C_{26}H_{47}BClNPRh$, M_r = 553.78, orange block, 0.237×0.162×0.053 mm^3, monoclinic space group $P2_1/n$, a = 8.4198(13) Å, b = 22.107(5) Å, c = 15.400(3) Å, β = 100.678(15)°, V = 2816.8(9) Å^3, Z = 4, ρ_{calcd} = 1.306 g·cm^{-3}, μ = 0.771 mm^{-1}, F(000) = 1168, T = 100(2) K, R_1 = 0.0361, wR^2 = 0.0550, 5761 independent reflections [2θ≤52.744°] and 294 parameters.

Crystal data for **1e**: $C_{28}H_{51}BClNPRh$, M_r = 581.83, orange block, 0.375×0.283×0.234 mm^3, monoclinic space group $P2_1$, a = 13.155(4) Å, b = 13.567(4) Å, c = 17.300(5) Å, β = 95.737(13)°, V = 3072.0(15) Å^3, Z = 4, ρ_{calcd} = 1.258 g·cm^{-3}, μ = 0.711 mm^{-1}, F(000) = 1232, T = 100(2) K, R_1 = 0.0189, wR^2 = 0.0477, 12580 independent reflections [2θ≤52.744°] and 624 parameters.

Crystal data for **1f**: $C_{28}H_{47}BClNPRh$, M_r = 577.80, orange block, 0.299×0.246×0.224 mm^3, monoclinic space group $P2_1/n$, a = 17.729(7) Å, b = 9.767(4) Å, c = 19.228(8) Å, β = 115.848(10)°, V = 2996(2) Å^3, Z = 4, ρ_{calcd} = 1.281 g·cm^{-3}, μ = 0.728 mm^{-1}, F(000) = 1216, T = 100(2) K, R_1 = 0.0264, wR^2 = 0.0588, 6125 independent reflections [2θ≤52.746°] and 312 parameters.

Crystal data for **1g**: $C_{36}H_{58}B_2ClNO_2PRh$, M_r = 727.78, orange block, 0.303×0.284×0.172 mm^3, monoclinic space group $P2_1/n$, a = 11.385(5) Å, b = 27.454(4) Å, c = 12.146(2) Å,

β = 95.31(3)°, V = 3779.9(19) Å^3, Z = 4, ρ_{calcd} = 1.279 g·cm^{-3}, μ = 0.595 mm^{-1}, F(000) = 1536, T = 100(2) K, R_1 = 0.0423, wR^2 = 0.0830, 7727 independent reflections [2θ≤52.742°] and 493 parameters.

Crystal data for **1h**: $C_{32}H_{52}BClN_2PRh$, M_r = 644.89, orange block, 0.286×0.194×0.158 mm^3, monoclinic space group $P2_1/n$, a = 8.3899(14) Å, b = 20.844(4) Å, c = 19.120(3) Å, β = 93.632(11)°, V = 3336.9(10) Å^3, Z = 4, ρ_{calcd} = 1.284 g·cm^{-3}, μ = 0.662 mm^{-1}, F(000) = 1360, T = 100(2) K, R_1 = 0.0277, wR^2 = 0.0609, 6821 independent reflections [2θ≤52.742°] and 357 parameters.

Crystal data for **1i**: $C_{31}H_{46}BClF_3NPRh$, M_r = 669.83, orange block, 0.329×0.179×0.143 mm^3, monoclinic space group $P2_1/c$, a = 14.758(4) Å, b = 11.7829(18) Å, c = 19.206(7) Å, β = 107.324(9)°, V = 3188.3(16) Å^3, Z = 4, ρ_{calcd} = 1.395 g·cm^{-3}, μ = 0.708 mm^{-1}, F(000) = 1392, T = 100(2) K, R_1 = 0.0226, wR^2 = 0.0481, 6509 independent reflections [2θ≤52.738°] and 364 parameters.

Crystal data for **1j**: $C_{39}H_{55}BClF_3N_2PRh$, M_r = 788.99, orange block, 0.268×0.259×0.102 mm^3, monoclinic space group $P2_1/c$, a = 16.099(7) Å, b = 12.139(3) Å, c = 20.044(6) Å, β = 96.049(12)°, V = 3895(2) Å^3, Z = 4, ρ_{calcd} = 1.345 g·cm^{-3}, μ = 0.592 mm^{-1}, F(000) = 1648, T = 103(2) K, R_1 = 0.0341, wR^2 = 0.0636, 7955 independent reflections [2θ≤52.744°] and 447 parameters.

Crystal data for **1k**: $C_{43}H_{69}B_2ClN_2PRh$, M_r = 804.95, orange block, 0.29×0.285×0.256 mm^3, triclinic space group $P\bar{1}$, a = 9.269(3) Å, b = 12.103(3) Å, c = 20.168(7) Å, α = 106.938(10)°, β = 91.473(14)°, γ = 96.900(13)°, V = 2144.3(11) Å^3, Z = 2, ρ_{calcd} = 1.247 g·cm^{-3}, μ = 0.529 mm^{-1}, F(000) = 856, T = 101(2) K, R_1 = 0.0236, wR^2 = 0.0561, 8743 independent reflections [2θ≤52.744°] and 471 parameters.

Crystal data for **1l**: $C_{39}H_{56}BClNPRh$, M_r = 718.98, orange block, 0.14×0.102×0.041 mm^3, monoclinic space group $P2_1/c$, a = 18.735(7) Å, b = 25.249(11) Å, c = 8.208(4) Å, β = 102.644(12)°, V = 3788(3) Å^3, Z = 4, ρ_{calcd} = 1.261 g·cm^{-3}, μ = 0.590 mm^{-1}, F(000) = 1516, T = 100(2) K, R_1 = 0.0423, wR^2 = 0.0659, 7762 independent reflections [2θ≤52.746°] and 409 parameters.

Crystal data for **2a**: $C_{22}H_{42}BClNP_2Rh$, M_r = 531.67, yellow block, 0.426×0.28×0.182 mm^3, orthorhombic space group *Pbca*, a = 10.096(3) Å, b = 18.272(9) Å, c = 28.341(2) Å, V = 5228(3) Å^3, Z = 8, ρ_{calcd} = 1.351 g·cm^{-3}, μ = 0.886 mm^{-1}, F(000) = 2224, T = 100(2) K, R_1 = 0.0250, wR^2 = 0.0578, 5152 independent reflections [2θ≤52.04°] and 266 parameters.

Crystal data for **2b**: $C_{27}H_{44}BClNP_2Rh$, M_r = 593.74, yellow block, 0.25×0.243×0.216 mm^3, monoclinic space group $P2_1/n$, a = 9.117(3) Å, b = 18.699(4) Å, c = 17.128(6) Å, β = 92.047(16)°, V = 2918.0(15) Å^3, Z = 4, ρ_{calcd} = 1.352 g·cm^{-3}, μ = 0.802 mm^{-1}, F(000) = 1240, T = 101(2) K, R_1 = 0.0222, wR^2 = 0.0479, 5966 independent reflections [2θ≤52.744°] and 310 parameters.

Crystal data for **2c**: $C_{31}H_{48}BClFeNP_2Rh$, M_r = 701.66, yellow needle, 0.417×0.164×0.088 mm^3, monoclinic space group $P2_1/n$, a = 11.621(3) Å, b = 38.859(7) Å, c = 14.802(4) Å, β = 99.837(7)°, V = 6586(3) Å^3, Z = 8, ρ_{calcd} = 1.415 g·cm^{-3}, μ = 1.141 mm^{-1}, F(000) = 2912, T = 100(2) K, R_1 = 0.0307, wR^2 = 0.0497, 13475 independent reflections [2θ≤52.744°] and 709 parameters.

Crystal data for **2d**: $C_{23}H_{44}BClNP_2Rh$, M_r = 545.70, yellow block, 0.415×0.30×0.272 mm^3, monoclinic space group $P2_1/c$, a = 13.118(5) Å, b = 14.618(8) Å, c = 28.215(10) Å, β = 92.720(16)°, V = 5404(4) Å^3, Z = 8, ρ_{calcd} = 1.341 g·cm^{-3}, μ = 0.859 mm^{-1}, F(000) = 2288, T = 100(2) K, R_1 = 0.0237, wR^2 = 0.0575, 10634 independent reflections [2θ≤52.044°] and 591 parameters.

Crystal data for **2e**: $C_{25}H_{48}BClNP_2Rh$, M_r = 573.75, yellow block, 0.265×0.216×0.121 mm^3, triclinic space group $P\bar{1}$, a = 9.9075(19) Å, b = 10.1161(14) Å, c = 14.978(4) Å, α = 84.197(7)°, β = 79.842(10)°, γ = 77.786(7)°, V = 1441.0(5) Å^3, Z = 2, ρ_{calcd} = 1.322 g·cm^{-3}, μ = 0.809 mm^{-1}, F(000) = 604, T = 100(2) K, R_1 = 0.0192, wR^2 = 0.0479, 5907 independent reflections [2θ≤52.744°] and 294 parameters.

Crystal data for **2f**: $C_{90}H_{147}B_3Cl_3N_3P_6Rh_3$, M_r = 1904.43, yellow block, 0.321×0.242×0.228 mm^3, triclinic space group $P\bar{1}$, a = 12.921(2) Å, b = 20.038(5) Å, c = 21.929(4) Å, α = 107.641(11)°, β = 104.548(13)°, γ = 103.913(12)°, V = 4919.3(17) Å^3,

$Z = 2$, $\rho_{calcd} = 1.286$ g·cm^{-3}, $\mu = 0.718$ mm^{-1}, $F(000) = 1998$, $T = 100(2)$ K, $R_1 = 0.0346$, $wR^2 = 0.0753$, 20119 independent reflections [2θ≤52.744°] and 1054 parameters.

Crystal data for **2g**: $C_{33}H_{55}B_2ClNO_2P_2Rh$, $M_r = 719.70$, yellow block, 0.226×0.22×0.18 mm^3, monoclinic space group $P2_1/c$, $a = 9.831(6)$ Å, $b = 19.140(9)$ Å, $c = 19.599(10)$ Å, $\beta = 101.13(3)°$, $V = 3618(3)$ Å^3, $Z = 4$, $\rho_{calcd} = 1.321$ g·cm^{-3}, $\mu = 0.663$ mm^{-1}, $F(000) = 1512$, $T = 100(2)$ K, $R_1 = 0.0626$, $wR^2 = 0.0926$, 7391 independent reflections [2θ≤52.742°] and 395 parameters.

Crystal data for **2l**: $C_{30}H_{47}BClNP_2Rh$, $M_r = 632.79$, yellow block, 0.266×0.258×0.098 mm^3, monoclinic space group $C2/c$, $a = 22.645(8)$ Å, $b = 18.484(6)$ Å, $c = 17.744(3)$ Å, $\beta = 122.497(8)°$, $V = 6264(3)$ Å^3, $Z = 8$, $\rho_{calcd} = 1.342$ g·cm^{-3}, $\mu = 0.752$ mm^{-1}, $F(000) = 2648$, $T = 101(2)$ K, $R_1 = 0.0295$, $wR^2 = 0.0574$, 6403 independent reflections [2θ≤52.744°] and 338 parameters.

Crystal data for **3a^{Me}**: $C_{30}H_{52}BN_3PRh$, $M_r = 599.43$, orange block, 0.315×0.244×0.174 mm^3, monoclinic space group $P2_1/n$, $a = 12.009(6)$ Å, $b = 18.218(8)$ Å, $c = 14.765(13)$ Å, $\beta = 107.74(2)°$, $V = 3077(3)$ Å^3, $Z = 4$, $\rho_{calcd} = 1.294$ g·cm^{-3}, $\mu = 0.630$ mm^{-1}, $F(000) = 1272$, $T = 100(2)$ K, $R_1 = 0.0676$, $wR^2 = 0.0922$, 6284 independent reflections [2θ≤52.744°] and 339 parameters.

Crystal data for **3a^{iPr}**: $C_{34}H_{60}BN_3PRh$, $M_r = 655.54$, orange block, 0.20×0.155×0.09 mm^3, orthorhombic space group $P2_12_12_1$, $a = 11.529(4)$ Å, $b = 16.437(5)$ Å, $c = 18.831(5)$ Å, $V = 3568.4(18)$ Å^3, $Z = 4$, $\rho_{calcd} = 1.220$ g·cm^{-3}, $\mu = 0.549$ mm^{-1}, $F(000) = 1400$, $T = 100(2)$ K, $R_1 = 0.0305$, $wR^2 = 0.0708$, 7016 independent reflections [2θ≤52.042°] and 377 parameters.

Crystal data for **3e^{Me}**: $C_{36}H_{65}BN_3PRh$, $M_r = 684.60$, yellow plate, 0.212×0.13×0.034 mm^3, monoclinic space group $P2_1/c$, $a = 11.713(2)$ Å, $b = 16.0270(19)$ Å, $c = 20.498(4)$ Å, $\beta = 106.075(7)°$, $V = 3697.5(11)$ Å^3, $Z = 4$, $\rho_{calcd} = 1.230$ g·cm^{-3}, $\mu = 0.532$ mm^{-1}, $F(000) = 1468$, $T = 100(2)$ K, $R_1 = 0.0366$, $wR^2 = 0.0692$, 7569 independent reflections [2θ≤52.744°] and 396 parameters.

Crystal data for **4a^{iPr}**: $C_{34}H_{55}BN_5Rh$, $M_r = 647.55$, yellow needle, 0.266×0.119×0.056 mm^3, monoclinic space group $P2_1/n$, $a = 11.060(3)$ Å, $b = 18.039(3)$ Å, $c = 17.701(2)$ Å,

β = 94.498(12)°, V = 3520.7(13) Å^3, Z = 4, ρ_{calcd} = 1.222 g·cm^{-3}, μ = 0.514 mm^{-1}, F(000) = 1376, T = 100(2) K, R_1 = 0.0292, wR^2 = 0.0577, 7203 independent reflections [2θ≤52.744°] and 384 parameters.

Crystal data for **4e^{iPr}**: $C_{37}H_{61}BN_5Rh$, M_r = 689.62, orange block, 0.31×0.257×0.11 mm^3, monoclinic space group $P2_1/n$, a = 11.969(2) Å, b = 19.828(4) Å, c = 15.990(3) Å, β = 91.168(6)°, V = 3793.9(12) Å^3, Z = 4, ρ_{calcd} = 1.207 g·cm^{-3}, μ = 0.481 mm^{-1}, F(000) = 1472, T = 100(2) K, R_1 = 0.0237, wR^2 = 0.0517, 7745 independent reflections [2θ≤52.744°] and 453 parameters.

Crystal data for **5**: $C_{28}H_{51}BClNPRh$, M_r = 581.83, yellow needles, 0.417×0.279×0.156 mm^3, monoclinic space group $P2_1/n$, a = 11.254(4) Å, b = 9.024(2) Å, c = 29.373(8) Å, β = 98.317(13)°, V = 2951.7(14) Å^3, Z = 4, ρ_{calcd} = 1.309 g·cm^{-3}, μ = 0.740 mm^{-1}, F(000) = 1232, T = 100(2) K, R_1 = 0.0553, wR^2 = 0.0688, 5800 independent reflections [2θ≤52.04°] and 315 parameters.

Crystal data for **6**: $C_{28}H_{51}BClNPRh$, M_r = 581.83, orange needle, 0.512×0.077×0.057 mm^3, monoclinic space group $P2_1/c$, a = 14.740(3) Å, b = 15.319(4) Å, c = 14.833(3) Å, β = 116.828(9)°, V = 2988.9(12) Å^3, Z = 4, ρ_{calcd} = 1.293 g·cm^{-3}, μ = 0.730 mm^{-1}, F(000) = 1232, T = 100(2) K, R_1 = 0.0438, wR^2 = 0.0981, 6124 independent reflections [2θ≤52.74°] and 321 parameters.

Crystal data for **7**: $C_{28}H_{51}BClNPRh$, M_r = 581.83, orange block, 0.121×0.093×0.069 mm^3, orthorhombic space group $Pca2_1$, a = 21.577(5) Å, b = 8.0109(19) Å, c = 34.845(8) Å, V = 6023(2) Å^3, Z = 8, ρ_{calcd} = 1.283 g·cm^{-3}, μ = 0.725 mm^{-1}, F(000) = 2464, T = 100(2) K, R_1 = 0.0695, wR^2 = 0.1221, 13278 independent reflections [2θ≤54.206°] and 634 parameters.

Crystal data for **I(Ia)**: $C_{21}H_{28}BN$, M_r = 305.25, colourless plate, 0.412×0.224×0.106 mm^3, monoclinic space group $P2_1/c$, a = 16.329(5) Å, b = 9.070(3) Å, c = 13.365(3) Å, β = 110.285(5)°, V = 1856.8(8) Å^3, Z = 4, ρ_{calcd} = 1.092 g·cm^{-3}, μ = 0.061 mm^{-1}, F(000) = 664, T = 100(2) K, R_1 = 0.0497, wR^2 = 0.1248, 3811 independent reflections [2θ≤52.744°] and 216 parameters.

Crystal data for **I(Ib)**: $C_{21}H_{28}BN$, M_r = 305.25, colourless plate, 0.291×0.157×0.108 mm^3, orthorhombic space group $Pca2_1$, a = 12.670(8) Å, b = 11.135(6) Å, c = 13.004(8) Å, V = 1834.6(18) Å^3, Z = 4, ρ_{calcd} = 1.105 g·cm^{-3}, μ = 0.062 mm^{-1}, F(000) = 664, T = 100(2) K, R_1 = 0.0484, wR^2 = 0.0961, 3484 independent reflections [2θ≤52.724°] and 216 parameters.

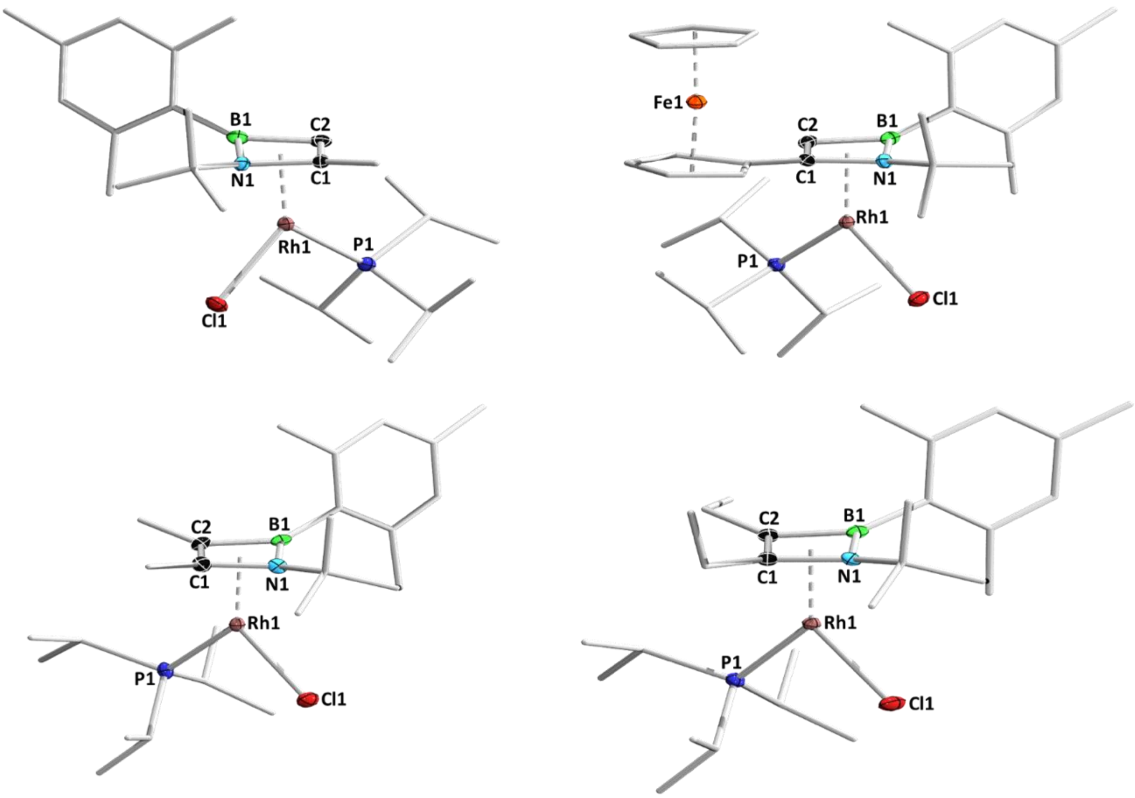

Figure S135. Crystallographically-derived molecular structures of complexes **1a** (top left), **1c** (top right), **1d** (bottom left) and **1e** (bottom right) with atomic displacement ellipsoids at the 50% probability level. Some ellipsoids and all hydrogen atoms are omitted for clarity.

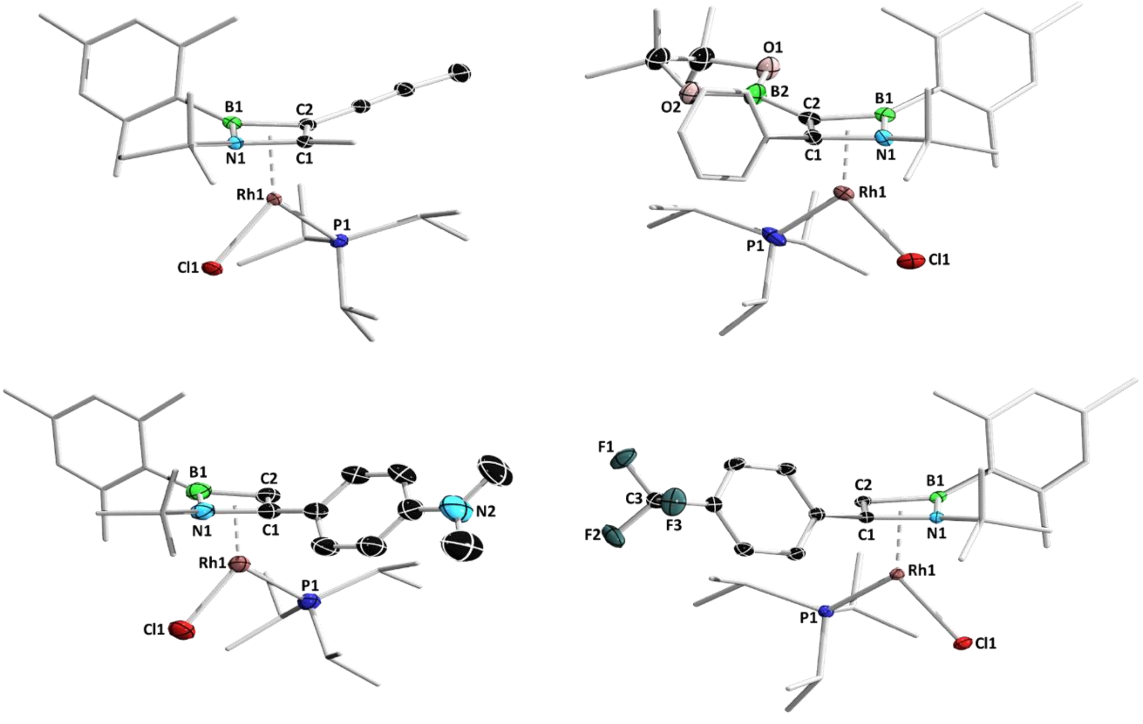

Figure S136. Crystallographically-derived molecular structures of complexes **1f** (top left), **1g** (top right), **1h** (bottom left) and **1i** (bottom right) with atomic displacement ellipsoids at the 50% probability level. Some ellipsoids and all hydrogen atoms are omitted for clarity.

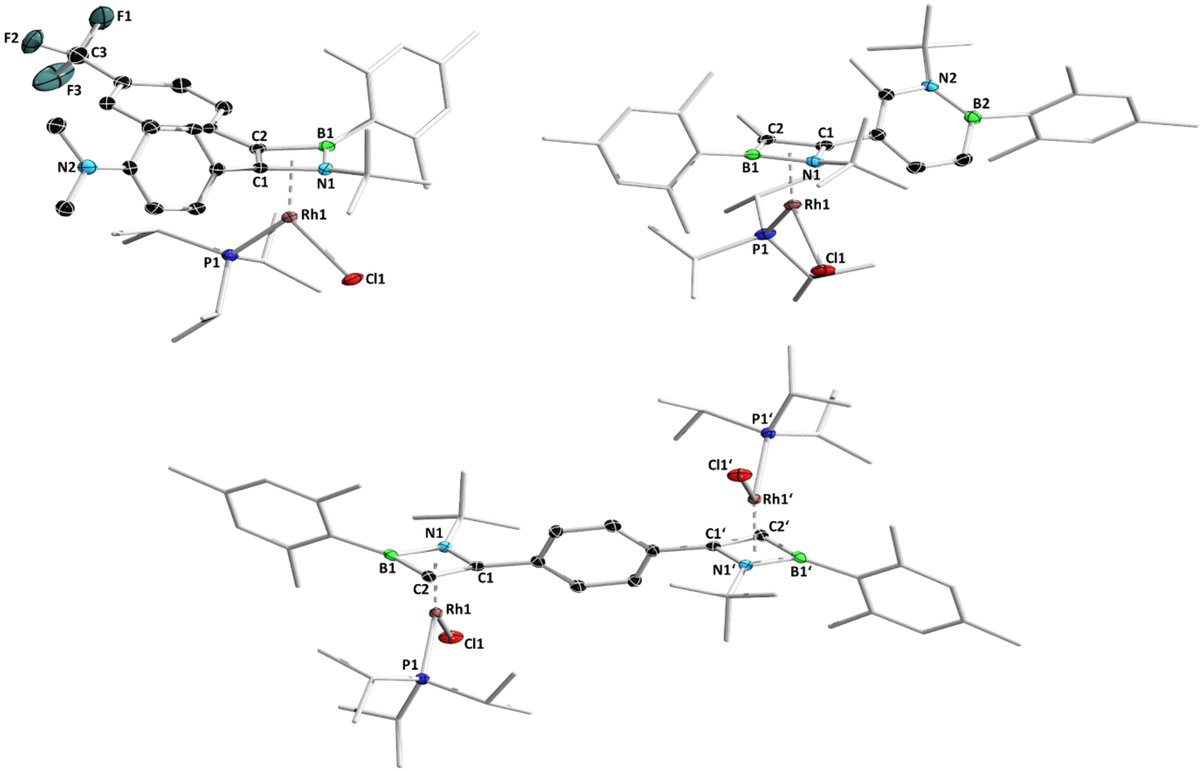

Figure S137. Crystallographically-derived molecular structures of complexes **1j** (left), **1k** (right) and **1l** (below) with atomic displacement ellipsoids at the 50% probability level. Co-crystallised solvent molecules, some ellipsoids and all hydrogen atoms are omitted for clarity.

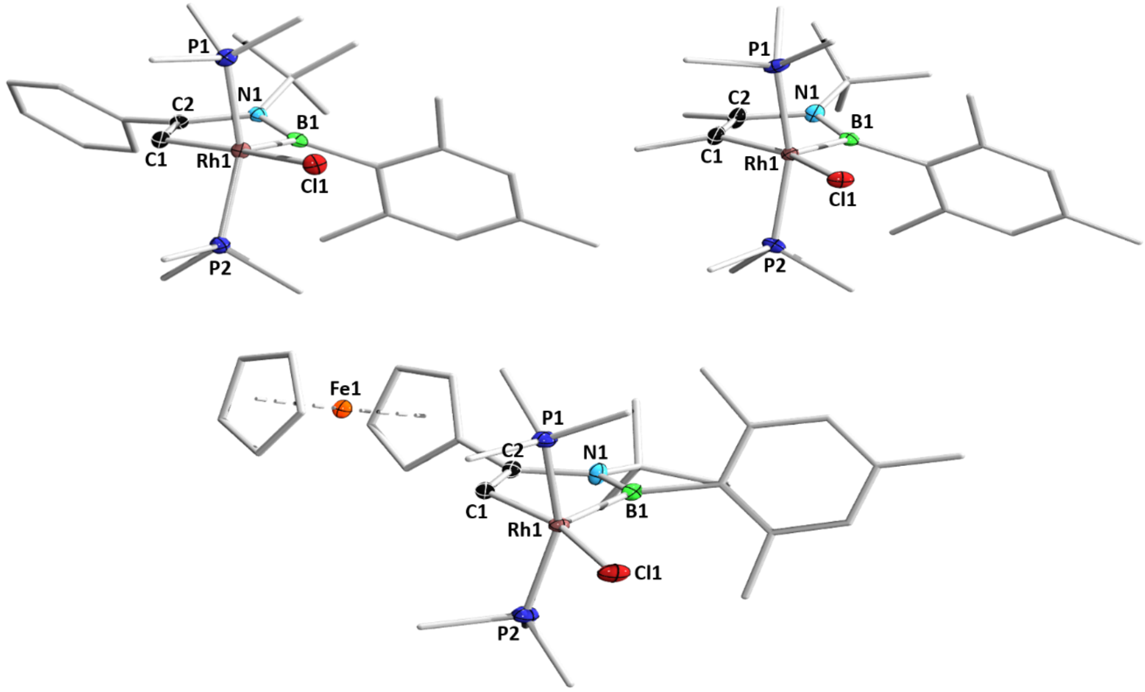

Figure S138. Crystallographically-derived molecular structures of complexes **2b** (left), **2c** (below) and **2d** (right) with atomic displacement ellipsoids at the 50% probability level. Some ellipsoids and all hydrogen atoms are omitted for clarity.

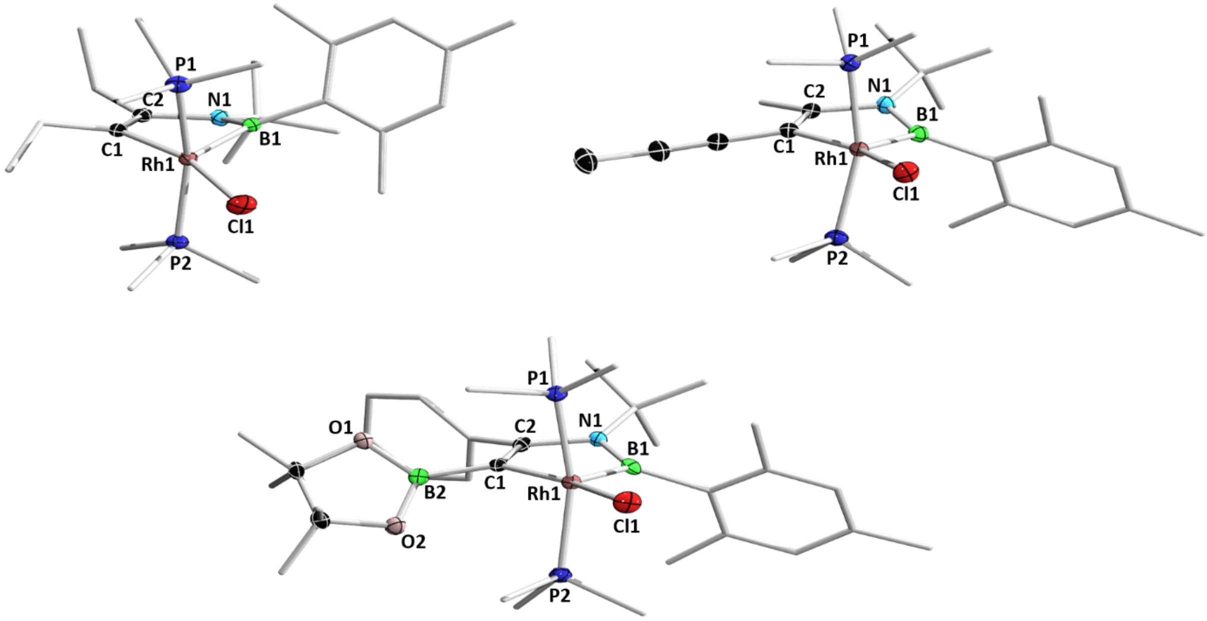

Figure S139. Crystallographically-derived molecular structures of complexes **2e** (left), **2f** (right) and **2g** (below) with atomic displacement ellipsoids at the 50% probability level. Co-crystallised solvent molecules, some ellipsoids and all hydrogen atoms are omitted for clarity.

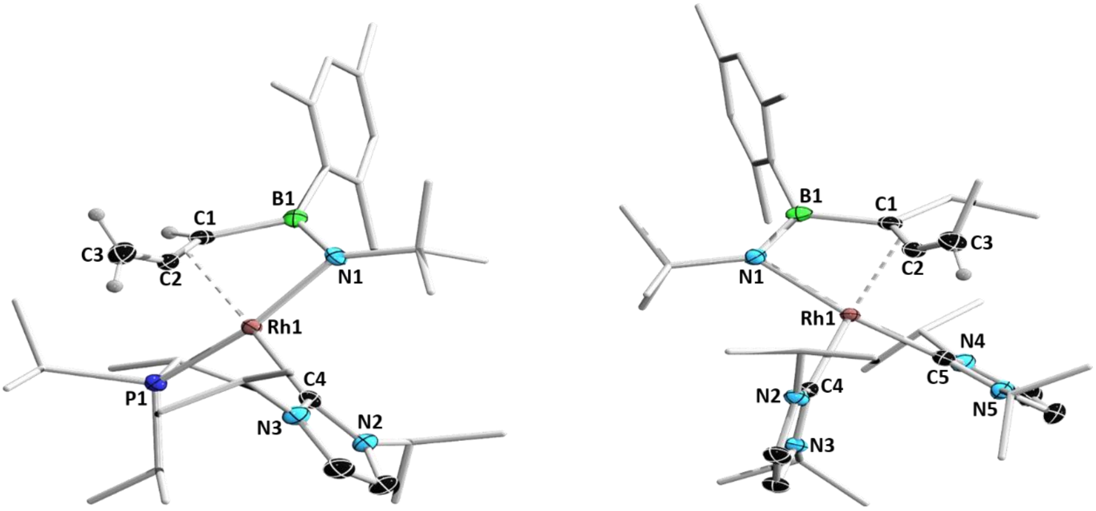

Figure S140. Crystallographically-derived molecular structures of complexes **3a**$^{i\mathrm{Pr}}$ (left) and **4e**$^{i\mathrm{Pr}}$ (right) with atomic displacement ellipsoids at the 50% probability level. Some ellipsoids and most hydrogen atoms are omitted for clarity.

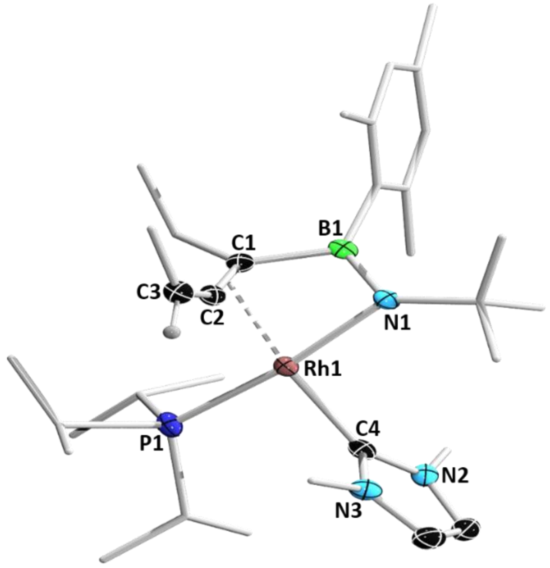

Figure S141. Crystallographically-derived molecular structures of complex **3e**^{Me} with atomic displacement ellipsoids at the 50% probability level. Co-crystallised solvent molecules, some ellipsoids and most hydrogen atoms are omitted for clarity.

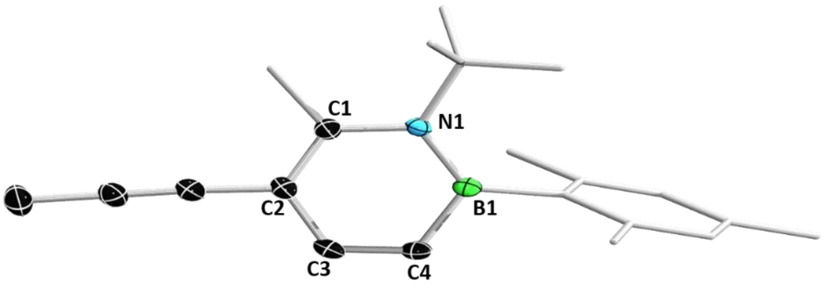

Figure S142. Crystallographically-derived molecular structure of compound **I(Ia)** with atomic displacement ellipsoids at the 50% probability level. Some ellipsoids and all hydrogen atoms are omitted for clarity.

Computational details

All calculations were performed with the Orca 4.1.1 software.[13] The structures were optimized with the PBE0 functional.[14] The def2-TZVP basis set[15] and its corresponding effective core potential (def2-ECP)[16] were used for Rh, while def2-SVP[15] was applied to all other atoms. Dispersion corrections were taking into account in the geometry optimizations by using Grimme's D3[17] model together with the Becke-Johnson (BJ) damping function.[18] In order to speed up the calculations, we included the resolution of the identity approximation for Coulomb integrals (RI-J)[19] and the chain of spheres numerical integration for Hartree-Fock exchange, COSX.[20] Hessian calculations were then performed for all optimized structures at the same level of theory. All geometries were characterized as minimum energy structures as all of their vibrational frequencies are real. Additionally, single-point calculations were performed at the PBE0-D3(BJ)/def2-QZVPP and TPSSh[21]-D3(BJ)/def2-QZVPP levels. Solvation effects were taken into account using the Solvation Model for Density (SMD)[22] with benzene ($\varepsilon = 2.28$) as solvent. A concentration correction of $\Delta G^{0\rightarrow *}$ = RTln(24.46) = 1.89 kcal mol^{-1} (T = 298.15 K) was included in the free energies of all species in order to account for the change in standard states in going from gas phase (1 atm) to the condensed phase (1 M) and to properly describe associative/dissociative steps.[23] Images of the 3D structures were obtained with CYLview.[24]

By collecting the Gibbs free energies of all structures in which calculations were performed, we were able to construct a free energy map involving the Rh.P^{i}Pr$_3$ η^4-azaborete complex **1a**, the respective azaborole complex **2a** featuring two PMe_3 ligands, and a variety of related species featuring distinct numbers (and types) of coordinating phosphines. We expect that, in solution, a complex equilibrium involving at least some of these species will be present, and a full description of the reaction from **1a** to **2a** should take into account all possible connections within the derived reaction network. We approached this problem by initially identifying in the map the thermodynamically preferred pathway, in which high-energy intermediates are avoided. We then performed calculations for identifying transition states (TS) along the thermodynamically preferred pathway. These were characterized by having one imaginary frequency mode in the Hessian calculation. In order to verify the connectivity of the TS, we performed additional geometry optimizations along the imaginary mode and intrinsic reaction coordinate (IRC)[25] calculations.

All species in the map are related to the complexes where R^1 and R^2 are Me and H, respectively. The structures were labelled as **1**, representing the azaborete, or **2**, related to the corresponding azaborole complexes. These can be followed by the letters **A**, **B**, or the combinations **AB** or **B$_2$**. **A** stands for P*i*Pr$_3$, while **B** represents PMe_3. Therefore, while **1** is the Rh η^4-azaborete chloride complex without any coordinating phosphine, **2AB** is the azaborole system in which the Rh features two coordinating phosphines: one P^{i}Pr$_3$ and one PMe_3. All molecular structures involved in the construction of the free energy map are shown in Figures S139 and S140. A flowchart depicting the reaction network is shown in Figure S141, and a mechanistic proposal for the transformation of **1A** into **2B$_2$** is given in Figure S142. Free energy values are given in kcal mol^{-1} as relative to that of the reactant, **1A**.

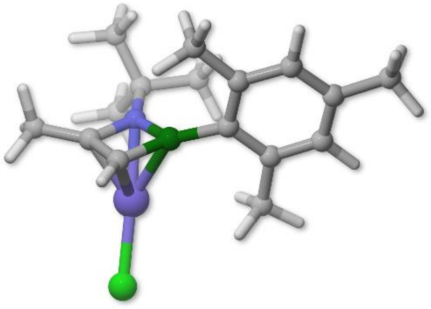

1 (+36.8)

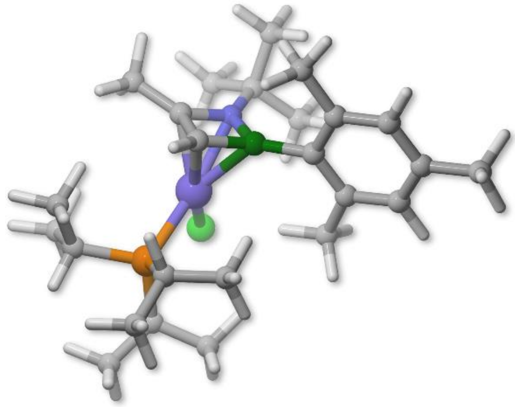

1A (0.0)

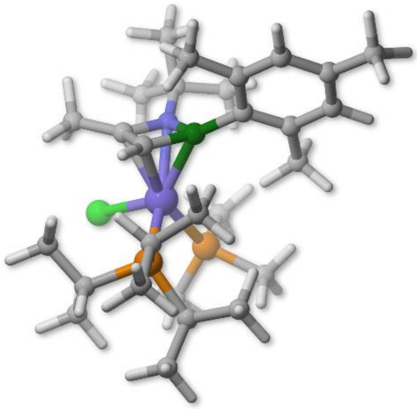

1AB (+7.8)

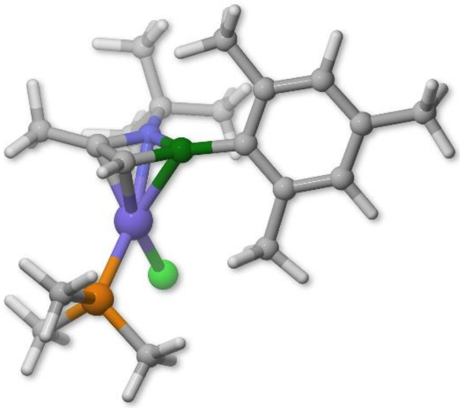

1B (+2.8)

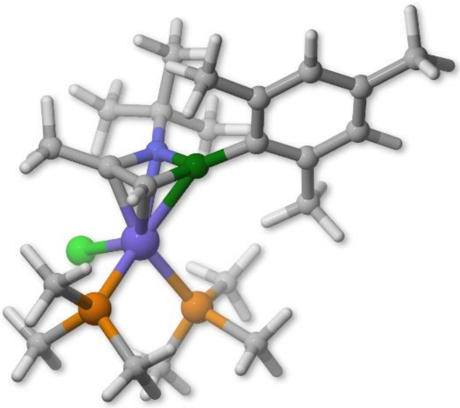

$1B_2$ (+0.4)

Figure S143. Images of the molecular structures of all species of type **1** taken into account in the free energy map. Geometries were optimized at the PBE0-D3(BJ)/def2-SVP,def2-TZVP(Rh) level of theory. The number in parenthesis are the respective free energies obtained at the PBE0-D3(BJ)/def2-QZVPP+SMD(Benzene) level.

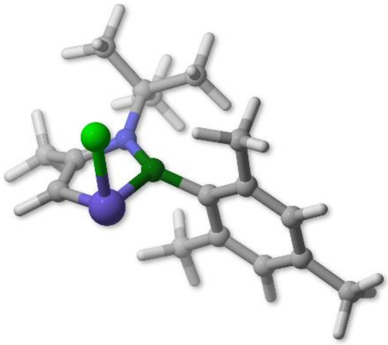

2 (+59.9)

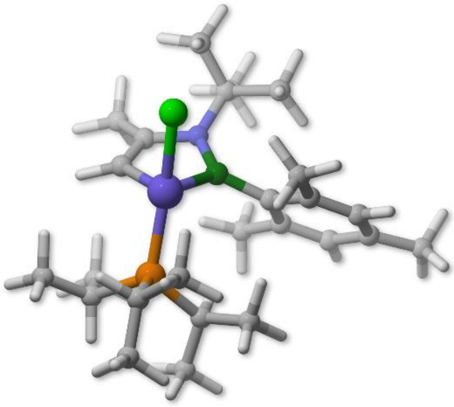

2A (+20.4)

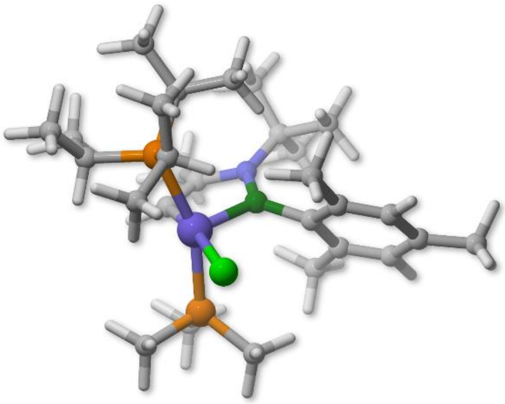

2AB (+6.5)

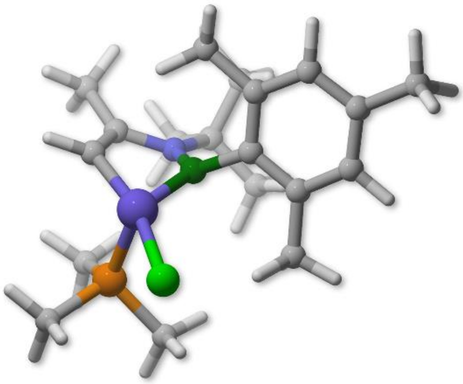

2B (+17.2)

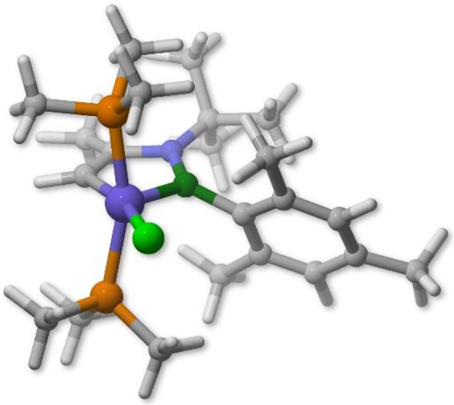

$2B_2$ (–9.1)

Figure S144. Images of the molecular structures of all species of type **2** taken into account in the free energy map. Geometries were optimized at the PBE0-D3(BJ)/def2-SVP,def2-TZVP(Rh) level of theory. The number in parenthesis are the respective free energies obtained at the PBE0-D3(BJ)/def2-QZVPP+SMD(Benzene) level.

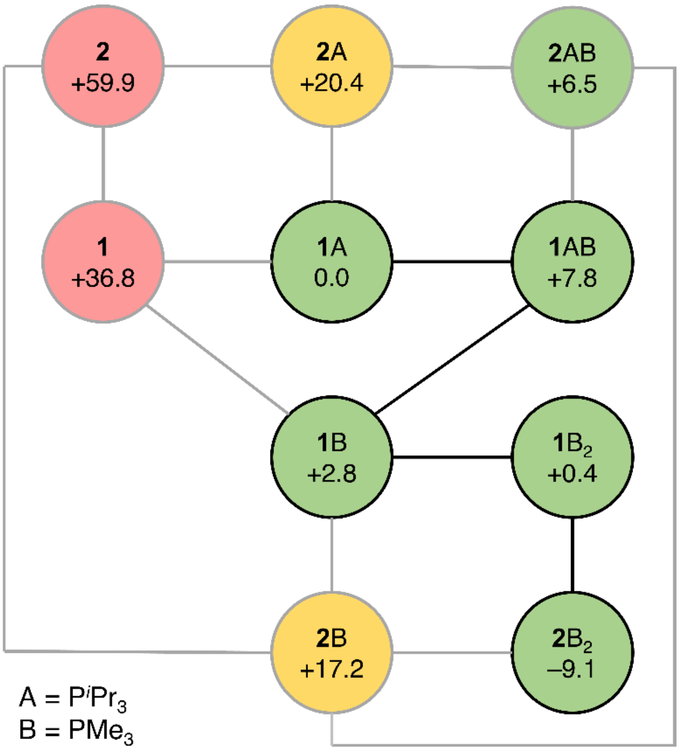

Figure S145. Free energy map illustrating distinct pathways connecting **1A** and **2B**$_2$. Each circle represents an intermediate, whose free energy (kcal mol^{-1}) is also shown. Green circles are low-energy intermediates whose energies are at most 10 kcal mol^{-1} higher than that of **1A**. Yellow circles represent intermediates with moderate free energies. Red circles indicate high-energy intermediates. The black lines highlight the thermodynamically preferred pathway connecting **1A** and **2B**$_2$. All energies are at the PBE0-D3(BJ)/def2-QZVPP+SMD level of theory.

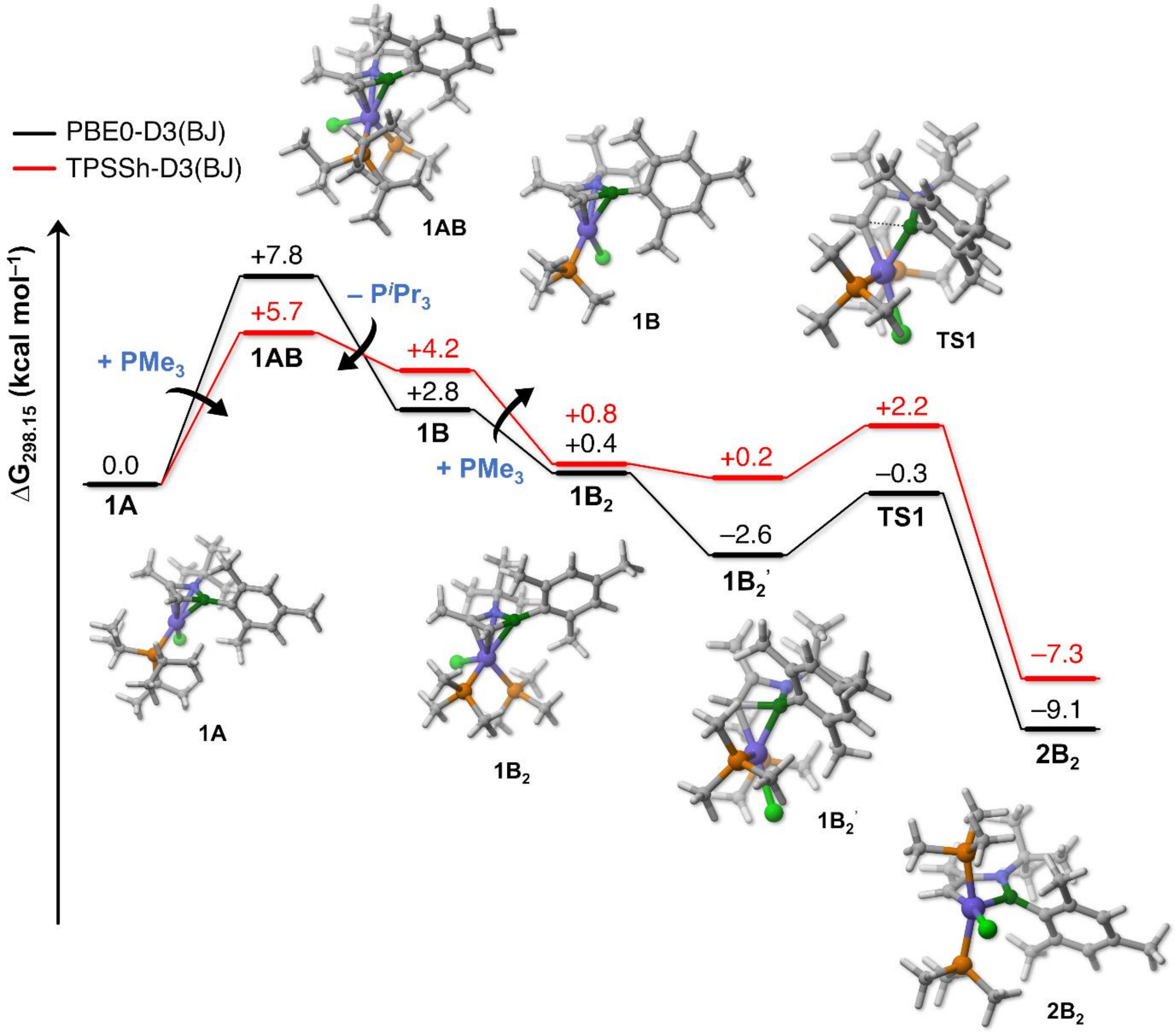

Figure S146. Relative Gibbs free energy profile (T = 298.15 K) of a proposed mechanistic pathway connecting **1A** and **2B$_2$** at the PBE0-D3(BJ)/def2-QZVPP+SMD (black curve) and TPSSh-D3(BJ)/def2-QZVPP+SMD (red curve) levels of theory.

Cartesian coordinates

PMe_3

E(PBE0-D3(BJ)/def2-QZVPP+SMD): -460.900770 E_h

E(TPSSh-D3(BJ)/def2-QZVPP+SMD): -461.217266 E_h

ΔΔG(298): 0.086349 E_h

Lowest frequency: 187.58 cm^{-1}

P	1.563305000	12.888692000	9.181026000
C	2.688000000	13.377363000	7.794656000
H	3.245086000	12.496434000	7.440349000
H	3.422815000	14.111218000	8.158511000
H	2.137841000	13.814817000	6.944224000
C	0.380781000	11.819362000	8.244674000
H	-0.003795000	12.307638000	7.333585000
H	-0.469862000	11.559535000	8.892869000
H	0.879571000	10.880242000	7.959920000
C	0.552072000	14.435594000	9.283805000
H	0.180827000	14.759198000	8.296725000
H	1.162190000	15.243230000	9.715828000
H	-0.308894000	14.273038000	9.949322000

P*i*Pr_3

E(PBE0-D3(BJ)/def2-QZVPP+SMD): -696.594465 E_h

E(TPSSh-D3(BJ)/def2-QZVPP+SMD): -697.229504 E_h

ΔΔG(298.15): 0.248698 E_h

Lowest frequency: 53.55 cm^{-1}

P	6.142848000	3.328984000	3.225734000
C	4.449714000	2.497452000	3.153518000
H	3.832087000	3.218522000	3.719467000
C	4.462397000	1.202240000	3.962107000

H	3.434217000	0.852916000	4.152231000
H	4.961491000	1.327964000	4.935355000
H	4.980340000	0.398947000	3.415535000
C	3.783276000	2.288859000	1.797609000
H	2.768719000	1.877037000	1.936328000
H	4.337109000	1.577035000	1.172176000
H	3.677250000	3.224454000	1.230179000
C	7.380921000	2.131272000	2.483681000
H	8.214487000	2.820064000	2.260361000
C	7.901335000	1.166973000	3.546394000
H	8.780244000	0.618007000	3.170089000
H	7.146821000	0.419868000	3.830853000
H	8.196198000	1.704434000	4.460263000
C	7.017263000	1.413897000	1.189777000
H	7.902498000	0.907815000	0.768747000
H	6.633801000	2.104633000	0.424546000
H	6.255545000	0.637328000	1.357506000
C	5.998106000	4.630541000	1.880661000
H	5.626148000	4.161729000	0.952847000
C	7.349639000	5.277810000	1.589228000
H	7.222215000	6.133596000	0.906471000
H	8.057944000	4.585928000	1.111386000
H	7.815044000	5.659678000	2.513094000
C	4.995448000	5.687524000	2.337315000
H	4.862518000	6.460544000	1.562794000
H	5.351534000	6.186164000	3.253205000
H	4.003057000	5.264452000	2.556939000

1

E(PBE0-D3(BJ)/def2-QZVPP+SMD): -1274.000389 E_h

E(TPSSh-D3(BJ)/def2-QZVPP+SMD): -1275.083890 E_h

ΔΔG(298.15): 0.321545 E_h

Lowest frequency: 27.48 cm^{-1}

C	5.478500000	5.240764000	6.342528000
N	6.480362000	5.048011000	7.341453000
B	6.199537000	3.536955000	7.339958000
Cl	7.482134000	3.545393000	3.414921000
C	4.793646000	6.481405000	5.905865000
H	4.293859000	6.281982000	4.947784000
H	5.488893000	7.318378000	5.764902000
H	4.022121000	6.781678000	6.633187000
C	5.158231000	3.835211000	6.227324000
H	4.404421000	3.361647000	5.599741000
Rh	7.024296000	4.313377000	5.484507000
C	6.249940000	6.804096000	9.015617000
H	6.802013000	7.478007000	9.687564000
H	5.642776000	6.124438000	9.632483000
H	5.572335000	7.418818000	8.405307000
C	8.184045000	5.222049000	9.044829000
H	8.788308000	5.912241000	9.652625000
H	8.866065000	4.604706000	8.441996000
H	7.628963000	4.552156000	9.715626000
C	7.235935000	6.016146000	8.151675000
C	5.831281000	2.196382000	9.471060000
C	6.649764000	2.423862000	8.343443000
C	8.047283000	6.954952000	7.257741000
H	8.625387000	7.653601000	7.881583000
H	7.413216000	7.549100000	6.585456000
H	8.755910000	6.384206000	6.637725000

C	6.210548000	1.255476000	10.433656000
H	5.562436000	1.088483000	11.299791000
C	7.397474000	0.531101000	10.319011000
C	8.185057000	0.741711000	9.183552000
H	9.106072000	0.163235000	9.063360000
C	7.832461000	1.665562000	8.196671000
C	8.718143000	1.817036000	6.992046000
H	9.590581000	1.151653000	7.052242000
H	9.099768000	2.846707000	6.884952000
H	8.174793000	1.585690000	6.062576000
C	4.569929000	2.988114000	9.682612000
H	3.832926000	2.420400000	10.268849000
H	4.103807000	3.278923000	8.729575000
H	4.775816000	3.918472000	10.239555000
C	7.837817000	-0.417100000	11.396587000
H	8.462750000	-1.227557000	10.993965000
H	6.981114000	-0.869400000	11.917590000
H	8.438281000	0.112232000	12.156128000

1A

E(PBE0-D3(BJ)/def2-QZVPP+SMD): -1970.677980 E_h

E(TPSSh-D3(BJ)/def2-QZVPP+SMD): -1972.397863 E_h

ΔΔG(298.15): 0.594676

Lowest frequency: 22.47 cm^{-1}

C	5.690609000	5.550545000	6.280383000
P	6.217524000	3.266574000	3.305181000
N	6.763977000	5.159363000	7.142135000
B	6.117815000	3.769879000	7.242689000
Cl	9.136006000	4.564104000	4.517720000
C	5.289875000	6.918523000	5.863551000

H	4.448915000	6.845752000	5.160969000
H	6.113445000	7.455981000	5.374406000
H	4.956076000	7.509551000	6.732627000
C	5.033171000	4.279869000	6.271192000
H	4.078209000	4.016527000	5.822799000
Rh	6.900498000	4.294591000	5.228365000
C	7.050690000	6.403165000	9.207528000
H	7.747110000	6.999857000	9.815778000
H	6.718180000	5.544621000	9.810346000
H	6.174077000	7.029869000	8.980739000
C	8.885995000	4.976119000	8.262159000
H	9.624058000	5.492977000	8.893331000
H	9.378421000	4.644570000	7.336376000
H	8.518922000	4.096333000	8.808572000
C	7.739128000	5.926369000	7.926263000
C	5.401304000	2.476627000	9.310329000
C	6.330329000	2.585306000	8.244205000
C	8.286061000	7.106542000	7.125684000
H	9.112312000	7.569615000	7.684913000
H	7.528221000	7.883920000	6.957594000
H	8.676688000	6.756718000	6.158179000
C	5.524206000	1.444096000	10.237526000
H	4.804081000	1.379215000	11.060124000
C	6.539721000	0.485491000	10.142207000
C	7.438763000	0.594257000	9.085286000
H	8.240004000	-0.145624000	8.986391000
C	7.355769000	1.626812000	8.142058000
C	8.383061000	1.674631000	7.048814000
H	9.382167000	1.910743000	7.448197000
H	8.148187000	2.446150000	6.299346000
H	8.459702000	0.705686000	6.531381000

C	4.273399000	3.462058000	9.452581000
H	3.712273000	3.292505000	10.382540000
H	3.568724000	3.384690000	8.609665000
H	4.634001000	4.502294000	9.459625000
C	6.644835000	-0.620381000	11.151560000
H	7.496551000	-1.281412000	10.937745000
H	5.732036000	-1.238148000	11.164107000
H	6.775026000	-0.220018000	12.169754000
C	4.948647000	1.941256000	3.626345000
H	4.103831000	2.545058000	3.999912000
C	5.351223000	1.005745000	4.762669000
H	4.495436000	0.371184000	5.043243000
H	5.665559000	1.564108000	5.655054000
H	6.176175000	0.338984000	4.476741000
C	4.484311000	1.178228000	2.392366000
H	3.609781000	0.555566000	2.641468000
H	5.268298000	0.500127000	2.023664000
H	4.193525000	1.843110000	1.565157000
C	7.617949000	2.582802000	2.282390000
H	8.344014000	3.408565000	2.380574000
C	8.259888000	1.375852000	2.957847000
H	9.232031000	1.162924000	2.486642000
H	7.640806000	0.470467000	2.859630000
H	8.448903000	1.564113000	4.024181000
C	7.353730000	2.335236000	0.802162000
H	8.293941000	2.024383000	0.318320000
H	7.007787000	3.235842000	0.274993000
H	6.619045000	1.536082000	0.627986000
C	5.367049000	4.444962000	2.139996000
H	5.222535000	3.902809000	1.189913000
C	6.277253000	5.644332000	1.894089000

H	5.825437000	6.320025000	1.149915000
H	7.272330000	5.353186000	1.528839000
H	6.430681000	6.208202000	2.827468000
C	4.001574000	4.883828000	2.652391000
H	3.579095000	5.652269000	1.985573000
H	4.082154000	5.322563000	3.658259000
H	3.280352000	4.054288000	2.697940000

1AB

E(PBE0-D3(BJ)/def2-QZVPP+SMD): -2431.592893 E_h

E(TPSSh-D3(BJ)/def2-QZVPP+SMD): -2433.632648 E_h

ΔΔG(298.15): 0.707554 E_h

Lowest frequency: 14.03 cm^{-1}

C	5.731192000	5.660653000	6.654880000
P	5.624512000	3.417937000	3.569625000
N	6.921885000	5.180477000	7.285241000
B	6.296834000	3.764695000	7.263703000
Cl	7.405339000	6.820017000	4.186429000
C	5.239248000	7.053248000	6.510455000
H	4.322757000	7.036443000	5.905861000
H	5.972665000	7.698030000	6.009684000
H	4.984440000	7.466859000	7.499452000
C	5.088076000	4.418437000	6.538167000
H	4.082413000	4.239525000	6.175050000
Rh	6.825442000	4.503803000	5.209892000
C	6.931245000	6.110656000	9.531992000
H	7.513065000	6.681586000	10.271231000
H	6.661624000	5.142055000	9.978228000
H	6.005765000	6.669624000	9.330125000
C	8.967760000	5.050927000	8.591829000

H	9.579275000	5.561090000	9.349968000
H	9.586387000	4.888304000	7.703011000
H	8.659531000	4.078762000	8.997769000
C	7.755738000	5.908053000	8.254433000
C	5.374860000	2.323721000	9.178627000
C	6.437907000	2.549499000	8.262723000
C	8.213959000	7.257486000	7.698231000
H	9.020551000	7.659015000	8.328885000
H	7.401857000	7.996206000	7.692564000
H	8.573276000	7.158623000	6.664225000
C	5.409497000	1.224601000	10.038089000
H	4.574223000	1.071553000	10.729724000
C	6.462493000	0.306491000	10.028998000
C	7.518310000	0.546309000	9.152580000
H	8.366176000	-0.146986000	9.131947000
C	7.522003000	1.645717000	8.286309000
C	8.701482000	1.830430000	7.380402000
H	9.549272000	2.290141000	7.914727000
H	8.432483000	2.490216000	6.543310000
H	9.055792000	0.866408000	6.984256000
C	4.172428000	3.225533000	9.252422000
H	3.561775000	2.990759000	10.136132000
H	3.532502000	3.107175000	8.364501000
H	4.455481000	4.287794000	9.299590000
C	6.421160000	-0.921413000	10.890287000
H	7.427388000	-1.246498000	11.193041000
H	5.957960000	-1.760573000	10.343837000
H	5.825415000	-0.756819000	11.800091000
C	4.132387000	2.476267000	4.222001000
H	3.522951000	3.300987000	4.619330000
C	4.451258000	1.532947000	5.379489000

H	3.546115000	1.354701000	5.981357000
H	5.225925000	1.928976000	6.047677000
H	4.794146000	0.555597000	5.016067000
C	3.286899000	1.767405000	3.170331000
H	2.350248000	1.416642000	3.632428000
H	3.795652000	0.876861000	2.774769000
H	3.014143000	2.411804000	2.323465000
C	6.484872000	2.192971000	2.431471000
H	7.445885000	2.694721000	2.246398000
C	6.778642000	0.886094000	3.161699000
H	7.540048000	0.307004000	2.614869000
H	5.879971000	0.257024000	3.222551000
H	7.141147000	1.056053000	4.185990000
C	5.868597000	1.915485000	1.058835000
H	6.590386000	1.339417000	0.456360000
H	5.632634000	2.827017000	0.495909000
H	4.951448000	1.316023000	1.121574000
C	4.832377000	4.629285000	2.388502000
H	4.144989000	4.034010000	1.762656000
C	5.848935000	5.330711000	1.493499000
H	5.315579000	5.988010000	0.787284000
H	6.461678000	4.639969000	0.897094000
H	6.516821000	5.956658000	2.105294000
C	4.029531000	5.669991000	3.161019000
H	3.501156000	6.331999000	2.456029000
H	4.714016000	6.288232000	3.761998000
H	3.272437000	5.222366000	3.822266000
P	8.947906000	4.039303000	4.246909000
C	9.684363000	2.362511000	4.024706000
H	9.105364000	1.789730000	3.292148000
H	9.695061000	1.804763000	4.968225000

H	10.714965000	2.461090000	3.648788000
C	9.227264000	4.683036000	2.548556000
H	10.268760000	4.483510000	2.253476000
H	9.016732000	5.760241000	2.556163000
H	8.555884000	4.203126000	1.826546000
C	10.314798000	4.872396000	5.140130000
H	11.227887000	4.871445000	4.525723000
H	10.519966000	4.346013000	6.081027000
H	10.011934000	5.905499000	5.354562000

1B

E(PBE0-D3(BJ)/def2-QZVPP+SMD): -1734.977790 E_h

E(TPSSh-D3(BJ)/def2-QZVPP+SMD): -1736.376795 E_h

ΔΔG(298.15): 0.430267 E_h

Lowest frequency: 21.80 cm^{-1}

C	5.186104000	5.153661000	6.382249000
P	6.265739000	3.379713000	3.329574000
N	6.288837000	5.019677000	7.305334000
B	5.997841000	3.519592000	7.378142000
Cl	8.904282000	4.614362000	4.701803000
C	4.477608000	6.377309000	5.931301000
H	3.818005000	6.104841000	5.095596000
H	5.173793000	7.152323000	5.584724000
H	3.851348000	6.800849000	6.733113000
C	4.853911000	3.762265000	6.360593000
H	4.015659000	3.270773000	5.871655000
Rh	6.668515000	4.236088000	5.369109000
C	6.129210000	6.751488000	9.029043000
H	6.713268000	7.439793000	9.659075000
H	5.603061000	6.045857000	9.687281000

H	5.379296000	7.349798000	8.490812000
C	8.136553000	5.261732000	8.826393000
H	8.779599000	5.971718000	9.367738000
H	8.763837000	4.683009000	8.132657000
H	7.695887000	4.563777000	9.551234000
C	7.060459000	6.021390000	8.057989000
C	6.030215000	2.349635000	9.640015000
C	6.556172000	2.405922000	8.331811000
C	7.727792000	7.021984000	7.117055000
H	8.325453000	7.734808000	7.705990000
H	6.992627000	7.601738000	6.541022000
H	8.388165000	6.502034000	6.407496000
C	6.511958000	1.409469000	10.553461000
H	6.085426000	1.383068000	11.561239000
C	7.516703000	0.504630000	10.211524000
C	8.006492000	0.541576000	8.904267000
H	8.772597000	-0.180069000	8.603493000
C	7.542475000	1.463462000	7.960681000
C	8.093637000	1.406091000	6.565320000
H	8.798127000	0.570023000	6.447875000
H	8.615159000	2.333201000	6.278441000
H	7.280034000	1.269985000	5.835505000
C	4.957220000	3.301408000	10.093864000
H	4.209044000	2.785956000	10.715296000
H	4.431467000	3.766111000	9.246546000
H	5.381408000	4.113491000	10.708665000
C	8.051397000	-0.464270000	11.225942000
H	8.669806000	-1.243235000	10.757648000
H	7.238336000	-0.960486000	11.778930000
H	8.676361000	0.055802000	11.971538000
C	4.579516000	2.761888000	2.975173000

H	3.860779000	3.586542000	3.083489000
C	7.341210000	1.973226000	2.889722000
H	8.378364000	2.284826000	3.084402000
C	6.554414000	4.583891000	1.988097000
H	6.360419000	4.136078000	1.000703000
H	4.319841000	1.974066000	3.695639000
H	4.513534000	2.353390000	1.954675000
H	7.219175000	1.674782000	1.837174000
H	7.106941000	1.119519000	3.540149000
H	5.900601000	5.454556000	2.136139000
H	7.598607000	4.921704000	2.058998000

1B$_2$

E(PBE0-D3(BJ)/def2-QZVPP+SMD): -2195.906039 E_h

E(TPSSh-D3(BJ)/def2-QZVPP+SMD): -2197.623329 E_h

ΔΔG(298.15): 0.540329 E_h

Lowest frequency: 3.95 cm^{-1}

C	5.501126000	5.241629000	6.522311000
P	5.656673000	4.190789000	3.284065000
N	6.694891000	4.972001000	7.314360000
B	6.322207000	3.505506000	7.325174000
Cl	7.764252000	6.679023000	4.420237000
C	4.723706000	6.504547000	6.424172000
H	3.877871000	6.335853000	5.743598000
H	5.334934000	7.322296000	6.019201000
H	4.303842000	6.812057000	7.397347000
C	5.161240000	3.842931000	6.393371000
H	4.263149000	3.393380000	5.981778000
Rh	6.818544000	4.522058000	5.182325000
C	6.750603000	5.656271000	9.649336000

H	7.276717000	6.264001000	10.400727000
H	6.764891000	4.608155000	9.981998000
H	5.704893000	5.997855000	9.612573000
C	8.857184000	5.301066000	8.355355000
H	9.428565000	5.897806000	9.080136000
H	9.342360000	5.386026000	7.373606000
H	8.885775000	4.254262000	8.684249000
C	7.423001000	5.811450000	8.280578000
C	5.595693000	2.111349000	9.315847000
C	6.635308000	2.381194000	8.381439000
C	7.437399000	7.281438000	7.872285000
H	8.123864000	7.828674000	8.534566000
H	6.448293000	7.746536000	7.977948000
H	7.774805000	7.395851000	6.831972000
C	5.805471000	1.193621000	10.342491000
H	4.999364000	1.009459000	11.060224000
C	7.009336000	0.496287000	10.483207000
C	8.015371000	0.750933000	9.556792000
H	8.967937000	0.218271000	9.639805000
C	7.846306000	1.679160000	8.521050000
C	8.980615000	1.886165000	7.562285000
H	9.874116000	2.287506000	8.067220000
H	8.689685000	2.588880000	6.771497000
H	9.282718000	0.934738000	7.096211000
C	4.251636000	2.785137000	9.239988000
H	3.627771000	2.508991000	10.102546000
H	3.710572000	2.490230000	8.328316000
H	4.334905000	3.882341000	9.215299000
C	7.171530000	-0.513881000	11.579274000
H	8.225981000	-0.771529000	11.751526000
H	6.639666000	-1.447353000	11.329465000

H	6.747980000	-0.144142000	12.525530000
C	3.829787000	4.209009000	3.465815000
H	3.512548000	5.140136000	3.954802000
C	5.840944000	2.603172000	2.375790000
H	6.855835000	2.498465000	1.970849000
C	5.860541000	5.449319000	1.976963000
H	5.220363000	5.217159000	1.112390000
P	8.828175000	3.611866000	4.226386000
C	9.080503000	1.808214000	3.958185000
H	8.318975000	1.442132000	3.256984000
H	8.959732000	1.260260000	4.900239000
H	10.075545000	1.598158000	3.533741000
C	9.206994000	4.229033000	2.533180000
H	10.231244000	3.948668000	2.243135000
H	9.101517000	5.323659000	2.550667000
H	8.511260000	3.809302000	1.794907000
C	10.386391000	4.130738000	5.049341000
H	11.263441000	3.788200000	4.479068000
H	10.444924000	3.742221000	6.073687000
H	10.370452000	5.230404000	5.085553000
H	5.659089000	1.775152000	3.076119000
H	5.125548000	2.542685000	1.541556000
H	6.909826000	5.512000000	1.666386000
H	5.594888000	6.425841000	2.404112000
H	3.354249000	4.143674000	2.475416000
H	3.496360000	3.360925000	4.077122000

2

E(PBE0-D3(BJ)/def2-QZVPP+SMD): -1273.963495 E_h

E(TPSSh-D3(BJ)/def2-QZVPP+SMD): -1275.045605 E_h

ΔΔG(298.15): 0.321487 E_h

Lowest frequency: 24.57 cm^{-1}

Rh	2.064469000	11.928019000	10.953095000
N	4.677752000	10.720421000	10.774376000
B	3.289232000	10.392170000	10.749865000
C	3.676224000	12.811803000	10.451246000
H	3.639612000	13.864310000	10.137524000
C	4.813787000	12.072146000	10.378204000
C	6.053520000	12.571359000	9.702429000
H	6.273802000	11.979574000	8.799812000
H	6.945872000	12.530473000	10.344295000
H	5.908207000	13.614842000	9.393063000
C	5.155272000	8.642287000	11.934799000
H	4.650980000	8.033816000	11.170609000
H	4.422097000	8.907598000	12.710587000
H	5.941133000	8.031412000	12.401914000
Cl	2.159993000	12.026189000	13.186308000
C	5.773894000	9.901030000	11.329581000
C	6.779445000	9.471195000	10.257989000
H	6.272723000	8.902703000	9.463192000
H	7.542735000	8.817833000	10.707971000
H	7.300650000	10.319177000	9.797143000
C	6.463557000	10.679813000	12.456801000
H	7.204187000	10.041327000	12.962162000
H	5.719210000	11.010352000	13.194958000
H	6.991819000	11.568913000	12.088585000
C	2.441310000	9.189313000	10.228233000
C	1.568298000	8.416480000	11.029004000

C	0.698224000	7.508204000	10.417446000
H	0.025074000	6.919549000	11.048318000
C	0.658122000	7.333468000	9.035430000
C	1.541148000	8.086206000	8.254678000
H	1.535700000	7.962750000	7.166728000
C	2.424612000	9.000768000	8.819752000
C	1.590257000	8.505980000	12.525155000
H	2.380081000	7.857600000	12.937969000
H	0.639264000	8.166508000	12.959709000
H	1.790012000	9.527781000	12.884272000
C	-0.306403000	6.385393000	8.389576000
H	0.217953000	5.676399000	7.729194000
H	-1.033010000	6.932534000	7.766152000
H	-0.872228000	5.806093000	9.133337000
C	3.332323000	9.790628000	7.921852000
H	3.212476000	10.878170000	8.071261000
H	3.126157000	9.571848000	6.864234000
H	4.390839000	9.561539000	8.121802000

2A

E(PBE0-D3(BJ)/def2-QZVPP+SMD): -1970.646038 E_h

E(TPSSh-D3(BJ)/def2-QZVPP+SMD): -1972.365675 E_h

ΔΔG(298.15): 0.595201 E_h

Lowest frequency: 14.83 cm^{-1}

Rh	2.436951000	12.497162000	10.795465000
N	4.533205000	10.591039000	10.461436000
B	3.104330000	10.664911000	10.372426000
C	4.249822000	12.894518000	10.200643000
H	4.545756000	13.895112000	9.870071000
C	5.109171000	11.857791000	10.175428000

C	6.503053000	11.985449000	9.638970000
H	6.614074000	11.416523000	8.700381000
H	7.277702000	11.624774000	10.330923000
H	6.715526000	13.041403000	9.420435000
C	4.373710000	8.444434000	11.692573000
H	3.851335000	7.886266000	10.906583000
H	3.625156000	8.887587000	12.365294000
H	4.971537000	7.730716000	12.278643000
Cl	2.926824000	12.349073000	13.051851000
C	5.309297000	9.515231000	11.132888000
C	6.284330000	8.818296000	10.177894000
H	5.734230000	8.323041000	9.364761000
H	6.845706000	8.044935000	10.724532000
H	7.013623000	9.502885000	9.729580000
C	6.040923000	10.120085000	12.339915000
H	6.549689000	9.323130000	12.904052000
H	5.314950000	10.618402000	12.997774000
H	6.795889000	10.863647000	12.056334000
C	2.172482000	9.468061000	9.966428000
C	1.166216000	8.926158000	10.801142000
C	0.537846000	7.736124000	10.436506000
H	-0.206492000	7.300247000	11.110728000
C	0.855359000	7.064168000	9.249297000
C	1.771147000	7.663264000	8.386868000
H	2.009796000	7.177783000	7.434695000
C	2.428956000	8.851411000	8.723358000
C	0.768416000	9.606918000	12.080346000
H	1.626331000	10.019325000	12.629833000
H	0.214210000	8.927238000	12.743096000
H	0.107176000	10.466552000	11.873389000
C	0.273875000	5.714588000	8.945365000

H	0.368999000	5.461993000	7.879265000
H	-0.787838000	5.648379000	9.228820000
H	0.806263000	4.934583000	9.513979000
C	3.407000000	9.418766000	7.728030000
H	2.946595000	9.466121000	6.727651000
H	4.301356000	8.782527000	7.637459000
H	3.754275000	10.423578000	8.000402000
P	1.386061000	13.118458000	8.874864000
C	0.013521000	14.155198000	9.622196000
H	0.601121000	14.987826000	10.046530000
C	-0.659007000	13.461142000	10.808664000
H	-1.281583000	14.185905000	11.358297000
H	0.071616000	13.068173000	11.538288000
H	-1.308507000	12.631449000	10.498513000
C	-1.003917000	14.749077000	8.658768000
H	-1.661032000	15.451004000	9.198623000
H	-1.647685000	13.973975000	8.218459000
H	-0.531348000	15.307322000	7.836772000
C	0.636192000	11.841889000	7.734606000
H	1.480924000	11.150236000	7.592208000
C	-0.476611000	11.057331000	8.419730000
H	-0.695776000	10.145082000	7.843361000
H	-1.404053000	11.646136000	8.476865000
C	0.185969000	12.335420000	6.360421000
H	-0.221809000	11.485181000	5.789742000
H	1.006208000	12.761531000	5.766367000
H	-0.611377000	13.089937000	6.427709000
C	2.221497000	14.306845000	7.708552000
H	1.469439000	14.542824000	6.936982000
C	3.410287000	13.631140000	7.026063000
H	3.879085000	14.321690000	6.306336000

H	3.110792000	12.729807000	6.470279000
H	4.167564000	13.328860000	7.761677000
C	2.603742000	15.610257000	8.406585000
H	3.217259000	16.230174000	7.733946000
H	3.187494000	15.435564000	9.322886000
H	1.721038000	16.205852000	8.681617000
H	-0.195216000	10.733102000	9.429758000

2AB

E(PBE0-D3(BJ)/def2-QZVPP+SMD): -2431.593403 E_h

E(TPSSh-D3(BJ)/def2-QZVPP+SMD): -2433.630659 E_h

ΔΔG(298.15): 0.706085 E_h

Lowest frequency: 19.73 cm^{-1}

Rh	2.266240000	12.543397000	11.304706000
P	2.891304000	12.792442000	13.599408000
N	4.609060000	10.863660000	10.614218000
B	3.175162000	10.834559000	10.749344000
C	4.090523000	13.130862000	10.769811000
H	4.304990000	14.182978000	10.532002000
C	5.041945000	12.219953000	10.476090000
P	1.452154000	13.123316000	9.227290000
C	6.330174000	12.612380000	9.808034000
H	6.303596000	12.407842000	8.723089000
H	7.230140000	12.118005000	10.196437000
H	6.467207000	13.695815000	9.936664000
C	4.889315000	8.406930000	11.052052000
H	4.236747000	8.017154000	10.262258000
H	4.292838000	8.502512000	11.968294000
H	5.675853000	7.662359000	11.244491000
Cl	-0.100395000	12.085512000	11.741510000

C	5.568309000	9.726889000	10.663449000
C	6.250010000	9.476254000	9.308176000
H	5.510711000	9.172600000	8.556489000
H	6.977168000	8.656173000	9.408832000
H	6.788192000	10.349939000	8.925520000
C	6.619735000	10.001250000	11.749191000
H	7.393874000	9.219560000	11.728563000
H	6.148890000	9.987177000	12.739982000
H	7.122410000	10.968264000	11.636154000
C	2.295903000	9.593060000	10.312530000
C	1.528383000	8.804774000	11.200205000
C	1.002015000	7.583692000	10.775304000
H	0.446701000	6.974059000	11.496428000
C	1.198815000	7.096692000	9.482456000
C	1.876082000	7.921197000	8.584267000
H	2.031379000	7.580016000	7.555602000
C	2.405635000	9.156194000	8.968273000
C	1.298398000	9.188774000	12.631099000
H	1.674569000	10.195014000	12.834641000
H	1.779379000	8.475222000	13.319560000
H	0.221252000	9.201075000	12.854884000
C	0.731821000	5.721951000	9.100188000
H	0.917815000	5.510836000	8.037376000
H	-0.343280000	5.584815000	9.298902000
H	1.264105000	4.958026000	9.688772000
C	3.085724000	9.972829000	7.902848000
H	2.341744000	10.480379000	7.266394000
H	3.683243000	9.337949000	7.231673000
H	3.745213000	10.738557000	8.327979000
C	4.347454000	11.822168000	14.241718000
C	1.408709000	12.457905000	14.695830000

C	3.384594000	14.593270000	13.862601000
C	2.594887000	13.596949000	7.878538000
H	3.136394000	12.716611000	7.511986000
H	3.332303000	14.312373000	8.266961000
H	2.037729000	14.056377000	7.047383000
C	0.165367000	12.122102000	8.409909000
H	-0.201851000	12.627741000	7.503888000
H	-0.642730000	11.992310000	9.141926000
H	0.553277000	11.127306000	8.157409000
C	0.572655000	14.707526000	9.525319000
H	0.157031000	15.111275000	8.588439000
H	1.267136000	15.440967000	9.960734000
H	-0.235595000	14.509087000	10.242845000
C	0.326930000	13.531406000	14.555460000
H	0.482109000	14.368541000	15.250211000
H	0.247728000	13.919072000	13.532420000
H	-0.651221000	13.082546000	14.787888000
C	1.645596000	12.129250000	16.164512000
H	2.048739000	12.986854000	16.722152000
H	0.682005000	11.862501000	16.628458000
H	2.320731000	11.276696000	16.312403000
C	3.171802000	15.160616000	15.267504000
H	3.549828000	16.196067000	15.298148000
H	2.116844000	15.187441000	15.558255000
H	3.716746000	14.597170000	16.034083000
C	4.812980000	14.880777000	13.398461000
H	5.558537000	14.453717000	14.082022000
H	5.013841000	14.480333000	12.397844000
H	4.978457000	15.969954000	13.385052000
C	4.052623000	10.329614000	14.251573000
H	4.944524000	9.773084000	14.580413000

H	3.236963000	10.069130000	14.940328000
H	3.776662000	9.968907000	13.256617000
C	4.957838000	12.246806000	15.577496000
H	5.854151000	11.636009000	15.770180000
H	5.276233000	13.295292000	15.599980000
H	4.270812000	12.086103000	16.418156000
H	0.998377000	11.564128000	14.197335000
H	2.699998000	15.124520000	13.177985000
H	5.082773000	12.011685000	13.438928000

2B

E(PBE0-D3(BJ)/def2-QZVPP+SMD): -1734.955494 E_h

E(TPSSh-D3(BJ)/def2-QZVPP+SMD): -1736.353106 E_h

ΔΔG(298.15): 0.430947 E_h

Lowest frequency: 23.89 cm^{-1}

Rh	1.479586000	12.279071000	10.204398000
N	4.186500000	11.066538000	10.411489000
B	2.785159000	10.808456000	10.203629000
C	3.006304000	12.808146000	11.371566000
H	2.857481000	13.614957000	12.104354000
C	4.198774000	12.164394000	11.315384000
P	2.346766000	13.473697000	8.596333000
C	5.354354000	12.479716000	12.212408000
H	6.185461000	12.970273000	11.681583000
H	5.765331000	11.583642000	12.703777000
H	5.010573000	13.167891000	12.995917000
C	4.935664000	9.572550000	8.660681000
H	4.827272000	10.357695000	7.896447000
H	3.997915000	9.004690000	8.719960000
H	5.722822000	8.879641000	8.332700000

Cl	-0.625356000	12.258422000	9.113947000
C	5.301546000	10.183390000	10.017144000
C	6.609525000	10.958021000	9.822037000
H	6.456005000	11.849036000	9.195055000
H	7.334958000	10.309436000	9.309100000
H	7.066671000	11.273318000	10.766882000
C	5.514637000	9.051303000	11.026079000
H	6.343871000	8.400345000	10.708322000
H	4.609164000	8.433660000	11.110899000
H	5.762487000	9.445968000	12.022634000
C	2.028503000	9.435382000	10.372733000
C	1.998395000	8.857058000	11.665525000
C	1.462901000	7.578122000	11.839655000
H	1.460498000	7.140530000	12.843080000
C	0.944332000	6.844137000	10.775269000
C	0.893881000	7.461346000	9.522055000
H	0.438946000	6.927234000	8.682572000
C	1.403401000	8.741246000	9.305297000
C	2.531501000	9.567874000	12.879778000
H	3.615272000	9.408091000	12.997999000
H	2.042757000	9.186752000	13.787459000
H	2.371145000	10.655661000	12.833491000
C	0.460305000	5.437494000	10.963713000
H	1.053423000	4.736142000	10.356622000
H	-0.589372000	5.321531000	10.648957000
H	0.544165000	5.122466000	12.013571000
C	1.261583000	9.360526000	7.946214000
H	0.624309000	10.257057000	8.010403000
H	0.804505000	8.658373000	7.235798000
H	2.232718000	9.671045000	7.532605000
C	4.113440000	13.918525000	8.616899000

H	4.707617000	13.001059000	8.709424000
H	4.325400000	14.555249000	9.484395000
H	4.372376000	14.446409000	7.686961000
C	2.124938000	12.760563000	6.931800000
H	2.467373000	13.472846000	6.165304000
H	1.058029000	12.532067000	6.801618000
H	2.700906000	11.829483000	6.849180000
C	1.496076000	15.083708000	8.476229000
H	1.913585000	15.682364000	7.652200000
H	1.613584000	15.627464000	9.424204000
H	0.427460000	14.886823000	8.311083000

2B$_2$

E(PBE0-D3(BJ)/def2-QZVPP+SMD): -2195.921711 E_h

E(TPSSh-D3(BJ)/def2-QZVPP+SMD): -2197.636540 E_h

ΔΔG(298.15): 0.540769 E_h

Lowest frequency: 27.66 cm^{-1}

Rh	2.220995000	12.563237000	11.218253000
P	2.799338000	12.624172000	13.443408000
N	4.605215000	10.830256000	10.817131000
B	3.165925000	10.852440000	10.786368000
C	4.112192000	13.099281000	10.869130000
H	4.373772000	14.157412000	10.713076000
C	5.084900000	12.173083000	10.729569000
P	1.511611000	13.258216000	9.121689000
C	6.479485000	12.543603000	10.306837000
H	6.643983000	12.353547000	9.232165000
H	7.284179000	12.024434000	10.842738000
H	6.612271000	13.621705000	10.475107000
C	4.722479000	8.361861000	11.217472000

H	4.130578000	8.019121000	10.361370000
H	4.050288000	8.477944000	12.079509000
H	5.449912000	7.575182000	11.466631000
Cl	-0.147991000	12.559231000	11.803166000
C	5.502115000	9.652541000	10.938184000
C	6.307785000	9.426287000	9.650643000
H	5.629882000	9.308919000	8.794740000
H	6.900211000	8.502836000	9.737632000
H	7.002907000	10.243847000	9.427263000
C	6.438892000	9.816519000	12.145152000
H	7.211506000	9.033357000	12.125194000
H	5.870505000	9.704681000	13.078448000
H	6.950202000	10.784456000	12.182611000
C	2.272236000	9.654046000	10.283279000
C	1.446474000	8.887193000	11.139470000
C	0.893200000	7.692757000	10.681639000
H	0.294797000	7.088965000	11.371534000
C	1.124159000	7.214440000	9.386734000
C	1.864339000	8.019934000	8.523650000
H	2.053890000	7.679366000	7.500527000
C	2.422378000	9.232244000	8.943902000
C	1.191533000	9.318101000	12.551439000
H	2.132478000	9.583787000	13.060030000
H	0.698628000	8.527913000	13.135150000
H	0.556714000	10.219518000	12.563710000
C	0.633490000	5.856550000	8.974070000
H	0.840476000	5.656053000	7.913001000
H	-0.448839000	5.740788000	9.144970000
H	1.135002000	5.072273000	9.563308000
C	3.194769000	10.037952000	7.935782000
H	2.508808000	10.550392000	7.240312000

H	3.841732000	9.397527000	7.317008000
H	3.820102000	10.800909000	8.415862000
C	4.296173000	11.807539000	14.088544000
H	4.139658000	10.720630000	14.125639000
H	4.534093000	12.177775000	15.096762000
H	5.123153000	12.025340000	13.401357000
C	1.531028000	12.228596000	14.697166000
H	0.585776000	12.690959000	14.380807000
H	1.840004000	12.600389000	15.685595000
H	1.372239000	11.143133000	14.744833000
C	3.137958000	14.395406000	13.778886000
H	3.967857000	14.718722000	13.136371000
H	3.395675000	14.566941000	14.835713000
H	2.246562000	14.985708000	13.519507000
C	2.713160000	13.657882000	7.801089000
H	3.208498000	12.748821000	7.440128000
H	3.483024000	14.320074000	8.221014000
H	2.213924000	14.162351000	6.959399000
C	0.191499000	12.292473000	8.314191000
H	-0.182960000	12.796948000	7.410385000
H	-0.611665000	12.189779000	9.058438000
H	0.553634000	11.285802000	8.065172000
C	0.704467000	14.885744000	9.381947000
H	0.324485000	15.300444000	8.434715000
H	1.427015000	15.586426000	9.825541000
H	-0.121408000	14.733615000	10.090965000

1B2‘

E(PBE0-D3(BJ)/def2-QZVPP+SMD): -2195.910206 E_h

E(TPSSh-D3(BJ)/def2-QZVPP+SMD): -2197.623433 E_h

ΔΔG(298): 0.539615 E_h

Lowest frequency: 28.97 cm^{-1}

Rh	2.278486000	12.595975000	11.353379000
P	3.302804000	12.976231000	13.379147000
N	4.658740000	10.666423000	11.524707000
B	3.403011000	10.951729000	10.723133000
C	4.054078000	12.493899000	10.345699000
H	4.188281000	13.135494000	9.470128000
C	5.122090000	11.680106000	10.690016000
P	0.995722000	12.833413000	9.428226000
C	6.494874000	11.813955000	10.115562000
H	6.903847000	10.879445000	9.712075000
H	7.177198000	12.156096000	10.911025000
H	6.494860000	12.574351000	9.324037000
C	4.356666000	8.571628000	12.684902000
H	3.523136000	8.242125000	12.049815000
H	3.946027000	9.135407000	13.533106000
H	4.861004000	7.677482000	13.079532000
Cl	0.203808000	12.889471000	12.514084000
C	5.343784000	9.418561000	11.883773000
C	5.802517000	8.565981000	10.690340000
H	4.956506000	8.322769000	10.033200000
H	6.228180000	7.620663000	11.059497000
H	6.585275000	9.055399000	10.096371000
C	6.547035000	9.747108000	12.771667000
H	7.036784000	8.821528000	13.110200000
H	6.229416000	10.308751000	13.662573000
H	7.300544000	10.342226000	12.236993000

C	2.665339000	9.869658000	9.832807000
C	1.552739000	9.194942000	10.388202000
C	0.846606000	8.260781000	9.618952000
H	-0.008270000	7.747335000	10.071006000
C	1.196315000	7.969132000	8.301842000
C	2.315300000	8.618254000	7.771826000
H	2.635204000	8.378311000	6.752988000
C	3.053009000	9.549323000	8.508549000
C	1.057106000	9.481005000	11.778339000
H	1.851755000	9.836598000	12.446108000
H	0.595850000	8.586591000	12.224239000
H	0.305352000	10.286797000	11.774720000
C	0.390836000	7.015295000	7.466560000
H	1.035424000	6.390178000	6.828930000
H	-0.293365000	7.563752000	6.796244000
H	-0.224057000	6.348571000	8.088972000
C	4.263237000	10.171197000	7.865261000
H	4.463627000	9.717700000	6.884479000
H	5.159723000	10.031675000	8.483417000
H	4.145910000	11.256118000	7.722978000
C	5.101125000	13.320363000	13.463299000
H	5.666121000	12.413436000	13.220191000
H	5.380945000	13.669603000	14.468851000
H	5.347162000	14.097808000	12.727246000
C	3.047289000	11.743299000	14.701148000
H	1.971124000	11.524066000	14.752465000
H	3.406939000	12.106295000	15.676070000
H	3.582616000	10.824305000	14.431195000
C	2.635167000	14.510807000	14.130574000
H	2.827467000	15.351449000	13.447929000
H	3.106750000	14.713451000	15.104757000

H	1.547870000	14.396753000	14.237534000
C	1.728116000	12.766977000	7.750431000
H	2.007081000	11.730304000	7.518644000
H	2.624428000	13.400573000	7.699150000
H	0.999947000	13.119717000	7.003614000
C	-0.490715000	11.792866000	9.231055000
H	-1.117734000	12.155276000	8.402222000
H	-1.051574000	11.834078000	10.175329000
H	-0.182171000	10.754799000	9.039050000
C	0.298252000	14.526927000	9.462604000
H	-0.373118000	14.705221000	8.608124000
H	1.118682000	15.259409000	9.446794000
H	-0.253797000	14.640743000	10.406027000

TS1

E(PBE0-D3(BJ)/def2-QZVPP+SMD): -2195.910206 E_h

E(TPSSh-D3(BJ)/def2-QZVPP+SMD): -2197.623433 E_h

ΔΔG(298): 0.539615 E_h

Lowest frequency: 163.04i cm^{-1}

Rh	2.297302000	12.570398000	11.325936000
P	3.260074000	12.934690000	13.374735000
N	4.680521000	10.676438000	11.446450000
B	3.345523000	10.890145000	10.841335000
C	4.099517000	12.610072000	10.391946000
H	4.254072000	13.345686000	9.593459000
C	5.139300000	11.764058000	10.674206000
P	1.059931000	12.850292000	9.361933000
C	6.520307000	11.918816000	10.127578000
H	6.885629000	11.024532000	9.603394000
H	7.234145000	12.141162000	10.938025000

H	6.540295000	12.763663000	9.426472000
C	4.424825000	8.594533000	12.644514000
H	3.604966000	8.233293000	12.008609000
H	3.994320000	9.159408000	13.482221000
H	4.949557000	7.719665000	13.054667000
Cl	0.179638000	12.823930000	12.444818000
C	5.395631000	9.458424000	11.840920000
C	5.889589000	8.604742000	10.663658000
H	5.048218000	8.302210000	10.025394000
H	6.370429000	7.691537000	11.045949000
H	6.635128000	9.128868000	10.050958000
C	6.580562000	9.820723000	12.740578000
H	7.082843000	8.907239000	13.093509000
H	6.240073000	10.381891000	13.623048000
H	7.329865000	10.424871000	12.211135000
C	2.581428000	9.819271000	9.956221000
C	1.467066000	9.125600000	10.489160000
C	0.761588000	8.219481000	9.687869000
H	-0.094873000	7.694181000	10.121629000
C	1.110689000	7.969219000	8.361161000
C	2.228434000	8.635167000	7.853983000
H	2.548265000	8.432576000	6.826787000
C	2.967598000	9.541858000	8.620999000
C	0.983733000	9.354957000	11.892084000
H	1.800208000	9.319654000	12.626652000
H	0.239336000	8.598876000	12.177770000
H	0.521405000	10.350129000	11.998121000
C	0.303994000	7.041996000	7.497268000
H	0.948588000	6.422260000	6.854267000
H	-0.366648000	7.610778000	6.830231000
H	-0.325015000	6.370288000	8.099790000

C	4.172927000	10.183404000	7.990220000
H	4.253628000	9.908502000	6.928933000
H	5.099259000	9.861287000	8.484677000
H	4.145137000	11.278784000	8.072181000
C	5.052480000	13.288344000	13.488315000
H	5.627900000	12.394546000	13.223355000
H	5.317552000	13.612457000	14.506106000
H	5.298405000	14.084508000	12.772888000
C	2.976573000	11.703895000	14.691757000
H	1.901788000	11.473257000	14.711719000
H	3.303799000	12.075915000	15.674407000
H	3.529311000	10.789258000	14.443030000
C	2.567862000	14.470385000	14.100579000
H	2.762199000	15.304644000	13.410610000
H	3.028159000	14.687684000	15.076859000
H	1.480466000	14.349569000	14.196945000
C	1.818202000	12.836615000	7.694765000
H	2.073780000	11.805253000	7.418874000
H	2.733534000	13.444202000	7.684740000
H	1.111207000	13.241834000	6.954244000
C	-0.415962000	11.807052000	9.122783000
H	-1.029135000	12.177106000	8.286776000
H	-0.992956000	11.832330000	10.057919000
H	-0.098519000	10.772419000	8.926040000
C	0.358665000	14.541715000	9.439559000
H	-0.290215000	14.749396000	8.574333000
H	1.177255000	15.276047000	9.469433000
H	-0.218010000	14.622391000	10.371539000

References

1. A. Van Der Ent, A. L. Onderdelinden and R. A. Schunn, in *Inorganic Syntheses*, **1990**, vol. 28, pp. 90-92.
2. W. Wolfsberger and H. Schmidbaur, *Syn. React. Inorg. Metal-Org. Chem.*, 1974, **4**, 149-156.
3. K. Issleib and A. Brack, *Z. anorg. allg. Chem.*, 1954, **277**, 258-270.
4. M. Schäfer, J. Schäfer, R. D. Dewhurst, W. C. Ewing, M. Krahfuß, M. W. Kuntze-Fechner, M. Wehner, C. Lambert and H. Braunschweig, *Chem. Eur. J.*, 2016, **22**, 8603-8609.
5. B. Liang, M. Dai, J. Chen and Z. Yang, *J. Org. Chem.*, 2005, **70**, 391-393.
6. G. Doisneau, G. Balavoine and T. Fillebeen-Khan, *J. Organomet. Chem.*, 1992, **425**, 113-117.
7. A. J. Arduengo, H. V. R. Dias, R. L. Harlow and M. Kline, *J. Am. Chem. Soc.*, 1992, **114**, 5530-5534.
8. T. Schaub, M. Backes and U. Radius, *Organometallics*, 2006, **25**, 4196-4206.
9. (a) C. Busetto, A. D'Alfonso, F. Maspero, G. Perego and A. Zazzetta, *J. Chem. Soc., Dalton Trans.*, 1977, 1828-1834; (b) H. Werner, J. Wolf, U. Schubert and K. Ackermann, *J. Organomet. Chem.*, 1983, **243**, C63-C70.
10. H. Braunschweig, K. Geetharani, J. O. C. Jimenez-Halla and M. Schäfer, *Angew. Chem. Int. Ed.*, 2014, **53**, 3500-3504.
11. G. Sheldrick, *Acta Cryst.*, 2015, **A71**, 3-8.
12. G. Sheldrick, *Acta Cryst.*, 2008, **A64**, 112-122.
13. F. Neese, *WIREs Comput. Mol. Sci.*, 2012, **2**, 73–78.
14. C. Adamo and V. Barone, *J. Chem. Phys.*, 1999, **110**, 6158–6170.
15. F. Weigend and R. Ahlrichs, *Phys. Chem. Chem. Phys.*, 2005, **7**, 3297.
16. D. Andrae, U. Häußermann, M. Dolg, H. Stoll and H. Preuß, *Theor. Chim. Acta*, 1990, **77**, 123–141.
17. S. Grimme, J. Antony, S. Ehrlich and H. Krieg, *J. Chem. Phys.*, 2010, **132**, 154104.
18. S. Grimme, S. Ehrlich and L. Goerigk, *J. Comput. Chem.*, 2011, **32**, 1456–1465.
19. F. Neese, *J. Comput. Chem.*, 2003, **24**, 1740–1747.
20. F. Neese, F. Wennmohs, A. Hansen and U. Becker, *Chem. Phys.*, 2009, **356**, 98–109.
21. (a) J. Tao, J. P. Perdew, V. N. Staroverov and G. E. Scuseria, *Phys. Rev. Lett.*, 2003, **91**, 146401; (b) V. N. Staroverov, G. E. Scuseria, J. Tao and J. P. Perdew, *J. Chem. Phys.*, 2003, **119**, 12129–12137.
22. A. V. Marenich, C. J. Cramer and D. G. Truhlar, *J. Phys. Chem. B*, 2009, **113**, 6378–6396.
23. (a) C. P. Kelly, C. J. Cramer, D. G. J. Truhlar, *J. Chem. Theory Comput*. 2005, **1**, 1133–1152. (b) M. Sparta, C. Riplinger, F. Neese, *J. Chem. Theory Comput*. 2014, **10**, 1099–1108.
24. C. Y. Legault, CYLview, version 1.0.561b; Université de Sherbrooke, Sherbrooke, QC, Canada, 2009; http://www.cylview.org.
25. 1 K. Ishida, K. Morokuma and A. Komornicki, *J. Chem. Phys.*, 1977, **66**, 2153–2156.
